# The Effect of Diabetes on Outcomes of Non‐Surgical Periodontal Therapy: A Systematic Review With a Meta‐Analysis and Trial Sequential Analysis

**DOI:** 10.1111/idh.70003

**Published:** 2025-12-18

**Authors:** L. P. M. Weijdijk, T. M. J. A. Thomassen, N. C. de Keyzer, E. E. J. Mayer, C. Valkenburg, G. A. van der Weijden, D. E. Slot

**Affiliations:** ^1^ Department of Periodontology Academic Center for Dentistry Amsterdam (ACTA) ACTA Is a Joint Venture Between the Faculty of Dentistry of the University of Amsterdam and the Faculty of Dentistry of the Vrije Universiteit Amsterdam Amsterdam the Netherlands; ^2^ Department of Oral and Maxillofacial Surgery, Amsterdam UMC and Academic Centre for Dentistry Amsterdam (ACTA) ACTA Is a Joint Venture Between the Faculty of Dentistry of the University of Amsterdam and the Faculty of Dentistry of the Vrije Universiteit Amsterdam Amsterdam the Netherlands

**Keywords:** diabetes, diabetes mellitus, periodontal therapy, periodontal treatment

## Abstract

**Objective:**

The aim of this systematic review was to critically assess and synthesise the current scientific evidence of the potential impact of diabetes mellitus (DM) on treatment outcomes in periodontitis patients following NSPT, incorporating recent advancements in meta‐analytical techniques.

**Methods:**

To identify eligible studies meeting this aim, a comprehensive search was conducted in MEDLINE‐PubMed and Cochrane‐CENTRAL from their inception until April 2024. The inclusion criterion for suitable studies was the availability of data for a group of patients with solely periodontitis and a group with both DM and periodontitis. Study parameters needed to include probing pocket depth (PPD) and clinical attachment level (CAL) as the primary outcomes of interest. As secondary study parameters, gingival indices, plaque indices, and gingival recession were considered. Data from all included studies were presented descriptively, and a meta‐analysis was conducted when quantitative methods were feasible.

**Results:**

Screening of the 3574 papers resulted in 32 eligible publications, which reported 30 unique studies. Meta‐analyses showed no differences of means in incremental changes from baseline to post‐NSPT between the DM and non‐diabetics (NDM) groups for CAL and PPD. Moreover, the secondary outcomes also revealed no significant differences regarding the response to therapy. Based on the Trial Sequential Analysis (TSA) of these meta‐analyses, the effect was found to be conclusive and reliable, indicating that additional data are unlikely to alter the summary effect.

**Conclusion:**

Based on the evidence profile, it can be stated with moderate certainty that the difference in treatment outcomes of periodontitis patients following NSPT between the DM and NDM groups is insignificant.

## 1 Introduction

Periodontitis is a chronic multifactorial inflammatory disease that leads to the destruction of tooth‐supporting structures [1]. Periodontitis ranks sixth among the world's most common diseases, affecting an estimated 11.2% of the global adult population [2]. It is characterised by irreversible damage to the root cementum, periodontal ligament, and alveolar bone, which can result in tooth loosening and eventual tooth loss [3]. The primary etiological factor initiating and advancing periodontitis is the dental plaque biofilm, an organised aggregation of microorganisms [4]. Treatment typically involves mechanical and/or ultrasonic debridement, known as non‐surgical periodontal therapy (NSPT). This includes removing supra‐ and subgingival bacterial plaque and calculus, alongside providing oral hygiene instructions to patients [5]. Due to the chronic nature of periodontitis, lifelong intensive supportive care is essential to prevent recurrence or further progression [6].

Studies have established a link between periodontitis and systemic conditions, including cardiovascular disease, respiratory disorders, rheumatoid arthritis, and diabetes mellitus.

(DM) [7, 8]. Individuals with DM face an elevated risk of developing and experiencing more severe periodontal disease [9, 10]. Moreover, the prevalence of DM is higher in populations with periodontitis compared to non‐diabetics (NDM) [11]. Evidence suggests a bidirectional relationship between DM and periodontitis [12]. Both diseases are chronic, inflammatory, and multifactorial, with inflammation playing a central role in their pathogenesis [13]. Hyperglycemia is the most commonly identified link between periodontitis and DM, as it purportedly not only increases the risk of developing periodontitis but also accelerates its progression, particularly in poorly controlled DM patients [14]. On the other hand, periodontitis is linked to a potential negative impact on glycemic control in DM patients [10, 15, 16, 17, 18].

It is projected that by 2035, approximately 592 million individuals will be diagnosed with DM globally [19], highlighting the need for a deeper understanding of its relationship with periodontitis. Recent findings from an umbrella review suggest that NSPT may contribute to improved glycemic control in type 2 DM [20]. However, despite the substantial evidence, only two systematic reviews (SRs) have examined the impact of DM on clinical periodontal outcomes following NSPT [9, 21]. The earlier SR, published in 2009 [9], necessitates updating given the emergence of various new studies in the field. A more recent SR from 2019 suggests that DM does not significantly affect short‐term treatment outcomes following NSPT, albeit with limitations in the parameters evaluated, particularly the absence of bleeding on probing (BOP), a critical indicator of gingival inflammation [22, 23]. Given that gingival inflammation is best assessed by BOP and plaque scores are essential secondary outcomes relative to the risk of periodontal diseases [23], it is imperative to include these. Moreover, most studies in the SR reported only short‐term clinical outcomes (up to 6 months), highlighting the need for more comprehensive, up‐to‐date investigations [22, 23]. In addition, new studies appeared, and modern techniques can be applied by performing a meta‐analysis.

For instance, by employing Trial Sequential Analysis (TSA), the balance between Type I and Type II errors can be assessed while the effect size is sufficiently robust to remain unaffected by additional studies. This method enhances the precision and reliability of evidence synthesis compared to traditional meta‐analysis techniques, particularly in determining the sufficiency of the available evidence.

Therefore, this SR aims to critically appraise and comprehensively synthesise the current scientific evidence of the potential impact of DM on treatment outcomes in periodontitis patients following NSPT, integrating recent advancements in meta‐analytical techniques.

## 2 Methods

The preparation and presentation of this SR are in accordance with the Cochrane Handbook for Systematic Reviews [24] and the guideline for meta‐analysis and systematic reviews of observational studies in epidemiology (MOOSE) [25]. See Appendices S9 and S10.1 and S10.2.

Following the initial discussion between the members of the research team, an *‘*a priori*’* protocol was developed using the Preferred Reporting Items for Systematic Review and Meta‐Analysis Protocols (PRISMA‐P) [26] (Appendix S10.1). This review was registered at the International Prospective Register of Systematic Reviews (PROSPERO) with the number CRD42021227543. The institutional review board of the Academic Centre of Dentistry in Amsterdam (ACTA) also provided approval under the following number: 2022‐43514. Further ethical approval was not required as this review involves analysis of previously published studies and does not involve primary data collection or human participants.

### 2.1 Focused Question

A precise review question was formulated utilizing the population, exposure, comparison, outcome, and type of study (PECO) framework as follows [27]:

– What is the potential impact of DM [exposure] on clinical treatment outcomes [outcome] in periodontitis patients undergoing NSPT [population] compared to periodontitis patients without DM (NDM) [comparison]?

This question is based on the supposition that DM is strongly associated with periodontal disease [15] and could therefore negatively influence the response to periodontal therapy.

### 2.2 Search Strategy

A structured search strategy was designed to retrieve all relevant studies that evaluated the clinical effects of DM patients compared to NDM periodontitis patients following NSPT. The search was designed by two reviewers (LPMW and DES). The National Library of Medicine, Washington, D.C. (MEDLINE‐PubMed) was searched from its initiation to March 2024 for appropriate papers that answered the focused question. Table 1 provides details regarding the search approach employed. No limitations were applied regarding language or publication date in the search engine's strategy. The reference lists of the studies included in this review were hand‐searched to identify additional potentially relevant studies. Additional grey literature was not sought nor examined.

**TABLE 1:** Search strategy used for PubMed‐MEDLINE.

**{<exposure**>**AND<outcome>}**
**<Exposure:>** {<(Glucose Metabolism Disorders [Mesh]) OR (Diabetes Mellitus [Mesh] OR (Diabetes Mellitus) OR Diabetes OR diabet* OR (glucose metabolism disorders)>
**<Outcome:>** <(Periodontitis [MeSH Terms] AND therapy) OR (periodontal therapy [MeSH Terms]) OR ((periodontal treatment)) OR (periodontal therapy)>}

*Note:* The asterisk (*) was used as a truncation symbol. The search strategy was customised according to the database being searched.

### 2.3 Screening and Selection

Titles and abstracts (when available) from all studies were independently screened, and two reviewers (LPMW and TMJAT) selected studies that potentially met the inclusion criteria. Only papers in the English language were selected.

Studies were categorised as ‘eligible’, ‘not eligible’, or ‘questionable’. This process was performed using the web tool Rayyan [28], which expedites the initial screening of abstracts and titles using a process of semi‐automation while incorporating a high level of usability. Disagreements concerning eligibility were resolved by consensus or, if disagreement persisted, by arbitration through a third reviewer (DES). The papers that fulfilled all the inclusion criteria were processed for data extraction.

The eligibility criteria were as follows:

– Any study type, either prospective or of observational design, in which periodontitis patients were compared and received non‐surgical periodontal therapy (NSPT)
– Conducted in humans who were:
  ○ ≥ 18 years of age
  ○ Diagnosed with mild to severe periodontitis
– Studies had to have at least two groups of patients:
  ○ Patients diagnosed with periodontitis without DM
  ○ Patients diagnosed with periodontitis and DM (or any synonym such as metabolic syndrome) either self‐reported or clinically assessed (based on the standard diagnostic criteria; e.g., fasting glucose HbA1c levels or a diagnosis of DM by a healthcare professional)
    * Type of DM: undefined, type I and/or type II.
– Treatment outcomes should include change over time:
  ○ Primary outcome: probing pocket depth (PPD) and clinical attachment level (CAL)
  ○ Secondary outcome: gingivitis indices, plaque indices and gingival recession (REC)

The exclusion criteria were as follows:

- Antibiotic intake
- Patients with solely dental implants
- Additional treatments (laser, alternative adjuvants such as vitamin D)
- Additional surgical therapy
- Other systemic diseases
- Gestational DM as well as prediabetes

The reasons for exclusion after full‐text reading were recorded (see Appendix S1). Thereafter, the selected full‐text papers that fulfilled the eligibility criteria were identified and included in this SR. They were also processed for data extraction, methodological quality assessment, and clinical, methodological, and statistical heterogeneity. Both descriptive and quantitative methods were used for analysis (for details see Appendices S5 and S6).

### 2.4 Methodological Quality Assessment

Two reviewers (LPMW and TMJAT) independently scored the individual methodological qualities of the included studies using a comprehensive combination of the critical appraisal checklist for analytical cross‐sectional studies, which was developed by the Joanna Briggs Institute [29], the Newcastle Ottawa scale adapted for cross‐sectional studies [30], and the Risk of Bias In Non‐randomised Studies—of Exposure (ROBINS‐E) tool [31], as reported by Van der Weijden et al. [32]. Judgement of risk of bias is presented according to the seven domains as suggested by the ROBINS‐E tool, which consists of:

1. Pre‐assessment domains: bias due to confounding, bias in the selection of study participants, and bias in the classification of exposure
2. Post‐assessment domains: bias due to deviations from the intended exposure, bias due to missing data, bias in the measurement of outcomes, and bias in the selection of the reported results

The judgements within each domain were carried forward to an overall risk of bias. A study was classified as having *a low risk* of bias when all domains were judged to have little risk of bias. *A moderate risk* of bias was assigned when one or more domains of the study were judged not to have a higher‐than‐moderate risk. A study was classified as having a *serious risk* of bias when one or more domains were scored as having serious risk. An overall *critical risk* of bias was scored when at least one domain was judged to be critical. The response option *‘no information’* was assigned if the study was judged to be at serious or critical risk of bias and there was a lack of information in one or more key domains [29, 30, 31, 32]. If there was a disagreement between the two reviewers, then a consensus was achieved through discussion. If disagreement persisted, then a third reviewer (DES) was consulted. This judgement was decisive.

#### 2.4.1 Data Extraction

Independent data extraction was performed by two reviewers (LPMW and TMJAT) utilising a specially designed standardised data extraction form. From the eligible papers, details on study design, demographics, DM diagnosis, NSPT, and clinical treatment outcomes were extracted. If DM regulation was not clearly differentiated for good and poorly controlled, HbA1c ≥ 7% levels were used to categorise the DM population. If the identified studies had multiple groups of subjects, only the groups fitting the selection criteria were included. Disagreement between the reviewers was resolved through discussion and consensus. If disagreement persisted, then the judgement of a third reviewer (DES) was decisive.

#### 2.4.2 Outcomes

Clinical treatment outcomes were determined as primary and secondary outcomes:

– Primary outcomes were determined as follows (for details see Appendix S2.1):
  ○ PPD: periodontal pocket depth as measured from the gingival margin to the base of the periodontal pockets with the tip of the periodontal probe and expressed in mm [33].
  ○ CAL: clinical attachment level as measured with a periodontal probe from the cement‐enamel joint (CEJ), or Relative Attachment Level (RAL) using a customised stent to the base of the pocket and expressed in mm [34].
– Secondary outcomes included the following indices, but are not limited to:
  ○ Gingivitis indices:
    * BOP: Bleeding on Probing following Ainamo and Bay 1975 [28].
    * GI: Gingival Index following Löe & Silness 1963 [31].
  ○ Plaque indices:
    * PI: Plaque index following O'Brien et al. 1972 [35]
    * PI: Plaque Index following Silness and Löe 1964 [36].
  ○ Gingival recession (REC): gingival recession as measured clinically from the cementoenamel junction to the free gingival margin using a periodontal probe. It reflects the exposure of the root cementum and is measured in mm [37, 38].

Details on additional indices which are included in the review can be found in the Appendix S2.2.

For all outcome parameters, their means and standard deviation (SD) at baseline and follow‐up were considered. When the SE was given, the following was used to calculate the SD = SE × √*N*. If outcomes at multiple time points were reported, only the outcomes with the longest follow‐up period but within 1 year were extracted. This was done to standardise treatment outcomes across studies and minimise variability from longer follow‐up periods, which may involve post‐treatment care or maintenance interventions, focusing on the immediate effect of NSPT. For those papers that provided insufficient data to be included in this paper, the first or corresponding author was contacted to request additional data.

#### 2.4.3 Assessment of Clinical and Methodological Heterogeneity

The factors utilised to assess the clinical heterogeneity of the various study outcomes were as follows: characteristics of participants (age, gender, and continent), DM type (I or II), level of DM (controlled, uncontrolled, or undefined), and details regarding NSPT method (manual and/or ultrasonic). Factors employed to assess the methodological heterogeneity were diversity in study design details, subject characteristics, evaluation period, side effects, and study funding. When clinical or methodological heterogeneity was presented across studies, sources of heterogeneity were investigated with subgroup or sensitivity analyses [24]. Factors that were potentially relevant for subgroup analysis were DM‐related details (type, duration, and regulation).

#### 2.4.4 Analysis

As a summary, a descriptive data presentation was utilised for all studies. A meta‐analysis was performed if two or more studies presenting data on the same parameter could be included and if the individual studies provided an SD of the mean results. Thereafter, the difference of means (DiffM) as well as the associated 95% confidence interval and *p*‐values for each parameter of interest was calculated between the two groups. As a guide for interpreting the results, a clinical relevance scale as presented by Smiley et al. (2015) for interpreting the mean differences for CAL was used [39]. Values of 0–0.2, > 0.2–0.4, > 0.4–0.6, and > 0.6 were respectively classified as zero effect, small effect, moderate effect, and substantial effect. *p*‐values ≤ 0.05 were considered to be significant.

Analysis was performed utilising Review Manager version 5.3 [40]. For a subsequent subgroup analysis, a meta‐analysis was performed if more than one study could be included.

The authors of this SR anticipated that there would be considerable heterogeneity among the included studies, as study designs and details presumably differ [11]. This variance was taken into consideration by primarily utilising the random‐effects model, the exception being when less than four studies were eligible for meta‐analysis. Otherwise, the fixed‐effects model was utilised, as done by the Cochrane Oral Health Group [24].

Sensitivity analyses were undertaken to evaluate the effect of excluding studies based on specific aspects in the domain of clinical or methodological heterogeneity. Testing for publication bias per outcome was conducted, as proposed by Egger et al. [41]. If the meta‐analysis involved a sufficient number of trials to make a visual inspection of the funnel plot meaningful (a minimum of 10 trials), then these plots were employed as tools to assess publication bias. The presence of asymmetry in the inverted funnel was regarded as suggestive of publication bias [24, 42, 43]. As planned, *‘*a priori*’*, relative to DM status, a subgroup analysis was conducted. Subgroup analyses were also performed for pockets ≥ 7 mm and geographical regions.

Trial sequential analysis (TSA) was applied to assess the robustness and reliability of the cumulative evidence and to reduce the risk of type I error. The required information size (RIS) and the trial sequential monitoring boundaries (TSMB) for benefit or futility were calculated. The RIS was calculated based on a type I error risk of α = 5% and a type II error risk of *β* = 0.20, with a statistical test power of 80%. RIS accounted for heterogeneity and multiple comparisons. The Lan‐DeMets version [44] of the O'Brien‐Fleming function [45] was used for calculating the TSMBs. TSA software version 0.9.5.10 Beta (Copenhagen Trial Unit, Copenhagen, Denmark) was used [46, 47, 48, 49, 50].

#### 2.4.5 Assessment of Statistical Heterogeneity

Statistically, heterogeneity was tested by the chi‐square test and *I* ^2^ statistic. A chi‐square test resulting in *p* < 0.1 was considered an indication of significant statistical heterogeneity. As a rough guide to assessing the possible magnitude of inconsistency across studies, an *I* ^2^ statistic of 0%–40% was interpreted to indicate unimportant levels of heterogeneity. An *I* ^2^ statistic of 30%–60% may represent moderate heterogeneity, and an *I* ^2^ statistic of 50%–90% may represent substantial heterogeneity. An *I* ^2^ statistic of greater than 75% was interpreted to indicate considerable heterogeneity and was further assessed with subgroup or sensitivity analysis [51].

### 2.5 Grading the ‘Body of Evidence’

Two reviewers (LPMW and DES) rated the quality of the evidence and the strength of the recommendations according to the following aspects: study limitations, inconsistency of results, indirectness of evidence, imprecision, and publication bias by utilizing the grading of recommendations assessment, development, and evaluation (GRADE) framework [52], which provides a systematic approach for considering and reporting each of these factors. An overall rating of confidence in effect estimates was considered critical for the final recommendation [53]. Any disagreement between the two reviewers was resolved after additional discussion. If a disagreement persisted, then the judgment of a fourth reviewer (GAW) was decisive.

## 3 Results

### 3.1 Search and Selection Process

**FIGURE 1:**
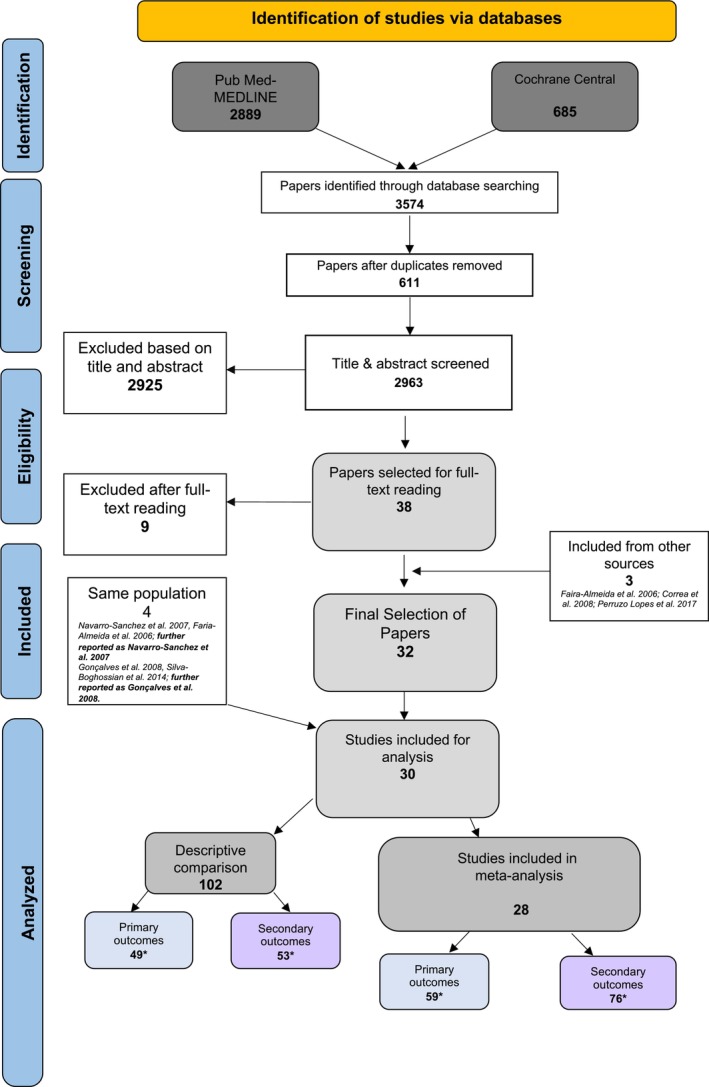
Search and selection results. *Comparisons.

Figure 1 illustrates the search process. A total of 2963 unique articles were identified. The screening of titles and abstracts initially resulted in 38 papers. After full‐text reading, 9 studies were excluded because they did not meet all the eligibility criteria. Reasons for exclusion are found in Appendix S1. Hand‐searching of the reference lists revealed 3 additional suitable systematic reviews. Consequently, 32 papers were identified which reported on 30 unique studies, as data from the paper of *Navarro‐Sanchez* et al. [54] *and Faria‐Almeida* et al. [55] (*further reported as Navarro‐Sanchez* et al. *and Gonçalves* et al. [56] *and Silva‐Boghossian* et al. [57]; *further reported as Gonçalves* et al. [56] concern the same study population). An overview of the included study IDS [54, 56, 58, 59, 60, 61, 62, 63, 64, 65, 66, 67, 68, 69, 70, 71, 72, 73, 74, 75, 76, 77, 78, 79, 80, 81, 82, 83, 84, 85] and their characteristics are presented in Table 2.

**TABLE 2:** Overview of included studies processed for data extraction.

Selection ID, authors, year, country, RoB	*N* subjects, type of population, follow up	Gender (*N* males/females), mean age (SD), range in years	Intervention	Original parameters	Original authors conclusion
I Tervonen et al. 1991 Finland Rob: critical	*N*: 79 ◊ **DM** Diabetes clinic **NDM** Occupational Health service Follow up: 3–4 months	**DM type I/II** *N*: 34 ♀: 10 ♂: 24 Mean age: ? Range: 18–51 DM duration: 8.9 years (1.0) **NDM** *N*: 45 ♀: 14 ♂: 31 Mean age: ? Range: 18–51	NSPT: SRP (mi, usi) Patient motivation, oral hygiene instructions, replacement of emergency restorations The number of appointments varied between the patients depending on individual treatment need	BOP, PD	No significant difference could be observed in the response to NSPT treatment between diabetics and controls in either the decrease in gingival bleeding or the healing of periodontal pockets
II Tervonen et al. 1997 Finland Rob: serious	*N*: 46 ◊ **DM** Diabetes clinic and hospital **NDM** Dental health care center clinic Follow up: 4 weeks, 6 months and 12 months	**DM type I** *N*: 36 ♀: ? ♂: ? Mean age: 29.4 (3.7) Range: 24–36 DM duration: 16.9 years (6.7) **NDM** *N*: 10 ♀: ? ♂: ? Mean age: 30.1 (3.8) Range: ?	NSPT: SRP Oral hygiene instruction and repeated when needed, removal of plaque retentive overhangs of fillings, restorative caries treatment.	PI, calculus, PD, BOP, AL	No statistically differences could be observed between the diabetics and healthy controls at any examination.
III Christgau et al. 1998 Germany Rob: moderate	*N*: 40 ◊ **DM** Moderate to advanced periodontitis Referred patients to the Endocrine Department of internal medicine **NDM** Moderate to advanced periodontitis Referred patients to the operative dentistry and periodontology Follow up: 2 weeks, 4 months	**DM type I + II** *N*: 20 ♀: 8 ♂: 12 Median age: 54.5 Range: 30–66 DM duration (median): 11.5 years **NDM** *N*: 20 ♀: 12 ♂: 8 Median age: 50.5 Range: 30–67	NSPT: SRP (mi + la, antiseptic) Pretreatment phase: patient motivation, oral hygiene instructions, supragingival scaling, placement of emergency restorations and removal of overhanging cervical crown margins, extractions	API, PBI, BOP, PPD, PAL	Patients with well controlled diabetes mellitus might respond to non‐surgical periodontal therapy similarly well as healthy controls
IV Sonoki et al. 2006 Japan Rob: serious	*N*: 11 ◊ **DM** Moderate to severe periodontitis Dental college department of periodontology **NDM** Moderate to severe periodontitis Dental college department of periodontology Follow up: 5.5–6.5 months	**DM type II** *N*: 5 ♀: 1 ♂: 4 Mean age: 60.4 (4.5) Range: ? DM duration: ? **NDM** *N*: 6 ♀: 4 ♂: 2 Mean age: 49.2 (3.2) Range: ?	NSPT: SRP Oral hygiene instruction, extractions, treatment of caries, root canal or other treatments Once weekly for supragingival scaling of one sextant for 6 weeks followed directly by subgingival scaling and rootplaning of one sextant for 6 weeks Periodontal maintenance therapy at 1 month interval (usi)	BOP, PD	Periodontal parameters improved in both groups
V Navarro‐Sanchez et al. 2007 Spain Rob: moderate	*N*: 20 **DM** Moderate generalised chronic periodontitis Referred patients periodontal clinic school of Dentistry **NDM** Moderate generalised chronic periodontitis Referred patients periodontal clinic school of Dentistry Follow up: 3, 6 months	**DM type II** *N*: 10 ♀: 2 ♂: 8 Mean age: 57.4 (6.1) ♦ Range: ? DM duration: 12 years **NDM** *N*: 10 ♀: 7 ♂: 3 Mean age: 56.4 (7.8) ♦ Range: ?	NSPT: SRP (mi + la) Pretreatment phase: provision of information/instructions on periodontal disease and oral hygiene, and supragingival prophylaxis Oral hygiene instruction, four 1‐h session of scaling and rootplaning over a maximum 4‐week period, supragingival prophylaxis	PI, BOP, PD, REC, CAL	**Navarro‐Sanchez et al. [54]** Short‐term periodontal healing after non‐surgical periodontal treatment is similar between type 2 diabetic and non‐diabetic periodontal patients, both groups showed a significant improvement in clinical periodontal status, and the presence of DM does not appear to have a major effect on the success of periodontal therapy **Faria‐Almeida et al. [55]** Both patient groups improved clinically after basic non‐surgical periodontal treatment. Among the clinical variables studied, only probing depth showed a statistically significant difference between the groups after periodontal treatment between baseline and 3 and 6 months
VI Da Cruz et al. 2008 Brazil Rob: serious	*N*: 20 **DM** Generalised chronic periodontitis Clinic Dental School **NDM** Generalised chronic periodontitis Clinic Dental School Follow up: 3 months	**DM type II** *N*: 10 ♀: ? ♂: ? Mean age: 47.10 (13.01) Range: 30–70 DM duration: 18.10 years (11.37) **NDM** *N*: 10 ♀: ? ♂: ? Mean age: 45.60 (7.24) Range: 30–70	NSPT: SRP (la) Pretreatment phase: supragingival biofilm was removed Scaling and rootplaning in one session, 2 h Oral hygiene instructions, plaque control at 2‐week interval during 3 months	PI, GI, PD, REC, CAL	Non‐surgical periodontal treatment using full‐mouth root planing did not provide a significant difference in clinical responses between DM and NDM groups after 3 months of follow‐up
VII Correa et al. 2008 Brazil Rob: moderate	*N*: 49 ◊ **DM** Chronic periodontitis Clinic of periodontics, School of Dentistry **NDM** Chronic periodontitis Periodontal clinic Follow up: 3 months	**DM type II** *N*: 23 ♀: 14 ♂: 9 Mean age: 47.5 (7.2) Range: 32–60 ♦ DM duration: 9.96y (6.78) **NDM** *N*: 26 ♀: 12 ♂: 14 Mean age: 41.6 (7.1) Range: 30–60 ♦	NSPT: SRP (mi + la) Manual scaling and rootplaning with anaesthesia in four sessions Oral hygiene instructions Plaque control twice a month for 3 months consisting of supragingival plaque removal and reinstruction of oral hygiene procedures	VPI, GBI, PD, CAL, BOP	Non‐surgical periodontal treatment improves the clinical periodontal status
VIII Gonçalves et al. 2008 Brazil Rob: moderate	*N*: 40 **DM** Chronic periodontitis Clinic of Periodontics for Diabetic Patients, School of Dentistry **NDM** Chronic periodontitis Clinic for Periodontics, School of Dentistry Follow up: 3 months	**DM type II** *N*: 20 ♀: 12 ♂: 8 Mean age: 45.80 (6.01) Range: 30–60 DM duration: 9.85 (7.10) **NDM** *N*: 20 ♀: 10 ♂: 10 Mean age: 43.65 (6.01) Range: 30–60	NSPT: SRP (la) Scaling and rootplaning (1 h) Oral hygiene instructions, biofilm control Plaque control programme twice a month during 3 months consisting of supragingival plaque removal and reinstructions of oral hygiene procedures	VPI, GBI, PD, CAL, BOP, presence of suppuration	**Gonçalves et al. [56]** In both groups, the periodontal therapy was effective in improving most clinical parameters **Silva‐Boghossian et al. [57]** SRP associated with a rigorous maintenance pro‐ gram improved the periodontal status at 3 months evaluation in individuals with DM2 and inadequate metabolic control compared with systemically healthy individuals
IX Dağ et al. 2009 Turkey Rob: serious	*N*: 45 **DM** Chronic periodontitis Department of Endocrinology **NDM** Chronic periodontitis Department of Periodontology, Faculty of Dentistry Follow up: 3 months	**DM type II (controlled/un‐controlled)** *N*: 30 ♀: 12 ♂: 18 Mean age: 52.67 ◊ Range:? DM duration: 7.23 ◊ Poorly controlled *N*: 15 ♀: 10 ♂: 5 Mean age: 53.13 (8.47) Range:? DM duration: 7.33 (2.76) Good controlled *N*: 15 ♀: 8 ♂: 7 Mean age: 52,20 (7.67) Range:? DM duration: 7.13 (1.84) **NDM** *N*: 15 ♀: 7 ♂: 8 Mean age: 49.5 (7.61) Range:?	NSPT: SRP (mi, usi) Pretreatment phase: oral hygiene instructions, placement of emergency restorations and extraction of non‐salvageable teeth After the therapy, patients did not receive periodontal interventions for 3 months	PI, GI, PD, GBI, CAL	Poorly controlled and well‐controlled diabetics might respond to non‐surgical periodontal therapy as well as non‐diabetic patients
X Kardeşler et al. 2009 Turkey Rob: serious	*N*: 40 **DM** Chronic periodontitis Department of Metabolic Diseases and Endocrinology, School of Medicine **NDM** Chronic periodontitis Periodontology Clinic, School of Dentistry Follow up: 1, 3 months	**DM type II (controlled/un‐controlled)** *N*: 25 ♀: 18 ♂: 7 Mean age: 52.88 ◊ Range: 44–64 DM duration: 7.38 ◊ Poorly controlled *N*: 12 ♀: 5 ♂: 7 Mean age: 50.25 (6.30) Range: 44–63 DM duration: 7.5 (4.40) Good controlled *N*: 13 ♀: 2 ♂: 11 Mean age: 55.31 (5.44) Range: 44–64 DM duration: 6.69 (4.48) **NDM** *N*: 15 ♀: 6 ♂: 9 Mean age: 51.31 (8.64) Range: 34–67	NSPT: SRP Oral hygiene instructions and supragingival scaling and rootplaning once a week for 4 weeks On recall visits at 1 and 3 months supragingival plaque was removed and reinstructions were given	PI, PD, CAL BOP	Patients with type 2 diabetes and chronic periodontitis exhibited similar clinical periodontal improvements as their systemically healthy counterparts. Even patients in the poorly controlled group showed clinical outcomes similar to those of systemically healthy patients with periodontitis
XI Kudva et al. 2010 India Rob: serious	*N*: 30 ◊ **DM** Moderate to severe periodontitis Diabetic Care and Research Centre **NDM** Moderate to severe periodontitis Diabetic Care and Research Centre Follow up: 3 months	**DM type II** *N*: 15 ♀:? ♂:? Mean age:? Range: 40–60 DM duration:? **NDM** *N*: 15 ♀:? ♂:? Mean age:? Range: 40–60	NSPT: SRP (mi, usi) Scaling and rootplaning in a single setting	PI, GI, PD	Both groups showed an improvement in clinical parameters assessed at 3 months after nonsurgical periodontal therapy
XII Hungund et al. 2012 India Rob: serious	*N*: 30 **DM** Moderate generalised periodontitis Out‐patient department, Hospital **NDM** Moderate generalised periodontitis Out‐patient department Periodontology, Dental College Follow up: 3, 6 months	**DM type II** *N*: 15 ♀: 9 ♂: 6 Mean age: 50.46 Range:? DM duration:? **NDM** *N*: 15 ♀: 6 ♂: 9 Mean age: 40.73 Range:?	NSPT: SRP (mi + la) Pretreatment phase: provision of information/instructions on periodontal disease, oral hygiene and supra‐gingival prophylaxis Pretreatment phases were repeated at follow up examinations on 3 and 6 months	PI, BI, GI, PPD	Both patients groups improved clinically, after basic non‐surgical periodontal treatment. A statistically significant improvement in clinical variables was found in both groups between baseline to 3 and 6 months (*p* < 0.05). The control group showed a greater reduction in probing depth, as a result of periodontal treatment
XIII Cirano et al. 2012 Brazil Rob: moderate	*N*: 31 **DM** Generalised severe chronic periodontitis Graduate clinic, University **NDM** Generalised severe chronic periodontitis Graduate clinic, University Follow up: 3, 6 months	**DM type II (uncontrolled)** *N*: 16 ♀:? ♂:? Mean age: 56.1 (11.7) Range:? DM duration:? **NDM** *N*: 15 ♀:? ♂:? Mean age: 54.7 (9.8) Range:?	NSPT: SRP (usi) One session with a time limit of 45 min using an ultrasonic scaler and subgingival tips	PI, BS, CAL, PD, REC	Full mouth ultrasonic debridement promotes clinical improvements in patients with type 2 uncontrolled diabetes and generalised severe chronic periodontitis
XIV Camargo et al. 2013 Brazil Rob: serious	*N*: 20 ◊ **DM** Generalised chronic periodontitis Graduate Clinic **NDM** Generalised chronic periodontitis Graduate Clinic Follow up: 3 m	**DM type II** *N*: 10 ♀:? ♂:? Mean age: 52 (8.72) Range: 30–65 DM duration: 6.6y (4.17) **NDM** *N*: 10 ♀:? ♂:? Mean age: 41.6 (7.55) Range: 30–65	NSPT: SRP (la) Full‐mouth scaling and rootplaning under local anaesthesia in a single session Oral hygiene instructions Plaque control every 2 weeks interval during 3 months	PD, REC, CAL, PI, GI	Periodontal therapy was effective to control the periodontal disease. Significant improvement of periodontal status was found in both groups DM and NDM after 3 months, but no significant changes were found between DM and NDM groups for gain of attachments
XV Buzinin et al. 2014 Malaysia Rob: moderate	*N*: 41 **DM** Moderate to advanced periodontitis **NDM** Moderate to advanced periodontitis Follow up: 2, 3 months	**DM type I** *N*: 20 ♀: 12 ◊ ♂: 8 ◊ Mean age: 37.45 (14.38) Range:? DM duration:? **NDM** *N*: 21 ♀: 14 ◊ ♂: 7 ◊ Mean age:? Range:?	NSPT: SRP (mi, usi) Oral hygiene instructions, full mouth debridement in a single session using manual instruments combined with ultrasonic scaling	VPI, GBI, PPD, PAL	Both DM and NDM treatments produced similar improvements in clinical parameters from baseline to 3 months after treatment. However, there were no statistically significant difference in periodontal parameters between groups (*p* > 0.05) at any point of time
XVI López et al. 2013 Chile Rob: moderate	*N*: 52 ◊ **DM** Chronic periodontitis Public Health Center, Dental Center, Hospital **NDM** Chronic periodontitis Public Health Center, Dental Center, Hospital Follow up: 3, 6, 9 months	**DM type II** *N*: 26 ♀: 18 ♂: 8 Mean age: 58.9 (8.9) Range: 47–67 DM duration:? **NDM** *N*: 26 ♀: 19 ♂: 7 Mean age: 55 (7.6) Range: 45–70	NSPT: SRP (mi) Pretreatment phase: extractions of hopeless teeth and emergency restorative treatments of caries lesions Oral hygiene instructions, supragingival scaling using an ultrasonic scaler and crown polishing At each recall visit oral hygiene is checked and reinforced and dental prophylaxis were performed	PD, BOP, CAL, PI	Routine prophylaxes every 3 months significantly improve periodontal health and prevent progression of chronic periodontitis in poorly controlled and well‐controlled T2DM
XVII Kara et al. 2015 Turkey Rob: moderate	*N*: 30 **DM** Chronic periodontitis Department of Metabolic Disease, School of Medicine **NDM** Chronic Periodontitis Periodontology department, Faculty of Dentistry Follow up: 1, 3 m	**DM type II** *N*: 15 ♀: 7 ♂: 8 Mean age: 42.8 (7.40) Range: 34–58 DM duration: 6.93 (3.66) **NDM** *N*: 15 ♀: 10 ♂: 5 Mean age: 47.73 (7.08) Range: 33–56	NSPT: SRP (mi, usi) Scaling and rootplaning in 2 sequential visits in 7 days using the combination of hand instruments and ultrasonic devices No adjunctive therap Oral hygiene instructions, supragingival scaling were performed during follow‐up monthly	PI, GI, PD, BOP, CAL	The improvement of periodontal parameters showed that T2DM patients can benefit from periodontal therapy, as well as non‐diabetic patients with periodontitis
XVIII Kaur et al. 2015 India Rob: low	*N*: 75 ◊ **DM** Moderate or severe chronic periodontitis Department of Periodontics and Oral implantology, Institute of Dental Science **NDM** Moderate or severe chronic periodontitis Department of Periodontics and Oral implantology, Institute of Dental Science Follow up: 3, 6 m	**DM type II (controlled/un‐controlled)** *N*: 50 ◊ ♀: 28 ◊ ♂: 22 ◊ Mean age: 51.82 ◊ Range:? DM duration: 8.57 ◊ Poorly controlled *N*: 27 ♀: 15 ♂: 12 Mean age: 50.96 (6.43) Range:? DM duration: 8.41 (6.25) Good controlled *N*: 23 ♀: 10 ♂: 13 Mean age: 52.83 (5.04) Range:? DM duration: 8.76 (6.71) **NDM** *N*: 25 ♀: 10 ♂: 15 Mean age: 51.56 (5.91) Range:?	NSPT: SRP (mi, usi) Oral hygiene instructions, four SRP sessions over a maximum of 2 weeks manual and ultrasonic. Oral hygiene instructions were reviewed at each visit and additional supportive SRP was done when necessary	PI, GI, PPD, CAL, PESA, PISA, BOP	Nonsurgical periodontal therapy improved periodontal health in patients with type 2 diabetes. However, patients with poor baseline glycemic control had less clinical improvement than those without diabetes and those with good glycemic control
XIX Pannicker et al. 2015 India Rob: moderate	*N*: 70 ◊ **DM** Chronic periodontitis Department of Periodontics **NDM** Chronic periodontitis Department of Periodontics Follow up: 6 weeks	**DM type II** *N*: 35 ♀: 16 ♂: 19 Mean age: 43.29 (8.40) Range: 30–60 DM duration:? **NDM** *N*: 35 ♀: 20 ♂: 15 Mean age: 44.09 (6.87) Range: 30–60	NSPT: SRP (mi, usi) Scaling and rootplaning Monitoring oral hygiene weekly	PI, GI, PD, CAL	A significant reduction in plaque index, gingival index, probing pocket depth and clinical attachment level in both groups occurred at 3 weeks, compared with baseline
XX Doğan et al. 2016 Turkey Rob: low	*N*: 40 ◊ **DM** Chronic periodontitis Department of Periodontology, Faculty of Dentistry **NDM** Chronic periodontitis Department of Periodontology, Faculty of Dentistry Follow up: 6wk	**DM type II** *N*: 20 ♀: 9 ♂: 11 Mean age: 47.35 (4.97) Range:? DM duration: 4.05 (0.76) **NDM** *N*: 20 ♀: 10 ♂: 10 Mean age: 49.05 (4.71) Range:?	NSPT: SRP (mi, usi + la) Scaling and rootplaning via the use of manual scalers and curets with local anaesthetic twice per week, every appointment lasted 45–60 min	PI, GI, PD, CAL, BOP	Mean probing depth, clinical attachment level, bleeding on probing, plaque index and gingival index levels were significantly lower among both groups after non‐surgical periodontal therapy
XXI Mishra et al. 2016 India Rob: moderate	*N*: 28 ◊ **DM** Chronic periodontitis Department of Periodontics and Oral Implantology, College of Dental Science and Hospital **NDM** Chronic Periodontitis Department of Periodontics and Oral Implantology, College of Dental Science and Hospital Follow up: 1 months	**DM type II** *N*: 14 ♀: 7 ♂: 7 Mean age: 48.07 (5.54) Range: 30–65 DM duration:? **NDM** *N*: 14 ♀: 6 ♂: 8 Mean age: 41.71 (8.06) Range: 30–65	NSPT: SRP Scaling and rootplaning	GI, PI, PPD	At the end of 1 month period the scores decreased significantly compared to the pre‐treatment values in both chronic periodontitis group and chronic periodontitis with diabetes mellitus group, but on inter‐group comparison it remained statistically insignificant
XXII Abreu et al. 2015 Brazil Rob: moderate	*N*: 60 ◊ **DM** Chronic periodontitis **NDM** Chronic periodontitis Follow up: 1 month	**DM type II** *N*: 30 ♀: 15 ♂: 15 Mean age: 55.43 (8.56) Range: 38–68 ♦ DM duration: 5 years ♦ **NDM** *N*: 30 ♀: 14 ♂: 16 Mean age: 44.47 (9.17) Range: 21–64 ♦	NSPT: SRP Four to six sessions of scaling and rootplaning Pretreatment phase: oral hygiene instructions, oral prophylaxis and supragingival scaling Post‐treatment phase for a period of 4wk: weekly plaque control (oral hygiene instructions, supragingival scaling and prophylaxis)	PD, CAL, BOP, PI	Both groups (chronic periodontitis and chronic periodontitis with diabetes mellitus) showed a significant improvement of the parameters, apart from clinical attachment level, which improved but not statistically after periodontal therapy in diabetics with chronic periodontitis
XXIII Perruzo Lopes et al. 2017 Germany Rob: moderate	*N*: 52 ◊ **DM** Chronic periodontitis Invited by radio and TV broadcast and treated at a dentistry clinic **NDM** Chronic periodontitis Invited by radio and TV broadcast and treated at a dentistry clinic Follow up: 6 months	**DM type I + II** *N*: 41 ◊ ♀: 20 ◊ ♂: 21 ◊ Mean age: 53.70 ◊ Range:? DM duration:? DM type I *N*: 14 ♀: 5 ♦ ♂: 9 ♦ Mean age: 42.5 (16.8) Range:? DM duration:? DM type II *N*: 27 ♀: 15 ♦ ♂: 12 ♦ Mean age: 59.5 (9.7) Range:? DM duration:? **NDM** *N*: 11 ♀: 6 ♦ ♂: 5 ♦ Mean age: 44.7 (12.7) Range:?	NSPT: SRP Instruction and motivation about oral hygiene, supra‐and subgingival scaling, root planing and coronal polishing. Patients received reinforcement of oral hygiene and maintenance periodontal therapy 2 g amoxicillin as antibiotic prophylaxis 1 h before the appointment was prescribed for diabetic patients	PI, BOP, PPD, CAL	Periodontal treatment is effective in controlling inflammation in patients with diabetes mellitus 1 and diabetes mellitus 2
XXIV Sundaram et al. 2017 India Rob: serious	*N*: 120 **DM** Outpatient clinic of the department of periodontics, Dental College and Hospital **NDM** Outpatient clinic of the department of periodontics, Dental College and Hospital Follow up: 3 months	**DM type II (controlled/un‐controlled)** *N*: 80 ◊ ♀:? ♂:? Mean age:? Range:? DM duration:? Poorly controlled *N*: 40 ♀:? ♂:? Mean age:? Range:? DM duration:? Well controlled *N*: 40 ♀:? ♂:? Mean age:? Range:? DM duration:? **NDM** *N*: 40 ♀:? ♂:? Mean age:? Range:?	NSPT: SRP (la) Scaling and rootplaning under local anaesthesia, oral hygiene instructions Professional plaque programme (twice a month for 3 months) and reinstructions of oral hygiene procedures	PI, GI, BOP, PD, CAL	A statistically significant reduction in all the clinical parameters within the groups was found except for the CAL in non‐diabetic patients. The subjects with DM had significantly more clinical attachment loss than nondiabetic subjects
XXV Gayathri et al. 2019 India Rob: critical	*N*: 60 **DM** ? **NDM** ? Follow up: 4 weeks	**DM type II** *N*: 30 ♀: 16 ♂: 14 Mean age:? Range: 31–70 DM duration:? **NDM** *N*: 30 ♀: 12 ♂: 18 Mean age:? Range: 31–70	NSPT: SRP Oral hygiene instructions, scaling and rootplaning. Recalled after 4 weeks	PPD CAL GI	Initial periodontal therapy seems to be beneficial in reducing serum NO levels along with periodontal parameters in CP patients with or without T2DM
XXVI Ahuja et al. 2019 India Rob: moderate	*N*: 60 DM: Outpatient Department of Periodontics and Implantology, Dental College and Research NDM: Outpatient Department of Periodontics and Implantology, Dental College and Research Follow up: 6 months	**DM type II** *N*: 30 ♀: 16 ♂: 14 Mean age: 41.33 (11.87) ♦ Range: 24–60 ♦ DM duration:? **NDM** *N*: 30 ♀: 13 ♂: 17 Mean age: 52.47 (8.23) ♦ Range: 39–72 ♦	NSPT: SRP Scaling and rootplaning, oral hygiene instructions. Recall visits at 3 and 6 months, supragingival plaque and oral hygiene re‐instructions	PPD CAL PI GI	NSPT was effective in improving clinical parameters, increasing GCF, reducing serum leptin levels, and also improving glycemic status in patients with CP and CP with T2DM
XXVII Pragada et al. 2019 India Rob: serious	*N*: 60 DM: Department of Periodontics, Dental College and Hospital NDM: Department of Periodontics, Dental College and Hospital. Follow up: 6 weeks	**DM type II** *N*: 30 ♀:? ♂:? Mean age:? Range: 30–60 DM duration:? **NDM** *N*: 30 ♀:? ♂:? Mean age:? Range: 30–60	NSPT: SRP (usi, mi + la) Scaling and rootplaning, oral hygiene instructions	PPD CAL GI PI	A decline in the values for all the parameters when evaluated with baseline values 6 weeks after the periodontal treatment, once again substantiating the significance of phase‐I therapy
XXVIII Almeida et al. 2019 Brazil Rob: low	*N*: 54 ◊ DM: Referred patients to the Dental Clinic of the Federal University NDM: Referred patients to the Dental Clinic of the Federal University Follow up: 12 months	**DM type II** *N*: 26 ♀: 15 ♂: 11 Mean age: 57.9 (8.1) ♦ Range: 36–69 ♦ DM duration: 7.7 years **NDM** *N*: 28 ♀: 21 ♂: 7 Mean age: 44.8 (11.7) ♦ Range: 35–75 ♦	NSPT: SRP (mi, usi) Pre‐treatment (removal of plaque and calculus, extraction, provisional restoration, and filling overhang removal), scaling and rootplaning, CHX solution, pocket irrigation, oral hygiene instructions. Supportive post‐therapy of plaque control, re‐instructions, subgingival debridement (at 3, 6, 9 and 12 months)	PPD CAL PI BOP	Type 2 diabetic subjects and systemically healthy subjects with mild‐to‐moderate periodontitis responded similarly to the proposed FMD protocol for up to 1 year
XXIX Mirnic et al. 2022 Servie Rob: serious	*N*: 92 DM: Hospital patients who were regular controlled by an endocrinologist NDM: referred patients to a periodontist Follow up: 3 months	**DM type II (controlled/un‐controlled)** *N*: 61 ♀: 26 ◊ ♂: 35 ◊ Mean age: 59.3 ◊ Range:? DM duration:? Poorly controlled ♀: 16 ♂: 16 Mean age: 58.3 Range:? DM duration:? Good controlled ♀: 10 ♂: 19 Mean age: 60.5 Range:? DM duration:? **NDM** *N*: 31 ♀: 13 ♂: 18 Mean age: 57.4 Range:?	NSPT: SRP (mi, usi) Scaling and rootplaning in one or two sessions 1 h, oral hygiene instructions	PI, GI, PPD, CAL	DM patients might respond to non‐surgical periodontal therapy similarly well to non‐diabetic patients. There were no significant differences in the treatment outcomes among the groups according to most of the clinical parameters measured. In conclusion, based on the results yielded by the present study, the periodontal therapy outcome in patients with diabetes does not seem to be significantly affected by the level of glycemic control
XXX Gomathi et al. 2023 India Rob: critical	*N*: 30 DM: Dental College and Hospital NDM: Dental College and Hospital Follow up: 6 weeks	**DM type II** *N*: 15 ♀:? ♂:? Mean age:? Range:? DM duration:? NDM *N*: 15 ♀:? ♂:? Mean age:? Range:?	NSPT: SRP (la) Scaling and rootplaning in two sessions under local anaesthesia	PI, GI, PPD, CAL,	After NSPT, there was a reduction in clinical parameters in both groups

*Note:* ?: unknown/not reported.

Abbreviations: BOP, bleeding on probing; CAL, clinical attachment level; CT, ◊ = Calculated by the authors of this review based on the presented data in the selected paper; DM, diabetes mellitus; GBI, gingival bleeding index; GI, gingival index; la, local anaesthesia; mi, manual instrumentation; NDM, non diabetics; NSPT, non‐surgical periodontal treatment; PD, pocket depth; PI, plaque index; SRP, scaling rootplaning; usi, ultrasonic instrumentation.

### 3.2 Methodological Quality Assessment

A summary of the methodological quality and potential risk of bias scores is presented in Appendix S3. Based on the assessment domains, the estimated potential risk of bias was low for three studies [71, 73, 81], moderate for most of the studies [54, 56, 60, 62, 67, 68, 69, 70, 72, 74, 75, 76, 79], serious for 12 studies [58, 61, 63, 64, 65, 66, 77, 78, 80, 82, 83, 84] and critical for two studies [59, 85].

### 3.3 Assessment of Clinical and Methodological Heterogeneity

Considerable heterogeneity was observed among the 30 included studies. Characteristics of the study population, details regarding NSPT, outcome parameters, and other original conclusions are displayed in Table 2. All data extracted from the studies included in this SR were treated and interpreted as emerging from a controlled observational design, with follow‐up periods ranging from 1 to 12 months. Most of the studies measured periodontal outcomes at 3–4 months. Three studies reported outcomes at 12 months [58, 80, 81]. In total, 1425 participants were included in this SR, consisting of 792 DM patients. Studies originating from the following world continents are present: nine in Europe [54, 58, 59, 60, 63, 64, 70, 73, 82], 12 in Asia [61, 65, 66, 68, 71, 72, 74, 77, 78, 79, 80, 85], and nine in South America [56, 62, 67, 69, 75, 77, 81, 83, 84]. Out of the 30 included papers, 26 studies specifically focused on DM type II. Two studies focused on DM type I [58, 68] and two other studies did not specify the type of DM [59, 60]. Only one paper differentiated between types I and II diabetes [76]. All studies include an NDM group in satisfactory general health who were drawn from the population of the country where the study was performed. Regarding the level of periodontal disease, various criteria and diagnoses were used as parameters for inclusion. The majority of the studies excluded smokers, while five studies did include smokers [54, 60, 64, 69, 70], and four other papers did not provide information on the smoking habits of the participants [58, 59, 61, 74].

Some studies used a combination of ultrasonic and manual instrumentation as part of their NSPT while others relied solely on one or the other. Information on procedures such as the use of anaesthesia, duration of instrumentation, subsequent use of rubber cups for polishing, follow‐up supportive periodontal care, and instructions regarding oral hygiene products was sparsely reported in the included studies (Appendix S4). In 6 of the 10 studies that provided data ≥ 6 months, it was reported that supportive periodontal therapy was administered.

Regarding the diagnosis and severity of periodontitis, various criteria were used across studies. Most studies relied on clinical measures such as PPD, CAL, and radiographic assessments. Several studies distinguished between different levels of disease severity, particularly highlighting the presence of deep pockets. However, the exact classification of periodontitis severity varied. Some studies provided detailed information on the staging and grading of periodontitis, while others used broader definitions. In general, participants with moderate to severe periodontitis were included. Clinical assessments of DM were conducted using various methods, including fasting plasma glucose, glucose, and HbA1c levels. Some studies used professionally diagnosed DM, while others relied on self‐reports obtained through medical questionnaires or dental/medical records. The differentiation between controlled and poorly controlled DM was primarily based on self‐reported data or HbA1c levels.

### 3.4 Side Effects and Industry Funding

The included papers did not report any adverse events or side effects. Eighteen studies were not sponsored or did not provide any information regarding study funding. The other 12 studies reported being supported by a university or a non‐profit grant organisation.

### 3.5 Study Results

#### 3.5.1 Description of Findings

Appendix S5.1 describes and summarizes the statistical differences as reported in the original studies between DM patients and NDM individuals regarding the primary and secondary outcome parameters. In detail, the majority of the comparisons revealed no statistically significant differences in the results of NSPT between the two groups on any of the parameters of interest. With respect to the primary outcomes, 22 comparisons concerning PPD in DM type II, five found a statistical difference in favor of the NDM. For the secondary outcomes, a higher reduction in the gingivitis indices was obtained in favor of NDM in 1/3 comparisons on DM type I and 7/21 for DM type II. For plaque, no differences were found between NDM and DM type I, while 5/19 studies reported higher reductions in NDM compared to DM type II (see Appendix S5.2).

#### 3.5.2 Meta‐Analysis

Two studies [58, 59] provided data for the percentages of sites concerning PPD and CAL and could therefore not be included in the meta‐analysis. Study XV [68] did not provide overall data, so their data were only included in sub‐analysis for deep pockets (> 7 mm). Due to the lack of standard deviation or standard error of the mean for incremental scores in some studies, only 18 studies within 21 comparisons were included for the meta‐analysis of PPD and 15 studies included 18 comparisons for CAL. Sub‐analysis for the primary outcomes was performed for good and poorly controlled DM. It was not possible to differentiate between DM Type I and Type II based on the extracted data. Meta‐analysis for the secondary outcomes was performed on % plaque, PI, BOP, GI, GBI and REC. For details see Appendices S6.1–S6.5 and S7.1–S7.52.

##### 3.5.2.1 Overall Scores

###### 3.5.2.1.1 Primary Outcomes

There was a significant difference of means between DM and NDM for PPD at the baseline (DiffM = 0.21, *p* = 0.004, 95% CI [0.07<>0.35]) and end scores (DiffM = 0.16, *p* = 0.01, 95% CI [0.03<>0.29]) in favour of NDM. The same was found for CAL at baseline (DiffM = 0.27, *p* < 0.0001, 95% CI [0.13<>0.41]) and end data (DiffM = 0.26, *p* = 0.02, 95% CI [0.05<>0.47]) (Appendices S7.1–S7.2 and S7.4–S7.5). However, the mean PPD reduction following treatment in the DM group was not significantly different from the NDM group (DiffM = 0.02, *p* = 0.57, 95% CI [0.06<>0.10]) (Appendix S7.3). The mean CAL gain in the DM group was also not significantly different from NDM (DiffM = 0.06, *p* = 0.38, 95% CI [−0.19<>0.07]) (Appendix S7.6). Table 3 summarises the detailed outcomes of the meta‐analysis performed on the outcome parameters. Sensitivity analysis was conducted to evaluate the influence of a single study on the overall effect estimate by stepwise omitting, one by one, each of the studies included in the meta‐analysis and re‐evaluating the summary effect estimates. Sensitivity analysis did not reveal any specific effect on the observed outcomes.

**TABLE 3:** Meta‐analysis for the baseline, end and incremental data evaluating the effect of non‐surgical periodontal therapy on the clinical parameters of periodontitis. 3A: Probing pocket depth (PPD), 3B: Clinical attachment level (CAL), 3C: Bleeding scores (BS), 3D: Gingival indices, 3E: Plaque indices, 3F: Gingival recession (REC).

Measurement moment	Included studies # comparisons	Model	DiffM (mm)	Test overall		Test for heterogeneity		For details see Figure/Appendix
				95% CI	*p*	*I* ^2^ value (%)	*p*	
(A) PPD								
Baseline								
Overall	32	Random	0.21	[0.07<>0.35]	**0.004**	91%	< 0.00001	S7.1
PPD > 7 mm	4	Fixed	1.41	[−0.53<>3.35]	0.16	72%	0.01	S7.37
DM well controlled	14	Random	0.23	[0.05<>0.42]	0.01	76%	< 0.00001	S7.25
DM poorly controlled		Random	0.14	[−0.23<>0.51]	0.46	89%	< 0.00001	S7.28
Europe	12	Random	−0.01	[−0.22<>0.20]	0.93	94%	< 0.00001	S7.40
Asia	12	Random	0.46	[0.27<>0.64]	**< 0.00001**	53%	0.02	S7.43
South America	8	Random	0.28	[−0.11<>0.67]	0.16	90%	< 0.00001	S7.46
End								
Overall	32	Random	0.16	[0.03<>0.29]	**0.01**	94%	< 0.00001	S7.2
PPD > 7 mm	4	Fixed	1.21	[−0.25<>2.67]	0.10	88%	< 0.0001	S7.38
DM well controlled		Random	0.15	[0.03<>0.26]	**0.01**	49%	0.02	S7.26
DM poorly controlled	8	Random	0.15	[−0.01<>0.32]	0.07	51%	0.05	S7.29
Europe	12	Random	0.03	[−0.17<>0.23]	0.76	97%	< 0.00001	S7.41
Asia	12	Random	0.28	[0.11<>0.45]	**0.001**	66%	0.0007	S7.44
South America	8	Random	0.20	[0.04<>0.37]	**0.02**	53%	0.04	S7.47
Difference								
Overall	21	Random	0.02	[0.06<>0.10]	0.57	88%	< 0.00001	S7.3
PPD > 7 mm	3	Fixed	0.34	[−0.94<>1.62]	0.60	73%	0.03	S7.39
DM well controlled	7	Random	0.03	[−0.07<>0.14]	0.54	76%	0.00003	S7.27
DM poorly controlled	5	Random	0.07	[−0.06<>0.21]	0.29	75%	0.003	S7.30
Europe	8	Random	0.08	[0.01<>0.16]	**0.03**	74%	0.003	S7.42
Asia	6	Random	0.13	[−0.11<>0.37]	0.28	86%	< 0.00001	S7.45
South America	7	Random	−0.18	[−0.34<>−0.03]	**0.02**	77%	0.0002	S7.48

(B) Cal								
Baseline								
Overall	27	Random	0.27	[0.13<>0.41]	**0.0001**	83%	< 0.00001	S7.4
DM well controlled	12	Random	0.23	[0.08<>0.38]	**0.003**	54%	0.01	S7.31
DM poorly controlled	8	Random	0.14	[−0.09<>0.38]	0.24	40%	0.11	S7.34
Europe	12	Random	0.22	[−0.02<>0.45]	0.07	90%	< 0.00001	S7.49
Asia	7	Random	0.25	[0.05<>0.46]	**0.02**	71%	0.002	S7.52
South America	8	Random	0.41	[0.08<>0.74]	**0.01**	61%	0.01	S7.55
End								
Overall	27	Random	0.26	[0.05<>0.47]	**0.02**	96%	< 0.00001	S7.5
DM well controlled	12	Random	0.22	[0.07<>0.37]	**0.003**	58%	0.006	S7.32
DM poorly controlled	8	Random	0.20	[−0.01<>0.42]	**0.07**	18%	0.29	S7.35
Europe	12	Random	0.24	[−0.14<>0.62]	0.21	98%	< 0.00001	S7.50
Asia	7	Random	0.21	[−0.00<>0.42]	**0.05**	76%	0.0003	S7.53
South America	8	Random	0.32	[0.05<>0.60]	**0.02**	52%	0.04	S7.56
Difference								
Overall	18	Random	−0.06	[−0.19<>0.07]	0.38	94%	< 0.00001	S7.6
DM well controlled	6	Random	0.06	[−0.04<>0.16]	0.25	45%	0.10	S7.33
DM poorly controlled	5	Random	0.08	[0.02<>0.14]	**0.01**	17%	0.30	S7.36
Europe	8	Random	−0.02	[0.23<>0.19]	0.86	97%	< 0.00001	S7.51
Asia	3	Fixed	−0.02	[−0.13<>0.09]	0.72	82%	0.004	S7.54
South America	7	Random	−0.13	[−0.32<>0.05]	0.17	73%	0.001	S7.57

(C–I) BOP %										
	Baseline	Overall	14	Random	0.38	[−8.01<>8.77]	0.93	95%	< 0.00001	S7.10
	End	Overall	14	Random	4.64	[1.39<>7.89]	**0.005**	97%	< 0.00001	S7.11
	Difference	Overall	13	Random	4.39	[−3.91<>12.71]	0.30	100%	< 0.00001	S7.12

(C‐II) GBI—Ainamo & Bay (1975)										
	Baseline	Overall	9	Random	1.81	[−4.03<>7.64]	0.54	54%	0.03	S7.13
	End	Overall	9	Random	1.57	[−0.05<>3.18]	0.06	66%	0.003	S7.14
	Difference	Overall	4	Random	−17.94	[−40.62<>4.73]	0.12	95%	< 0.00001	S7.15

(D) Gingival index—Loë & Silness (1963)										
	Baseline	Overall	17	Random	0.13	[0.05<>0.22]	**0.003**	57%	0.002	S7.7
	End	Overall	17	Random	0.18	[0.10<>0.25]	**< 0.00001**	84%	< 0.00001	S7.8
	Difference	Overall	7	Random	0.01	[0.01<>0.13]	0.86	59%	0.02	S7.9

(E–I) Plaque scores %										
	Baseline	Overall	17	Random	3.69	[−3.88<>11.25]	0.34	95%	< 0.00001	S7.19
	End	Overall	17	Random	−0.48	[−7.65<>6.70]	0.90	96%	< 0.00001	S7.20
	Difference	Overall	14	Random	−2.80	[6.47<>0.87]	0.14	83%	< 0.00001	S7.21

(E‐II) PI – Silness en Loë (1964)										
	Baseline	Overall	15	Random	0.03	−0.09<>0.16	0.59	77%	< 0.00001	S7.22
	End	Overall	15	Random	0.07	−0.06<>0.19	0.29	92%	< 0.00001	S7.23
	Difference	Overall	4	Random	−0.28	−0.80<>0.23	0.28	94%	< 0.00001	S7.24

(E) Gingival recession (REC)										
	Baseline	Overall	4	Random	0.22	[−0.11<>0.56]	0.18	11%	0.34	S7.16
	End	Overall	4	Random	0.05	[−0.49<>0.60]	0.85	68%	0.02	S7.17
	Difference	Overall	3	Fixed	−0.10	[−0.27<>0.06]	0.23	0%	0.67	S7.18

Significance (*p*)	Not significant Meta‐analyses outcomes: *p* ≥ 0.05	**Significant** **Meta‐analyses outcomes: *p* < 0.05**

Test of heterogeneity (*I* ^2^)	Potential not important 0%–40%	Moderate 30%–60%	Substantial 50%–90%	Considerable 75%–100%

*Note:* Meta‐analyses outcomes resulting in a *p* < 0.05 was considered to be statistically significant [18]. As a guideline, to assess the potential magnitude of inconsistency between studies, an *I* ^2^ statistic of 0%–40% may represent unimportant levels of heterogeneity, 30%–60% may represent moderate heterogeneity, 50%–90% may represent substantial heterogeneity. An *I* ^2^ statistic of greater than 75% was interpreted to indicate considerable heterogeneity [18].

###### 3.5.2.1.2 Trial Sequential Analysis

**FIGURE 2:**
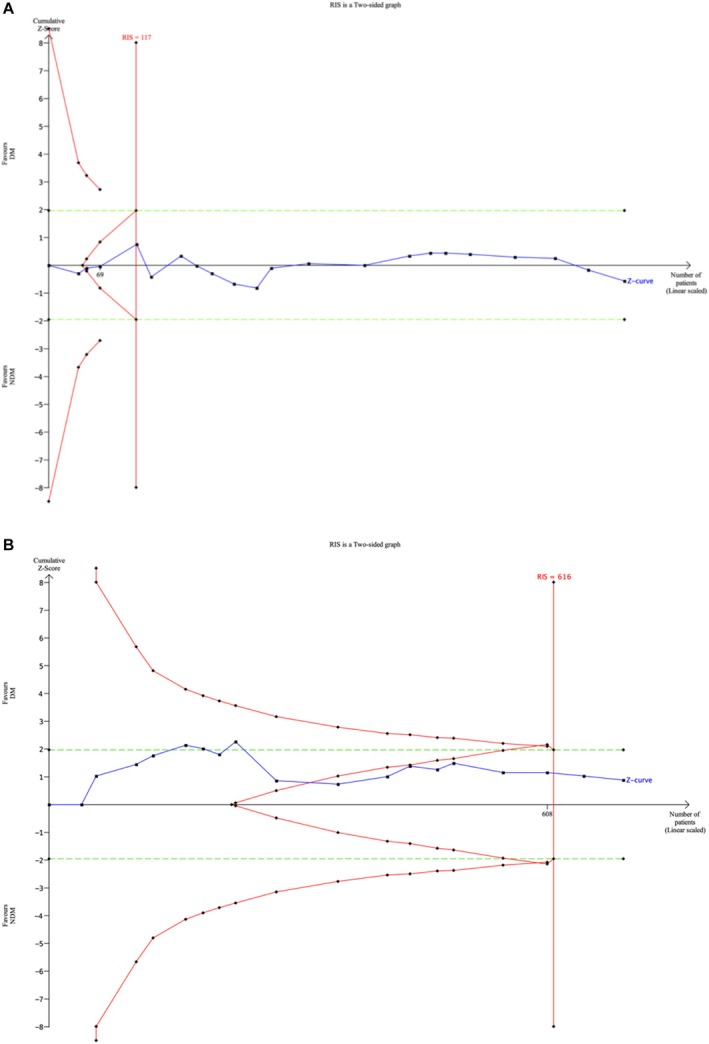
Trial Sequential analysis. The cumulative blue Z‐curves were constructed with each cumulative *Z*‐value calculated after including a new trial according to publication date. Crossing of the two‐sided *Z* = 1.96 indicates a traditionally significant result. Crossing of the red trial sequential monitoring boundaries is needed to obtain reliable evidence adjusted for random error risk. Z‐curves not crossing *Z*= 1.96 indicate absence of evidence if the information size is not reached or lack of the predefined intervention effect if the information size is not reached [81]. The green dotted lines represent the traditional boundary. The vertical red line represents the estimated heterogeneity‐adjusted required information size, the number of participants needed for the meta‐analysis sample size. (A) TSA of the incremental PPD scores. The user‐defined mean difference effect was set at 0.31, with a Type 1 error of 5.0% and a power of 80%. The estimated inconsistency was *I* ^2^ = 88%, and the estimated diversity was *D* ^2^ = 94%. (B) TSA of the incremental CAL scores. The user‐defined mean difference effect was set at 0.21 (34) with a Type 1 Error of 5.0%, and a power of 80%. The estimated inconsistency was *I* ^2^ = 94%, and the estimated diversity was *D* ^2^ = 97%.

Figure 2A illustrates that the cumulative Z‐curves crossed the futility boundary after including three studies for PPD, indicating a low likelihood of significant differences between the two treatment arms, making the addition of trials potentially ineffective. The futility was confirmed after including four studies, when the sample size surpassed the required meta‐analysis sample size, leading to the confident inference that there was no difference in effect between the groups. Regarding CAL, the Z curves crossed the futility boundary after the inclusion of 10 studies and the ineffectiveness of additional trials was confirmed after 17 studies (Figure 2B). It can be confidently inferred that there was no difference in effect between the groups, despite three Z‐values exceeding 1.96 in the preceding analysis. Based on the TSA of these meta‐analyses, the effect was found to be conclusive and reliable, indicating that additional data are unlikely to alter the summary effect [47, 86].

###### 3.5.2.1.3 Secondary Outcomes

At baseline, significant higher scores were found for GI in NDM (DiffM = 0.13, *p* = 0.003, 95% CI [0.05<>0.22]). There were also higher scores found post‐NSPT in NDM for GI (DiffM = 0.18, *p* < 0.0001, 95% CI [0.10<>0.25]) and BOP (DiffM = 4.64, *p* = 0.005, 95% CI [1.39<>7.89]). For the other secondary parameters, no significant differences were found. For details Table 3 and Appendices S7.7–S7.24.

##### 3.5.2.2 Sub Analysis

The sub‐analysis regarding the regulation of DM revealed a significant difference in favor of NDM compared to controlled DM for PPD before (DiffM = 0.23, *p* = 0.01, 95% CI 0.05<>0.42) and after treatment (DiffM = 0.15, *p* = 0.01, 95% CI 0.03<>0.26). Also, for CAL significant differences were in favor of NDM at baseline (DiffM = 0.23, *p* = 0.003, 95% CI 0.08<>0.38) and after treatment (DiffM = 0.22, *p* = 0.003, 95% CI 0.07<>0.37). For uncontrolled DM significant findings were found for CAL concerning the incremental data (DiffM = 0.08, *p* = 0.01, 95% CI 0.02<>0.14) and estimated to be a ‘zero’ effect [39] (Table 3A,B and Appendices S7.25–S7.36). Sub‐analysis for PPD ≥ 7 mm showed no significant difference in treatment response between the groups (Table 3A, Appendix S7.39). Based on a sub‐analysis of geographical regions in which the study was performed, significant findings were found for PPD reduction in Europe (DiffM = 0.08, *p* = 0.03, 95% CI 0.01<>0.16) in favor of NDM but estimated to be a ‘zero’ effect [36]. In contrast, significant findings were found for PPD in favor of DM in South America (DiffM = −0.18, *p* = 0.02, 95% CI −0.34<>0.03) but also estimated to be a ‘zero’ effect [36]. For CAL, the data revealed no overall statistical differences between the groups across different geographical regions (Table 3A,B, Appendices S7.40–S7.57).

#### 3.5.3 Statistical Heterogeneity

The funnel plot (Appendices S8.1–S8.20) shows that almost all outcomes are located at the top of the funnel, which is suggestive of publication bias. Egger's test shows a non‐significant *p*‐value for the primary outcomes (*p* = 0.2771, *p* = 0.4690). Statistically, heterogeneity was tested and was significant for all performed meta‐analyses, except for data concerning REC and the sub‐analysis for DM regulation on CAL (Table 3).

### 3.6 Evidence Profile

Table 4 presents a summary of the factors employed to establish the body of evidence profile according to the GRADE [53] framework relative to the magnitude of the risk.

**TABLE 4:** Summary of findings table of the quality and body of evidence and appraisal of the strength and the estimated evidence profile and of the outcome regarding NSPT.

Determinants of the quality	Primary outcomes		Secondary outcomes		
	PPD	CAL	Bleeding indices	Plaque indices	REC
Study design	Controlled observational studies				
included studies	30				
#comparisons	32	27	40	32	4
Risk of bias (Appendix S3)	Low to critical	Low to critical	Low to critical	Low to critical	Moderate to serious
Consistency (Table 3)	Rather consistent	Rather consistent	Rather consistent	Rather consistent	Rather consistent
Directness (Table 2)	Generalizable	Generalizable	Generalizable	Generalizable	Generalizable
Precision (Table 3, Appendix S4)	Rather precise	Rather precise	Rather precise	Rather precise	Rather precise
Reporting bias (Appendix S5)	Likely	Likely	Likely	Likely	Likely
Strength based on the quality and body of evidence	Moderate	Moderate	Moderate	Moderate	Moderate
Direction of recommendation	There is a moderate certainty that patients with DM undergoing NSPT can be treated as effectively as NDM with comparable outcomes for periodontal parameters				

The 30 observational studies examined (portrayed in Figure 1) demonstrated that the potential risk of bias was estimated to be ‘low’ to ‘critical’ (Appendix S3). Data from the included studies were derived from different populations and continents. Therefore, these findings are considered to be ‘generalizable’. Based on the heterogeneity between the included studies, data were judged to be ‘rather inconsistent’ (presented in Table 3). The data were considered to be ‘rather precise’ because all selected studies focused on NSPT as a primary outcome and because the majority revealed an overlap in the overall 95% CI (Table 3, Appendix S7). As publication bias may have been present, the presence of reporting bias is likely. Considering all GRADE aspects, the evidence profile that emerges from this review is that the strength of the evidence is ‘moderate’ [53]. Based on a synthesis of this evidence, there is moderate certainty that patients with DM and periodontitis have comparable treatment outcomes to NDM when undergoing NSPT.

## 4 Discussion

### 4.1 Summary of Findings

The present review summarised and synthesised the available literature for the response to treatment of DM periodontitis patients undergoing NSPT. The majority of the 30 included studies demonstrated significant improvements in periodontal parameters following NSPT for both DM and NDM (see Appendix S6). This is consistent with the findings of a recent systematic review, which reported that NSPT is effective for infection control in periodontitis patients [87].

The present analysis observed no clinically relevant difference in treatment response between the DM and NDM periodontitis patients, suggesting that periodontitis patients with diabetes can be treated comparably effectively as non‐diabetic patients undergoing NSPT. This finding warrants caution in overgeneralizing these results, considering the statistical heterogeneity, limitations, and potential biases in the data that emerged from the included studies. On the other hand, the TSA demonstrates that additional data are unlikely to alter the summary effect. As prediabetes may be reversible [88], data regarding this condition were excluded. Gestational diabetes consists of high blood glucose only during pregnancy [89] and was consequently not analysed in the present SR.

### 4.2 The Association Between DM and Periodontal Therapy

Previous ‘in vitro*’* and *‘*in vivo*’* studies, both in animals and humans, have established a relationship between DM and periodontitis [8, 9, 10, 21]. Supposedly, the host response to bacterial challenge does differ in patients with DM involving aspects of immune functioning, neutrophil activity, and cytokine biology [90]. Moreover, the accumulation of reactive oxygen species, oxidative stress, and interactions between advanced glycation end‐products (AGEs) in the periodontal tissues and their receptor contribute to increased inflammation in the periodontal tissues in people with DM [90]. It might therefore be presumed that DM patients exhibit a reduced response to therapy as compared to NDM individuals [91]. However, the nature of the bacterial challenge in patients with DM and periodontitis does not seem to differ from that of NDM [92, 93, 94, 95]. Also, a recent review showed that periodontal treatment was effective in reducing PPD and CAL in adults with type I/II DM [91]. This is consistent with the present comprehensive synthesis which indicates that periodontitis patients with DM receiving NSPT can achieve similar clinical results after they have undergone NSPT compared to NDM, challenging the more assertive claims made in previous studies that periodontitis is more difficult to manage in DM patients [96, 97]. Such as can be found in the *Treatment of stage I–III periodontitis—The EFP S3 level clinical practice guideline* (Sanz et al. 2020) [98] that DM is a proven risk factor in the etiopathogenesis of periodontitis and therefore, the control should be an integral component in the treatment of these patients.

### 4.3 Included Study Designs

Given the aim of our SR, where DM is regarded as an exposure rather than the primary focus of an intervention, an RCT design was not feasible for the research question. It is namely not possible to randomly assign periodontitis patients to DM and NDM groups. However, some included studies were initially designed as RCTs, where treatments were randomised within groups. From these studies, we extracted data specifically comparing DM and NDM periodontitis patients receiving NSPT while excluding data that were not relevant to our research question. Consequently, although some included studies were originally designed as RCTs or other interventional studies, we treated the extracted data as controlled observational data, as we focused primarily on periodontal outcomes between DM and NDM groups rather than assessing the effect of a specific intervention. For example, the study by Kaur et al. (2015) [71], originally designed as an RCT, was classified as a controlled observational study in our analysis because its primary focus was on periodontal treatment outcomes in DM versus NDM patients. This methodological decision ensures consistency in the interpretation of results across all included studies and aligns with the nature of our analysis.

### 4.4 Primary Outcomes

The baseline DiffM values were significantly different for CAL and PPD between DM and NDM (0.27 and 0.21 mm, respectively—see Table 3A,B). Considering these baseline differences, the potential for bias due to unmeasured variables, and differences in patient characteristics (including age and smoking habits) and study design variations should be acknowledged. In the present analysis, the DiffM was skewed towards the DM group. This could be attributed to confounding factors such as impaired glycemic control, altered inflammatory response, and compromised immune function in diabetic individuals, which may predispose them to a more severe form of periodontal disease [16]. Such differences in baseline scores can affect the validity and reliability of a study. If groups being compared start with different baselines, it becomes challenging to attribute observed differences at follow‐up to the intervention or treatment being tested rather than to pre‐existing differences. However, the end DiffM values were also significantly different for CAL and PPD between DM and NDM to more or less the same extent (0.26 and 0.16 mm, respectively—see Table 3A,B). Consequently, the incremental DiffM indicating the treatment effect was very small (0.06 and 0.02 mm, respectively—see Table 3A,B) and not significantly different. The extent of this difference in treatment effect with CAL can be interpreted as a ‘zero‐effect’ [39]. Smiley et al. (2015) [39] unfortunately do not provide guidelines for the interpretation of changes in PPD. However, as changes in PPD encompass both changes in CAL and the location of the gingival margin, it is reasonable to assume that changes in PPD should be at least as large as changes in CAL and presumably larger. Given that the DiffM of the incremental difference for PPD was 0.02 mm while it was 0.06 mm for CAL, one can safely interpret the difference in treatment effect between DM and NDM as also being a ‘zero‐effect’.

### 4.5 Secondary Outcomes

Analysis of the plaque indices (Table 3EI&II) shows that there was no difference between groups, indicating that the level of oral hygiene was comparable for DM and NDM. Similar to the PPD and CAL scores, the GI scores at baseline and end‐trial were also significantly different. However, the change in GI as a result of treatment was such that no significant difference was observed in the incremental difference between scores (see Table 3D). This also suggests that there is no difference in treatment response between DM and NDM, although one may argue whether the GI is an appropriate index concerning periodontitis. The GI is based on both visual signs of inflammation and bleeding on probing, with an emphasis on visual signs. It may therefore not capture the severity of periodontitis accurately, because, in the case of thick gingiva, the inflammatory infiltrate is not visible from the oral aspect [99, 100]. Studies have shown that for an objective diagnosis of the presence of an inflammatory lesion, the determination of BOP is a more reliable method [100]. The present analysis shows no significant DiffM in the incremental difference between baseline and end‐trial BOP scores (see Table 3CI&II). Additionally, the remaining meta‐analyses of REC did not reveal any statistical differences (*p* < 0.05), reinforcing that DM did not affect the response to periodontal treatment (see Table 3E).

### 4.6 Trial Sequential Analysis

TSA is valuable in SRs because it helps maintain the reliability and validity of conclusions drawn from meta‐analyses by controlling for random errors and adjusting for the risk of false positives and negatives. It is a cumulative random‐effects meta‐analysis method that estimates a “required information size” (i.e., required meta‐analysis sample size) using the same framework as sample size calculations for individual studies, while also accounting for heterogeneity and multiple comparisons when new studies are added. This ensures that the cumulative evidence is sufficiently robust before confirming or refuting the effectiveness, thus preventing premature conclusions and improving the overall quality of the evidence synthesis in meta‐analysis [47, 50]. Based on the TSA of these meta‐analyses, the effect was found to be conclusive and reliable, suggesting that the evidence of these meta‐analyses is firm, indicating that additional data are unlikely to alter the summary effect (for details see Figure 2A,B).

### 4.7 Sub‐Analysis for Uncontrolled and Controlled DM

The differentiation between controlled and poorly controlled DM in the primary studies was mainly done by self‐report (questionnaires) or clinical assessment using HbA1c levels. This distinction is important, as self‐reported data can be subject to recall bias and inaccuracies, potentially leading to misclassification of DM control status. In contrast, HbA1c levels provide a more objective and reliable measure of long‐term blood glucose control. When the regulation of DM was not differentiated, reported HbA1c levels were used to categorize the DM population for analysis. This could ensure a clearer distinction between the groups, providing a more accurate representation of each group.

Data from a USA National Health and Nutrition Examination Survey show a higher likelihood of periodontitis in patients with poorly controlled DM compared to NDM individuals [101]. Moreover, it has been shown that poorly controlled DM is associated with more severe periodontal disease [102]. Sub‐analysis regarding DM regulation on the primary outcomes found no significant DiffM in the incremental data for controlled DM compared to NDM. However, for uncontrolled DM patients, a significant DiffM was observed for the incremental scores regarding CAL (DiffM = 0.08, *p* = 0.01). Despite this, if the corresponding DiffM is interpreted according to Smiley et al. (2015), the effect is estimated as zero. Consequently, DM regulation appears to have no clinical effect on the response to NSPT (For details see Table 3A,B, Appendices S7.25–S7.36).

### 4.8 Type of DM

DM type I typically develops during childhood or adolescence, although it can also occur in adults. In contrast, type II often develops later in life and is more common in adults, accounting for approximately 90% of all DM cases [103]. Additionally, it is important to note that periodontitis is more prevalent in older people [104]. Therefore, only patients aged 18 and above were included in this study. Out of the 30 included papers, 26 studies specifically focused on DM type II, so the results may primarily reflect the effectiveness of NSPT in this population. Only three studies focused on DM type I [58, 68, 76], but two did not provide an SD or SE. Therefore, it was not possible to perform a subgroup analysis to compare types I and II DM data. In the descriptive analysis (see Appendix S5.1), none of the type I DM studies identified a significant difference compared to NDM for PPD. Furthermore, a recent evidence review has summarised that NSPT was effective in both type I and II [91]. However, this study compared NSPT to usual care and did not include a control group. While it is plausible that periodontal therapy is also effective in patients with DM type I, and this SR may have limitations in its generalizability, the broader evidence supports the effectiveness of NSPT across both types of DM.

### 4.9 Smoking

Smoking is a well‐established risk factor for both periodontitis and DM, increasing periodontal tissue loss and presumably complicating the treatment of DM [15, 16, 105]. Moreover, smoking impairs periodontal treatment outcomes [105]. The majority of the studies excluded smokers with only five also enrolled smokers [54, 60, 63, 69, 70]. These did however not provide separate treatment outcomes for smokers and non‐smokers. Therefore, it was not possible to evaluate the potential impact of smoking on NSPT outcomes.

### 4.10 Adjunctive Antibiotics

Adjunctive antibiotics have been used to improve clinical outcomes of NSPT [106]. It is reported that the adjunctive use of antibiotics may have limited benefit for periodontal treatment outcomes in patients with DM [107]. In the present review, the effect of antibiotics on treatment outcomes in DM subjects could not be determined because none of the included studies included the systemic use of antibiotics. In one included study [76], amoxicillin as antibiotic prophylaxis 1 h before the appointment was prescribed for DM patients.

### 4.11 Limitations

Factors such as differentiation between DM types I and II, smoking habits, and various examination protocols may have influenced the heterogeneity. These could not be further analysed due to a lack of complete descriptions of the population included in the original studies.

The heterogeneity observed among the included studies highlights the need for caution when generalising the results. Also, variations in DM control levels at follow‐up periods may have influenced treatment responses. Moreover, the language restriction to English resulted in one potential study that was excluded (see Appendix S1).

## 5 Conclusion

Although there was a significant difference of means for the primary outcomes between DM and NDM groups at baseline, a similar difference was observed in the end data. This indicates that the treatment effect between the two groups was not significantly different, as is evident from the DiffM of the incremental data for CAL and PPD. Therefore, the overall evidence from this SR indicates that there is no statistically significant difference in treatment outcomes between periodontitis patients with DM and NDM. Based on the evidence profile, it can be stated with moderate certainty that the difference in treatment outcomes following NSPT between periodontitis patients with DM and NDM is clinically insignificant. Future studies should aim to strengthen the evidence base by standardising clinical examination protocols, considering factors such as differentiating between type 1 and type 2 DM, accounting for smoking habits, and adopting the latest periodontitis classification.

## 6 Clinical Relevance

### 6.1 Scientific Rationale for the Study

Evidence supports an increased risk for periodontal diseases in diabetes (DM) patients. This may potentially influence the response to periodontal therapy. This systematic review aimed to evaluate the impact of DM on treatment outcomes following non‐surgical periodontal therapy (NSPT).

### 6.2 Principle Findings

In periodontitis patients, the treatment outcomes following NSPT did not show a significant difference between patients with or without DM.

### 6.3 Practical Implications

Periodontitis patients with DM can be treated as effectively as non‐DM periodontitis patients when undergoing NSPT.

## Author Contributions

L.P.M.W.: contributed to conception and design, search and selection, analysis and interpretation, and drafted the manuscript. T.M.J.A.T.: contributed to selection, analysis and interpretation and critically revised the manuscript. N.C.K.: contributed to selection, analysis and interpretation of the data. E.E.J.M.: contributed to analysis and critically revised the manuscript. C.V.: contributed to analysis and critically revised the manuscript. G.A.W.: contributed to conception and design, analysis and interpretation, and critically revised the manuscript. D.E.S.: contributed to conception and design, search and selection, analysis and interpretation, and critically revised the manuscript. All authors gave final approval and agreed to be accountable for all aspects of the work ensuring integrity and accuracy.

## Funding

This research did not receive any specific grant from funding agencies in the public, commercial, or not‐for‐profit sectors. This study was self‐funded by the authors and their respective institutions as work for this paper is funded by a regular academic appointment at the Academic Center for Dentistry Amsterdam (ACTA) of Slot, Thomassen, Van der Weijden and Weijdijk. This study is registered at the International Prospective Register of Systematic Reviews (PROSPERO) by number CRD42021227543 and approved by the ACTA Institutional Review Board, by reference number 2022‐43514.

## Conflicts of Interest

The authors declare no conflicts of interest.

## Supporting information


**Appendix S1:** idh70003‐sup‐0001‐supinfo.pdf.

## Data Availability

The data that support the findings of this study are available from the corresponding author upon reasonable request.

## References

[1] E. Könönen , M. Gursoy , and U. K. Gursoy , “Periodontitis: A Multifaceted Disease of Tooth‐Supporting Tissues,” Journal of Clinical Medicine 8, no. 8 (2019): 1135. 31370168 10.3390/jcm8081135 PMC6723779

[2] N. J. Kassebaum , A. G. C. Smith , E. Bernabé , et al., “Global, Regional, and National Prevalence, Incidence, and Disability Adjusted Life Years for Oral Conditions for 195 Countries, 1990–2015: A Systematic Analysis for the Global Burden of Diseases, Injuries, and Risk Factors,” Journal of Dental Research 96, no. 4 (2017): 380–387. 28792274 10.1177/0022034517693566 PMC5912207

[3] C. A. Ramseier , A. Anerud , M. Dulac , et al., “Natural History of Periodontitis: Disease Progression and Tooth Loss Over 40 Years,” Journal of Clinical Periodontology 44, no. 12 (2017): 1182–1191. 28733997 10.1111/jcpe.12782

[4] A. A. Abdulkareem , F. B. Al‐Taweel , A. J. B. Al‐Sharqi , S. S. Gul , A. Sha , and I. L. C. Chapple , “Current Concepts in the Pathogenesis of Periodontitis: From Symbiosis to Dysbiosis,” Journal of Oral Microbiology 15 (2023): 2197779. 37025387 10.1080/20002297.2023.2197779 PMC10071981

[5] W. J. Teeuw , V. E. Gerdes , and B. Loos , “Effect of Periodontal Treatment on Glycemic Control of Diabetic Patients: A Systematic Review and Meta‐Analysis,” Diabetes Care 33, no. 2 (2010): 421–427. 20103557 10.2337/dc09-1378 PMC2809296

[6] I. Chapple , B. Mealey , T. E. Van Dyke , et al., “Periodontal Health and Gingival Diseases and Conditions on an Intact and a Reduced Periodontium: Consensus Report of Workgroup 1 of the 2017 World Workshop on the Classification of Periodontal and Peri‐Implant Diseases and Conditions,” Journal of Periodontology 89 (2018): S74–S84. 29926944 10.1002/JPER.17-0719

[7] N. J. Kassebaum , E. Bernabé , M. Dahiya , B. Bhandari , C. J. L. Murray , and W. Marcenes , “Global Burden of Severe Periodontitis in 1990–2010: A Systematic Review and Meta‐Regression,” Journal of Dental Research 93, no. 11 (2014): 1045–1053. 25261053 10.1177/0022034514552491 PMC4293771

[8] I. L. Chapple and R. Genco , “Diabetes and Periodontal Diseases: Consensus Report of the Joint EFP/AAP Workshop on Periodontitis and Systemic Diseases,” Journal of Periodontology 84 (2013): S106–S112. 23631572 10.1902/jop.2013.1340011

[9] N. G. M. Chávarry , M. V. Vettore , C. Sansone , and A. Sheiham , “The Relationship Between Diabetes Mellitus and Destructive Periodontal Disease: A Meta‐Analysis,” Oral Health & Preventive Dentistry 7, no. 2 (2009): 107–127. 19583037

[10] Y. S. Khader , A. S. Dauod , S. S. El‐Qaderi , A. Alkafajei , and W. Q. Batayha , “Periodontal Status of Diabetics Compared With Nondiabetics: A Meta‐Analysis,” Journal of Diabetes and Its Complications 20, no. 1 (2006): 59–68. 16389170 10.1016/j.jdiacomp.2005.05.006

[11] L. Ziukaite , D. E. Slot , and F. A. van der Weijden , “Prevalence of Diabetes Mellitus in People Clinically Diagnosed With Periodontitis: A Systematic Review and Meta‐Analysis of Epidemiologic Studies,” Journal of Clinical Periodontology 6, no. 45 (2018): 650–662. 10.1111/jcpe.12839 29125699

[12] I. Păunică , M. Giurgiu , A. S. Dumitriu , et al., “The Bidirectional Relationship Between Periodontal Disease and Diabetes Mellitus‐A Review,” Diagnostics (Basel) 13, no. 4 (2023): 681. 36832168 10.3390/diagnostics13040681 PMC9954907

[13] M. Zheng , C. Wang , A. Ali , Y. A. Shih , Q. Xie , and C. Guo , “Prevalence of Periodontitis in People Clinically Diagnosed With Diabetes Mellitus: A Meta‐Analysis of Epidemiologic Studies,” Acta Diabetologica 58, no. 10 (2021): 1307–1327. 34028620 10.1007/s00592-021-01738-2

[14] A. Mirzaei , E. Shahrestanaki , E. Daneshzad , et al., “Association of Hyperglycaemia and Periodontitis: An Updated Systematic Review and Meta‐Analysis,” Journal of Diabetes & Metabolic Disorders 20, no. 2 (2021): 1327–1336. 34900784 10.1007/s40200-021-00861-9 PMC8630338

[15] P. M. Preshaw , A. L. Alba , D. Herrera , et al., “Periodontitis and Diabetes: A Two‐Way Relationship,” Diabetologia 55, no. 1 (2012): 21–31. 22057194 10.1007/s00125-011-2342-y PMC3228943

[16] E. Lalla and P. N. Papapanou , “Diabetes Mellitus and Periodontitis: A Tale of Two Common Interrelated Diseases,” Nature Reviews. Endocrinology 7, no. 12 (2011): 738–748. 10.1038/nrendo.2011.106 21709707

[17] X. Zhou , W. Zhang , X. Liu , W. Zhang , and Y. Li , “Interrelationship Between Diabetes and Periodontitis: Role of Hyperlipidemia,” Archives of Oral Biology 60, no. 4 (2015): 667–674. 25443979 10.1016/j.archoralbio.2014.11.008

[18] M. Kumar , L. Mishra , R. Mohanty , and R. Nayak , “Diabetes and Gum Disease: The Diabolic Duo,” Diabetes & Metabolic Syndrome: Clinical Research & Reviews 8, no. 4 (2014): 255–258. 10.1016/j.dsx.2014.09.022 25450824

[19] L. Guariguata , D. R. Whiting , I. Hambleton , J. Beagley , U. Linnenkamp , and J. E. Shaw , “Global Estimates of Diabetes Prevalence for 2013 and Projections for 2035,” Diabetes Research and Clinical Practice 103, no. 2 (2014): 137–149. 24630390 10.1016/j.diabres.2013.11.002

[20] G. L. Di Domenico , M. Minoli , N. Discepoli , A. Ambrosi , and M. de Sanctis , “Effectiveness of Periodontal Treatment to Improve Glycemic Control: An Umbrella Review,” Acta Diabetologica 60, no. 1 (2023): 101–113. 36261746 10.1007/s00592-022-01991-z

[21] Y. T. Hsu , M. Nair , N. Angelov , E. Lalla , and C. T. Lee , “Impact of Diabetes on Clinical Periodontal Outcomes Following Non‐Surgical Periodontal Therapy,” Journal of Clinical Periodontology 46, no. 2 (2019): 206–217. 30536853 10.1111/jcpe.13044

[22] N. P. Lang , A. Joss , T. Orsanic , F. A. Gusberti , and B. E. Siegrist , “A Predictor for the Progression of Periodontal Disease?,” Journal of Clinical Periodontology 13, no. 6 (1986): 590–600. 3489010 10.1111/j.1600-051x.1986.tb00852.x

[23] N. P. Lang and P. M. Bartold , “Periodontal Health,” Journal of Periodontology 89, no. 1 (2018): S9–S16. 29926938 10.1002/JPER.16-0517

[24] J. P. T. Higgins , J. Thomas , J. Chandler , et al., “Cochrane Handbook for Systematic Reviews of Interventions Version 6.4 (Updated August 2023),” in Cochrane Handbook for Systematic Reviews of Interventions: Cochrane Book Series, 2nd ed. (John Wiley & Sons, 2019).

[25] D. F. Stroup , J. A. Berlin , S. C. Morton , et al., “Meta‐Analysis of Observational Studies in Epidemiology: A Proposal for Reporting. Meta‐Analysis of Observational Studies in Epidemiology (MOOSE) Group,” Journal of the American Medical Association 283, no. 15 (2000): 2008–2012. 10789670 10.1001/jama.283.15.2008

[26] L. Shamseer , D. Moher , M. Clarke , et al., “Preferred Reporting Items for Systematic Review and Meta‐Analysis Protocols (PRISMA‐P) 2015: Elaboration and Explanation,” BMJ (Clinical Research Ed.) 2, no. 349 (2015): g7647. 10.1136/bmj.g7647 25555855

[27] R. L. Morgan , P. Whaley , K. A. Thayer , and H. J. Schünemann , “Identifying the PECO: A Framework for Formulating Good Questions to Explore the Association of Environmental and Other Exposures With Health Outcomes,” Environment International 121, no. Pt1 (2018): 1027–1031. 30166065 10.1016/j.envint.2018.07.015 PMC6908441

[28] M. Ouzzani , H. Hammady , Z. Fedorowicz , and A. Elmagarmid , “Rayyan‐a Web and Mobile App for Systematic Reviews,” Systematic Reviews 5 (2016): 210. 27919275 10.1186/s13643-016-0384-4 PMC5139140

[29] S. Moola , Z. Munn , C. Tufanaru , et al., “Chapter 7: Critical Appraisal Checklist for Analytical Cross Sectional Studies,” in Joanna Briggs Institute Reviewer's Manual, ed. E. Aromataris and Z. Munn (Joanna Briggs Institute, 2017), https://reviewersmanual.joannabriggs.org/.

[30] G. A. Wells , B. Shea , D. O'Connell , et al., “The Newcastle‐Ottawa Scale (NOS) for Assessing the Quality if Nonrandomized Studies in Meta‐Analyses,” (2012), https://www.ohri.ca/programs/clinical_epidemiology/oxford.asp.

[31] J. P. T. Higgins , R. L. Morgan , A. A. Rooney , et al., “A Tool to Assess Risk of Bias in Non‐Randomized Follow‐Up Studies of Exposure Effects (ROBINS‐E),” Environment International 186 (2024): 108602. 38555664 10.1016/j.envint.2024.108602 PMC11098530

[32] F. N. van der Weijden , R. B. Kuitert , F. R. U. Berkhout , and G. A. van der Weijden , “Influence of Tooth Position on Wind Instrumentalists' Performance and Embouchure Comfort: A Systematic Review,” Journal of Orofacial Orthopedics 79 (2018): 205–218. 29532091 10.1007/s00056-018-0128-2 PMC5954010

[33] M. A. Listgarten , “Periodontal Probing: What Does It Mean?,” Journal of Clinical Periodontology 7, no. 3 (1980): 165–176. 7000852 10.1111/j.1600-051x.1980.tb01960.x

[34] J. Highfield , “Diagnosis and Classification of Periodontal Disease,” Australian Dental Journal 54, no. 1 (2009): 11–26. 10.1111/j.1834-7819.2009.01140.x 19737262

[35] T. J. O'Leary , R. B. Drake , and J. E. Naylor , “The Plaque Control Record,” Journal of Periodontology 43, no. 1 (1972): 38. 4500182 10.1902/jop.1972.43.1.38

[36] J. Silness and H. Loe , “Periodontal Disease in Pregnancy. II. Correlation Between Oral Hygiene and Periodontal Condition,” Acta Odontologica Scandinavica 22 (1964): 121–135. 14158464 10.3109/00016356408993968

[37] H. M. Goldman and D. W. Cohen , Periodontal Therapy (St Louis, 1968), 80–82.

[38] M. G. Newman , H. Takei , P. R. Klokkeveld , and F. A. Carranza , Eds, Carranza's Clinical Periodontology (Elsevier, 2014).

[39] C. J. Smiley , S. L. Tracy , E. Abt , et al., “Evidence‐Based Clinical Practice Guideline on the Nonsurgical Treatment of Chronic Periodontitis by Means of Scaling and Root Planing With or Without Adjuncts,” Journal of the American Dental Association 146, no. 7 (2015): 525–535. 26113100 10.1016/j.adaj.2015.01.026

[40] The Nordic Cochrane Centre, The Cochrane Collaboration . “Review Manager (RevMan) [Internet]. Version 6.5. London: The Cochrane Collaboration,” (2024), [cited 2025 Oct 28], https://revman.cochrane.org.

[41] M. Egger , G. D. Smith , M. Schneider , and C. Minder , “Bias in Meta‐Analysis Detected by a Simple, Graphical Test,” British Medical Journal 315, no. 7109 (1997): 629–634. 9310563 10.1136/bmj.315.7109.629 PMC2127453

[42] D. F. Stroup , J. A. Berlin , S. C. Morton , et al., “Meta‐Analysis of Observational Studies in Epidemiology: A Proposal for Reporting,” Journal of the American Medical Association 283, no. 15 (2000): 2008–2012. 10789670 10.1001/jama.283.15.2008

[43] D. Moher , A. Liberati , J. Tetzlaff , D. G. Altman , and PRISMA GROUP , “Preferred Reporting Items for Systematic Reviews and Meta‐Analyses: The PRISMA Statement,” PLoS Medicine 6, no. 7 (2009): e1000097. 19621072 10.1371/journal.pmed.1000097 PMC2707599

[44] D. L. DeMets and K. K. Lan , “Interim Analysis: The Alpha Spending Function Approach,” Statistics in Medicine 13 (1994): 1341–1352. 7973215 10.1002/sim.4780131308

[45] P. C. O'Brien and T. R. Fleming , “A Multiple Testing Procedure for Clinical Trials,” Biometrics 35 (1979): 549. 497341

[46] J. Wetterslev , K. Thorlund , J. Brok , and C. Gluud , “Trial Sequential Analysis May Establish When Firm Evidence Is Reached in Cumulative Meta‐Analysis,” Journal of Clinical Epidemiology 61 (2008): 64–75. 18083463 10.1016/j.jclinepi.2007.03.013

[47] K. Thorlund , J. Engstrøm , J. Wetterslev , G. Imberger , and C. Gluud , “User Manual for Trial Sequential Analysis (TSA),” Copenhagen Trial Unit, 2011, Copenhagen, Denmark: Centre for Clinical Intervention Research (2017), 1–119.

[48] J. Brok , K. Thorlund , J. Wetterslev , and C. Gluud , “Apparently Conclusive Meta‐Analyses May Be Inconclusive–Trial Sequential Analysis Adjustment of Random Error Risk due to Repetitive Testing of Accumulating Data in Apparently Conclusive Neonatal Meta‐Analyses,” International Journal of Epidemiology 38 (2009): 287–298. 18824466 10.1093/ije/dyn188

[49] K. Thorlund , A. Anema , and E. Mills , “Interpreting Meta‐Analysis According to the Adequacy of Sample Size. An Example Using Isoniazid Chemoprophylaxis for Tuberculosis in Purified Protein Derivative Negative HIV‐Infected Individuals,” Clinical Epidemiology 2 (2010): 57–66. 20865104 10.2147/clep.s9242 PMC2943189

[50] P. S. Roshanov , B. B. Dennis , N. Pasic , A. X. Garg , and M. Walsh , “When Is a Meta‐Analysis Conclusive? A Guide to Trial Sequential Analysis With an Example of Remote Ischemic Preconditioning for Renoprotection in Patients Undergoing Cardiac Surgery,” Nephrology, Dialysis, Transplantation 32 (2017): ii23–ii30. 10.1093/ndt/gfw219 28380638

[51] R. Ryan and Cochrane Consumers and Communication Review Group , “Heterogeneity and Subgroup Analyses in Cochrane Consumers and Communication Group Reviews: Planning the Analysis at Protocol Stage,” (2016), https://cccrg.cochrane.org/.

[52] N. Meader , K. King , A. Llewellyn , et al., “A Checklist Designed to Aid Consistency and Reproducibility of GRADE Assessments: Development and Pilot Validation,” Systematic Reviews 24, no. 3 (2014): 82. 10.1186/2046-4053-3-82 PMC4124503 25056145

[53] G. Guyatt , A. D. Oxman , S. Sultan , et al., “GRADE Guidelines: 11. Making an Overall Rating of Confidence in Effect Estimates for a Single Outcome and for All Outcomes,” Journal of Clinical Epidemiology 66, no. 2 (2013): 151–157. 22542023 10.1016/j.jclinepi.2012.01.006

[54] A. B. Navarro‐Sanchez , R. Faria‐Almeida , and A. Bascones‐Martinez , “Effect of Non‐Surgical Periodontal Therapy on Clinical and Immunological Response and Glycaemic Control in Type 2 Diabetic Patients With Moderate Periodontitis,” Journal of Clinical Periodontology 34, no. 10 (2007): 835–843. 17850602 10.1111/j.1600-051X.2007.01127.x

[55] R. Faria‐Almeida , A. Navarro , and A. Bascones , “Clinical and Metabolic Changes After Conventional Treatment of Type 2 Diabetic Patients With Chronic Periodontitis,” Journal of Periodontology 77, no. 4 (2006): 591–598. 16584339 10.1902/jop.2006.050084

[56] D. Gonçalves , “The Effect of Non‐Surgical Periodontal Therapy on Peroxidase Activity in Diabetic Patients: A Case–Control Pilot Study,” Journal of Clinical Periodontology 35 (2008): 799–806. 18651848 10.1111/j.1600-051X.2008.01289.x

[57] C. M. Silva‐Boghossian , S. R. P. Orrico , D. Gonçalves , F. O. B. Correa , and A. P. V. Colombo , “Microbiological Changes After Periodontal Therapy in Diabetic Patients With Inadequate Metabolic Control,” Brazilian Oral Research 28, no. 1 (2014): 1–9. 10.1590/1807-3107bor-2014.vol28.0007 24918369

[58] T. Tervonen and K. Karjalainen , “Periodontal Disease Related to Diabetic Status,” Journal of Clinical Periodontology 24, no. 7 (1997): 505–510. 9226392 10.1111/j.1600-051x.1997.tb00219.x

[59] T. Tervonen , M. Knuuttila , L. Pohjamo , and H. Nurkkala , “Immediate Response to Non‐Surgical Periodontal Treatment in Subjects With Diabetes Mellitus,” Journal of Clinical Periodontology 18, no. 1 (1991): 65–68. 2045520 10.1111/j.1600-051x.1991.tb01121.x

[60] M. Christgau , K.‐D. Palitzsch , G. Sclimah , U. Kreiner , and S. Frenzel , “Periodontal Therapy in Diabetics,” Journal of Clinical Periodontclogy 25 (1998): 112–124. 10.1111/j.1600-051x.1998.tb02417.x 9495610

[61] K. Sonoki , S. Nakashima , Y. Takata , et al., “Decreased Lipid Peroxidation Following Periodontal Therapy in Type 2 Diabetic Patients,” Journal of Periodontology 77, no. 11 (2006): 1907–1913. 17076618 10.1902/jop.2006.060088

[62] F. O. B. Correa , D. Gonçalves , C. M. S. Figueredo , A. Gustafsson , and S. R. P. Orrico , “The Short‐Term Effectiveness of Non‐Surgical Treatment in Reducing Levels of Interleukin‐1β and Proteases in Gingival Crevicular Fluid From Patients With Type 2 Diabetes Mellitus and Chronic Periodontitis,” Journal of Periodontology 79, no. 11 (2008): 2143–2150. 10.1902/jop.2008.080132 29539236

[63] A. Daǧ , E. T. Firat , Ş. Arikan , A. K. Kadiroǧlu , and A. Kaplan , “The Effect of Periodontal Therapy on Serum TNF‐α and HbA1c Levels in Type 2 Diabetic Patients,” Australian Dental Journal 54, no. 1 (2009): 17–22. 19228128 10.1111/j.1834-7819.2008.01083.x

[64] L. Kardeşler , N. Buduneli , Ş. Çetinkalp , and D. F. Kinane , “Adipokines and Inflammatory Mediators After Initial Periodontal Treatment in Patients With Type 2 Diabetes and Chronic Periodontitis,” Journal of Periodontology 81, no. 1 (2010): 24–33. 20059414 10.1902/jop.2009.090267

[65] P. Kudva , S. Tabasum , and N. Garg , “Evaluation of Clinical and Metabolic Changes After Non Surgical Periodontal Treatment of Type 2 Diabetes Mellitus Patients: A Clinico Biochemical Study,” Journal of Indian Society of Periodontology 14, no. 4 (2010): 257–262. 21731253 10.4103/0972-124X.76933 PMC3118078

[66] S. Hungund and B. J. Panseriya , “Reduction in HbA1c Levels Following Non‐Surgical Periodontal Therapy in Type‐2 Diabetic Patients With Chronic Generalized Periodontitis: A Periodontist's Role,” Journal of Indian Society of Periodontology 16, no. 1 (2012): 16–21. 22628957 10.4103/0972-124X.94598 PMC3357026

[67] F. R. Cirano , C. Pêra , P. Ueda , et al., “Clinical and Metabolic Evaluation of One‐Stage, Full‐Mouth, Ultrasonic Debridement as a Therapeutic Approach for Uncontrolled Type 2 Diabetic Patients With Periodontitis,” Quintessence International (Berlin) 43, no. 8 (2012): 671–681. 23034420

[68] S. M. Buzinin , A. M. Alabsi , A. T. B. Tan , V. K. Vincent‐Chong , and D. Swaminathan , “Effects of Nonsurgical Periodontal Therapy on Clinical Response, Microbiological Profile, and Glycemic Control in Malaysian Subjects With Type 1 Diabetes,” Scientific World Journal 2014 (2014): 232535. 25147841 10.1155/2014/232535 PMC4132400

[69] N. J. López , A. Quintero , P. A. Casanova , and B. Martínez , “Routine Prophylaxes Every 3 Months Improves Chronic Periodontitis Status in Type 2 Diabetes,” Journal of Periodontology 85, no. 7 (2014): e232–e240. 24354651 10.1902/jop.2013.130400

[70] G. Kara , E. Cifcibasi , K. Karsidag , and S. Cintan , “Short Term Effects of Periodontal Therapy on Inflammatory Markers in Patients With Type‐2 Diabetes,” Saudi Medical Journal 36, no. 4 (2015): 469–476. 25828285 10.15537/smj.2015.4.10380 PMC4404482

[71] P. K. Kaur , S. C. Narula , R. Rajput , R. K. Sharma , and S. Tewari , “Periodontal and Glycemic Effects of Nonsurgical Periodontal Therapy in Patients With Type 2 Diabetes Stratified by Baseline HbA1c,” Journal of Oral Science 57, no. 3 (2015): 201–211. 26369484 10.2334/josnusd.57.201

[72] J. J. Pannicker and D. S. Mehta , “Effects of Scaling and Root Planing on Gingival Crevicular Fluid Vascular Endothelial Growth Factor Level in Chronic Periodontitis Patients With and Without Diabetes Mellitus: A Clinicobiochemical Study,” Journal of Indian Society of Periodontology 20, no. 3 (2016): 244–248. 27563195 10.4103/0972-124X.176395 PMC4976542

[73] Ş. B. Doğan , U. Ballı , F. Ö. Dede , E. Sertoğlu , and K. Tazegül , “Chemerin as a Novel Crevicular Fluid Marker of Patients With Periodontitis and Type 2 Diabetes Mellitus,” Journal of Periodontology 87, no. 8 (2016): 923–933. 26991487 10.1902/jop.2016.150657

[74] V. Mishra , L. Shettar , M. Bajaj , A. S. Math , and S. L. Thakur , “Interlinking Periodontitis and Type 2 Diabetes Mellitus by Assessment of Crevicular Visfatin Levels in Health and in Disease Before and After Initial Periodontal Therapy,” Journal of Clinical and Diagnostic Research 10, no. 8 (2016): ZC67–ZC71. 10.7860/JCDR/2016/18656.8283 PMC5028447 27656567

[75] I. S. Abreu , V. T. Euzebio Alves , A. P. S. Benedete , et al., “Gingival Crevicular Fluid Levels of Protease‐Activated Receptors Type 1 and Type 2 in Diabetic Patients With Periodontitis,” Journal of Periodontal Research 51, no. 5 (2016): 577–585. 26564991 10.1111/jre.12336

[76] C. C. Peruzzo Lopes , P. do Monte Ribeiro Busato , M. F. Michelin Mânica , et al., “Effect of Basic Periodontal Treatment on Glycemic Control and Inflammation in Patients With Diabetes Mellitus Type 1 and Type 2: Controlled Clinical Trial,” Journal of Public Health (Germany) 25, no. 4 (2017): 443–449.

[77] G. Sundaram , T. Ramakrishnan , H. Parthasarathy , J. Moses , and T. Lalitha , “Evaluation of Micronutrient (Zinc, Magnesium, and Copper) Levels in Serum and Glycemic Status After Nonsurgical Periodontal Therapy in Type 2 Diabetic Patients With Chronic Periodontitis,” Contemporary Clinical Dentistry 8, no. 1 (2017): 26–32. 28566847 10.4103/0976-237X.205036 PMC5426161

[78] S. Gayathri , E. Koshi , A. Sadasivan , P. R. Arunima , and K. Jaya Kumar , “Effect of Initial Periodontal Therapy on Serum Nitric Oxide Levels in Chronic Periodontitis Patients With or Without Type 2 Diabetes Mellitus,” Journal of Contemporary Dental Practice 20, no. 2 (2019): 197–203. 31058635

[79] C. R. Ahuja , A. P. Kolte , R. A. Kolte , M. Gupta , and S. Chari , “Effect of Non‐Surgical Periodontal Treatment on Gingival Crevicular Fluid and Serum Leptin Levels in Periodontally Healthy Chronic Periodontitis and Chronic Periodontitis Patients With Type 2 Diabetes Mellitus,” Journal of Investigative and Clinical Dentistry 10, no. 3 (2019): e12420. 31172690 10.1111/jicd.12420

[80] L. Pragada , D. S. Mehta , V. Manasa , C. G. Bathini , S. Kesari , and R. Bansal , “Effect of Scaling and Root Planing on Gingival Crevicular Fluid Levels of Adrenomedullin in Chronic Periodontitis Patients With and Without Diabetes Mellitus Type 2: A Clinico‐Biochemical Study,” Annals of African Medicine 18, no. 2 (2019): 92–96. 31070151 10.4103/aam.aam_40_18 PMC6521633

[81] M. L. Almeida , P. M. Duarte , E. A. Figueira , et al., “Effects of a Full‐Mouth Disinfection Protocol on the Treatment of Type‐2 Diabetic and Non‐Diabetic Subjects With Mild‐To‐Moderate Periodontitis: One‐Year Clinical Outcomes,” Clinical Oral Investigations 24, no. 1 (2020): 333–341. 31102044 10.1007/s00784-019-02927-8

[82] J. Mirnić , M. Đurić , N. Nikolić , et al., “Clinical and Microbiological Assessment of Non‐Surgical Treatment of Chronic Periodontitis in Controlled and Uncontrolled Type 2 Diabetic Patients,” Acta Clinica Croatica 60, no. 3 (2021): 406–414. 10.20471/acc.2021.60.03.10 PMC8907956 35282487

[83] G. A. da Cruz , S. de Toledo , E. A. Sallum , et al., “Clinical and Laboratory Evaluations of Non‐Surgical Periodontal Treatment in Subjects With Diabetes Mellitus,” Journal of Periodontology 79, no. 7 (2008): 1150–1157. 18597596 10.1902/jop.2008.070503

[84] G. A. Da Cruz Galhardo Camargo , M. De Andrade Lima , T. Vieira Fortes , C. Salgado De Souza , A. Maria De Jesus , and R. Pacheco De Almeida , “Effect of Periodontal Therapy on Metabolic Control and Levels of IL‐6 in the Gingival Crevicular Fluid in Type 2 Diabetes Mellitus,” Indian Journal of Dental Research 24, no. 1 (2013): 110–116, https://www.ijdr.in/article.asp?issn=0970‐9290;year=2013;volume=24;issue=1;spage=110;epage=116;aulast=Galhardo. 23852243 10.4103/0970-9290.114953

[85] G. D. Gomathi , S. Gopalakrishnan , U. Sudhakar , A. Raghavan , and K. V. Narayan , “Effects of Non‐Surgical Periodontal Therapy on Saliva and Gingival Crevicular Fluid Levels of Chemerin in Periodontitis Subjects With and Without Type 2 Diabetes Mellitus,” Cureus 15, no. 1 (2023): e33388. 36751175 10.7759/cureus.33388 PMC9899038

[86] G. Imberger , K. Thorlund , C. Gluud , and J. Wetterslev , “False‐Positive Findings in Cochrane Meta‐Analyses With and Without Application of Trial Sequential Analysis: An Empirical Review,” BMJ Open 6, no. 8 (2016): e011890, 10.1136/bmjopen-2016-011890. PMC4985805 27519923

[87] J. Suvan , Y. Leira , F. M. Moreno Sancho , F. Graziani , J. Derks , and C. Tomasi , “Subgingival Instrumentation for Treatment of Periodontitis. A Systematic Review,” Journal of Clinical Periodontology 47, no. S22 (2020): 155–175. 31889320 10.1111/jcpe.13245

[88] P. Tuso , “Prediabetes and Lifestyle Modification: Time to Prevent a Preventable Disease,” Permanente Journal 18, no. 3 (2014): 88–93. 25102521 10.7812/TPP/14-002 PMC4116271

[89] National Center for Health Statistics (NCHS) , National Health and Nutrition Examination Survey Data (U.S. Department of Health and Human Services, Centers for Disease Control and Prevention, 2022) Centers for Disease Control and Prevention (CDC), https://www.cdc.gov/nchs/nhanes/index.htm.

[90] J. J. Taylor , P. M. Preshaw , and E. Lalla , “A Review of the Evidence for Pathogenic Mechanisms That May Link Periodontitis and Diabetes,” Journal of Clinical Periodontology 40, no. Suppl. 14 (2013): S113–S134. 23627323 10.1111/jcpe.12059

[91] Evidence Review D for Periodontal Treatment to Improve Diabetic Control in Adults With Type 1 or Type 2 Diabetes: Periodontal Treatment to Improve Diabetic Control in Adults With Type 1 or Type 2 Diabetes: Evidence Review D (National Institute for Health and Care Excellence (NICE), 2022). 36881683

[92] A. B. Novaes, Jr. , F. Gonzalez Gutierrez , M. F. Grisi , and A. B. Novaes , “Periodontal Disease Progression in Type II Non‐Insulin‐Dependent Diabetes Mellitus Patients (NIDDM). Part II—Microbiological Analysis Using the BANA Test,” Brazilian Dental Journal 8 (1997): 27–33. 9485634

[93] H. Thorstensson , G. Dahlén , and A. Hugoson , “Some Suspected Periodontopathogens and Serum Antibody Response in Adult Long‐Duration Insulin‐Dependent Diabetics,” Journal of Clinical Periodontology 22 (1995): 449–458. 7560223 10.1111/j.1600-051x.1995.tb00176.x

[94] A. C. Feitosa , M. de Uzeda , and A. B. Novaes, Jr. , “ *Actinobacillus actinomycetemcomitans* in Brazilian Insulin‐Dependent Individuals With Diabetes Mellitus,” Brazilian Dental Journal 3 (1992): 25–31. 1303114

[95] J. J. Zambon , H. Reynolds , J. G. Fisher , M. Shlossman , R. Dunford , and R. J. Genco , “Microbiological and Immunological Studies of Adult Periodontitis in Patients With Noninsulin‐Dependent Diabetes Mellitus,” Journal of Periodontology 59, no. 1 (1988): 23–31. 3276868 10.1902/jop.1988.59.1.23

[96] R. Kolte , A. Kolte , P. Bawankar , and V. Bajaj , “Effect of Nonsurgical Periodontal Therapy on Metabolic Control and Systemic Inflammatory Markers in Patients of Type 2 Diabetes Mellitus With Stage III Periodontitis,” Contemporary Clinical Dentistry 14, no. 1 (2023): 45–51. 37249991 10.4103/ccd.ccd_514_21 PMC10209773

[97] L. C. G. Belizário , C. M. S. Figueredo , J. V. S. Rodrigues , et al., “The Impact of Type 2 Diabetes Mellitus on Non‐Surgical Periodontal Treatment: A Non‐Randomized Clinical Trial,” Journal of Clinical Medicine 13, no. 19 (2024): 5978. 39408037 10.3390/jcm13195978 PMC11477662

[98] M. Sanz , D. Herrera , M. Kebschull , et al., “Treatment of Stage I–III Periodontitis—The EFP S3 Level Clinical Practice Guideline,” Journal of Clinical Periodontology 47, no. S22 (2020): 4–60. 32383274 10.1111/jcpe.13290 PMC7891343

[99] A. M. Polson and J. G. Caton , “Current Status of Bleeding in the Diagnosis of Periodontal Diseases,” Journal of Periodontology 56, no. 11 (1985): 1–3. 10.1902/jop.1985.56.11s.1 3878401

[100] G. Greenstein , J. Caton , and A. M. Poison , “Histologie Characteristics Associated With Bleeding After Probing and Visual Signs of Inflammation,” Journal of Periodontology 52, no. 8 (1981): 420–425. 6973623 10.1902/jop.1981.52.8.420

[101] P. I. Eke , L. Wei , G. O. Thornton‐Evans , et al., “Risk Indicators for Periodontitis in US Adults: NHANES 2009 to 2012,” Journal of Periodontology 87, no. 10 (2016): 1174–1185. 27367420 10.1902/jop.2016.160013 PMC11370315

[102] C. Tsai , C. Hayes , and G. W. Taylor , “Glycemic Control of Type 2 Diabetes and Severe Periodontal Disease in the US Adult Population,” Community Dentistry and Oral Epidemiology 30, no. 3 (2002): 182–192. 12000341 10.1034/j.1600-0528.2002.300304.x

[103] International Diabetes Federation , “What Is Diabetes,” (2024), https://idf.org/about‐diabetes/what‐is‐diabetes/.

[104] P. I. Eke , G. O. Thornton‐Evans , L. Wei , W. S. Borgnakke , B. A. Dye , and R. J. Genco , “Periodontitis in US Adults: National Health and Nutrition Examination Survey 2009–2014,” Journal of the American Dental Association 149, no. 7 (2018): 576–588.e6. 29957185 10.1016/j.adaj.2018.04.023 PMC8094373

[105] L. Heasman , F. Stacey , P. M. Preshaw , G. I. McCracken , S. Hepburn , and P. A. Heasman , “The Effect of Smoking on Periodontal Treatment Response: A Review of Clinical Evidence,” Journal of Clinical Periodontology 33, no. 4 (2006): 241–253. 16553633 10.1111/j.1600-051X.2006.00902.x

[106] D. Zandbergen , D. E. Slot , R. Niederman , and F. A. Van der Weijden , “The Concomitant Administration of Systemic Amoxicillin and Metronidazole Compared to Scaling and Root Planing Alone in Treating Periodontitis: A Systematic Review,” BMC Oral Health 16, no. 1 (2016): 27. 26928597 10.1186/s12903-015-0123-6 PMC4770674

[107] C. M. M. L. Santos , R. Lira , R. G. Fischer , A. P. P. Santos , and B. H. Oliveira , “Systemic Antibiotics in Periodontal Treatment of Diabetic Patients: A Systematic Review,” PLoS One 10, no. 12 (2015): e0145262. 26693909 10.1371/journal.pone.0145262 PMC4687852

