# Supplementary material for: The Effect of Diabetes on Outcomes of Non‐Surgical Periodontal Therapy: A Systematic Review With a Meta‐Analysis and Trial Sequential Analysis
Source: Int J Dent Hyg. 2025 Dec 18;24(2):321–52. doi: 10.1111/idh.70003 (PMC13050388; doi:10.1111/idh.70003)
Supplement: Supplementary file 1 — Appendix S1: idh70003‐sup‐0001‐supinfo.pdf. [file IDH-24-321-s001.pdf]

Online Appendices

The Effect of Diabetes on Outcomes of Non-Surgical Periodontal Therapy: A Systematic Review and Meta-analysis

L.P.M. Weijdijk

T.M.J.A. Thomassen

N. de Keyzer

E.E.J. Mayer

C. Valkenburg

G.A. van der Weijden

D.E. Slot

Online Appendix S1

Excluded studies (N=8) based after the seconds phase of the search and selection procedure. When based on the full-text papers were assessed for the defined eligibility criteria.

Reason	Author
Additional (surgical) therapy	1. Westfelt et al. 1996<br>2. Costa et al. 2013
No intervention of NSPT	3. Collin et al. 1998<br>4. Tanwir et al. 2009<br>5. Haseeb et al. 2012
Language restriction	6. Shen et al. 2008 (Chinese)
Deviant parameter of interest	7. Rael et al. 2021 (tooth loss)
No (healthy) control group	8. Sinha et al. 2021<br>9. Kolte et al. 2023

References:

1. Westfelt E, Rylander H, Blohmé G, Jonasson P, Lindhe J. The effect of periodontal therapy in diabetics. Results after 5 years. J Clin Periodontol. 1996 Feb;23(2):92-100. doi: 10.1111/j.1600-051x.1996.tb00540.x. PMID: 8849844.
2. Costa FO, Miranda Cota LO, Pereira Lages EJ, Soares Dutra Oliveira AM, Dutra Oliveira PA, Cyrino RM, Medeiros Lorentz TC, Cortelli SC, Cortelli JR. Progression of periodontitis and tooth loss associated with glycemic control in individuals undergoing periodontal maintenance therapy: a 5-year follow-up study. J Periodontol. 2013 May;84(5):595-605. doi: 10.1902/jop.2012.120255. Epub 2012 Jul 6. PMID: 22769441.
3. Collin HL, Uusitupa M, Niskanen L, Kontturi-Närhi V, Markkanen H, Koivisto AM, Meurman JH. Periodontal findings in elderly patients with non-insulin dependent diabetes mellitus. J Periodontol. 1998 Sep;69(9):962-6. doi: 10.1902/jop.1998.69.9.962. PMID: 9776023.
4. Almas, Khalid & Al-Qahtani, Mohammed & Al-Yami, Marzouk & Khan, Nazeer. (2001). The Relationship Between Periodontal Disease And Blood Glucose Level Among Type II Diabetic Patients. The journal of contemporary dental practice. 2. 18-25. 10.5005/jcdp-2-4-1.
5. Haseeb M, Khawaja KI, Ataullah K, Munir MB, Fatima A. Periodontal disease in type 2 diabetes mellitus. J Coll Physicians Surg Pak. 2012 Aug;22(8):514-8. PMID: 22868018.
6. Shen CJ, Yin YZ, Shu R. [The effect of initial periodontal therapy on metabolic control in type 2 diabetes mellitus]. Shanghai Kou Qiang Yi Xue. 2008 Feb;17(1):6-9. Chinese. PMID: 18360659.
7. Raedel M, Noack B, Priess HW, Bohm S, Walter MH. Massive data analyses show negative impact of type 1 and 2 diabetes on the outcome of periodontal treatment. Clin Oral Investig. 2021 Apr;25(4):2037-2043. doi: 10.1007/s00784-020-03512-0. Epub 2020 Aug 20. PMID: 32820433; PMCID: PMC7966218.
8. Sinha S, Sonoo, PR, Siddhartha R, Singh SK, Singh A. (2021). Effect of conventional periodontal treatment (Scaling and Root Planing) on Type-2 diabetic patient with moderate generalized chronic periodontitis: A clinical study. Journal of Pharmacy & Bioallied Sciences, 13(Suppl 1), S706.
9. Kolte RA, Kolte AP, Bawankar PV, Bajaj VA. Effect of Nonsurgical Periodontal Therapy on Metabolic Control and Systemic Inflammatory Markers in Patients of Type 2 Diabetes Mellitus with Stage III Periodontitis. *Contemp Clin Dent*. 2023;14(1):45-51.

Online Appendix S2.1
Primary outcomes

Periodontal pocket depth (PPD)

PPD, the depth of the periodontal pocket using a periodontal probe, is measured from gingival margin to the base of the periodontal pockets with the tip of the periodontal probe. It is utilized as an indicator to evaluate the severity of periodontal disease and track the effectiveness of NSPT. PPD is an important clinical indicator for determining the severity of periodontal disease and is expressed in mm.

Clinical attachment level (CAL)

CAL is used to assess the loss of periodontal tissue support in periodontitis and to monitor disease progression as well as the effect of NSPT on the periodontium (1). CAL is measured with a periodontal probe as the distance from the cement-enamel joint (CEJ) to the base of the pocket (2).
To interpret the results a clinical relevance scale was utilized prior to assessing the outcomes. The clinical relevance of CAL data was defined as follows: 0-0.2 zero effect, >0.2-0.4 small effect, >0.4-0.6 moderate effect and >0.6 substantial effect (3).

The primary outcomes, PPD and CAL, were chosen due to their wide recognition and reliability as measures of periodontal health. PPD reflects the inflammatory status of periodontal tissues, with increased probing depth indicating inflammation or edema. However, PPD alone cannot differentiate between periodontal health, gingivitis, or periodontitis, as it may occur without attachment loss. Therefore, it should be interpreted in relation to the cementoenamel junction (CEJ) for more accurate assessment. CAL, on the other hand, measures the loss of periodontal attachment due to connective tissue breakdown and apical migration of the junctional epithelium, reflecting past disease progression. While increased PPD indicates current disease activity, CAL offers insight into cumulative attachment loss. Together, these two parameters are essential for evaluating periodontal health, disease progression, and the success of therapy (4).

References:

1. Farook FF, Alodwene H, Alharbi R, Alyami M, Alshahrani A, Almohammadi D, et al. Reliability assessment between clinical attachment loss and alveolar bone level in dental radiographs. Clin Exp Dent Res. 2020 Sep 12;6(6):596–601.
2. Highfield J. Diagnosis and classification of periodontal disease. Aust Dent J. 2009 Sep 3;54(1):11–26.
3. Smiley CJ, Tracy SL, Abt E, Michalowicz BS, John MT, Gunsolley J, et al. Evidence-based clinical practice guideline on the nonsurgical treatment of chronic periodontitis by means of scaling and root planing with or without adjuncts. The Journal of the American Dental Association. 2015 Jul;146(7):525–35.
4. Heitz-Mayfield LJA. Conventional diagnostic criteria for periodontal diseases (plaque-induced gingivitis and periodontitis). Periodontol 2000. 2024 Jun;95(1):10-19. doi: 10.1111/prd.12579. Epub 2024 Jun 3. PMID: 38831568.

Appendix S2.2

Secondary outcomes

Gingivitis Indices

Bleeding on probing (BOP)—Ainamo & Bay 1975

BOP was recorded as positive or negative and is regarded as an objective indicator of gum inflammation (1). The record of BOP was either present or absent within 30 seconds of probing at six sites per tooth and recorded as the average number of teeth BOP for each group (2, 3).

Gingival Bleeding Index (GBI) - Ainamo & Bay 1975

The Gingival Bleeding Index (GBI), introduced by Ainamo & Bay (1975), is performed through gentle probing of the orifice of the gingival crevice. If bleeding occurs within 10 seconds a positive finding is recorded and the number of positive sites is recorded and then expressed as a percentage of the number of sites examined (1).

Papilla bleeding index (PBI)—Saxer & Mühlemann 1975

The is a sensitive indicator of the severity of gingival inflammation in individual patients. The PBI does not require a great amount of time, since only 28 measurement sites in the complete dentition are evaluated (4).

Sulcus Bleeding Index (SBI) – Mühlemann and Son

An early sign of gingivitis is bleeding on probing and, in 1971, Muhlemann and Son described the Sulcus Bleeding Index (SBI). The criteria for scoring are as follows: Score 0 – health looking papillary and marginal gingiva no bleeding on probing; Score 1 – healthy looking gingiva, bleeding on probing; Score 2 – bleeding on probing, change in color, no edema; Score 3 – bleeding on probing, change in color, slight edema; Score 4 –bleeding on probing, change in color, obvious edema; Score 5 –spontaneous bleeding, change in color, marked edema. Four gingival units are scored systematically for each tooth: the labial and lingual marginal gingival (M units) and the mesial and distal papillary gingival (P units). Scores for these units are added and divided by four. Adding the scores of the undivided teeth and dividing them by the number of teeth can determine the sulcus bleeding index (5).

Gingival index (GI)—Löe & Silness 1963

The following scoring system is used for GI: 0 = normal gingiva; 1 = mild inflammation: slight change in color, slight oedema, no bleeding on probing; 2 = moderate inflammation: redness, oedema, and glazing, or bleeding on probing; 3 = severe inflammation: marked redness and oedema, tendency toward spontaneous bleeding, ulceration. The scores obtained from the four areas of the tooth can be aggregated and divided by four to calculate the GI for the tooth (6).

Plaque Indices

Plaque index (PI)—O'Leary et al. 1972

The plaque control record presented by O'Leary, Drake and Naylor (1972), makes use of a chart in which each of the 4 surfaces of each tooth (but not the occlusal or incisal surface) is represented. Both a disclosant and an explorer are used to establish the presence of plaque. Any plaque at the dento-gingival junction,

irrespective of its amount, is recorded. The prescenes of plaque (+) or absence (-) is recorded in a chart and the plaque indices are expressed as an exact precentage (7).

Approximal plaque index (API)—Lange 1986

Following application of disclosing solution, a simple yes/no decision is made concerning wheter the examined interproximal surfaces are covered by plaque (+) or not (-). The proportion of plaque-covered interproximal spaces is expressed as percentage. Usually, analogous to the pailla bleeding index (PBI), in a given quadrant the interproximal spaces are scores from only one aspect. It correlates well with the PBI (8).

Plaque index (PI)—Silness & Löe 1964

This index ascertains the thickness of plaque along the gingival margin. To visualize plaque, teeth are dried with air, the plaque is not stained. The PI is inidcated for epidemiological studies in wich the GI is recored simultaneously. The results of the PI were interpreted as follows: no plaque (grade 0), thin plaque layer at the gingival margin; only detectable by scraping with a probe (grade 1), moderate layer of plaque along the gingival margin; interdental spaces free but plaque is visible (grade 2), abundant plaque along the gingival margin; interdental spaces filled with plaque (grade 3) (9).

Visible Plaque Index (VPI) - Ainamo & Bay 1975

The Visible Plaque Index (VPI) was proposed by Ainamo and Bay to assess the quality of oral hygiene through clinical observation of the presence of biofilm on dental surfaces by means of simple categorical definitions (presence or absence of plaque (1).

Gingival recession

It is defined as the apical migration of the gingival margin below the cementoenamel junction, resulting in exposure of the root surface. Gingival recession is measured clinically as the distance from the cementoenamel junction to the depth of the free gingival margin using a periodontal probe. It reflects the exposure of the root cementum (10,11). Difference in gingival recession reduction after NSPT between the NDM and periodontitis patients is measured in mm.

References

1. Ainamo J, Bay I. Problems and proposals for recording gingivitis and plaque. Int Dent J. 1975;25(4):229–35.
2. Lang NP, Joss A, Orsanic T, Gusberti FA, Siegrist BE. Bleeding on probing. A predictor for the progression of periodontal disease? J Clin Periodontol. 1986 Jul;13(6).
3. Hugoson A, Sjödin B, Norderyd O. Trends over 30 years, 1973–2003, in the prevalence and severity of periodontal disease. J Clin Periodontol. 2008 May;35(5):405–14.
4. Saxer UP, Mühlemann HR. Motivation and education. Monatsschr Zahnheilkd. 1975;85(9):905–19.
5. Mühlemann HR, Son S. Gingival sulcus bleeding--a leading symptom in initial gingivitis. Helv Odontol Acta. 1971;15(2):107–13.
6. Löe H. The Gingival Index, the Plaque Index and the Retention Index Systems. J Periodontol. 1967 Nov;38(6):610–6.
7. O’Leary TJ, Drake RB, Naylor JE. The plaque control record. J Periodontol. 1972 Jan;43(1):38.
8. Lange DE, Plagmann HC, Eenboom A, Promesberger A. A. Klinische Bewertungsverahren zur Objektivierung der Mundhygiene [Clinical methods for the objective evaluation of oral hygiene]. Dtsch Zahnarztl Z. 1977 Jan;32(1):44–7.
9. Silness J, Loe H. Löe and Silness Plaque Index (PI). Acta Odontol Scand. 1964;22.
10. Goldman HM, Cohen DW. Periodontal therapy. CV Mosby Company. 1973;
11. Newman MG, Takei H, Klokkeveld PR, Carranza FA, editors. Carranza’s clinical periodontology. St. Louis : Elsevier; 2014.

Online Appendix S3

Risk of Bias assessment using a comprehensive combination of criteria as suggested by the critical appraisal checklist for analytical cross-sectional studies, the Newcastle Ottawa scale adapted for cross sectional studies, and the ROBINS-E tool. Judgement of risk of bias is presented according to the 7 domains as suggested by the ROBINS-E tool.

**Included studies:**	I Tervonen et al. 1991	II Tervonen et al. 1997	III Christgau et al. 1998	IV Sonoki et al. 2006	V Navarro Sanchez et al. 2007	VI Da Cruz et al. 2008	VII Correa et al. 2008	VIII Conçalves et al. 2008	IX Dag et al. 2009
**Pre-assessment domains**									
**1. Bias due to confounding**									
■ Were confounding factors defined?	No	No	Yes	Yes	Yes	Yes	No	No	No
■ Were confounding factors assessed?	NR	NR	Yes	No	Yes	Yes	Yes	Yes	Yes
■ Were strategies to deal with confounding factors stated?	No	Yes	Yes	Yes	Yes	Yes	Yes	Yes	Yes
Risk of bias judgement	*Critical*	*Serious*	*Low*	*Moderate*	*Low*	*Low*	*Moderate*	*Moderate*	*Moderate*
**2. Bias arising from measurement of the exposure**									
Risk of bias judgement	*Low*	*Low*	*Low*	*Low*	*Low*	*Low*	*Low*	*Low*	*Low*
**3. Bias in selection of participants into the study (or into the analysis)**									
■ Were the criteria for inclusion in the sample clearly defined?	No	No	Yes	Yes	Yes	Yes	Yes	Yes	Yes
■ Were the study subjects described in detail?	Yes	Yes	Yes	Yes	Yes	Yes	Yes	Yes	Yes
■ Is the study sample representative of the average in the target population?	Yes	Yes	Yes	Yes	Yes	Yes	Yes	Yes	Yes
■ Is the sample size justified and satisfactory?	NR	NR	NR	NR	NR	NR	NR	Yes	NR
Risk of bias judgement	*Serious*	*Serious*	*Moderate*	*Moderat e*	*Moderate*	*Moderate*	*Moderate*	*Low*	*Moderate*

Post-assessment domains

4. Bias due to post-exposure interventions

Risk of bias judgement	*Low*	*Low*	*Low*	*Low*	*Low*	*Low*	*Low*	*Low*	Low

5. Bias due to missing data

■ Were outcome data available for (nearly) all participants?	Yes	Yes	Yes	No	Yes	Yes	Yes	Yes	Yes
Risk of bias judgement	*Low*	*Low*	*Low*	*Moderate*	*Low*	*Low*	*Low*	*Low*	*Low*

6. Bias arising from the measurement of the outcome

■ Were the investigators blinded to the condition?	Yes	Yes	Yes	NR	Yes	NR	NR	NR	NR
■ Were the investigators calibrated?	NR	NR	Yes	NR	Yes	NR	Yes	Yes	NR
■ Were the outcomes measured in a valid and reliable way?	Yes	Yes	Yes	Yes	Yes	Yes	Yes	Yes	Yes
■ Was appropriate statistical analysis used?	Yes	Yes	Yes	Yes	Yes	Yes	Yes	Yes	Yes
Risk of bias judgement	*Moderate*	*Moderate*	*Low*	*Serious*	*Low*	*Serious*	*Moderate*	*Moderate*	*Serious*

7. Bias in selection of the reported result

■ Are the reported effect estimates based on the results?	Yes	Yes	Yes	Yes	Yes	Yes	Yes	Yes	Yes
Risk of bias judgement	*Low*	*Low*	*Low*	*Low*	*Low*	*Low*	*Low*	*Low*	*Low*

**OVERALL Risk of bias**	Critical	Serious	Moderate	Serious	Moderate	Serious	Moderate	Moderate	Serious

Judgements: Low, moderate, serious, critical, not reported (NR), not applicable (NA)

Online Appendix S3 (continued)

Risk of Bias assessment using a comprehensive combination of criteria as suggested by the critical appraisal checklist for analytical cross-sectional studies, the Newcastle Ottawa scale adapted for cross sectional studies, and the ROBINS-E tool. Judgement of risk of bias is presented according to the 7 domains as suggested by the ROBINS-E tool.

**Included studies:**	X Kardesler et al. 2009	XI Kudva et al. 2010	XII Hungund et al. 2012	XIII Cirano et al. 2012	XIV Camargo et al. 2013	XV Buzinin et al. 2014	XVI López et al. 2013	XVII Kara et al. 2015	XVIII Kaur et al. 2015
**Pre-assessment domains**									
**1. Bias due to confounding**									
■ Were confounding factors defined?	No	No	No	No	No	No	Yes	No	Yes
■ Were confounding factors assessed?	Yes	Yes	Yes	Yes	Yes	Yes	Yes	Yes	Yes
■ Were strategies to deal with confounding factors stated?	Yes	Yes	Yes	Yes	Yes	Yes	Yes	Yes	Yes
Risk of bias judgement	*Moderate*	*Moderate*	*Moderate*	*Moderate*	*Moderate*	*Moderate*	*Low*	*Moderate*	*Low*
**2. Bias arising from measurement of the exposure**									
Risk of bias judgement	*Low*	*Low*	*Low*	*Low*	*Low*	*Low*	*Low*	*Low*	*Low*
**3. Bias in selection of participants into the study (or into the analysis)**									
■ Were the criteria for inclusion in the sample clearly defined?	Yes	Yes	Yes	Yes	Yes	Yes	Yes	Yes	Yes
■ Were the study subjects described in detail?	Yes	No	Yes	Yes	Yes	Yes	Yes	Yes	Yes
■ Is the study sample representative of the average in the target population?	Yes	Yes	Yes	Yes	Yes	Yes	Yes	Yes	Yes
■ Is the sample size justified and satisfactory?	NR	NR	NR	Yes	Yes	Yes	NR	Yes	Yes
Risk of bias judgement	*Moderate*	*Serious*	*Moderate*	*Low*	*Low*	*Low*	*Moderate*	*Low*	*Low*

Post-assessment domains

4. Bias due to post-exposure interventions

Risk of bias judgement	*Low*	*Low*	*Low*	*Low*	*Low*	*Low*	*Low*	*Low*	Low

5. Bias due to missing data

■ Were outcome data available for (nearly) all participants?	Yes	Yes	Yes	Yes	Yes	Yes	Yes	Yes	Yes
Risk of bias judgement	*Low*	*Low*	*Low*	*Low*	*Low*	*Low*	*Low*	*Low*	*Low*

6. Bias arising from the measurement of the outcome

■ Were the investigators blinded to the condition?	NR	NR	NR	NR	NR	NR	No	Yes	Yes
■ Were the investigators calibrated?	NR	NR	NR	Yes	NR	Yes	Yes	Yes	Yes
■ Were the outcomes measured in a valid and reliable way?	Yes	Yes	Yes	Yes	Yes	Yes	Yes	Yes	Yes
■ Was appropriate statistical analysis used?	Yes	Yes	NR	Yes	Yes	Yes	Yes	Yes	Yes
Risk of bias judgement	*Serious*	*Serious*	*Serious*	*Moderate*	*Serious*	*Moderate*	*Moderate*	*Low*	*Low*

7. Bias in selection of the reported result

■ Are the reported effect estimates based on the results?	Yes	Yes	Yes	Yes	Yes	Yes	Yes	Yes	Yes
Risk of bias judgement	*Low*	*Low*	*Low*	*Low*	*Low*	*Low*	*Low*	*Low*	*Low*

**OVERALL Risk of bias**	Serious	Serious	Serious	Moderate	Serious	Moderate	Moderate	Moderate	Low

Judgements: Low, moderate, serious, critical, not reported (NR), not applicable (NA)

Online Appendix S3 (continued)

Included studies:	XIX Pannicker et al. 2015	XX Dogan et al. 2016	XXI Mishra et al. 2016	XXII Abreu et al. 2015	XXIII Perruzo Lopes et al. 2017	XXIV Sundaram et al. 2017	XXV Gayathri et al. 2019	XXVI Ahuja et al. 2019	XXVII Pragda et al. 2019	XXVIII Almeida et al. 2019	XXIX Mirnic et al. 2022	XXX Gomathi et al. 2023
**Pre-assessment domains**												
**1. Bias due to confounding**												
■ Were confounding factors defined?	No	Yes	No	No	No	No	No	No	No	Yes	No	No
■ Were confounding factors assessed?	Yes	Yes	Yes	Yes	Yes	Yes	No	Yes	Yes	Yes	Yes	No
■ Were strategies to deal with confounding factors stated?	Yes	Yes	Yes	Yes	Yes	Yes	No	Yes	Yes	Yes	Yes	No
Risk of bias judgement	*Moderate*	*Low*	*Moderate*	*Moderate*	*Moderate*	*Moderate*	*Critical*	*Moderate*	*Moderate*	*Low*	*Moderate*	*Critical*
**2. Bias arising from measurement of the exposure**												
Risk of bias judgement	*Low*	*Low*	*Low*	*Low*	*Low*	*Low*	*Low*	*Low*	*Low*	*Low*	*Low*	*Low*
**3. Bias in selection of participants into the study (or into the analysis)**												
■ Were the criteria for inclusion in the sample clearly defined?	Yes	Yes	Yes	Yes	Yes	Yes	Yes	Yes	Yes	Yes	Yes	Yes
■ Were the study subjects described in detail?	Yes	Yes	Yes	Yes	Yes	Yes	No	Yes	No	Yes	Yes	No
■ Is the study sample representative of the average in the target population?	Yes	Yes	Yes	Yes	Yes	Yes	Yes	Yes	Yes	Yes	Yes	Yes
■ Is the sample size justified and satisfactory?	NR	Yes	Yes	NR	NR	NR	NR	Yes	Yes	Yes	NR	NR
Risk of bias judgement	*Moderate*	*Low*	*Low*	*Moderate*	*Moderate*	*Moderate*	*Serious*	*Low*	*Moderate*	*Low*	*Moderate*	*Serious*

Post-assessment domains

4. Bias due to post-exposure interventions

Risk of bias judgement	*Low*	*Low*	*Low*	*Low*	*Low*	*Low*	*Low*	*Low*	*Low*	*Low*	*Low*	*Low*

5. Bias due to missing data

■ Were outcome data available for (nearly) all participants?	Yes	Yes	Yes	Yes	Yes	Yes	Yes	Yes	Yes	Yes	Yes	Yes
Risk of bias judgement	*Low*	*Low*	*Low*	*Low*	*Low*	*Low*	*Low*	*Low*	*Low*	*Low*	*Low*	*Low*

6. Bias arising from the measurement of the outcome

■ Were the investigators blinded to the condition?	NR	Yes	NR	NR	Yes	NR	NR	NR	NR	Yes	NR	NR
■ Were the investigators calibrated?	Yes	Yes	Yes	Yes	NR	NR	NR	Yes	NR	Yes	NR	NR
■ Were the outcomes measured in a valid and reliable way?	Yes	Yes	Yes	Yes	Yes	Yes	Yes	Yes	Yes	Yes	Yes	Yes
■ Was appropriate statistical analysis used?	Yes	Yes	Yes	Yes	Yes	Yes	Yes	Yes	Yes	Yes	Yes	Yes
Risk of bias judgement	*Moderate*	*Low*	*Moderate*	*Moderate*	*Moderate*	*Serious*	*Serious*	*Moderate*	*Serious*	*Low*	*Serious*	*Serious*

7. Bias in selection of the reported result

■ Are the reported effect estimates based on the results?	Yes	Yes	Yes	Yes	Yes	Yes	Yes	Yes	Yes	Yes	Yes	Yes
Risk of bias judgement	*Low*	*Low*	*Low*	*Low*	*Low*	*Low*	*Low*	*Low*	*Low*	*Low*	*Low*	*Low*

**OVERALL Risk of bias**	Moderate	Low	Moderate	Moderate	Moderate	Serious	Critical	Moderate	Serious	Low	Serious	Critical

Judgements: Low, moderate, serious, critical, not reported (NR), not applicable (NA)

Online Appendix S4

Overview of included studies processed for data extraction

Phase/type of the treatment	Selection ID<br>I<br>Tervonen et al.1991	II<br>Tervonen et al.1997	III<br>Christgau et al. 1998	IV<br>Sonoki et al. 2006	V<br>Navarro Sanchez et al. 2007	VI<br>Da Cruz et al. 2008	VII<br>Correa et al. 2008	VIII<br>Gonçalves et al. 2008	IX<br>Dag et al. 2009	X<br>Kardesler et al. 2009	XI<br>Kudva et al. 2010	XII<br>Hungund et al. 2012	XIII<br>Cirano et al. 2012	XIV<br>Camargo et al. 2013
**Preliminary phase:**														
Patient motivation	+	+	+	NR	NR	NR	NR	NR	NR	NR	NR	NR	NR	NR
Oral hygiene instructions	+	+	+	+	+	+	+	+	+	NR	NR	+	NR	+
Supragingival plaque removal/supragingival scaling	NR	NR	+	+	+	?	+	+	NR	NR	NR	+	NR	+
Information/instructions about periodontal disease	NR	+	NR	NR	NR	NR	NR	NR	NR	NR	NR	+	NR	NR
**Restorative:**														
Restorations	+	+	+	+	NR	NR	NR	NR	+	NR	NR	NR	NR	NR
Removal of plaque retaining restorations	NR	+	+	+	NR	NR	NR	NR	NR	NR	NR	NR	NR	NR
Extractions	NR	NR	+	+	NR	NR	NR	NR	+	NR	NR	NR	NR	NR
**SRP phase:**														
Type of instrumentation	mi + usi	NR	hi	NR	mi + usi	NR	hi	NR	mi + usi	NR	mi + usi	hi	usi	NR
Duration of an appointment provided	NR	NR	NR	NR	1h	2h	NR	1h	Unlimited	NR	NR	NR	45m	NR
Number of treatment sessions provided	dpp	NR	4	6	4	1	4	4	dpp	4	1	NR	1	1
Use of anesthesia	NR	NR	+	NR	NR	+	+	+	NR	NR	NR	+	NR	+
Pocket irrigation with antimicrobial solution	NR	NR	+	NR	NR	NR	NR	NR	NR	NR	NR	NR	NR	NR
**Post-SRP phase:**														
Supportive periodontal therapy provided	NR	NR	NR	+	NR	NR	+	+	NR	+	NR	NR	+	NR
Oral hygiene re-instructions	NR	+	NR	NR	+	+	+	+	?	+	NR	+	+	+
Follow up	3-4m	4w, 6m, 12m	2wk, 4m	5.5-6.5m	3m, 6m	3m	3m	3m	3m	1m, 3m	3m	3m, 6m	3m, 6m	3m

Phase/type of the treatment	XV Buzinin et al. 2014	XVI López et al. 2013	XVII Kara et al. 2015	XVIII Kaur et al. 2015	XIX Pannicker et al. 2015	XX Dogan et al. 2016	XXI Mishra et al. 2016	XXII Abreu et al. 2015	XXIII Perruzo Lopes et al. 2017	XXIV Sundaram et al. 2017	XXV Ahuja et al. 2019	XXVI Gayathri et al. 2019	XXVII Pragada et al. 2019	XXVIII Almeida et al. 2019	XXIX Mirnic et al. 2022	XXX Gomathi et al. 2023
**Preliminary phase:**																
Patient motivation	NR	NR	NR	NR	NR	NR	NR	NR	+	NR	NR	NR	NR	NR	NR	NR
Oral hygiene instructions	NR	+	NR	+	NR	+	NR	+	+	+	+	+	+	+	+	NR
Supragingival plaque removal/supragingival scaling	NR	+	NR	NR	NR	NR	NR	+	+	NR	+	NR	+	+	NR	NR
Information/instructions about periodontal disease	NR	NR	NR	NR	NR	NR	NR	NR	NR	NR	NR	NR	NR	NR	NR	NR
**Restorative:**																
Restorations	NR	+	NR	NR	NR	NR	NR	NR	NR	NR	NR	NR	NR	+	NR	NR
Removal of plaque retaining restorations	NR	NR	NR	NR	NR	NR	NR	NR	NR	NR	NR	NR	NR	+	NR	NR
Extractions	NR	+	NR	NR	NR	NR	NR	NR	NR	NR	NR	NR	NR	+	NR	NR
**SRP phase:**																
Type of instrumentation	mi + usi	usi	mi + usi	mi + usi	mi + usi	mi + usi	NR	NR	NR	NR	NR	NR	mi+usi	mi + usi	mi + usi	mi
Duration of an appointment provided	NR	dpp	NR	NR	NR	45-60min	NR	NR	NR	NR	NR	NR	NR	NR	1h	NR
Number of treatment sessions provided	1	NR	2	4	NR	2	NR	4-6	NR	NR	NR	NR	2	1	1-2	2
Use of anesthesia	NR	NR	NR	NR	NR	+	NR	NR	NR	+	NR	NR	+	NR	NR	+
Pocket irrigation with antimicrobial solution	NR	NR	NR	NR	NR	NR	NR	NR	NR	NR	NR	NR	NR	+	NR	NR
**Post-SRP phase:**																
Supportive periodontal therapy provided	NR	+	+	+	NR	NR	NR	+	+	+	NR	NR	NR	+	NR	NR
Oral hygiene re-instructions	NR	+	+	+	+	NR	NR	+	+	+	+	NR	NR	+	NR	NR
	2m, 3m	3m, 6m, 9m	1m, 3m	3m, 6m	6wk	6wk	1m	1m	6m	3m	6m	6wk	12m	12m	3m	6wk

+: Yes, -: No, NR: Not reported, ?: Not specified/unclear? = unknown/not reported, mi = manual instrumentation, usi = ultrasonic instrumentation, h: hour(s), m: minutes, dpp: depends per person

Online Appendix S5.1

A descriptive table of statistical significance levels of the difference between poor or good controlled DM and NDM regarding to primary and secondary clinical parameters

Author	Exposure	Primary outcomes		Secondary outcomes			Comparison
		CAL	PPD	Gingivitis indices	Plaque indices	REC	
**Sub-analysis DM type I**							
II Tervonen et al. 1997	DMT I	0	0	0	0	□	NDM
XV Buzizin et al. 2014	DMT I	0	0	0	0	□	NDM
XXIII Perruzo Lopes et al. 2017	DMT I	+	0	-	+	□	NDM
**Total**<br>**+ DM**<br>**+ NDM**<br>**No difference**		 **1/3**<br>**0/3**<br>**2/3**	 **0/3**<br>**0/3**<br>**3/3**	 **0/3**<br>**1/3**<br>**2/3**	 **1/3**<br>**0/3**<br>**2/3**	 **NA**	
**Sub-analysis DM type II**							
IV Sonoki et al. 2006	DMT II	□	0	0	□	□	NDM
V Navarro-Sanchez et al. 2007/Faria-Almeida et al. 2006	DMT II	0	-	0	0	0	NDM
VI Da Cruz et al. 2008	DMT II	0	0	0	0	0	NDM
VII Correa et al. 2008	DMT II	0	0	-	-	□	NDM
VIII Conçalves et al. 2008/Silva-Boghossian et al. 2014	DMT II	0	0	-	-	□	NDM
IX Dag et al. 2009	DMT II	?	?	?	?	□	NDM
X Kardesler et al. 2009	DMT II	?	?	?	?	□	NDM
XI Kudva et al. 2010	DMT II	□	0	0	0	□	NDM
XII Hungund et al. 2012	DMT II	□	?	?	?	□	NDM
XIII Cirano et al. 2012	DMT II	0	0	0	0	0	NDM
XIV Camargo et al. 2013	DMT II	0	0	0	0	0	NDM
XVI López et al. 2013	DMT II	0	0	0	0	□	NDM
XVII Kara et al. 2015	DMT II	0	0	0	0	□	NDM
XVIII Kaur et al. 2015	DMT II	?	?	?	?	□	NDM
XIX Pannicker et al. 2015	DMT II	0	0	0	0	□	NDM

XX Dogan et al. 2016	DMT II	0	0	0	0	□	NDM
XXI Mishra et al. 2016	DMT II	□	-	-	0	□	NDM
XXII Abreu et al. 2015	DMT II	0	0	0	0	□	NDM
XXIII Perruzo Lopes et al. 2017	DMT II	0	0	-	0	□	NDM
XXIV Sundaram et al. 2017	DMT II	?	?	?	?	□	NDM
XXV Gayathri et al. 2019	DMT II	0	0	-	□	□	NDM
XXVI Ahuja et al. 2019	DMT II	-	-	-	-		NDM
XXVII Pragada et al. 2019	DMT II	0	0	0	0	□□	NDM
XXVIII Almeida et al. 2019	DMT II	0	0	0	-	□	NDM
XXIX Mirnic et al. 2021	DMT II	0	-	0	0	□	NDM
XXX Gomathi et al. 2023	DMT II	-	-	-	-		NDM
**Total**<br>**+ DM**<br>**+ NDM**<br>**No difference**		**0/18**<br>**2/18**<br>**16/18**	**0/22**<br>**5/22**<br>**17/22**	**0/21**<br>**7/21**<br>**14/21**	**0/19**<br>**5/19**<br>**14/19**	**0/4**<br>**0/4**<br>**4/4**	
**Sub-analysis DM type I/II**							
I Tervonen et al. 1991	DMT I/II	□	0	0	□	□	NDM
III Christgau et al. 1998	DMT I/II	0	0	0	0	□	NDM
**Total**<br>**+ DM**<br>**+ NDM**<br>**No difference**		**0/1**<br>**0/1**<br>**1/1**	**0/2**<br>**0/2**<br>**2/2**	**0/2**<br>**0/2**<br>**2/2**	**0/1**<br>**0/1**<br>**1/1**	**NA**	

A positive sign (+) implies that the DM group had significant more reduction of parameters or CAL gain than the NDM group. With a negative sign (-) the DM group had significant less reduction or gain than the NDM group. No differences between the two groups were assigned with the number zero (0).

Abbreviations: □, no data available due to lack of evaluation, + significant higher (percentage) of reduction for DM, - significant higher (percentage) of reduction for NDM (for GI/CAL higher numbers), 0 no difference between DM group and non-DM, ? no inter-group evaluation between DM and NDM, *CAL* clinical attachment level, *PPD* periodontal pocket depth, *BLI* bone loss index, *BOP* bleeding on probing, *GI* gingival index, *DMT I* diabetic mellitus type I, *DMT II* diabetic mellitus type II, *DMT B* diabetic mellitus type I or II or both, PI* Silness & Löe 1964, PI** O'Leary et al. 1972, PI recorded at 6 sites (yes/no), VPI visible plaque index following Ainamo

Online Appendix S5.2

A descriptive table of statistical significance levels of the difference between poor or good controlled DM and NDM regarding to primary and secondary clinical parameters.

**Author**	Exposure	**Primary outcomes**		**Secondary outcomes**			Comparison
		CAL	PPD	Gingivitis indices	Plaque indices	REC	
**Analysis poor controlled DM**							
VIII Conçalves et al. 2008/Silva-Boghossian et al. 2014	DM -	0	0	-	-	□	NDM
IX Dag et al. 2009	DM -	0	0	0	0	□	NDM
X Kardesler et al. 2009	DM -	0	0	-	0	□	NDM
XIII Cirano et al. 2012	DM -	0	0	0	0	0	NDM
XVI López et al. 2013	DM -	0	0	0	0	□	NDM
XVIII Kaur et al. 2015	DM -	0	**-**	-	0	□	NDM
XXIV Sundaram et al. 2017	DM -	?	?	?	?	□	NDM
XXIX Mirnic et al. 2021	DM -	0	-	0	0	□	NDM
**Total**<br>**+ DM**<br>**+ NDM**<br>**No difference**		**0/7**<br>**0/7**<br>**7/7**	**0/7**<br>**2/7**<br>**5/7**	**0/3**<br>**3/6**<br>**3/6**	**0/7**<br>**1/7**<br>**6/7**	**0/1**<br>**0/1**<br>**1/1**	
**Analysis good controlled DM**							
IX Dag et al. 2009	DM +	0	0	0	0	□	NDM
X Kardesler et al. 2009	DM +	0	0	0	0	□	NDM
XVI López et al. 2013	DM +	0	0	0	0	□	NDM
XVIII Kaur et al. 2015	DM +	0	0	0	0	□	NDM
XIX Pannicker et al. 2015	DM +	0	0	0	0	□	NDM
XX Dogan et al. 2016	DM +	0	0	0	0	□	NDM
XXI Mishra et al. 2016	DM +	□	-	-	0	□	NDM
XXII Abreu et al. 2015	DM +	0	0	0	0	□	NDM
XXIV Sundaram et al. 2017	DM +	?	?	?	?	□	NDM
XXV Gayathri et al. 2019	DM +	0	0	-	□	□	NDM

XXVI Ahuja et al. 2019	DM +	-	-	-	-	□	NDM
XXVII Pragada et al. 2019	DM +	0	0	0	0	□	NDM
XXIX Mirnic et al. 2021	DM +	0	-	0	0	□	NDM
**Total**<br>**+ DM**<br>**+ NDM**<br>**No difference**		**0/11**<br>**1/11**<br>**10/11**	**0/12**<br>**3/12**<br>**9/12**	**0/12**<br>**2/12**<br>**9/12**	**0/11**<br>**1/11**<br>**10/11**	**NA**	

A positive sign (+) implies that the DM group had significant more reduction of parameters or CAL gain than the NDM group. With a negative sign (-) the DM group had significant less reduction or gain than the NDM group. No differences between the two groups were assigned with the number zero (0).

Abbreviations: □, no data available due to lack of evaluation, + significant higher (percentage) of reduction for DM, - significant higher (percentage) of reduction for NDM (for GI/CAL higher numbers), 0 no difference between DM group and non-DM, ? no inter-group evaluation between DM and NDM, *CAL* clinical attachment level, *PPD* periodontal pocket depth, *BLI* bone loss index, *BOP* bleeding on probing, *GI* gingival index*, DMT I* diabetic mellitus type I, *DMT II* diabetic mellitus type II, *DMT B* diabetic mellitus type I or II or both, PI* Silness & Löe 1964, PI** O'Leary et al. 1972, PI recorded at 6 sites (yes/no), VPI visible plaque index following Ainamo

Online Appendix S6

Mean (SD) scores for the different intervention groups with various indices and their modifications. Within-group analyses are presented.

S6.1 probing pocket depth (PPD), S6.2: clinical attachment level (CAL), S6.3: bleeding score (BS), S6.4: gingival indices, S6.5: plaque indices, S6.6: gingival recession (REC)

**Online Appendix S6.1**						
**PPD**	**Group**	**Baseline Mean (SD)**		**End Mean (SD)**	**Difference Mean (SD)**	**Significant within groups**
I Tervonen et al. 1991	DM	4-5mm	9.2 (13.3) %	3.8 (7.4) %	-5.4 (8.1) %	?
	Control	4-5mm	6.2 (9.7) %	2.0 (3.4) %	-4.1 (8.1) %	?
	DM	≥6mm	0.8 (2.4) %	0.6 (3.4) %	-0.1 (3.8) %	?
	Control	≥6mm	0.9 (3.5) %	0.3 (1.3) %	-0.6 (2.3) %	?
II Tervonen et al. 1997	DM	≥4mm	6.5 (7.6) %	?	?	?
	Control	≥4mm	6.9 (13.6) %	?	?	?
III Christgau et al. 1998	DM	3.6 (1.6) mm ♦		3.2 (1.2) mm ♦	-0.4 (1.1) mm ♦	Yes
	Control	3.8 (1.7) mm ♦		3.3 (1.3) mm ♦	-0.5 (1.0) mm ♦	Yes
IV Sonoki et al. 2006	DM	3.30 (0.63) mm ◊		2.58 (0.38) mm ◊	-0.76 (0.56) mm ♦	Yes
	Control	2.93 (0.81) mm ◊		2.16 (0.42) mm ◊	-0.72 (0.46) mm ♦	Yes
V Navarro-Sanchez et al. 2007	DM	4.1 (0.3) mm		3.0 (0.4) mm	-1.1 (0.2) mm	Yes
	Control	3.7 (0.5) mm		2.6 (0.2) mm	-1.1 (0.3) mm	Yes
	DM	4-6mm sites	64.9 (22.6) %	17.7 (11.0) %	-47.2 (22.0) %	Yes
	Control		53.1 (20.9) %	7.1 (5.0) %	-46.0 (18.1) %	Yes
	DM	≥7mm sites	4.1 (3.5) %	1.7 (2.5) %	-2.4 (3.1) %	Yes
	Control		1.2 (1.5) %	0.3 (0.6) %	-0.9 (1.2) %	Yes
VI Da Cruz et al.2008	DM	5.72 (1.13) mm		5.00 (1.29) mm	-0.71 (0.19) mm	Yes
	Control	4.79 (0.9) mm		3.97 (0.82) mm	-0.82 (0.13) mm	Yes
VII Correa et al. 2008	DM	4.2 (1.0) mm ♦		2.8 (0.4) mm ♦	1.5 (0.7) mm ♦	Yes ♦
	Control	3.6 (0.6) mm ♦		2.4 (0.3) mm ♦	1.2 (0.6) mm ♦	Yes ♦
	DM	≤ 3mm	49.4 (22.8) % ♦	83.7 (11.7) % ♦	+34.3 (15.6) % ♦	Yes ♦
	Control		60.4 (14.1) % ♦	90.4 (6.6) % ♦	+30.0 (13.2) % ♦	Yes ♦

	DM	4-6mm	39.3 (14.8) % ♦	15.3 (10.7) % ♦	-24.0 (9.7) % ♦	Yes ♦
	Control		31.6 (9.8) % ♦	9.4 (6.5) % ♦	-22.3 (10.3) % ♦	Yes ♦
	DM	≥7mm	13.0 (12.9) % ♦	1.0 (1.7) % ♦	-12.0 (12.8) % ♦	Yes ♦
	Control		8.0 (6.7) % ♦	0.3 (0.9) % ♦	-7.7 (6.8) % ♦	Yes ♦
VIII Gonçalves et al. 2008	DM (-)	4.18 (0.90) mm ♦		2.76 (0.41) mm ♦	-1.42 (0.61) mm ♦	Yes ♦
	Control	3.62 (0.56) mm ♦		2.44 (0.30) mm ♦	-1.17 (0.54) mm ♦	Yes ♦
IX Dağ et al.2009	DM (-)	2.84 (0.65) mm		2.37 (0.59) mm	-0.47 mm ◊	Yes
	DM (+)	2.67 (0.45) mm		2.30 (0.43) mm	-0.37 mm ◊	Yes
	Control	2.61 (0.38) mm		2.36 (0.68) mm	-0.25 mm ◊	Yes
X Kardesler et al. 2009	DM (-)	4.1 (1.0) mm		3.1 (0.6) mm	-1.06 (0.54) mm ♦	Yes
	DM (+)	3.7 (0.4) mm		2.8 (0.4) mm	-0.93 (0.37) mm ♦	Yes
	Control	3.9 (0.6) mm		2.7 (0.3) mm	-1.17 (0.51) mm ♦	Yes
XI Kudva et al. 2010	DM	3.31 (1.54)		1.69 (1.06)	-1.62 (0.79)	Yes
	Control	3.63 (1.61)		1.78 (1.06)	-1.85 (0.81)	Yes
XII Hungund et al. 2012	DM	3.16 (0.65)		2.67 (0.43)	-0.49 (0.18)	Yes
	Control	3.04 (0.64)		2.03 (0.45)	-1.01 (0.19)	Yes
XIII Cirano et al. 2012	DM (-)	5.3 (0.3) mm		3.0 (0.5) mm	-2.3 mm ◊	Yes
	Control	5.9 (0.2) mm		3.2 (0.5) mm	-2.7 mm ◊	Yes
	DM	≤ 4mm	3.6 (0.2) mm	2.5 (0.3) mm	-1.1 mm ◊	Yes
	Control		3.9 (0.2) mm	2.6 (0.4) mm	- 1.3 mm ◊	Yes
	DM	5-6mm	5.3 (0.2) mm	2.9 (0.4) mm	-2.4 mm ◊	Yes
	Control		5.5 (0.2) mm	3.0 (0.5) mm	-2.5 mm ◊	Yes
	DM	≥7mm	7.9 (0.6) mm	4.0 (1.2) mm	-3.9 mm ◊	Yes
	Control		8.2 (1.0) mm	4.2 (1.3) mm	-4.0 mm ◊	Yes
XIV Camargo et al. 2013	DM	3.46 (0.55) mm		2.62 (0.6) mm	-0.9 (0.37) mm	No
	Control	2.81 (0.55) mm		2.49 (0.48) mm	-0.33 (0.31) mm	Yes
XV Buzinin et al. 2014	DM	< 4mm	79.69 (15.31) %	97.76 (1.87) %	+18.07% ◊	Yes
	Control		74.96 (11.49) %	95.7 (4.13) %	+20.74% ◊	Yes
	DM	4-6 mm	19.68 (13.80) %	2.26 (1.82) %	-17.42% ◊	Yes
	Control		27.35 (13.28) %	4.10 (3.62) %	-23.25% ◊	Yes

	DM	>6mm	0.68 (1.58) %	0.00 (0.00) %	-0.68% ◊	No
	Control		1.78 (2.80) %	0.20 (0.69) %	-1.58% ◊	Yes
XVI López et al. 2014	DM		3.3 (0.5) mm	2.79 (0.58) ♦	-0.51 (0.08) ♦	Yes
	Control		3.1 (0.5) mm	2.75 (0.57) ♦	-0.35 (0.07) ♦	Yes
	DM	≥4mm	43.7 (20.3) %	25.3 (21.8) % ♦	-18.4 (1.05) % ♦	?
	Control		40.4 (30.9) %	22.0 (16.0) % ♦	-18.4 (14.9) % ♦	?
	DM	≥6mm	3.38 (1.78) %	2.87 (6.08) %	-0.96 (3.60) %	?
	DM (-)		6.34 (11.0) % ♦	4.66 (8.31) % ♦	0.0 (1.68) % ♦	No
	DM(+)		1.71 (1.8) % ♦	1.39 (1.40) % ♦	-0.32 (0.40) % ♦	?
	Control		3.37 (3.58) % ♦	2.14 (2.86) % ♦	-1.22 (3.42) % ♦	Yes
	DM (-)		3.12 (0.65) mm ♦	2.79 (0.69) mm ♦	-0.33 (0.04) mm ♦	?
	DM (+)		3.17 (0.56) mm ♦	2.86 (0.56) mm ♦	-0.31 (0.0) mm ♦	?
XVII Kara et al. 2015	DM		4.13 (0.44) mm	2.34 (0.26) mm	-1.79 mm ◊	Yes
	Control		4.12 (0.43) mm	2.31 (0.29) mm	-1.81 mm ◊	Yes
XVIII Kaur et al. 2015	DM		2.96 (0.46) mm	2.15 (0.42) mm	-0.81 (0.28) mm	Yes
	DM (-)		3.08 (0.45) mm	2.29 (0.37) mm	-0.79 (0.26) mm	Yes
	DM (+)		2.82 (0.44) mm	1.98 (0.43) mm	-0.84 (0.31) mm	Yes
	Control		2.77 (0.58) mm	1.91 (0.44) mm	-0.86 (0.29) mm	Yes
	DM (-)	<3mm	33.02 (5.26) %	52.19 (8.64) %	+19.16 (8.11) %	Yes
	DM (+)		42.67 (9.06) %	66.87 (11.14) %	+24.20 (10.30) %	Yes
	Control		39.69 (9.23) %	62.94 (9.70) %	+23.25 (8.4) %	Yes
	DM (-)	4-6mm	53.92 (5.05) %	40.76 (7.72) %	-13.17 (6.55) %	Yes
	DM (+)		46.06 (9.54) %	28.49 (10.23) %	-17.56 (8.99) %	Yes
	Control		49.49 (8.00) %	34.15 (9.48) %	-15.34 (6.71) %	Yes
	DM (-)	≥7mm	13.05 (3.78) %	7.05 (3.15) %	-5.99 (2.14) %	Yes
	DM (+)		11.27 (2.97) %	4.63 (1.97) %	-6.64 (3.25)%	Yes
	Control		10.82 (4.09) %	2.91 (1.30) %	-7.91 (3.64) %	Yes
XIX Pannicker et al. 2015	DM (+)		7.94 (1.57) mm	5.74 (1.24) mm	-2.2 mm ◊	Yes
	Control		7.45 (1.68) mm	5.62 (1.33) mm	- 1.83 mm ◊	Yes
XX Dogan et al.	DM (+)		4.11 (0.34) mm	2.67 (0.26) mm	-1.44 mm ◊	?

2016	Control	4.09 (0.37) mm	2.61 (0.30) mm	-.1.48 mm ◊	?
XXI Mishra et al. 2016	DM (+)	7.43 (0.94) mm ♦	7.00 (0.78) mm ♦	-0.43 (0.51) mm ♦	?
	Control	6.64 (0.50) mm ♦	6.36 (0.50) mm ♦	-0.29 (0.47) mm ♦	?
XXII Abreu et al. 2015	DM (+)	3.16 (0.39) mm ♦	2.54 (0.74) mm ♦	-1.12 (0.07) mm ♦	Yes
	Control	2.97 (0.63) mm ♦	2.20 (0.79) mm ♦	-0.74 (0.74) mm ♦	Yes
XXIII Perruzo Lopes et al. 2017	DM I	1.83 (0.05) mm	1.36 (0.02) mm	-0.47 (0.02) mm	Yes
	DM II	2.33 (0.06) mm	1.83 (0.06) mm	-0.50 (0.03) mm	Yes
	Control	2.12 (0.06) mm	1.76 (0.04) mm	-0.48 (0.04) mm	Yes
XXIV Sundaram et al. 2017	DM (-)	4.80 (0.57) mm	1.99 (0.71) mm	-2.81 mm ◊	Yes
	DM (+)	4.04 (1.02) mm	1.88 (0.54) mm	-2.16 mm ◊	Yes
	Control	3.84 (1.33) mm	1.87 (1.06) mm	-1.97 mm ◊	Yes
XXV Gayathri et al. 2019	DM (+)	3.57 (0.40) mm	3.44 (0.42) mm	-0.13 ◊ mm	No
	Control	3.29 (0.42) mm	3.16 (0.46) mm	-0.13 ◊ mm	No
XXVI Ahuja et al. 2019	DM (+)	5.60 (0.38) mm	3.07 (0.60) mm	-2.53 (0.24) mm	Yes
	Control	4.98 (0.49) mm	2.42 (0.72) mm	-2.57 (0.26) mm	Yes
XXVII Pragada et al. 2019	DM (+)	7.3 (1.69) mm	5.26 (1.18) mm	-2.04 ◊ mm	Yes
	Control	6.83 (1.70) mm	5.06 (1.36) mm	-1.77 ◊ mm	Yes
XXVIII Almeida et al. 2019	DM	2.84 (0.67) mm	2.40 (0.58) mm	-0.44 (0.28) mm	?
	Control	2.79 (0.74) mm	2.32 (0.43) mm	-0.47 (0.60) mm	?
XXIX Mirnic et al. 2022	DM (-)	2.11 (0.47) mm	2.03 (0.35) mm	-0.08 (0.27) mm	Yes
	DM (+)	2.09 (0.51) mm	1.98 (0.53) mm	-0.11 (0.16) mm	Yes
	Control	2.38 (0.60) mm	2.05 (0.52) mm	-0.34 (0.23) mm	No
XXX Gomathi et al. 2023	DM (+)	5.60 (0.54) mm	2.97 (0.20) mm	2.63 mm ◊	Yes
	Control	4.72 (0.40) mm	3.0 (0.16) mm	1.72 mm ◊	Yes

Abbreviations: ?: Not specified/unclear, ♦: At request provided by the original author, ◊: calculated by the review authors, +: well controlled, -: poorly controlled, DM I: diabetes mellitus type I, DM II: diabetes mellitus type II, DM: diabetes mellitus.

Online Appendix S6.1						
PPD	Group	Baseline Mean (SD)		End Mean (SD)	Difference Mean (SD)	Significant within groups
I Tervonen et al. 1991	DM	≥6mm	0.8 (2.4) %	0.6 (3.4) %	-0.1 (3.8) %	?
	Control		0.9 (3.5) %	0.3 (1.3) %	-0.6 (2.3) %	?
V Navarro-Sanchez et al. 2007	DM	≥7mm	4.1 (3.5) %	1.7 (2.5) %	-2.4 (3.1) %	Yes
	Control		1.2 (1.5) %	0.3 (0.6) %	-0.9 (1.2) %	Yes
VII Correa et al. 2008	DM	≥7mm	13.0 (12.9) % ♦	1.0 (1.7) % ♦	-12.0 (12.8) % ♦	Yes ♦
	Control		8.0 (6.7) % ♦	0.3 (0.9) % ♦	-7.7 (6.8) % ♦	Yes ♦
XIII Cirano et al. 2012	DM (-)	≥7mm	7.9 (0.6) mm	4.0 (1.2) mm	-3.9 mm ◊	Yes
	Control		8.2 (1.0) mm	4.2 (1.3) mm	-4.0 mm ◊	Yes
XV Buzinin et al. 2014	DM	>6mm	0.68 (1.58) mm	0.00 (0.00) mm	-0.68 mm ◊	No
	Control		1.78 (2.80) mm	0.20 (0.69) mm	-0.20 mm ◊	No
XVI López et al. 2014	DM	≥6mm	3.83 (3.5) % ♦	2.87 (6.08) % ♦	-0.96 (3.60) % ♦	?
	DM (-)		6.34 (11.0) % ♦	4.66 (8.31) % ♦	0.0 (1.68) % ♦	No
	DM (+)		1.71 (1.8) % ♦	1.39 (1.40) % ♦	-0.32 (0.40) % ♦	?
	Control		3.37 (3.58) % ♦	2.14 (2.86) % ♦	-1.22 (3.42) % ♦	Yes
XVIII Kaur et al. 2015	DM	≥7mm	12.24 (3.52) %	5.94 (2.91) %	-6.29 (2.70) %	Yes
	DM (-)		13.05 (3.78) %	7.05 (3.15) %	-5.99 (2.14) %	Yes
	DM (+)		11.27 (2.97) %	4.63 (1.97) %	-6.64 (3.25)%	Yes
	Control		10.82 (4.09) %	2.91 (1.30) %	7.91 (3.64) %	Yes

Abbreviations: ?: not specified/unclear, ♦: At request provided by the original author, ◊: calculated by the review authors, +: well controlled, -: poorly controlled, DM I: diabetes mellitus type I, DM II: diabetes mellitus type II, DM: diabetes mellitus

Online Appendix S6.2

CAL	Group	Baseline Mean (SD)		End Mean (SD)	Difference Mean (SD)	Significant within groups
II Tervonen et al. 1997	DM	≥2mm	11.4 (13.1)%	?	?	?
	Control	≥2mm	7.9 (8.8)%	?	?	?
III Christgau et al. 1998	DM	4.3 (2.0) mm ♦		4.1 (1.8) mm ♦	-0.2 (1.1) mm ♦	No
	Control	4.3 (1.9) mm ♦		4.1 (1.8) mm ♦	-0.2 (1.1) mm ♦	No
V Navarro-Sanchez et al. 2007	DM	5.7 (1.9) mm		5.2 (2.0) mm	-0.5 (0.2) mm	Yes
	Control	4.9 (0.8) mm		4.5 (0.8) mm	-0.4 (0.2) mm	Yes
VI Da Cruz et al.2008	DM	4.49 (0.7) mm		3.28 (0.54) mm	-1.21 (0.24) mm	Yes
	Control	4.03 (0.37) mm		2.90 (0.26) mm	-1.12 (0.15) mm	Yes
VII Correa et al. 2008	DM	5.4 (1.3) mm ♦		4.6 (1.1) mm ♦	-0.8 (0.6) mm ♦	Yes ♦
	Control	4.5 (1.1) mm ♦		4.0 (0.8) mm ♦	-0.6 (0.6) mm ♦	No ♦
	DM	≤ 3mm	24.6 (18.4) %♦	31.4 (24.3) % ♦	-6.8 (10.4) % ♦	Yes ♦
	Control		40.1 (19.2) % ♦	42.5 (18.3) % ♦	-2.4 (8.2) % ♦	Yes ♦
	DM	4-6mm	46.8 (10.3) % ♦	54.7 (16.4) % ♦	-8.0 (14.7) % ♦	Yes ♦
	Control		40.9 (11.2) % ♦	50.3 (12.8) % ♦	-9.5 (9.2) % ♦	Yes ♦
	DM	≥7mm	28.9 (20.5) % ♦	14.2 (14.5) % ♦	-14.7 (10.4) % ♦	Yes ♦
	Control		19.1 (13.7) % ♦	7.2 (9.5) % ♦	-11.9 (9.8) % ♦	Yes ♦
VIII Gonçalves et al. 2008	DM (-)	4.97 (1.45) mm ♦		4.34 (1.29) mm ♦	-0.63 (0.43) mm ♦	Yes
	Control	4.25 (1.01) mm ♦		3.97 (0.83) mm ♦	-0.46 (0.40) mm ♦	Yes
IX Dağ et al. 2009	DM (-)	4.3 (0.97) mm		3.04 (0.81) mm	-1.26 mm ◊	Yes
	DM (+)	4.25 (0.82) mm		3.03 (0.79) mm	-1.22 mm ◊	Yes
	Control	4.31 (0.59) mm		2.91 (0.56) mm	-1.40 mm ◊	Yes
X Kardesler et al. 2009	DM (-)	4.4 (1.1) mm		4.2 (0.9) mm	-0.21 (0.52) mm ♦	No
	DM (+)	4.3 (0.9) mm		3.9 (0.9) mm	-0.30 (0.53) mm ♦	No
	Control	4.3 (0.7) mm		3.9 (0.7) mm	-0.36 (0.62) mm ♦	No
XIII Cirano et al. 2012	DM (-)	6.0 (0.7) mm		3.9 (0.8) mm	-2.1 mm ◊	Yes
	Control	6.3 (0.7) mm		4.2 (0.9) mm	-2.1 mm ◊	Yes
	DM	≤ 4mm	4.0 (0.5) mm	3.2 (0.7) mm	-0.8 mm ◊	Yes
	Control		4.4 (0.4) mm	3.2 (0.6) mm	-1.2 mm ◊	Yes

	DM	5-6mm	6.0 (0.7) mm	3.9 (0.9) mm	-2.1 mm ◊	Yes
	Control		6.0 (0.9) mm	4.0 (1.1) mm	-2.0 mm ◊	Yes
	DM	≥7mm	8.7 (0.9) mm	5.5 (1.3) mm	-3.2 mm ◊	Yes
	Control		8.8 (1.3) mm	5.6 (1.6) mm	-3.2 mm ◊	Yes
XIV Camargo et al. 2013	DM	3.71 (0.62) mm		2.85 (0.66) mm	-0.85 (0.28) mm	Yes
	Control	3.30 (0.90) mm		2.92 (0.48) mm	-0.37 (0.63) mm	Yes
XV Buzinin et al. 2014	DM	<4mm	7.53 (9.98) %	12.34 (14.93) %	+4.81 % ◊	Yes
	Control		9.24 (5.5) %	19.56 (9.94) %	+10.32 % ◊	Yes
	DM	4-6 mm	26.90 (18.85) %	7.46 (9.22) %	-19.44 % ◊	Yes
	Control		34.38 (10.69) %	11.97 (10.02) %	- 22.41 % ◊	Yes
	DM	>6mm	1.85 (3.26) %	0.54 (1.67) %	-1.31 % ◊	Yes
	Control		3.12 (5.77) %	1.06 (3.29) %	-2.06 % ◊	Yes
XVI López et al. 2014	DM	3.6 (0.8) mm		3.57 (0.99) ♦	-0.03 (0.19) ♦	No
	DM (-)	3.73 (0.9) mm ♦		3.66 (0.85) ♦	0.07 (0.05) ♦	No
	DM (+)	3.59 (0.91) mm ♦		3.44 (0.86) ♦	-0.15 (0.05) ♦	No
	Control	3.6 (0.8) mm		3.43 (0.74) ♦	-0.17 (0.06) ♦	No
XVII Kara et al. 2015	DM	4.78 (0.58) mm		3.06 (0.73) mm	-1.72 mm ◊	Yes
	Control	4.49 (0.41) mm		2.75 (0.50) mm	-1.74 mm ◊	Yes
XVIII Kaur et al. 2015	DM	3.46 (0.53) mm		2.75 (0.62) mm	-0.71 (0.36) mm	Yes
	DM (-)	3.59 (0.52) mm		2.90 (0.72) mm	-0.71 (0.38) mm	Yes
	DM (+)	3.30 (0.52) mm		2.58 (0.43) mm	-0.72 (0.34) mm	Yes
	Control	3.14 (0.53) mm		2.41 (0.55) mm	-0.73 (0.31) mm	Yes
XIX Pannicker et al. 2015	DM (+)	11.97 (2.43) mm		9.85 (2.39) mm	-2.12 mm ◊	Yes
	Control	9.82 (1.31) mm		7.48 (1.22) mm	-2.34 mm ◊	Yes
XX Dogan et al. 2016	DM (+)	4.31 (0.36) mm		3.09 (0.43) mm	-1.22 mm ◊	?
	Control	4.25 (0.35) mm		2.88 (0.38) mm	-1.37 mm ◊	?
XXI Mishra et al. 2016	DM (+)	2.51 (0.27) mm ♦		1.63 (0.24) mm ♦	- 0.88 (0.13) mm ♦	?
	Control	2.08 (0.37) mm ♦		1.41 (0.27) mm ♦	-0.66 (0.31) mm ♦	?
XXII Abreu et al. 2015	DM (+)	4.15 (0.90) mm ♦		3.58 (0.92) mm ♦	-1.28 (1.63) mm ♦	Yes
	Control	3.77 (0.71) mm ♦		3.10 (1.12) mm ♦	-0.64 (1.09) mm ♦	No

XXIII Perruzo Lopes et al. 2017	DM I	2.62 (0.09) mm	1.56 (0.04) mm	-1.06 (0.04) mm	Yes
	DM II	3.10 (0.12) mm	2.44 (0.12) mm	-0.66 (0.08) mm	Yes
	Control	2.49 (0.06) mm	1.82 (0.04) mm	-0.67 (0.04) mm	Yes
XXIV Sundaram et al. 2017	DM (-)	4.03 (0.47) mm	2.73 (1.12) mm	-1.3 mm ◊	Yes
	DM (+)	4.36 (0.67) mm	3.06 (1.05) mm	-1.3 mm ◊	Yes
	Control	4.13 (0.79) mm	2.97 (0.99) mm	-1.16 mm ◊	No
XXV Gayathri et al. 2019	DM (+)	3.65 (0.45) mm	3.61 (0.48) mm	-0.04 mm ◊	No
	Control	3.60 (0.42) mm	3.47 (0.58) mm	-0.13 mm ◊	No
XXVI Ahuja et al. 2019	DM (+)	6.01 (0.38) mm	3.75 (0.86) mm	-2.25 (0.67) mm	Yes
	Control	5.35 (0.55) mm	2.81 (0.78) mm	-2.55 (0.25) mm	Yes
XXVII Pragada et al. 2019	DM (+)	10.07 (1.63) mm	7.53 (1.04) mm	-2.54 ◊ mm	Yes
	Control	9.67 (1.51) mm	7.10 (1.21) mm	-2.57 ◊ mm	Yes
XXVIII Almeida et al. 2019	DM	3.83 (2.02) mm	3.52 (1.91) mm	-0.30 (0.79) mm	Yes
	Control	2.36 (1.57) mm	2.11 (1.20) mm	-0.25 (0.74) mm	Yes
XXIX Mirnic et al. 2022	DM (-)	2.62 (1.36) mm	2.39 (1.27) mm	-0.23 (0.27) mm	No
	DM (+)	2.81 (0.98) mm	2.57 (1.01) mm	-0.24 (0.24) mm	No
	Control	2.32 (1.39) mm	1.98 (1.31) mm	-0.34 (0.3) mm	No
XXX Gomathi et al. 2023	DM (+)	4.36 (0.82) mm	3.03 (0.19) mm	-1.33 mm ◊	Yes
	Control	4.38 (0.65) mm	3.05 (0.15) mm	-1.33 mm ◊	Yes

Abbreviations: ?: not specified/unclear, ♦: At request provided by the original author, ◊: calculated by the review authors, +: well controlled, -: poorly controlled, DM I: diabetes mellitus type I, DM II: diabetes mellitus type II, DM: diabetes mellitus

Online Appendix S6.3						
**Bleeding score** Overall	**Index**	**Group**	**Baseline Mean % (SD)**	**End Mean % (SD)**	**Difference Mean % (SD)**	**Significant within groups**
I Tervonen et al. 1991	BOP	DM	52.6 (32.2)	24.9 (22.0)	-27.7 (24.7)	?
		Control	60.1 (26.3)	29.9 (26.2)	-30.3 (24.3)	?
II Tervonen et al. 1997	BOP	DM	53.7 (18.3)	?	?	?
		Control	55.0 (18.6)	?	?	?
III Christgau et al. 1998	BOP	DM	64.9 (22.7) ♦	29.5 (17.5) ♦	-35.4 (20.3) ♦	Yes
		Control	72.1 (16.5)♦	24.8 (10.1) ♦	-47.3 (16.4) ♦	Yes
	PBI	DM	48.7 (20.3) ♦	23.2 (14.6) ♦	-25.5 (19.2) ♦	?
	Saxer & Mühlemann 1975	Control	40.1 (19.9) ♦	17.8 (9.8) ♦	-22.3 (13.8) ♦	?
IV Sonoki et al. 2006	BOP	DM	46.0 (21.9) ♦	17.3 (17.5) ♦	-28.7 (21.4) ♦	Yes
		Control	31.5 (17.8) ♦	12.8 (12.2) ♦	-18.6 (11.3) ♦	Yes
V Navarro-Sanchez et al. 2007	BOP	DM	91.0 (14.8)	11.4 (9.7)	-79.6 (16.7)	Yes
		Control	83.9 (17.2)	12.1 (5.9)	-71.8 (16.4)	Yes
V Da Cruz et al. 2008	GBI	DM	81.97 (25.11)	10.72 (3.97)	-71.85 (21.23) ♦	Yes
	Ainamo & Bay (1975)	Control	82.55 (23.81)	9.30 (2.95)	-72.26 (23.12) ♦	Yes
VII Correa et al. 2008	BOP	DM	90.4 (10.5) ♦	28.9 (16.5) ♦	-68.5 (15.9) ♦	Yes ♦
		Control	75.6 (15.2) ♦	16.3 (10.3) ♦	59.3 (15.7) ♦	Yes ♦
	GBI	DM	46.8 (22.0) ♦	11.4 (10.8) ♦	73.5 (19.5) ♦	Yes ♦
	Ainamo & Bay (1975)	Control	38.1 (14.9) ♦	3.7 (3.0) ♦	34.5 (14.6) ♦	Yes ♦
VIII Gonçalves et al. 2008	BOP	DM (-)	90.9 (9.9) ♦	28.2 (15.9) ♦	-69.21 (15.64) ♦	?
		Control	76.1 (16.1) ♦	15.8 (10.7) ♦	-78.67 (13.97) ♦	?
	GBI	DM (-)	47.3 (23.2) ♦	10.8 (10.4) ♦	-74.63 (19.02) ♦	?
	Ainamo & Bay (1975)	Control	38.4 (16.5) ♦	2.9 (2.3) ♦	-90.81 (9.50) ♦	?
IX Dag et al. 2009	GBI	DM (-)	33.0 (18.0)	4.0 (2.0)	-29.0 ◊	Yes
		DM (+)	28.0 (12.0)	4.0 (1.0)	-24.0 ◊	Yes
	Ainamo & Bay (1975)	Control	37.0 (18.0)	3.0 (2.0)	-34.0 ◊	Yes
X Kardesler et al. 2009	BOP	DM (-)	86.0 (12.3)	40.6 (8.2)	-45.40 (11.98) ♦	No
		DM (+)	81.5 (7.9)	25.4 (6.9)	-56.10 (9.80) ♦	No
		Control	78.8 (16.5)	28.1 (9.2)	-50.70 (18.24) ♦	No

XII Hungund et al. 2012	GBI *Ainamo & Bay (1975)*	DM	85.92 (13.30)	54.66 (12.11)	-31.25 (1.19)	Yes
		Control	86.66 (13.54)	57.24 (8.24)	-29.41 (4.72)	Yes
XIII Cirano et al. 2012	SBI *Mühlemann & Son (1975)*	DM (-)	67.5 (16.4)	17.6 (10.5)	-49.9 ◊	No
		Control	65.5 (17.1)	19.1 (11.4)	-46.4 ◊	No
XIV Camargo et al.2013	GBI *Ainamo & Bay (1975)*	DM	61.11 (15.91)	13.51 (3.9)	-47.6 (15.53) ♦	Yes
		Control	39.99 (20.31)	22.44 (14.87)	-16.95 (19.27) ♦	Yes
XV Buzinin et al. 2014	GBI *Ainamo & Bay (1975)*	DM	36.7 (22.2)	12.15 (7.68)	-24.55 ◊	Yes
		Control	34.04 (20.56)	11.76 (9.69)	-22.28 ◊	Yes
XVI López et al. 2014	BOP	DM	57.4 (15.1)	32.5 (13.4) ♦	-24.9 (1.7) ♦	Yes
		DM (-)	44.4 (15.3)	33.0 (11.98) ♦	-16.0 (1.98) ♦	?
		DM (+)	50.0 (9.6)	34.38 (8.14) ♦	-14.5 (2.0) ♦	?
		Control	47.2 (12.8)	33.2 (10.0) ♦	-14.0 (2.8) ♦	Yes
XVII Kara et al. 2015	BOP	DM	75.73 (12.18)	24.24 (11.58)	-51.49 ◊	Yes
		Control	73 (15.65)	15.43 (5.72)	-57.57 ◊	Yes
XVIII Kaur et al. 2015	BOP	DM	73.68 (14.63)	38.96 (11.62)	-34.71 (14.78)	Yes
		DM (-)	75.15 (14.15)	45.03 (9.70)	-30.11 (12.54)	Yes
		DM (+)	71.95 (15.33)	31.85 (9.56)	-40.05 (15.63)	Yes
		Control	70.58 (16.62)	29.10 (8.65)	-41.48 (15.27)	Yes
XX Dogan et al. 2016	GBI *Ainamo & Bay (1975)*	DM (+)	70.29 (8.35)	9.24(1.89)	-61.05 ◊	?
		Control	75.83 (10.07)	8.91(1.68)	-66.92 ◊	?
XXII Abreu et al. 2015	BOP	DM	38.24 (19.29) ♦	13.31 (15.12) ♦	-27.58 (21.90) ♦	Yes
		Control	63.00 (23.87) ♦	15.11 (20.27) ♦	-46.30 (23.62) ♦	Yes
XXIII Perruzo Lopes et al. 2017	BOP	DM I	37.77 (3.38)	5.67 (0.75)	-32.10 (0.51)	Yes
		DM II	51.36 (3.22)	12.73 (1.20)	-38.63 (2.00)	Yes
		Control	63.05 (2.78)	8.23 (0.35)	-54.82 (0.51)	Yes
XXIV Sundaram et al. 2017	SBI *Mühlemann & Son (1975)*	DM (-)	2.11 (0.59)*	0.79 (0.29)*	-1.32* ◊	Yes
		DM (+)	2.16 (0.53)*	1.02 (0.31)*	-1.14* ◊	Yes
		Control	2.16 (0.68)*	0.92 (0.38)*	-1.24* ◊	Yes
XXVIII Almeida et al. 2019	BOP	DM	45.5 (22.4)	29.1 (15.6)	-16.5 (16.2) ♦	No
		Control	40.9 (18.9)	22.4 (13.3)	-18.5 (19.8) ♦	No

Abbreviations: ?: not specified/unclear, ♦: At request provided by the original author, ◊: calculated by the review authors, +: well controlled, -: poorly controlled, DM I: diabetes mellitus type I, DM II: diabetes mellitus type II, DM: diabetes mellitus, *scores in stead of percentages

Online Appendix S6.3					
**Bleeding on probing (BOP)**	**Group**	**Baseline Mean % (SD)**	**End Mean % (SD)**	**Difference Mean % (SD)**	**Significant within groups**
I Tervonen et al. 1991	DM	52.6 (32.2)	24.9 (22.0)	-27.7 (24.7)	?
	Control	60.1 (26.3)	29.9 (26.2)	-30.3 (24.3)	?
II Tervonen et al. 1997	DM	53.7 (18.3)	?	?	?
	Control	55.0 (18.6)	?	?	?
III Christgau et al. 1998	DM	64.9 (22.7) ♦	29.5 (17.5) ♦	-35.4 (20.3) ♦	Yes
	Control	72.1 (16.5) ♦	24.8 (10.1) ♦	-47.3 (16.4) ♦	Yes
IV Sonoki et al. 2006	DM	46.0 (21.9) ♦	17.3 (17.5) ♦	-28.7 (21.4) ♦	Yes
	Control	31.5 (17.8) ♦	12.8 (12.2) ♦	-18.6 (11.3) ♦	Yes
V Navarro-Sanchez et al. 2007	DM	91.0 (14.8)	11.4 (9.7)	-79.6 (16.7)	Yes
	Control	83.9 (17.2)	12.1 (5.9)	-71.8 (16.4)	Yes
VII Correa et al. 2008	DM	90.4 (10.5) ♦	28.9 (16.5) ♦	-68.5 (15.9) ♦	Yes ♦
	Control	75.6 (15.2) ♦	16.3 (10.3) ♦	-59.3 (15.7) ♦	Yes ♦
VIII Gonçalves et al. 2008	DM (-)	90.9 (9.9) ♦	28.2 (15.9) ♦	-69.21 (15.64) ♦	?
	Control	76.1 (16.1) ♦	15.8 (10.7) ♦	-78.67 (13.97) ♦	?
X Kardesler et al. 2009	DM (-)	86.0 (12.3)	40.6 (8.2)	-45.40 (11.98) ♦	No
	DM (+)	81.5 (7.9)	25.4 (6.9)	-56.10 (9.80) ♦	No
	Control	78.8 (16.5)	28.1 (9.2)	-50.70 (18.24) ♦	No
XVI López et al. 2014	DM	57.4 (15.1)	32.5 (13.4) ♦	-24.9 (1.7) ♦	Yes
	DM (-)	44.4 (15.3)	33.0 (11.98) ♦	-16.0 (1.98) ♦	?
	DM (+)	50.0 (9.6)	34.38 (8.14) ♦	-14.5 (2.0) ♦	?
	Control	47.2 (12.8)	33.2 (10.0) % ♦	-14.0 (2.8) ♦	Yes
XVII Kara et al. 2015	DM	75.73 (12.18)	24.24 (11.58)	-51.49 ◊	Yes
	Control	73 (15.65)	15.43 (5.72)	-57.57 ◊	Yes
XVIII Kaur et al. 2015	DM	73.68 (14.63)	38.96 (11.62)	-34.71 (14.78)	Yes
	DM (-)	75.15 (14.15)	45.03 (9.70)	-30.11 (12.54)	Yes
	DM (+)	71.95 (15.33)	31.85 (9.56)	-40.05 (15.63)	Yes
	Control	70.58 (16.62)	29.10 (8.65)	-41.48 (15.27)	Yes
XXII Abreu et al.	DM (+)	38.24 (19.29) ♦	13.31 (15.12) ♦	-27.58 (21.90) ♦	Yes

2015	Control	63.00 (23.87) ♦	15.11 (20.27) ♦	-46.30 (23.62) ♦	Yes
XXIII Perruzo Lopes et al. 2017	DM I	37.77 (3.38)	5.67 (0.75)	-32.10 (0.51)	Yes
	DM II	51.36 (3.22)	12.73 (1.20)	-38.63 (2.00)	Yes
	Control	63.05 (2.78)	8.23 (0.35)	-54.82 (0.51)	Yes
XXVIII Almeida et al. 2019	DM	45.5 (22.4)	29.1 (15.6)	-16.5 (16.2) ♦	No
	Control	40.9 (18.9)	22.4 (13.3)	-18.5 (19.8) ♦	No

Abbreviations: ?: not specified/unclear, ♦: At request provided by the original author, ◊: calculated by the review authors, +: well controlled, -: poorly controlled, DM I: diabetes mellitus type I, DM II: diabetes mellitus type II, DM: diabetes mellitus

Online Appendix S6.3					
**Gingival bleeding index (GBI)** *Ainamo & Bay (1975)*	**Group**	**Baseline Mean % (SD)**	**End Mean % (SD)**	**Difference Mean % (SD)**	**Significant within groups**
V Da Cruz *et al.* 2008	DM	81.97 (25.11)	10.72 (3.97)	-71.85 (21.23) ♦	Yes
	Control	82.55 (23.81)	9.30 (2.95)	-72.26 (23.12) ♦	Yes
VII Correa et al. 2008	DM	46.8 (22.0) ♦	11.4 (10.8) ♦	-73.5 (19.5) ♦	Yes ♦
	Control	38.1 (14.9) ♦	3.7 (3.0) ♦	-34.5 (14.6) ♦	Yes ♦
VIII Gonçalves et al. 2008	DM (-)	47.3 (23.2) ♦	10.8 (10.4) ♦	-74.63 (19.02) ♦	?
	Control	38.4 (16.5) ♦	2.9 (2.3) ♦	-90.81 (9.50) ♦	?
IX Dag et al. 2009	DM (-)	33.0 (18.0) ◊	4.0 (2.0) ◊	-29.0 ◊	Yes
	DM (+)	28.0 (12.0) ◊	4.0 (1.0) ◊	-24.0 ◊	Yes
	Control	37.0 (18.0) ◊	3.0 (2.0) ◊	-34.0 ◊	Yes
XII Hungund et al. 2012	DM	85.92 (13.30)	54.66 (12.11)	-31.25 (1.19)	Yes
	Control	86.66 (13.54)	57.24 (8.24)	-29.41 (4.72)	Yes
XIV Camargo et al. 2013	DM	61.11 (15.91)	13.51 (3.9)	-47.6 (15.53) ♦	Yes
	Control	39.99 (20.31)	22.44 (14.87)	-16.95 (19.27) ♦	Yes
XV Buzinin et al. 2014	DM	36.7 (22.2)	12.15 (7.68)	24.55 ◊	Yes
	Control	34.04 (20.56)	11.76 (9.69)	22.28 ◊	Yes
XX Dogan et al. 2016	DM (+)	70.29 (8.35)	9.24(1.89)	-61.05 ◊	?
	Control	75.83 (10.07)	8.91(1.68)	-66.92 ◊	?

Abbreviations: ?: Not specified/unclear, ♦: At request provided by the original author, ◊: calculated by the review authors, +: well controlled, -: poorly controlled, DM I: diabetes mellitus type I, DM II: diabetes mellitus type II, DM: diabetes mellitus.

Online Appendix S6.3					
**Papilla bleeding index (PBI)** *Saxer & Mühlemann (1975)*	**Group**	**Baseline Mean % (SD)**	**End Mean % (SD)**	**Difference Mean % (SD)**	**Significant within groups**
III Christgau et al. 1998	DM	48.7 (20.3) ♦	23.2 (14.6) ♦	-25.5 (19.2) ♦	?
	Control	40.1 (19.9) ♦	17.8 (9.8)	-22.3 (13.8) ♦	?

Abbreviations: ?: not specified/unclear, ♦: At request provided by the original author, ◊: calculated by the review authors, +: well controlled, -: poorly controlled, DM I: diabetes mellitus type I, DM II: diabetes mellitus type II, DM: diabetes mellitus

Online Appendix S6.3					
**Sulcus bleeding index (SBI)** *Mühlemann & Son (1975)*	**Group**	**Baseline Mean (SD)**	**End Mean (SD)**	**Difference Mean (SD)**	**Significant within groups**
XIII Cirano et al. 2012	DM (-)	67.5 % (16.4)	17.6 % (10.5)	-49.9 % ◊	No
	Control	65.5 % (17.1)	19.1 % (11.4)	-46.4 % ◊	No
XXIV Sundaram et al. 2017	DM (-)	2.11 (0.59)	0.79 (0.29)	-1.32 ◊	Yes
	DM (+)	2.16 (0.53)	1.02 (0.31)	-1.14 ◊	Yes
	Control	2.16 (0.68)	0.92 (0.38)	-1.24 ◊	Yes

Abbreviations: ?: not specified/unclear, ♦: At request provided by the original author, ◊: calculated by the review authors, +: well controlled, -: poorly controlled, DM I: diabetes mellitus type I, DM II: diabetes mellitus type II, DM: diabetes mellitus

Online Appendix S6.3					
**Gingival Index** Loë & Silness (1963)	**Group**	**Baseline Mean (SD)**	**End Mean (SD)**	**Difference Mean (SD)**	**Significant within groups**
IX Dağ et al. 2009	DM (-)	1.32 (0.40)	0.13 (0.10)	-1.19 ◊	Yes
	DM (+)	1.04 (0.31)	0.09 (0.06)	-0.95 ◊	Yes
	Control	1.24 (0.40)	0.07 (0.03)	-1.17 ◊	Yes
XI Kudva et al. 2010	DM	1.38 (0.38)	0.73 (0.32)	-0.65 (0.27)	Yes
	Control	1.32 (0.20)	0.58 (0.14)	-0.74 (0.28)	Yes
XII Hungund et al. 2012	DM	1.81 (0.61)	1.22 (0.29)	-0.58 (0.32)	Yes
	Control	1.93 (0.36)	1.26 (0.20)	-0.66 (0.16)	Yes
XVII Kara et al. 2015	DM	1.60 (0.21)	0.58 (0.22)	-1.02 ◊	Yes
	Control	1.54 (0.29)	0.30 (0.09)	-1.24 ◊	Yes
XVIII Kaur et al. 2015	DM	1.57 (0.28)	0.64 (0.26)	-0.94 (0.26)	Yes
	DM (-)	1.60 (0.25)	0.76 (0.26)	-0.84 (0.26)	Yes
	DM (+)	1.56 (0.30)	0.50 (0.18)	-1.05 (0.22)	Yes
	Control	1.55 (0.28)	0.42 (0.19)	-1.13 (0.4)	Yes
XIX Pannicker et al. 2015	DM (+)	2.27 (0.19)	1.43 (0.26)	-0.84 ◊	Yes
	Control	2.23 (0.21)	1.46 (0.29)	-0.77 ◊	Yes

XX Dogan et al. 2016	DM (+)	2.29 (0.40)	0.76 (0.34)	-1.53 ◊	?
	Control	2.34 (0.34)	0.81 (0.30)	-1.53 ◊	?
XXI Mishra et al. 2016	DM (+)	2.51 (0.27) ♦	1.63 (0.24) ♦	-0.88 (0.13) ♦	?
	Control	2.08 (0.37) ♦	1.41 (0.27) ♦	-0.66 (0.31) ♦	?
XXIV Sundaram et al. 2017	DM (-)	2.46 (0.77)	0.94 (0.34)	-1.52 ◊	Yes
	DM (+)	2.66 (0.37)	0.67 (0.21)	-1.99 ◊	Yes
	Control	2.43 (0.62)	0.69 (0.26)	-1.74 ◊	Yes
XXVI Gayathri et al. 2019	DM (+)	2.03 (1.32)	1.71 (0.33)	-0.32 ◊	Yes
	Control	1.93 (0.30)	1.27 (0.23)	-0.66 ◊	Yes
XXVI Ahuja et al. 2019	DM (+)	2.05 (0.40)	0.85 (0.40)	-1.2 (0.52)	?
	Control	1.83 (0.30)	0.53 (0.30)	-1.3 (0.20)	?
XXVII Pragada et al. 2019	DM (+)	2.40 (0.33)	1.49 (1.80)	-0.91 ◊	Yes
	Control	2.11 (0.47)	1.02 (0.23)	-1.09 ◊	Yes
XXIX Mirnic et al. 2022	DM (-)	1.59 (0.58)	0.95 (0.46)	-0.64 (0.45)	No
	DM (+)	1.62 (0.64)	0.86 (0.37)	-0.76 (0.43)	No
	Control	0.94 (0.72)	0.37 (0.45)	-0.57 (0.53)	No
XXX Gomathi et al. 2023	DM (+)	2.95 (0.29)	1.48 (0.13)	-1.47 ◊	Yes
	Control	2.83 (0.26)	1.31 (0.18)	-1.52 ◊	Yes

Abbreviations: ?: not specified/unclear, ♦: At request provided by the original author, ◊: calculated by the review authors, +: well controlled, -: poorly controlled, DM I: diabetes mellitus type I, DM II: diabetes mellitus type II, DM: diabetes mellitus

Online Appendix S6.4						
**Plaque** Overall	**Index**	**Group**	**Baseline Mean (SD)**	**End Mean (SD)**	**Difference Mean (SD)**	**Significant within groups**
II Tervonen et al. 1997	PI *Silness & Loë (1964)*	DM	36.6 (21.6) %	27.8 (20.5) %	-8.8% ◊	?
		Control	28.8 (28.9) %	25.4 (21.8) %	-3.4% ◊	?
III Christgau et al. 1998	API *Lange (1986)*	DM	78.7 (19.7) % ♦	47.7 (25.1) % ♦	-31.1 (19.6) % ♦	Yes
		Control	79.7 (16.3) % ♦	42.2 (18.1) % ♦	-37.5 (18.7) % ♦	Yes
V Navarro-Sanchez et al. 2007	Supragingival plaque index	DM	84.7 (25.8) %	11.1 (12.2) %	-73.6 (23.1) %	Yes
		Control	83.9 (16.6) %	7.9 (9.7) %	-76.0 (14.0) %	Yes
VI Da Cruz et al.2008	VPI *Ainamo & Bay (1975)*	DM	90.24 (16.41) %	7.21 (2.98) %	-83.03(16.03) % ♦	Yes
		Control	91.33 (12.94) %	6.01 (2.66) %	-85.32(13.44) % ♦	Yes
VII Correa et al. 2008	VPI *Ainamo & Bay (1975)*	DM	84.5 (11.1) % ♦	18.2 (13.8) % ♦	-79.1 (13.9) % ♦	Yes ♦
		Control	65.4 (14.0) % ♦	8.6 (3.4) % ♦	-56.8 (13.6) % ♦	Yes ♦
VIII Gonçalves et al. 2008	VPI *Ainamo & Bay (1975)*	DM (-)	85.62 (11.33) % ♦	17.88 (14.23) % ♦	-79.79 (14.05) % ♦	?
		Control	65.29 (14.87) % ♦	8.06 (2.79) % ♦	-87.14 (4.79) % ♦	?
IX Dag et al. 2009	PI *Silness & Loë (1964)*	DM (-)	2.05 (0.68)	0.30 (0.22)	-1.75 ◊	Yes
		DM (+)	1.82 (0.66)	0.19 (0.05)	-1.63 ◊	Yes
		Control	2.34 (0.52)	0.19 (0.26)	2.15 ◊	Yes
X Kardesler et al. 2009	Dichotomous PI	DM (-)	99.7 (1.2) %	44.2 (6.3) %	-55.44 (6.86) % ♦	Yes
		DM (+)	97.4 (5.4) %	35.6 (8.7) %	-61.78 (9.69) % ♦	Yes
		Control	95.5 (9.4) %	39.5 (13.5) %	-56.10 (13.96) % ♦	Yes
XI Kudva et al. 2010	PI *Silness & Loë (1964)*	DM	1.51 (0.44)	0.79 (0.33)	-0.72 (0.37)	Yes
		Control	1.63 (0.46)	0.85 (0.20)	-0.88 (0.56)	Yes
XII Hungund et al. 2012	PI *O'leary's index*	DM	92.93 (12.85) %	64.53 (16.72) %	-28.40 (3.87) %	Yes
		Control	93.46 (10.63) %	70.66 (17.26) %	-22.80 (6.63) %	Yes
XIII Cirano et al. 2012	VPI *Ainamo & Bay (1975)*	DM (-)	67.5 (16.4) %	17.6 (10.5) %	-49.9 % ◊	Yes
		Control	65.5 (17.1) %	19.1 (11.4) %	-46.4 % ◊	Yes
XIV Camargo et al.2013	API *Lange (1986)*	DM	83.64 (14.09) %	29.39 (18.30) %	-54.25 (13.02) % ♦	Yes
		Control	52.85 (26.56) %	30.35 (13.95) %	-22.5 (35.56) % ♦	Yes
XV Buzinin et al. 2014	VPI *Ainamo & Bay (1975)*	DM	45.85 (27.39) %	10.80 (10.81) %	-35.05 % ◊	Yes
		Control	32.4 (20.60) %	9.8 (5.86) %	-22.06 % ◊	Yes

XVI López et al. 2014	Surfaces with plaque	DM	89.38 (13.0) %	54 (18) % ♦	-35.0 (5.0) % ♦	Yes
		DM (-)	79 (16) %	49 (16) % ♦	-29.0 (0.20) % ♦	?
		DM (+)	84 (22) %	55 (20) % ♦	-29.0 (1.7) % ♦	?
		Control	81.3 (18.7) %	53 (18.6) % ♦	-28 (0.1) % ♦	Yes
XVII Kara et al. 2015	PI	DM	1.65 (0.30)	0.46 (0.19)	-1.19 ◊	Yes
	Silness & Loë (1964)	Control	1.83 (0.03)	0.32 (0.17)	-1.51 ◊	Yes
XVIII Kaur et al. 2015	PI	DM	1.64 (0.26)	0.28 (0.09)	-1.36 (0.26)	Yes
		DM (-)	1.65 (0.23)	0.29 (0.08)	-1.36 (0.22)	No
		DM (+)	1.64 (0.30)	1.37 (0.29)	-0.27 (0.10)	Yes
		Control	1.66 (0.41)	0.27 (0.09)	-1.39 (0.37)	Yes
XIX Pannicker et al. 2015	PI	DM (+)	2.23 (0.19)	1.48 (0.23)	-0.75 ◊	Yes
	Silness & Loë (1964)	Control	2.16 (0.20)	1.45 (0.30)	-0.71 ◊	Yes
XX Dogan et al. 2016	PI	DM (+)	2.55 (0.33)	0.54 (0.31)	-2.01 ◊	?
	Silness & Loë (1964)	Control	2.38 (0.32)	0.54 (0.30)	-1.84 ◊	?
XXI Mishra et al. 2016	PI	DM (+)	2.19 (0.44) ♦	1.29 (0.20) ♦	-0.98 (0.40) ♦	?
		Control	2.27 (0.41) ♦	1.47 (0.29) ♦	0.72 (0.31) ♦	?
XXII Abreu et al. 2015	PI	DM (+)	72.96 (14.30) % ♦	19.07 (11.78) % ♦	-54.00 (26.27) % ♦	Yes
		Control	83.01 (12.01) % ♦	16.59 (24.27) % ♦	?	Yes
XXIII Perruzo Lopes et al. 2017	PI	DM I	36.00 (3.10) %	4.87 (0.55) %	-31.13 (0.60) %	Yes
		DM II	39.71 (3.01) %	12.26 (1.59) %	-27.51 (2.02) %	Yes
	Silness & Loë (1964)	Control	56.63 (2.98) %	31.52 (3.49) %	-25.11 (3.00) %	Yes
XXIV Sundaram et al. 2017	PI	DM (-)	1.66 (0.55)	0.60 (0.20)	-1.06 ◊	Yes
		DM (+)	2.11 (0.44)	0.94 (0.08)	-1.17 ◊	Yes
	Silness & Loë (1964)	Control	2.13 (0.69)	0.80 (0.24)	-1.33 ◊	Yes
XXVI Ahuja et al. 2019	PI	DM (+)	2.45 (0.33)	0.34 (0.20)	-2.11 (0.45)	?
	Silness & Loë (1964)	Control	2.09 (0.40)	1.03 (0.41)	-1.06 (0.58)	?
XXVII Pragada et al. 2019	PI	DM (+)	2.43 (0.46)	1.31 (0.48)	-1.12 ◊	Yes
		Control	2.19 (0.50)	1.02 (0.018)	-1.17 ◊	Yes
XXVIII Almeida et al. 2019	VPI	DM	62.3 (26.8) %	36.4 (15.8) %	-25.9 (22.4) % ♦	No
	Ainamo & Bay (1975)	Control	46.4 (19.1) %	23.4 (18.6) %	-23.0 (23.8) % ♦	No

XXIX Mirnic et al. 2021	PI	DM (-)	1.88 (0.37)	1.31 (0.37)	-0.42 ◊	Yes
		DM (+)	1.74 (0.48)	1.14 (0.46)	-0.31 ◊	Yes
	Silness & Loë (1964)	Control	1.32 (0.51)	0.66 (0.49)	-0.39 ◊	Yes
XXX Gomathi et al. 2023	PI	DM (+)	2.69 (0.41)	1.86 (0.12)	-0.83 ◊	Yes
	Silness & Loë (1964)	Control	2.78 (0.24)	1.40 (0.31)	-1.38 ◊	Yes

Abbreviations: ?: not specified/unclear, ♦: At request provided by the original author, ◊: calculated by the review authors, +: well controlled, -: poorly controlled, DM I: diabetes mellitus type I, DM II: diabetes mellitus type II, DM: diabetes mellitus

Online Appendix S6.4					
**Plaque index** *Silness & Loë (1964)*	**Group**	**Baseline Mean (SD)**	**End Mean (SD)**	**Difference Mean (SD)**	**Significant within groups**
II Tervonen et al. 1997	DM	36.6 (21.6)%	27.8 (20.5)%	-8.8% ◊	?
	Control	28.8 (28.9)%	25.4 (21.8)%	-3.4% ◊	?
IX Dag et al. 2009	DM (-)	2.05 (0.68)	0.30 (0.22)	-1.75 ◊	Yes
	DM (+)	1.82 (0.66)	0.19 (0.05)	-1.63 ◊	Yes
	Control	2.34 (0.52)	0.19 (0.26)	2,15 ◊	Yes
XI Kudva et al. 2010	DM	1.51 (0.44)	0.79 (0.33)	-0.72 (0.37)	Yes
	Control	1.63 (0.46)	0.85 (0.20)	-0.88 (0.56)	Yes
XVII Kara et al. 2015	DM	1.65 (0.30)	0.46 (0.19)	-1.19% ◊	Yes
	Control	1.83 (0.03)	0.32 (0.17)	-1.51% ◊	Yes
XVIII Kaur et al. 2015	DM	1.64 (0.26)	0.28 (0.09)	-1.36 (0.26)	Yes
	DM (-)	1.65 (0.23)	0.29 (0.08)	-1.36 (0.22)	No
	DM (+)	1.64 (0.30)	1.37 (0.29)	-0.27 (0.10)	Yes
	Control	1.66 (0.41)	0.27 (0.09)	-1.39 (0.37)	Yes
XIX Pannicker et al. 2015	DM (+)	2.23 (0.19)	1.48 (0.23)	-0.75 ◊	Yes
	Control	2.16 (0.20)	1.45 (0.30)	-0.71 ◊	Yes
XX Dogan et al. 2016	DM (+)	2.55 (0.33)	0.54 (0.31)	-2.01 ◊	?
	Control	2.38 (0.32)	0.54 (0.30)	-1.84 ◊	?
XXI Mishra et al. 2016	DM (+)	2.19 (0.44) ♦	1.29 (0.20) ♦	-0.98 (0.40) ♦	?
	Control	2.27 (0.41) ♦	1.47 (0.29) ♦	0.72 (0.31) ♦	?
XXIII Perruzo Lopes et al. 2017	DM I	36.00 (3.10)%	4.87 (0.55)%	-31.13 (0.60)%	Yes
	DM II	39.71 (3.01)%	12.26 (1.59) %	-27.51 (2.02)%	Yes
	Control	56.63 (2.98) %	31.52 (3.49)%	-25.11 (3.00)%	Yes
XXIV Sundaram et al. 2017	DM (-)	1.66 (0.55)	0.60 (0.20)	-1.06 ◊	Yes
	DM (+)	2.11 (0.44)	0.94 (0.08)	-1.17 ◊	Yes
	Control	2.13 (0.69)	0.80 (0.24)	-1.33 ◊	Yes
XXVI Ahuja et al. 2019	DM (+)	2.45 (0.33)	0.34 (0.20)	-2.11 (0.45)	?
	Control	2.09 (0.40)	1.03 (0.41)	-1.06 (0.58)	?
XXVII Pragada et	DM (+)	2.43 (0.46)	1.31 (0.48)	-1.12 ◊	Yes

al. 2019	Control	2.19 (0.50)	1.02 (0.018)	-1.17 ◊	Yes
XXIX Mirnic et al. 2021	DM (-)	1.88 (0.37)	1.31 (0.37)	-0.42 ◊	Yes
	DM (+)	1.74 (0.48)	1.14 (0.46)	-0.31 ◊	Yes
	Control	1.32 (0.51)	0.66 (0.49)	-0.39 ◊	Yes
XXX Gomathi et al. 2023	DM (+)	2.69 (0.41)	1.86 (0.12)	-0.83 ◊	Yes
	Control	2.78 (0.24)	1.40 (0.31)	-1.38 ◊	Yes

**Online Appendix S6.4**					
**Visible plaque index (VPI)** *Ainamo & Bay (1975)*	**Group**	**Baseline Mean % (SD)**	**End Mean % (SD)**	**Difference Mean % (SD)**	**Significant within groups**
VI Da Cruz et al.2008	DM	90.24 (16.41)	7.21 (2.98)	-83.03 (16.03) ♦	Yes
	Control	91.33 (12.94)	6.01 (2.66)	-85.32 (13.44) ♦	Yes
VII Correa et al. 2008	DM	84.5 (11.1) ♦	18.2 (13.8) ♦	-79.1 (13.9) ♦	Yes ♦
	Control	65.4 (14.0) ♦	8.6 (3.4) ♦	-56.8 (13.6) ♦	Yes ♦
VIII Gonçalves et al. 2008	DM (-)	85.62 (11.33) ♦	17.88 (14.23) ♦	-79.79 (14.05) ♦	?
	Control	65.29 (14.87) ♦	8.06 (2.79) ♦	-87.14 (4.79) ♦	?
XIII Cirano et al. 2012	DM (-)	67.5 (16.4)	17.6 (10.5)	-49.9 ◊	Yes
	Control	65.5 (17.1)	19.1 (11.4)	-46.4 ◊	Yes
XV Buzinin et al. 2014	DM	45.85 (27.39)	10.80 (10.81)	-35.05 ◊	Yes
	Control	32.4 (20.60)	9.8 (5.86)	-22.06 ◊	Yes
XXVIII Almeida et al. 2019	DM	62.3 (26.8)	36.4 (15.8)	-25.9 (22.4) ♦	No
	Control	46.4 (19.1)	23.4 (18.6)	-23.0 (23.8) ♦	No

Abbreviations: ?: Not specified/unclear, ♦: At request provided by the original author, ◊: calculated by the review authors, +: well controlled, -: poorly controlled, DM I: diabetes mellitus type I, DM II: diabetes mellitus type II, DM: diabetes mellitus.

Online Appendix S6.4					
**Approximal plaque index (API)** *Lange (1986)*	**Group**	**Baseline Mean % (SD)**	**End Mean % (SD)**	**Difference Mean % (SD)**	**Significant within groups**
III Christgau et al. 1998	DM	78.7 (19.7) ♦	47.7 (25.1) ♦	-31.1 (19.6) ♦	Yes
	Control	79.7 (16.3) ♦	42.2 (18.1) ♦	-37.5 (18.7) ♦	Yes
XIV Camargo et al.2013	DM	83.64 (14.09)	29.39 (18.30)	-54.25 (13.02) ♦	Yes
	Control	52.85 (26.56)	30.35 (13.95)	-22.5 (35.56) ♦	Yes

Abbreviations: ?: Not specified/unclear, ♦: At request provided by the original author, ◊: calculated by the review authors, +: well controlled, -: poorly controlled, DM I: diabetes mellitus type I, DM II: diabetes mellitus type II, DM: diabetes mellitus.

Online Appendix S6.5					
**Gingival recession (REC)**	**Group**	**Baseline Mean (SD)**	**End Mean (SD)**	**Difference Mean (SD)**	**Significant within groups**
V Navarro-Sanchez et al. 2007	DM	1.6 (1.7) mm	2.2 (1.7) mm	+0.6 (0.3) mm	Yes
	Control	1.2 (0.7) mm	1.9 (0.7) mm	+0.7 (0.3) mm	Yes
VI Da Cruz et al.2008	DM	2.86 (0.69) mm	2.98 (0.7) mm	+0.12 (0.2) mm	Yes
	Control	2.17 (0.9) mm	2.34 (0.65) mm	+0.18 (0.31) mm	Yes
XIII Cirano et al. 2012	DM (-)	0.6 (0.5) mm	1.0 (0.7) mm	+0.4 mm ◊	Yes
	Control	0.4 (0.6) mm	1.0 (0.7) mm	+0.6mm ◊	Yes
XIV Camargo et al. 2013	DM	1.68 (0.72) mm	1.51 (0.62) mm	-0.18 (0.23) mm	No
	Control	1.9 (0.85) mm	2.02 (0.41) mm	+0.13 (0.77) mm	No

Abbreviations: ?: not specified/unclear, ♦: At request provided by the original author, ◊: calculated by the review authors, +: well controlled, -: poorly controlled, DM I: diabetes mellitus type I, DM II: diabetes mellitus type II, DM: diabetes mellitus

Online Appendix S7.1

Meta-analysis evaluating the effect of NSPT on DM compared to non-DM regarding PPD at baseline

	DM			Control				Mean Difference
Study or Subgroup	Mean	SD	Total	Mean	SD	Total	Weight	IV, Random, 95% CI
Abreu et al. 2016	3.16	0.39	30	2.97	0.63	30	3.9%	0.19 [−0.08, 0.46]
Ahuja et al. 2019	5.6	0.38	30	4.98	0.49	30	4.1%	0.62 [0.40, 0.84]
Almeida et al. 2020	2.84	0.67	26	2.79	0.74	28	3.4%	0.05 [−0.33, 0.43]
Camargo et al. 2013	3.46	0.55	10	2.81	0.55	10	3.0%	0.65 [0.17, 1.13]
Christgau et al. 1998	3.6	1.6	20	3.8	1.7	20	1.4%	−0.20 [−1.22, 0.82]
Cirano et al. 2012	5.3	0.3	16	5.9	0.2	15	4.2%	−0.60 [−0.78, −0.42]
Correa et al. 2008	4.2	1	23	3.6	0.6	26	3.0%	0.60 [0.13, 1.07]
Da Cruz et al. 2008	5.72	1.13	10	4.7	0.9	10	1.6%	1.02 [0.12, 1.92]
Dag et al. DM + 2009	2.67	0.45	15	2.61	0.38	8	3.6%	0.06 [−0.29, 0.41]
Dag et al. DM − 2009	2.84	0.65	15	2.61	0.38	8	3.3%	0.23 [−0.19, 0.65]
Dogan et al. 2016	4.11	0.34	20	4.09	0.37	20	4.1%	0.02 [−0.20, 0.24]
Gayathri et al. 2019	3.57	0.4	30	3.29	0.42	30	4.1%	0.28 [0.07, 0.49]
Gomathi et al. 2023	5.6	0.54	15	4.72	0.4	15	3.6%	0.88 [0.54, 1.22]
Goncalvez /Silva et al.	4.18	0.9	20	3.62	0.56	20	3.1%	0.56 [0.10, 1.02]
Hungund et al. 2010	3.16	0.65	15	3.04	0.64	15	3.1%	0.12 [−0.34, 0.58]
Kara et al. 2015	4.13	0.44	15	4.12	0.428	15	3.7%	0.01 [−0.30, 0.32]
Kardesler et al. DM+ 2009	3.7	0.4	13	3.9	0.6	8	3.0%	−0.20 [−0.67, 0.27]
Kardesler et al. DM− 2009	4.1	1	12	3.9	0.6	8	2.2%	0.20 [−0.50, 0.90]
Kaur et al. 2015	2.96	0.46	50	2.77	0.58	25	3.9%	0.19 [−0.07, 0.45]
Kudva et al. 2010	3.31	1.54	15	3.63	1.61	15	1.2%	−0.32 [−1.45, 0.81]
Lopez et al. 2014	3.3	0.5	26	3.1	0.5	26	3.9%	0.20 [−0.07, 0.47]
Mirnic et al. DM + 2022	2.09	0.51	29	2.38	0.6	16	3.6%	−0.29 [−0.64, 0.06]
Mirnic et al. DM − 2022	2.11	0.47	32	2.38	0.6	16	3.6%	−0.27 [−0.61, 0.07]
Mishra et al. 2016	7.43	0.94	14	6.64	0.5	14	2.7%	0.79 [0.23, 1.35]
Navarro-Sanchez /Faria	4.1	0.3	8	3.7	0.5	10	3.5%	0.40 [0.03, 0.77]
Pannicker et al. 2016	7.94	1.57	35	7.45	1.68	35	2.0%	0.49 [−0.27, 1.25]
Peruzzo Lopes DM1 2017	1.83	0.05	14	2.12	0.06	6	4.5%	−0.29 [−0.34, −0.24]
Peruzzo Lopes DM2 2017	2.33	0.06	27	2.12	0.06	6	4.5%	0.21 [0.16, 0.26]
Pragada et al. 2019	7.3	1.69	30	6.83	1.7	30	1.7%	0.47 [−0.39, 1.33]
Sonoki et al. 2006	3.3	0.63	5	2.93	0.81	6	1.7%	0.37 [−0.48, 1.22]
Sundaram et al. DM+ 2017	4.04	1.02	40	3.84	1.33	20	2.3%	0.20 [−0.46, 0.86]
Sundaram et al. DM− 2017	4.8	0.57	40	3.84	1.33	20	2.5%	0.96 [0.35, 1.57]
**Total (95% CI)**			**700**			**561**	**100.0%**	**0.21 [0.07, 0.35]**

Heterogeneity: Tau^2 = 0.12; Chi^2 = 337.43, df = 31 (P < 0.00001); I^2 = 91%

Test for overall effect: Z = 2.86 (P = 0.004)

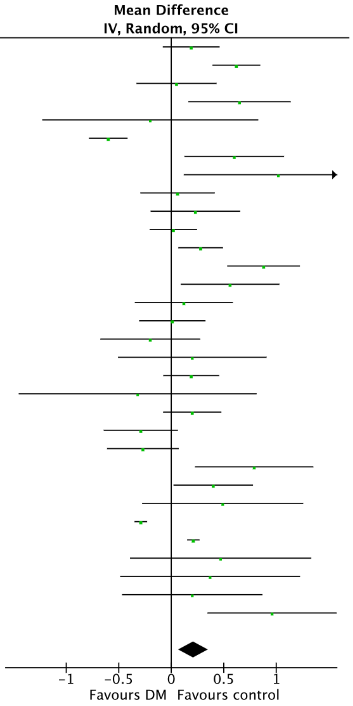

Online Appendix S7.2

Meta-analysis evaluating the effect of NSPT on DM compared to non-DM regarding PPD post-NSPT

	DM			Control				Mean Difference
Study or Subgroup	Mean	SD	Total	Mean	SD	Total	Weight	IV, Random, 95% CI
Abreu et al. 2016	2.54	0.74	30	2.2	0.79	30	3.1%	0.34 [−0.05, 0.73]
Ahuja et al. 2019	3.07	0.6	30	2.42	0.72	30	3.3%	0.65 [0.31, 0.99]
Almeida et al. 2020	2.4	0.58	26	2.32	0.43	28	3.6%	0.08 [−0.19, 0.35]
Camargo et al. 2013	2.62	0.6	10	2.49	0.48	10	2.7%	0.13 [−0.35, 0.61]
Christgau et al. 1998	3.2	1.2	20	3.3	1.3	20	1.7%	−0.10 [−0.88, 0.68]
Cirano et al. 2012	3	0.5	16	3.2	0.5	15	3.2%	−0.20 [−0.55, 0.15]
Correa et al. 2008	2.8	0.4	23	2.4	0.3	26	3.9%	0.40 [0.20, 0.60]
Da Cruz et al. 2008	5	1.29	10	3.97	0.82	10	1.3%	1.03 [0.08, 1.98]
Dag et al. DM + 2009	2.3	0.43	15	2.36	0.68	8	2.5%	−0.06 [−0.58, 0.46]
Dag et al. DM − 2009	2.37	0.59	15	2.36	0.68	8	2.4%	0.01 [−0.55, 0.57]
Dogan et al. 2016	2.67	0.26	20	2.61	0.3	20	4.0%	0.06 [−0.11, 0.23]
Gayathri et al. 2019	3.44	0.42	30	3.16	0.46	30	3.8%	0.28 [0.06, 0.50]
Gomathi et al. 2023	2.97	0.2	15	3	0.16	15	4.1%	−0.03 [−0.16, 0.10]
Goncalvez /Silva et al.	2.76	0.41	20	2.44	0.3	20	3.8%	0.32 [0.10, 0.54]
Hungund et al. 2010	2.67	0.43	15	2.03	0.45	15	3.4%	0.64 [0.33, 0.95]
Kara et al. 2015	2.34	0.258	15	2.31	0.288	15	3.9%	0.03 [−0.17, 0.23]
Kardesler et al. DM+ 2009	2.8	0.4	13	2.7	0.3	8	3.5%	0.10 [−0.20, 0.40]
Kardesler et al. DM− 2009	3.1	0.6	12	2.7	0.3	8	3.0%	0.40 [0.00, 0.80]
Kaur et al. 2015	2.15	0.42	50	1.91	0.44	25	3.8%	0.24 [0.03, 0.45]
Kudva et al. 2010	1.69	1.06	15	1.78	1.06	15	1.7%	−0.09 [−0.85, 0.67]
Lopez et al. 2014	2.79	0.58	24	2.75	0.57	26	3.4%	0.04 [−0.28, 0.36]
Mirnic et al. DM + 2022	1.98	0.53	29	2.05	0.52	16	3.4%	−0.07 [−0.39, 0.25]
Mirnic et al. DM − 2022	2.03	0.35	32	2.05	0.52	16	3.6%	−0.02 [−0.30, 0.26]
Mishra et al. 2016	7	0.78	14	6.36	0.5	14	2.7%	0.64 [0.15, 1.13]
Navarro-Sanchez /Faria	3	0.4	8	2.6	0.2	10	3.5%	0.40 [0.10, 0.70]
Pannicker et al. 2016	5.74	1.24	35	5.62	1.33	35	2.2%	0.12 [−0.48, 0.72]
Peruzzo Lopes DM1 2017	1.36	0.02	14	1.76	0.04	6	4.3%	−0.40 [−0.43, −0.37]
Peruzzo Lopes DM2 2017	1.83	0.06	27	1.76	0.04	6	4.2%	0.07 [0.03, 0.11]
Pragada et al. 2019	5.26	1.18	30	5.06	1.36	30	2.1%	0.20 [−0.44, 0.84]
Sonoki et al. 2006	2.58	0.38	5	2.16	0.42	6	2.7%	0.42 [−0.05, 0.89]
Sundaram et al. DM+ 2017	1.88	0.54	40	1.87	1.06	20	2.6%	0.01 [−0.48, 0.50]
Sundaram et al. DM− 2017	1.99	0.71	40	1.87	1.06	20	2.6%	0.12 [−0.39, 0.63]
**Total (95% CI)**			**698**			**561**	**100.0%**	**0.16 [0.03, 0.29]**

Heterogeneity: Tau^2 = 0.10; Chi^2 = 523.45, df = 31 (P < 0.00001); I^2 = 94%

Test for overall effect: Z = 2.49 (P = 0.01)

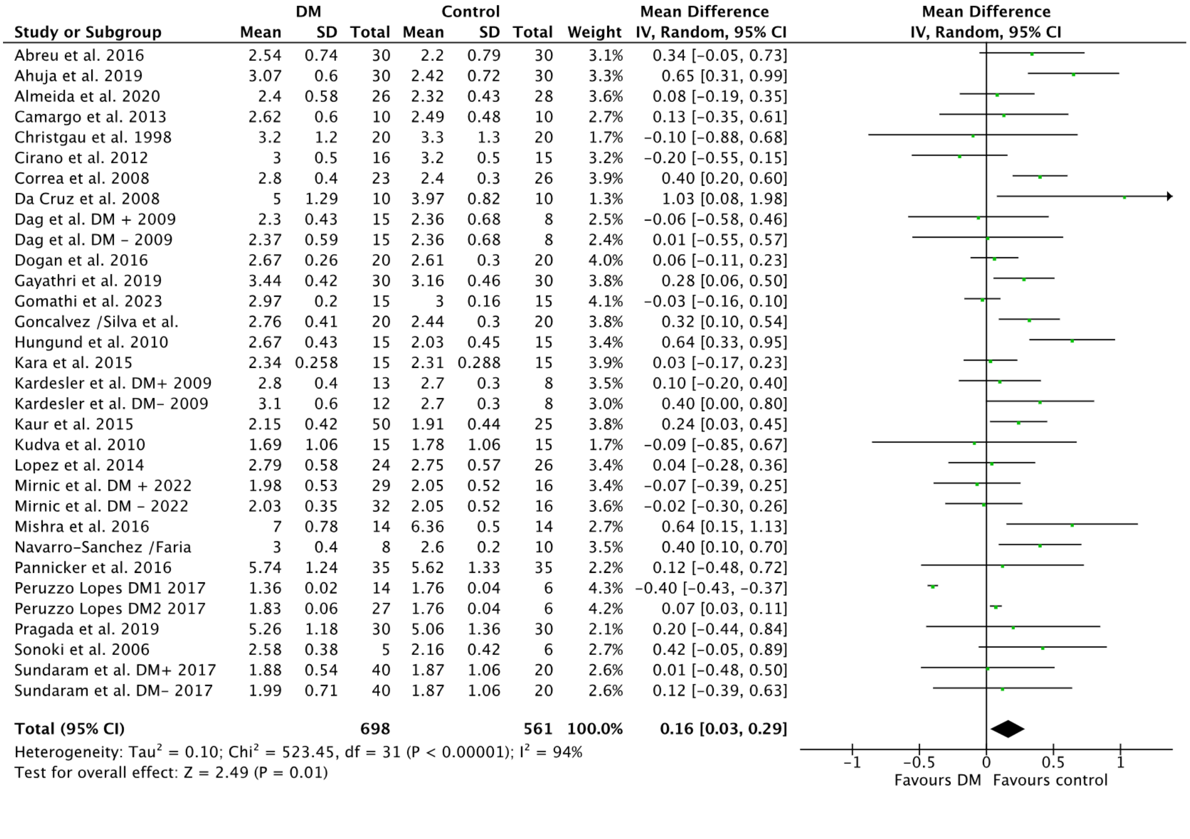

Online Appendix S7.3

Meta-analysis evaluating the effect of NSPT on DM compared to non-DM regarding PPD on incremental scores

Study or Subgroup	DM Mean	DM SD	DM Total	Control Mean	Control SD	Control Total	Weight	Mean Difference IV, Random, 95% CI
Abreu et al. 2016	−1.12	0.07	30	−0.74	0.74	30	4.3%	−0.38 [−0.65, −0.11]
Ahuja et al. 2019	−2.53	0.24	30	−2.57	0.26	30	6.9%	0.04 [−0.09, 0.17]
Almeida et al. 2020	−0.44	0.28	26	−0.47	0.6	28	4.6%	0.03 [−0.22, 0.28]
Camargo et al. 2013	−0.9	0.37	10	−0.33	0.31	10	3.8%	−0.57 [−0.87, −0.27]
Christgau et al. 1998	−0.4	1.1	20	−0.5	1	20	1.2%	0.10 [−0.55, 0.75]
Correa et al. 2008	−1.5	0.7	23	−1.2	0.6	26	2.9%	−0.30 [−0.67, 0.07]
Da Cruz et al. 2008	−0.71	0.19	10	−0.82	0.13	10	6.6%	0.11 [−0.03, 0.25]
Goncalvez /Silva et al.	−1.42	0.61	20	−1.17	0.54	20	3.0%	−0.25 [−0.61, 0.11]
Hungund et al. 2010	−0.49	0.18	15	−1.01	0.19	15	6.8%	0.52 [0.39, 0.65]
Kardesler et al. DM+ 2009	−0.93	0.37	13	−1.17	0.51	8	2.6%	0.24 [−0.17, 0.65]
Kardesler et al. DM− 2009	−1.06	0.54	12	−1.17	0.51	8	2.1%	0.11 [−0.36, 0.58]
Kaur et al. 2015	−0.81	0.28	50	−0.86	0.29	25	6.7%	0.05 [−0.09, 0.19]
Kudva et al. 2010	−1.62	0.79	15	−1.85	0.81	15	1.5%	0.23 [−0.34, 0.80]
Lopez et al. 2014	−0.51	0.08	24	−0.35	0.07	26	8.2%	−0.16 [−0.20, −0.12]
Mirnic et al. DM + 2022	−0.11	0.16	29	−0.34	0.23	16	6.9%	0.23 [0.10, 0.36]
Mirnic et al. DM − 2022	−0.08	0.27	32	−0.34	0.23	16	6.5%	0.26 [0.11, 0.41]
Mishra et al. 2016	−0.43	0.51	14	−0.29	0.47	14	3.0%	−0.14 [−0.50, 0.22]
Navarro-Sanchez /Faria	−1.1	0.2	8	−1.1	0.3	10	4.8%	0.00 [−0.23, 0.23]
Peruzzo Lopes DM1 2017	−0.47	0.02	14	−0.48	0.04	6	8.3%	0.01 [−0.02, 0.04]
Peruzzo Lopes DM2 2017	−0.5	0.03	27	−0.48	0.04	6	8.2%	−0.02 [−0.05, 0.01]
Sonoki et al. 2006	−0.76	0.56	5	−0.72	0.46	6	1.4%	−0.04 [−0.65, 0.57]
**Total (95% CI)**			**427**			**345**	**100.0%**	**0.02 [−0.06, 0.10]**

Heterogeneity: $Tau^2 = 0.02$; $Chi^2 = 171.07$, df = 20 ($P < 0.00001$); $I^2 = 88\%$

Test for overall effect: Z = 0.57 (P = 0.57)

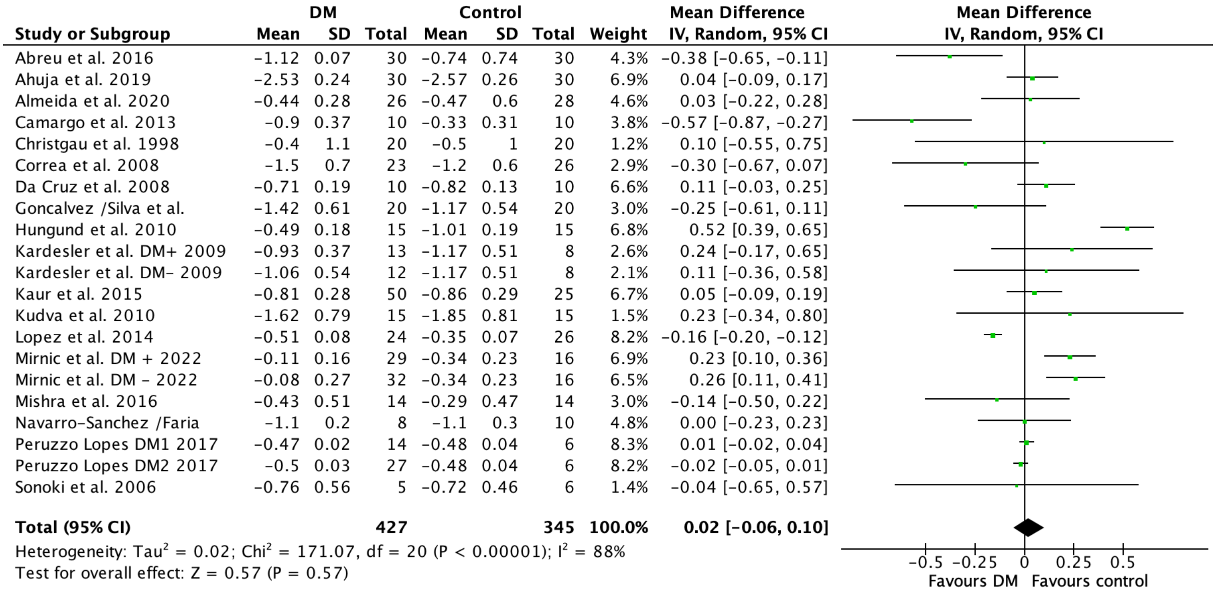

Online Appendix S7.4

Meta-analysis evaluating the effect of NSPT on DM compared to non-DM regarding CAL at baseline

Study or Subgroup	DM Mean	DM SD	DM Total	Control Mean	Control SD	Control Total	Weight	Mean Difference IV, Random, 95% CI
Abreu et al. 2016	4.15	0.99	30	3.77	0.71	30	4.1%	0.38 [−0.06, 0.82]
Ahuja et al. 2019	6.01	0.38	30	5.35	0.55	30	5.6%	0.66 [0.42, 0.90]
Almeida et al. 2020	3.83	2.02	26	2.36	1.57	28	1.6%	1.47 [0.50, 2.44]
Camargo et al. 2013	3.71	0.62	10	3.3	0.9	10	2.6%	0.41 [−0.27, 1.09]
Christgau et al. 1998	4.3	2	20	4.3	1.9	20	1.1%	0.00 [−1.21, 1.21]
Cirano et al. 2012	6	0.7	16	6.3	0.7	15	3.7%	−0.30 [−0.79, 0.19]
Correa et al. 2008	5.4	1.3	23	4.5	1.1	26	2.6%	0.90 [0.22, 1.58]
Da Cruz et al. 2008	4.49	0.7	10	4.03	0.37	10	3.7%	0.46 [−0.03, 0.95]
Dag et al. DM + 2009	4.25	0.82	15	4.31	0.59	8	3.1%	−0.06 [−0.64, 0.52]
Dag et al. DM − 2009	4.3	0.97	15	4.31	0.59	8	2.8%	−0.01 [−0.65, 0.63]
Dogan et al. 2016	4.31	0.36	20	4.25	0.35	20	5.8%	0.06 [−0.16, 0.28]
Gayathri et al. 2019	3.65	0.45	30	3.6	0.42	30	5.8%	0.05 [−0.17, 0.27]
Gomathi et al. 2023	4.36	0.82	15	4.38	0.65	15	3.4%	−0.02 [−0.55, 0.51]
Goncalvez /Silva et al.	4.97	1.45	20	4.25	1.01	20	2.2%	0.72 [−0.05, 1.49]
Kara et al. 2015	4.78	0.579	15	4.49	0.411	15	4.7%	0.29 [−0.07, 0.65]
Kardesler et al. DM+ 2009	4.3	0.9	13	4.3	0.7	8	2.6%	0.00 [−0.69, 0.69]
Kardesler et al. DM− 2009	4.4	1.1	12	4.3	0.7	8	2.1%	0.10 [−0.69, 0.89]
Kaur et al. 2015	3.46	0.53	50	3.14	0.53	25	5.5%	0.32 [0.07, 0.57]
Lopez et al. 2014	3.6	0.8	26	3.6	0.8	26	4.1%	0.00 [−0.43, 0.43]
Mirnic et al. DM + 2022	2.81	0.98	29	2.32	1.39	16	2.2%	0.49 [−0.28, 1.26]
Mirnic et al. DM − 2022	2.62	1.36	32	2.32	1.39	16	2.0%	0.30 [−0.53, 1.13]
Mishra et al. 2016	2.51	0.27	14	2.08	0.37	14	5.6%	0.43 [0.19, 0.67]
Navarro-Sanchez /Faria	5.7	1.9	8	4.9	0.8	10	0.9%	0.80 [−0.61, 2.21]
Pannicker et al. 2016	11.97	2.43	35	9.82	1.31	35		Not estimable
Peruzzo Lopes DM1 2017	2.62	0.09	14	2.49	0.06	6	6.6%	0.13 [0.06, 0.20]
Peruzzo Lopes DM2 2017	3.1	0.12	27	2.49	0.06	6	6.6%	0.61 [0.54, 0.68]
Pragada et al. 2019	10.07	1.63	30	9.67	1.51	30		Not estimable
Sundaram et al. DM+ 2017	4.36	0.67	40	4.13	0.79	20	4.3%	0.23 [−0.17, 0.63]
Sundaram et al. DM− 2017	4.03	0.47	40	4.13	0.79	20	4.5%	−0.10 [−0.48, 0.28]
**Total (95% CI)**			**600**			**460**	**100.0%**	**0.27 [0.13, 0.41]**

Heterogeneity: $Tau^2 = 0.08$; $Chi^2 = 152.44$, df = 26 ($P < 0.00001$); $I^2 = 83\%$

Test for overall effect: Z = 3.80 (P = 0.0001)

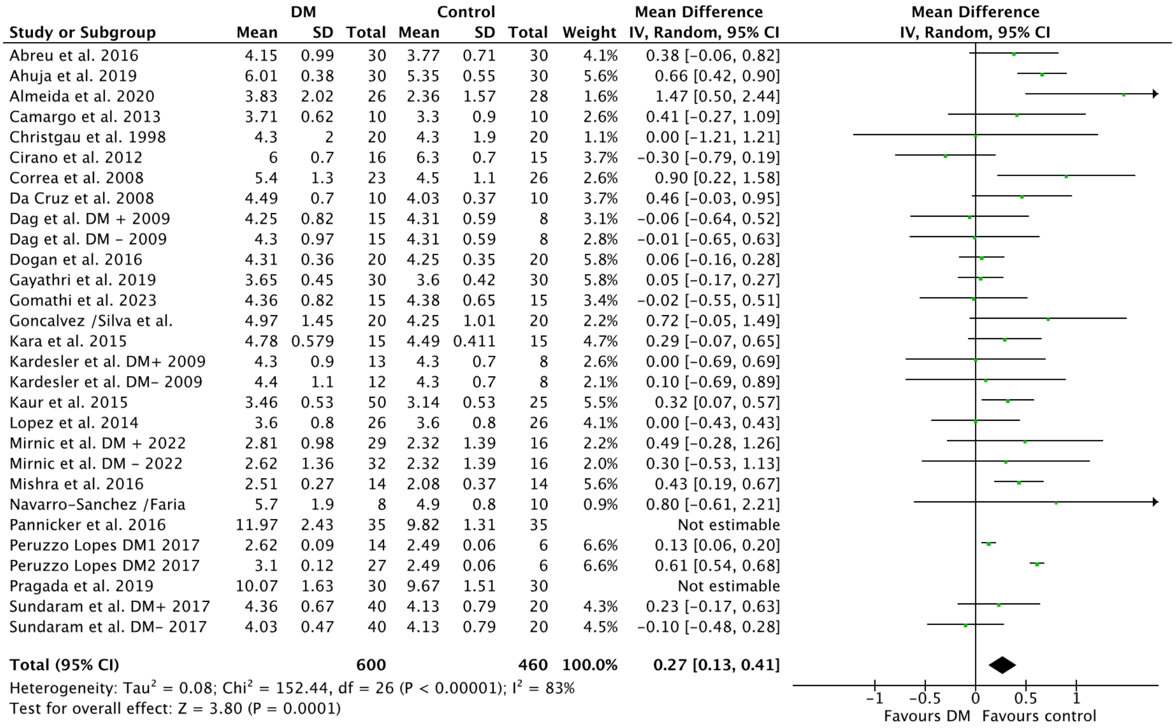

Online Appendix S7.5

Meta-analysis evaluating the effect of NSPT on DM compared to non-DM regarding CAL post-NSPT

Study or Subgroup	DM Mean	DM SD	DM Total	Control Mean	Control SD	Control Total	Weight	Mean Difference IV, Random, 95% CI
Abreu et al. 2016	3.58	0.92	30	3.1	1.12	30	3.8%	0.48 [−0.04, 1.00]
Ahuja et al. 2019	3.75	0.86	30	2.81	0.78	30	4.1%	0.94 [0.52, 1.36]
Almeida et al. 2020	3.52	1.91	26	2.11	1.2	28	2.7%	1.41 [0.55, 2.27]
Camargo et al. 2013	2.85	0.66	10	2.92	0.48	10	3.8%	−0.07 [−0.58, 0.44]
Christgau et al. 1998	4.1	1.8	20	4.1	1.8	20	2.1%	0.00 [−1.12, 1.12]
Cirano et al. 2012	3.9	0.8	16	4.2	0.9	15	3.5%	−0.30 [−0.90, 0.30]
Correa et al. 2008	4.6	1.1	23	4	0.8	26	3.7%	0.60 [0.06, 1.14]
Da Cruz et al. 2008	3.28	0.54	10	2.9	0.26	10	4.2%	0.38 [0.01, 0.75]
Dag et al. DM + 2009	3.03	0.79	15	2.91	0.56	8	3.6%	0.12 [−0.44, 0.68]
Dag et al. DM – 2009	3.04	0.81	15	2.91	0.56	8	3.6%	0.13 [−0.43, 0.69]
Dogan et al. 2016	3.09	0.43	20	2.88	0.38	20	4.5%	0.21 [−0.04, 0.46]
Gayathri et al. 2019	3.61	0.48	30	3.47	0.58	30	4.5%	0.14 [−0.13, 0.41]
Gomathi et al. 2023	3.03	0.19	15	3.05	0.15	15	4.8%	−0.02 [−0.14, 0.10]
Goncalvez /Silva et al.	4.34	1.29	20	3.97	0.83	20	3.3%	0.37 [−0.30, 1.04]
Kara et al. 2015	3.06	0.726	15	2.75	0.498	15	4.0%	0.31 [−0.14, 0.76]
Kardesler et al. DM+ 2009	3.9	0.9	13	3.9	0.7	8	3.2%	0.00 [−0.69, 0.69]
Kardesler et al. DM– 2009	4.2	0.9	12	3.9	0.7	8	3.2%	0.30 [−0.40, 1.00]
Kaur et al. 2015	2.75	0.62	50	2.41	0.55	25	4.5%	0.34 [0.06, 0.62]
Lopez et al. 2014	3.57	0.99	24	3.43	0.74	26	3.9%	0.14 [−0.35, 0.63]
Mirnic et al. DM + 2022	2.57	1.01	29	1.98	1.31	16	3.1%	0.59 [−0.15, 1.33]
Mirnic et al. DM – 2022	2.39	1.27	32	1.98	1.31	16	2.9%	0.41 [−0.37, 1.19]
Mishra et al. 2016	1.63	0.24	14	1.41	0.27	14	4.7%	0.22 [0.03, 0.41]
Navarro-Sanchez /Faria	5.2	2	8	4.5	0.8	10	1.5%	0.70 [−0.77, 2.17]
Pannicker et al. 2016	9.85	2.39	35	7.48	1.22	35		Not estimable
Peruzzo Lopes DM1 2017	1.56	0.04	14	1.82	0.04	6	4.8%	−0.26 [−0.30, −0.22]
Peruzzo Lopes DM2 2017	2.44	0.12	27	1.82	0.04	6	4.8%	0.62 [0.56, 0.68]
Pragada et al. 2019	7.53	1.04	30	7.1	1.21	30		Not estimable
Sundaram et al. DM+ 2017	3.06	1.05	40	2.97	0.99	20	3.7%	0.09 [−0.45, 0.63]
Sundaram et al. DM– 2017	2.73	1.12	40	2.97	0.99	20	3.6%	−0.24 [−0.80, 0.32]
**Total (95% CI)**			**598**			**460**	**100.0%**	**0.26 [0.05, 0.47]**

Heterogeneity: $Tau^2 = 0.25$; $Chi^2 = 714.98$, df = 26 ($P < 0.00001$); $I^2 = 96\%$
Test for overall effect: Z = 2.39 (P = 0.02)

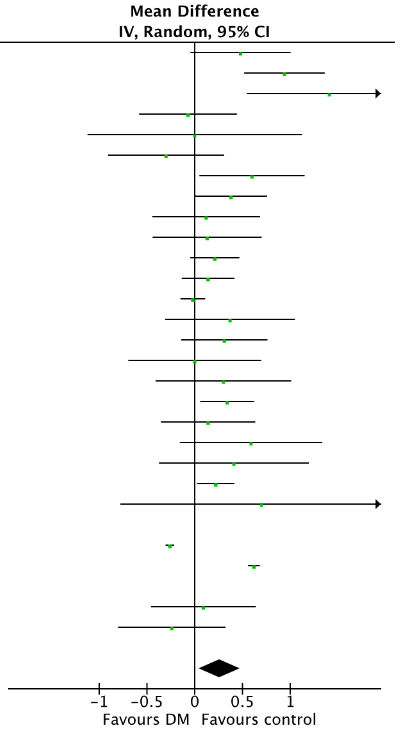

Online Appendix S7.6

Meta-analysis evaluating the effect of NSPT on DM compared to non-DM regarding CAL on incremental scores

Study or Subgroup	DM Mean	DM SD	DM Total	Control Mean	Control SD	Control Total	Weight	Mean Difference IV, Random, 95% CI
Abreu et al. 2016	−1.28	1.63	30	−0.64	1.09	30	2.5%	−0.64 [−1.34, 0.06]
Ahuja et al. 2019	−2.25	0.67	30	−2.55	0.25	30	5.9%	0.30 [0.04, 0.56]
Almeida et al. 2020	−0.3	0.79	26	−0.25	0.74	28	4.4%	−0.05 [−0.46, 0.36]
Camargo et al. 2013	−0.85	0.28	10	−0.37	0.63	10	4.3%	−0.48 [−0.91, −0.05]
Christgau et al. 1998	−0.2	1.1	20	−0.2	1.1	20	2.6%	0.00 [−0.68, 0.68]
Correa et al. 2008	−0.8	0.6	23	−0.6	0.6	26	5.1%	−0.20 [−0.54, 0.14]
Da Cruz et al. 2008	−1.21	0.24	10	−1.12	0.15	10	6.6%	−0.09 [−0.27, 0.09]
Goncalvez /Silva et al.	−0.63	0.43	20	−0.46	0.4	20	5.9%	−0.17 [−0.43, 0.09]
Kardesler et al. DM+ 2009	−0.3	0.5	13	−0.4	0.6	8	3.7%	0.10 [−0.40, 0.60]
Kardesler et al. DM– 2009	−0.2	0.5	12	−0.4	0.6	8	3.7%	0.20 [−0.30, 0.70]
Kaur et al. 2015	−0.71	0.36	50	−0.73	0.31	25	6.8%	0.02 [−0.14, 0.18]
Lopez et al. 2014	−0.03	0.19	24	−0.17	0.06	26	7.3%	0.14 [0.06, 0.22]
Mirnic et al. DM + 2022	−0.24	0.24	29	−0.34	0.3	16	6.7%	0.10 [−0.07, 0.27]
Mirnic et al. DM – 2022	−0.23	0.27	32	−0.34	0.3	16	6.6%	0.11 [−0.06, 0.28]
Mishra et al. 2016	−0.88	0.13	14	−0.66	0.31	14	6.6%	−0.22 [−0.40, −0.04]
Navarro-Sanchez /Faria	−0.5	0.2	8	−0.4	0.2	10	6.5%	−0.10 [−0.29, 0.09]
Peruzzo Lopes DM1 2017	−1.06	0.04	14	−0.67	0.04	6	7.4%	−0.39 [−0.43, −0.35]
Peruzzo Lopes DM2 2017	−0.66	0.08	27	−0.67	0.04	6	7.4%	0.01 [−0.03, 0.05]
**Total (95% CI)**			**392**			**309**	**100.0%**	**−0.06 [−0.19, 0.07]**

Heterogeneity: $Tau^2 = 0.06$; $Chi^2 = 295.18$, df = 17 ($P < 0.00001$); $I^2 = 94\%$
Test for overall effect: Z = 0.87 (P = 0.38)

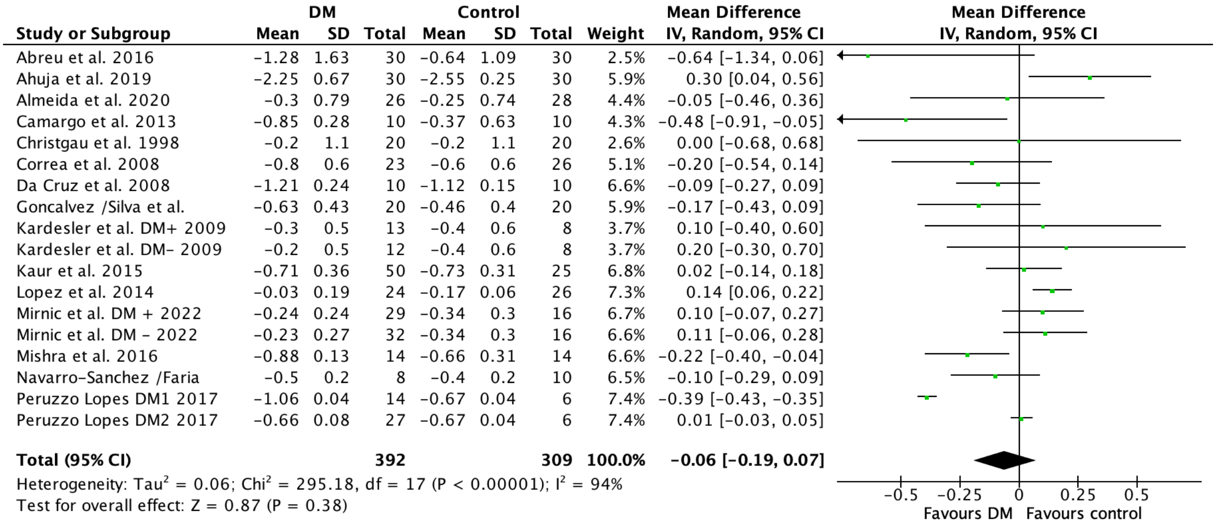

Online Appendix S7.7

Meta-analysis evaluating the effect of NSPT on DM compared to non-DM regarding GI at baseline

Study or Subgroup	DM Mean	DM SD	DM Total	Control Mean	Control SD	Control Total	Weight	Mean Difference IV, Random, 95% CI
Ahuja et al. 2019	2.05	0.4	30	1.83	0.3	30	7.9%	0.22 [0.04, 0.40]
Dag et al. DM + 2009	1.04	0.31	15	1.24	0.4	8	4.5%	−0.20 [−0.52, 0.12]
Dag et al. DM − 2009	1.32	0.4	15	1.24	0.4	8	4.1%	0.08 [−0.26, 0.42]
Dogan et al. 2016	2.29	0.4	20	2.34	0.34	20	6.5%	−0.05 [−0.28, 0.18]
Gayathri et al. 2019	2.03	1.32	30	1.93	0.3	30	2.5%	0.10 [−0.38, 0.58]
Gomathi et al. 2023	2.95	0.29	15	2.83	0.26	15	7.4%	0.12 [−0.08, 0.32]
Hungund et al. 2010	1.81	0.61	15	1.93	0.36	15	3.9%	−0.12 [−0.48, 0.24]
Kara et al. 2015	1.6	0.211	15	1.54	0.287	15	7.9%	0.06 [−0.12, 0.24]
Kaur et al. 2015	1.57	0.28	50	1.55	0.28	25	9.3%	0.02 [−0.11, 0.15]
Kudva et al. 2010	1.38	0.38	15	1.32	0.2	15	6.8%	0.06 [−0.16, 0.28]
Mirnic et al. DM + 2022	1.62	0.64	29	0.94	0.72	16	3.1%	0.68 [0.26, 1.10]
Mirnic et al. DM − 2022	1.59	0.58	32	0.94	0.72	16	3.3%	0.65 [0.24, 1.06]
Mishra et al. 2016	2.51	0.27	14	2.08	0.37	14	6.2%	0.43 [0.19, 0.67]
Pannicker et al. 2016	2.27	0.19	35	2.23	0.21	35	10.6%	0.04 [−0.05, 0.13]
Pragada et al. 2019	2.4	0.33	30	2.11	0.47	30	7.1%	0.29 [0.08, 0.50]
Sundaram et al. DM+ 2017	2.66	0.37	40	2.43	0.62	20	5.0%	0.23 [−0.06, 0.52]
Sundaram et al. DM− 2017	2.46	0.77	40	2.43	0.62	20	3.8%	0.03 [−0.33, 0.39]
**Total (95% CI)**			**440**			**332**	**100.0%**	**0.13 [0.05, 0.22]**

Heterogeneity: $Tau^2 = 0.02$; $Chi^2 = 37.23$, df = 16 (P = 0.002); $I^2 = 57\%$
Test for overall effect: Z = 3.02 (P = 0.003)

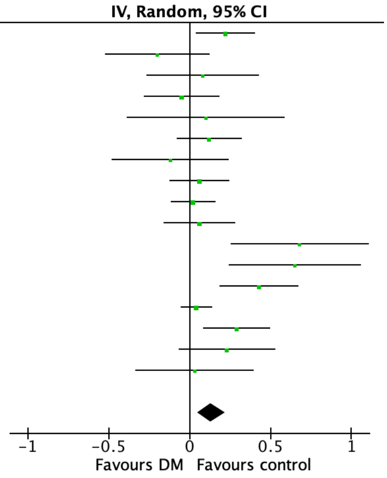

Online Appendix S7.8

Meta-analysis evaluating the effect of NSPT on DM compared to non-DM regarding GI post-NSPT

Study or Subgroup	DM Mean	DM SD	DM Total	Control Mean	Control SD	Control Total	Weight	Mean Difference IV, Random, 95% CI
Ahuja et al. 2019	0.85	0.4	30	0.53	0.3	30	5.6%	0.32 [0.14, 0.50]
Dag et al. DM + 2009	0.09	0.06	15	0.07	0.03	8	8.2%	0.02 [−0.02, 0.06]
Dag et al. DM − 2009	0.13	0.1	15	0.07	0.03	8	8.0%	0.06 [0.01, 0.11]
Dogan et al. 2016	0.76	0.34	20	0.81	0.3	20	5.2%	−0.05 [−0.25, 0.15]
Gayathri et al. 2019	1.71	0.33	30	1.27	0.23	30	6.4%	0.44 [0.30, 0.58]
Gomathi et al. 2023	1.48	0.13	15	1.31	0.18	15	7.0%	0.17 [0.06, 0.28]
Hungund et al. 2010	1.22	0.29	15	1.26	0.2	15	5.6%	−0.04 [−0.22, 0.14]
Kara et al. 2015	0.58	0.217	15	0.3	0.089	15	6.9%	0.28 [0.16, 0.40]
Kaur et al. 2015	0.64	0.26	50	0.42	0.19	25	7.2%	0.22 [0.12, 0.32]
Kudva et al. 2010	0.73	0.32	15	0.58	0.14	15	5.7%	0.15 [−0.03, 0.33]
Mirnic et al. DM + 2022	0.86	0.37	29	0.37	0.45	16	4.2%	0.49 [0.23, 0.75]
Mirnic et al. DM − 2022	0.95	0.46	32	0.37	0.45	16	3.9%	0.58 [0.31, 0.85]
Mishra et al. 2016	1.63	0.24	14	1.41	0.27	14	5.4%	0.22 [0.03, 0.41]
Pannicker et al. 2016	1.43	0.26	35	1.46	0.29	35	6.7%	−0.03 [−0.16, 0.10]
Pragada et al. 2019	1.49	1.8	30	1.02	0.23	30	1.1%	0.47 [−0.18, 1.12]
Sundaram et al. DM+ 2017	0.67	0.21	40	0.69	0.26	20	6.6%	−0.02 [−0.15, 0.11]
Sundaram et al. DM− 2017	0.94	0.34	40	0.69	0.26	20	6.1%	0.25 [0.09, 0.41]
**Total (95% CI)**			**440**			**332**	**100.0%**	**0.18 [0.10, 0.25]**

Heterogeneity: $Tau^2 = 0.02$; $Chi^2 = 101.24$, df = 16 (P < 0.00001); $I^2 = 84\%$
Test for overall effect: Z = 4.64 (P < 0.00001)

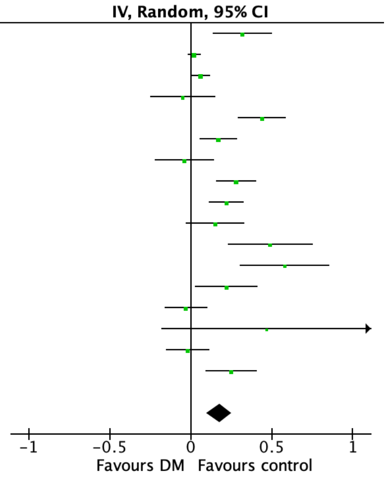

Online Appendix S7.9

Meta-analysis evaluating the effect of NSPT on DM compared to non-DM regarding GI on incremental scores

Study or Subgroup	DM Mean	DM SD	DM Total	Control Mean	Control SD	Control Total	Weight	Mean Difference IV, Random, 95% CI
Ahuja et al. 2019	−1.2	0.52	30	−1.3	0.2	30	15.1%	0.10 [−0.10, 0.30]
Hungund et al. 2010	−0.58	0.32	15	−0.66	0.16	15	16.3%	0.08 [−0.10, 0.26]
Kaur et al. 2015	−0.94	0.26	50	−1.13	0.4	25	16.8%	0.19 [0.02, 0.36]
Kudva et al. 2010	−0.65	0.27	15	−0.74	0.28	15	15.3%	0.09 [−0.11, 0.29]
Mirnic et al. DM + 2022	−0.76	0.43	29	−0.57	0.53	16	10.0%	−0.19 [−0.49, 0.11]
Mirnic et al. DM − 2022	−0.64	0.45	32	−0.57	0.53	16	10.0%	−0.07 [−0.37, 0.23]
Mishra et al. 2016	−0.88	0.13	14	−0.66	0.31	14	16.6%	−0.22 [−0.40, −0.04]
**Total (95% CI)**			**185**			**131**	**100.0%**	**0.01 [−0.11, 0.13]**

Heterogeneity: $Tau^2 = 0.02$; $Chi^2 = 14.57$, df = 6 (P = 0.02); $I^2 = 59\%$
Test for overall effect: Z = 0.18 (P = 0.86)

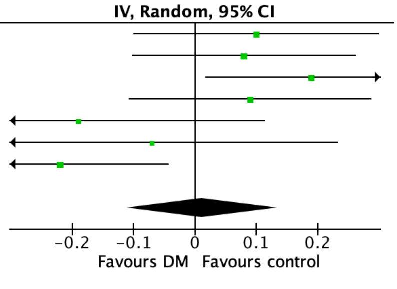

Online Appendix S7.10

Meta-analysis evaluating the effect of NSPT on DM compared to non-DM regarding BOP at baseline

Study or Subgroup	DM Mean	DM SD	DM Total	Control Mean	Control SD	Control Total	Weight	Mean Difference IV, Random, 95% CI
Abreu et al. 2016	38.24	19.29	30	63	23.87	30	7.1%	−24.76 [−35.74, −13.78]
Almeida et al. 2020	45.5	22.4	26	40.9	18.9	28	7.1%	4.60 [−6.50, 15.70]
Christgau et al. 1998	64.9	22.7	20	72.1	16.5	20	6.9%	−7.20 [−19.50, 5.10]
Correa et al. 2008	90.4	10.5	23	75.6	15.2	26	7.7%	14.80 [7.55, 22.05]
Goncalvez /Silva et al.	90.91	9.87	20	76.13	16.15	20	7.5%	14.78 [6.48, 23.08]
Kara et al. 2015	75.73	12.182	15	73	15.651	15	7.3%	2.73 [−7.31, 12.77]
Kardesler et al. DM+ 2009	81.5	7.9	13	78.8	16.5	8	6.9%	2.70 [−9.51, 14.91]
Kardesler et al. DM− 2009	86	12.3	12	78.8	16.5	8	6.7%	7.20 [−6.19, 20.59]
Kaur et al. 2015	73.68	14.63	50	70.58	16.62	23	7.6%	3.10 [−4.81, 11.01]
Lopez et al. 2014	57.4	15.1	26	47.2	12.8	26	7.6%	10.20 [2.59, 17.81]
Navarro-Sanchez /Faria	91	14.8	8	83.9	17.2	10	6.5%	7.10 [−7.69, 21.89]
Peruzzo Lopes DM1 2017	37.77	3.38	14	63.05	2.78	6	8.1%	−25.28 [−28.12, −22.44]
Peruzzo Lopes DM2 2017	51.36	3.22	27	63.05	2.78	6	8.1%	−11.69 [−14.22, −9.16]
Sonoki et al. 2006	46	21.9	5	31.5	17.8	6	4.9%	14.50 [−9.40, 38.40]
**Total (95% CI)**			**289**			**232**	**100.0%**	**0.38 [−8.01, 8.77]**

Heterogeneity: $Tau^2 = 225.49$; $Chi^2 = 268.17$, df = 13 (P < 0.00001); $I^2 = 95\%$
Test for overall effect: Z = 0.09 (P = 0.93)

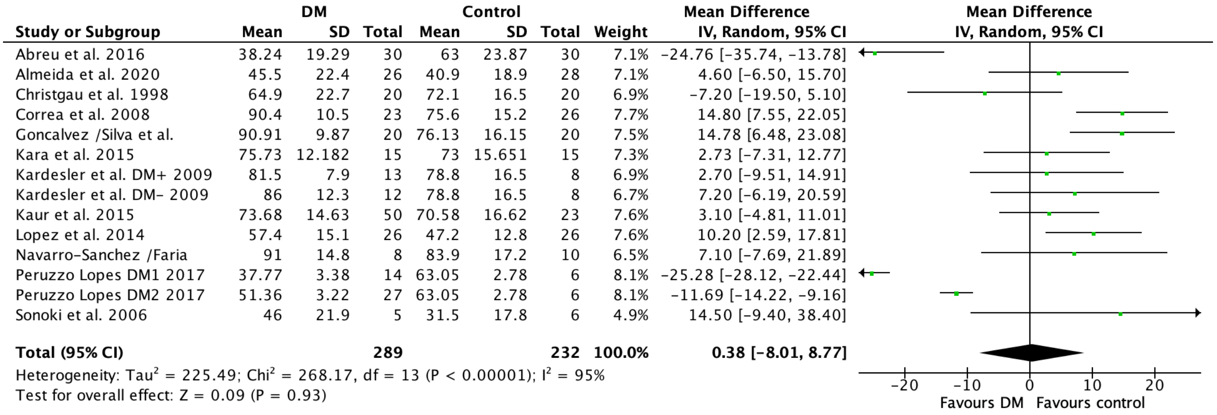

Online Appendix S7.11

Meta-analysis evaluating the effect of NSPT on DM compared to non-DM regarding BOP post-NSPT

Study or Subgroup	DM Mean	DM SD	DM Total	Control Mean	Control SD	Control Total	Weight	Mean Difference IV, Random, 95% CI
Abreu et al. 2016	13.31	15.12	30	15.11	20.27	30	5.9%	−1.80 [−10.85, 7.25]
Almeida et al. 2020	29.1	15.6	26	22.4	13.3	28	6.7%	6.70 [−1.06, 14.46]
Christgau et al. 1998	29.5	17.5	20	24.8	10.1	20	6.0%	4.70 [−4.16, 13.56]
Correa et al. 2008	28.9	16.5	23	16.3	10.3	26	6.7%	12.60 [4.78, 20.42]
Goncalvez /Silva et al.	28.21	15.88	20	15.79	10.72	20	6.3%	12.42 [4.02, 20.82]
Kara et al. 2015	24.24	11.578	15	15.43	5.719	15	7.5%	8.81 [2.28, 15.34]
Kardesler et al. DM+ 2009	25.4	6.9	13	28.1	9.2	8	7.0%	−2.70 [−10.10, 4.70]
Kardesler et al. DM− 2009	40.6	8.2	12	28.1	9.2	8	6.6%	12.50 [4.62, 20.38]
Kaur et al. 2015	38.96	11.62	50	29.1	8.56	25	8.9%	9.86 [5.21, 14.51]
Lopez et al. 2014	32.5	13.4	24	33.2	10	26	7.5%	−0.70 [−7.30, 5.90]
Navarro-Sanchez /Faria	11.4	9.7	8	12.2	5.9	10	6.8%	−0.80 [−8.45, 6.85]
Peruzzo Lopes DM1 2017	5.67	0.75	14	8.23	0.35	6	10.8%	−2.56 [−3.04, −2.08]
Peruzzo Lopes DM2 2017	12.73	1.2	27	8.23	0.35	6	10.8%	4.50 [3.97, 5.03]
Sonoki et al. 2006	17.3	17.5	5	12.8	12.2	6	2.5%	4.50 [−13.68, 22.68]
**Total (95% CI)**			**287**			**234**	**100.0%**	**4.64 [1.39, 7.89]**

Heterogeneity: $Tau^2 = 25.31$; $Chi^2 = 421.49$, df = 13 (P < 0.00001); $I^2 = 97\%$
Test for overall effect: Z = 2.80 (P = 0.005)

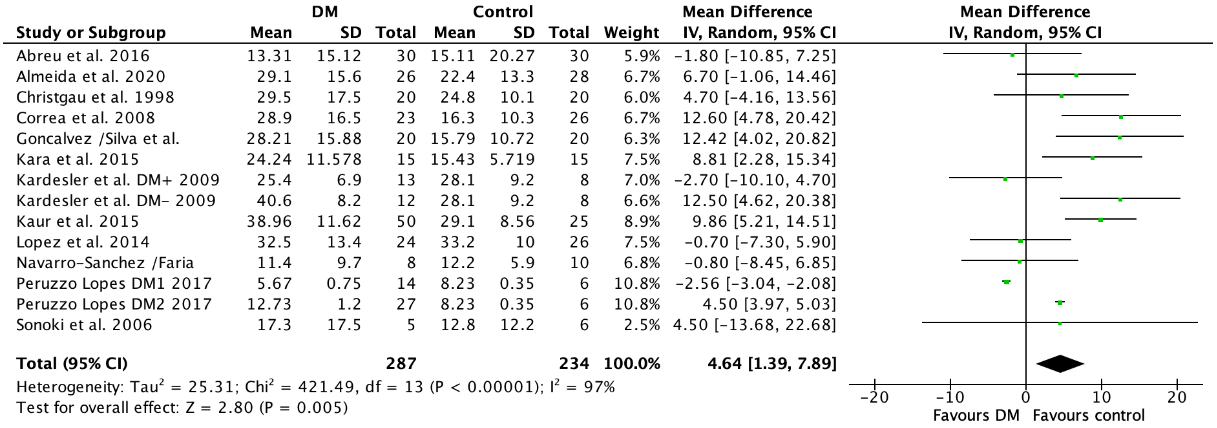

Online Appendix S7.12

Meta-analysis evaluating the effect of NSPT on DM compared to non-DM regarding BOP on incremental scores

Study or Subgroup	DM Mean	DM SD	DM Total	Control Mean	Control SD	Control Total	Weight	Mean Difference IV, Random, 95% CI
Abreu et al. 2016	−27.58	21.9	30	−46.3	23.62	30	7.5%	18.72 [7.19, 30.25]
Almeida et al. 2020	−16.5	16.2	26	−18.5	19.8	28	7.9%	2.00 [−7.62, 11.62]
Christgau et al. 1998	−35.4	20.3	20	−47.3	16.4	20	7.5%	11.90 [0.46, 23.34]
Correa et al. 2008	−68.5	15.9	23	−59.3	15.7	26	8.0%	−9.20 [−18.07, −0.33]
Goncalvez /Silva et al.	−69.21	15.64	20	−78.67	13.97	20	7.9%	9.46 [0.27, 18.65]
Kardesler et al. DM+ 2009	−56.1	9.8	13	−50.7	18.24	8	7.1%	−5.40 [−19.12, 8.32]
Kardesler et al. DM− 2009	−45.4	11.98	12	−50.7	18.24	8	7.0%	5.30 [−9.04, 19.64]
Kaur et al. 2015	−34.71	14.78	50	−41.48	15.27	25	8.2%	6.77 [−0.48, 14.02]
Lopez et al. 2014	−24.9	1.7	24	−14	2.8	26	8.8%	−10.90 [−12.17, −9.63]
Navarro-Sanchez /Faria	−79.6	16.7	8	−71.8	16.4	10	6.8%	−7.80 [−23.20, 7.60]
Peruzzo Lopes DM1 2017	−32.1	0.51	14	−54.82	0.51	6	8.8%	22.72 [22.23, 23.21]
Peruzzo Lopes DM2 2017	−38.63	2	27	−54.82	0.51	6	8.8%	16.19 [15.33, 17.05]
Sonoki et al. 2006	−28.7	21.4	5	−18.6	11.3	6	5.7%	−10.10 [−30.92, 10.72]
**Total (95% CI)**			**272**			**219**	**100.0%**	**4.39 [−3.94, 12.71]**

Heterogeneity: $Tau^2 = 205.10$; $Chi^2 = 2446.86$, df = 12 (P < 0.00001); $I^2 = 100\%$
Test for overall effect: Z = 1.03 (P = 0.30)

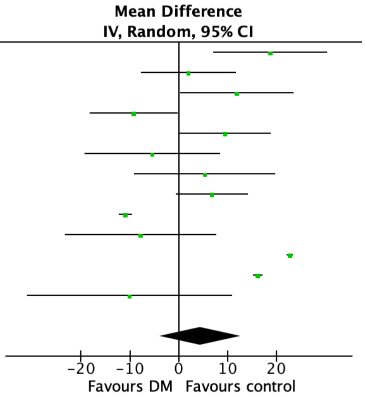

Online Appendix S7.13

Meta-analysis evaluating the effect of NSPT on DM compared to non-DM regarding GBI at baseline

	DM			Control				Mean Difference
Study or Subgroup	Mean	SD	Total	Mean	SD	Total	Weight	IV, Random, 95% CI
Buzinin et al. 2014	36.7	22.2	20	34.04	20.56	21	10.6%	2.66 [−10.45, 15.77]
Camargo et al. 2013	61.11	15.91	10	39.39	20.31	10	8.4%	21.72 [5.73, 37.71]
Correa et al. 2008	46.8	22	23	38.1	14.9	26	12.9%	8.70 [−1.96, 19.36]
Da Cruz et al. 2008	81.97	25.11	10	82.55	23.81	10	5.6%	−0.58 [−22.03, 20.87]
Dag et al. DM + 2009	28	12	15	37	18	8	9.9%	−9.00 [−22.87, 4.87]
Dag et al. DM − 2009	33	18	15	37	18	8	8.8%	−4.00 [−19.45, 11.45]
Dogan et al. 2016	70.29	8.35	20	75.83	10.07	20	18.6%	−5.54 [−11.27, 0.19]
Goncalvez /Silva et al.	47.3	23.2	20	38.4	16.5	20	11.1%	8.90 [−3.58, 21.38]
Hungund et al. 2010	85.92	13.3	15	86.66	13.54	15	14.1%	−0.74 [−10.34, 8.86]
**Total (95% CI)**			**148**			**138**	**100.0%**	**1.81 [−4.03, 7.64]**

Heterogeneity: $Tau^2 = 39.08$; $Chi^2 = 17.24$, df = 8 (P = 0.03); $I^2 = 54\%$
Test for overall effect: Z = 0.61 (P = 0.54)

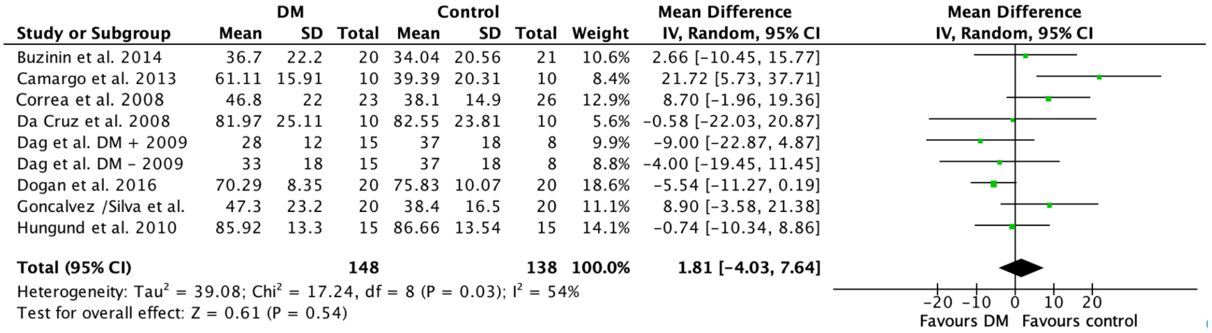

Online Appendix S7.14

Meta-analysis evaluating the effect of NSPT on DM compared to non-DM regarding GBI post-NSPT

	DM			Control				Mean Difference
Study or Subgroup	Mean	SD	Total	Mean	SD	Total	Weight	IV, Random, 95% CI
Buzinin et al. 2014	12.15	7.68	20	11.76	9.69	21	6.6%	0.39 [−4.95, 5.73]
Camargo et al. 2013	13.51	3.9	10	22.44	14.87	10	2.6%	−8.93 [−18.46, 0.60]
Correa et al. 2008	11.4	10.8	23	3.7	3	26	8.1%	7.70 [3.14, 12.26]
Da Cruz et al. 2008	10.72	3.97	10	9.3	2.95	10	12.6%	1.42 [−1.65, 4.49]
Dag et al. DM + 2009	4	1	15	3	2	8	19.3%	1.00 [−0.48, 2.48]
Dag et al. DM − 2009	4	2	15	3	2	8	18.3%	1.00 [−0.72, 2.72]
Dogan et al. 2016	9.24	1.89	20	8.91	1.68	20	20.8%	0.33 [−0.78, 1.44]
Goncalvez /Silva et al.	10.8	10.4	20	2.9	2.3	20	7.9%	7.90 [3.23, 12.57]
Hungund et al. 2010	54.66	12.11	15	57.24	8.24	15	3.9%	−2.58 [−9.99, 4.83]
**Total (95% CI)**			**148**			**138**	**100.0%**	**1.57 [−0.05, 3.18]**

Heterogeneity: $Tau^2 = 2.96$; $Chi^2 = 23.27$, df = 8 (P = 0.003); $I^2 = 66\%$
Test for overall effect: Z = 1.90 (P = 0.06)

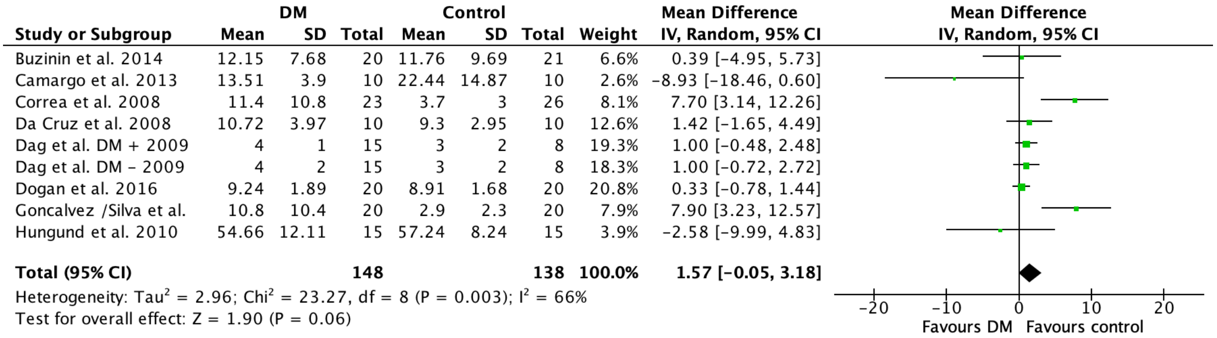

Online Appendix S7.15

Meta-analysis evaluating the effect of NSPT on DM compared to non-DM regarding GBI on incremental scores

	DM			Control				Mean Difference
Study or Subgroup	Mean	SD	Total	Mean	SD	Total	Weight	IV, Random, 95% CI
Camargo et al. 2013	−47.6	15.53	10	−16.95	19.27	10	24.2%	−30.65 [−45.99, −15.31]
Correa et al. 2008	−73.5	19.5	23	−34.5	14.6	26	25.9%	−39.00 [−48.75, −29.25]
Da Cruz et al. 2008	−71.85	21.23	10	−72.26	23.12	10	22.7%	0.41 [−19.04, 19.86]
Hungund et al. 2010	−31.25	1.19	20	−29.41	4.72	20	27.2%	−1.84 [−3.97, 0.29]
**Total (95% CI)**			**63**			**66**	**100.0%**	**−17.94 [−40.62, 4.73]**

Heterogeneity: $Tau^2 = 491.40$; $Chi^2 = 65.26$, df = 3 (P < 0.00001); $I^2 = 95\%$
Test for overall effect: Z = 1.55 (P = 0.12)

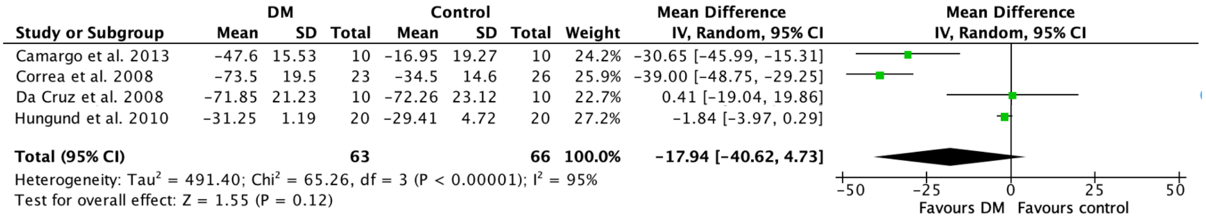

Online Appendix S7.16

Meta-analysis evaluating the effect of NSPT on DM compared to non-DM regarding REC at baseline

	DM			Control				Mean Difference	Mean Difference
**Study or Subgroup**	**Mean**	**SD**	**Total**	**Mean**	**SD**	**Total**	**Weight**	**IV, Random, 95% CI**	**IV, Random, 95% CI**
Camargo et al. 2013	1.68	0.72	10	1.9	0.85	10	20.6%	−0.22 [−0.91, 0.47]	
Cirano et al. 2012	0.6	0.5	16	0.4	0.6	15	52.8%	0.20 [−0.19, 0.59]	
Da Cruz et al. 2008	2.86	0.69	10	2.17	0.9	10	19.9%	0.69 [−0.01, 1.39]	
Navarro-Sanchez /Faria	1.6	1.7	8	1.2	0.7	10	6.7%	0.40 [−0.86, 1.66]	
**Total (95% CI)**			**44**			**45**	**100.0%**	**0.22 [−0.11, 0.56]**	

Heterogeneity: $Tau^2 = 0.01$; $Chi^2 = 3.37$, df = 3 (P = 0.34); $I^2 = 11\%$
Test for overall effect: Z = 1.33 (P = 0.18)

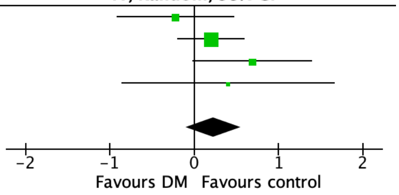

Online Appendix S7.17

Meta-analysis evaluating the effect of NSPT on DM compared to non-DM regarding REC post-NSPT

	DM			Control				Mean Difference	Mean Difference
**Study or Subgroup**	**Mean**	**SD**	**Total**	**Mean**	**SD**	**Total**	**Weight**	**IV, Random, 95% CI**	**IV, Random, 95% CI**
Camargo et al. 2013	1.51	0.62	10	2.02	0.41	10	30.7%	−0.51 [−0.97, −0.05]	
Cirano et al. 2012	1	0.7	16	1	0.7	15	29.7%	0.00 [−0.49, 0.49]	
Da Cruz et al. 2008	2.98	0.7	10	2.34	0.65	10	26.8%	0.64 [0.05, 1.23]	
Navarro-Sanchez /Faria	2.2	1.7	8	1.9	0.7	10	12.7%	0.30 [−0.96, 1.56]	
**Total (95% CI)**			**44**			**45**	**100.0%**	**0.05 [−0.49, 0.60]**	

Heterogeneity: $Tau^2 = 0.20$; $Chi^2 = 9.37$, df = 3 (P = 0.02); $I^2 = 68\%$
Test for overall effect: Z = 0.19 (P = 0.85)

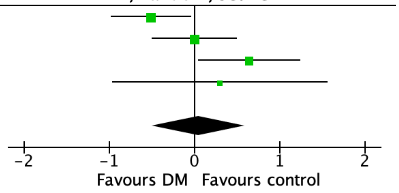

Online Appendix S7.18

Meta-analysis evaluating the effect of NSPT on DM compared to non-DM regarding REC on incremental scores

	DM			Control				Mean Difference	Mean Difference
**Study or Subgroup**	**Mean**	**SD**	**Total**	**Mean**	**SD**	**Total**	**Weight**	**IV, Fixed, 95% CI**	**IV, Fixed, 95% CI**
Camargo et al. 2013	−0.18	0.23	10	0.13	0.77	10	11.2%	−0.31 [−0.81, 0.19]	
Da Cruz et al. 2008	0.12	0.2	10	0.18	0.31	10	53.1%	−0.06 [−0.29, 0.17]	
Navarro-Sanchez /Faria	0.6	0.3	8	0.7	0.3	10	35.7%	−0.10 [−0.38, 0.18]	
**Total (95% CI)**			**28**			**30**	**100.0%**	**−0.10 [−0.27, 0.06]**	

Heterogeneity: $Chi^2 = 0.80$, df = 2 (P = 0.67); $I^2 = 0\%$
Test for overall effect: Z = 1.20 (P = 0.23)

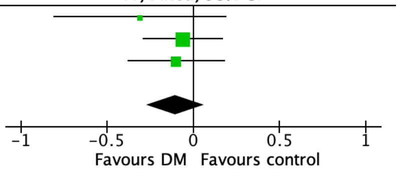

Online Appendix S7.19

Meta-analysis evaluating the effect of NSPT on DM compared to non-DM regarding plaque% at baseline

Study or Subgroup	DM Mean	DM SD	DM Total	healthy Mean	healthy SD	healthy Total	Weight	Mean Difference IV, Random, 95% CI
Abreu et al. 2016	72.96	14.3	30	83.01	12.01	30	6.4%	-10.05 [-16.73, -3.37]
Almeida et al. 2020	62.3	26.8	26	46.4	19.1	28	5.7%	15.90 [3.40, 28.40]
Buzinin et al. 2014	45.85	27.39	20	32.4	20.6	21	5.4%	13.45 [-1.44, 28.34]
Camargo et al. 2013	83.64	14.09	10	52.85	26.56	10	4.8%	30.79 [12.16, 49.42]
Christgau et al. 1998	78.7	19.7	20	79.7	16.3	20	5.9%	-1.00 [-12.21, 10.21]
Cirano et al. 2012	67.5	16.4	16	65.5	17.1	15	5.8%	2.00 [-9.81, 13.81]
Correa et al. 2008	84.5	11.1	23	65.4	14	26	6.4%	19.10 [12.06, 26.14]
Da Cruz et al. 2008	90.24	16.41	10	91.33	12.94	10	5.6%	-1.09 [-14.04, 11.86]
Goncalvez /Silva et al.	85.62	11.33	20	65.29	14.87	20	6.3%	20.33 [12.14, 28.52]
Hungund et al. 2010	92.93	12.85	15	93.46	10.63	15	6.2%	-0.53 [-8.97, 7.91]
Kardesler et al. DM+ 2009	97.4	5.4	13	95.5	9.4	8	6.4%	1.90 [-5.24, 9.04]
Kardesler et al. DM- 2009	99.7	1.2	12	95.5	9.4	8	6.4%	4.20 [-2.35, 10.75]
Lopez et al. 2014	89	13	26	81	18.7	26	6.2%	8.00 [-0.75, 16.75]
Navarro-Sanchez /Faria	84.7	25.8	8	83.9	16.6	10	4.5%	0.80 [-19.83, 21.43]
Peruzzo Lopes DM1 2017	36	3.1	14	56.63	2.98	6	6.7%	-20.63 [-23.51, -17.75]
Peruzzo Lopes DM2 2017	39.71	3.01	27	56.63	2.98	5	6.7%	-16.92 [-19.77, -14.07]
Tervonen et al. 1997	36.6	21.6	36	28.8	28.9	10	4.7%	7.80 [-11.45, 27.05]
**Total (95% CI)**			**326**			**268**	**100.0%**	**3.69 [-3.88, 11.25]**

Heterogeneity: $Tau^2 = 220.57$; $Chi^2 = 297.51$, df = 16 ($P < 0.00001$); $I^2 = 95\%$
Test for overall effect: Z = 0.96 (P = 0.34)

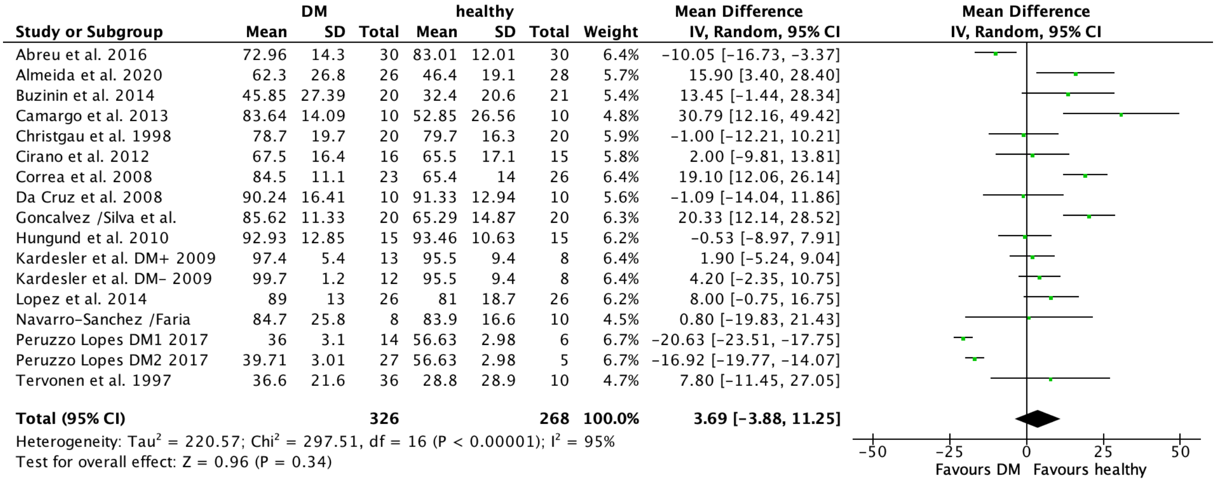

Online Appendix S7.20

Meta-analysis evaluating the effect of NSPT on DM compared to non-DM regarding plaque% post-NSPT

Study or Subgroup	DM Mean	DM SD	DM Total	healthy Mean	healthy SD	healthy Total	Weight	Mean Difference IV, Random, 95% CI
Abreu et al. 2016	19.07	11.78	30	16.59	24.27	30	5.8%	2.48 [-7.17, 12.13]
Almeida et al. 2020	36.4	15.8	26	23.4	18.6	28	5.9%	13.00 [3.82, 22.18]
Buzinin et al. 2014	10.8	10.81	20	9.8	5.86	21	6.3%	1.00 [-4.36, 6.36]
Camargo et al. 2013	29.39	18.3	10	30.35	13.95	10	5.2%	-0.96 [-15.22, 13.30]
Christgau et al. 1998	47.7	25.1	20	42.2	18.1	20	5.3%	5.50 [-8.06, 19.06]
Cirano et al. 2012	17.6	10.5	16	19.1	11.4	15	6.1%	-1.50 [-9.23, 6.23]
Correa et al. 2008	18.2	13.8	23	8.6	3.4	26	6.3%	9.60 [3.81, 15.39]
Da Cruz et al. 2008	7.21	2.98	10	6.01	2.66	10	6.5%	1.20 [-1.28, 3.68]
Goncalvez /Silva et al.	17.88	14.23	20	8.06	2.79	20	6.2%	9.82 [3.46, 16.18]
Hungund et al. 2010	64.53	16.72	15	70.66	17.26	15	5.5%	-6.13 [-18.29, 6.03]
Kardesler et al. DM+ 2009	35.6	8.7	13	39.5	13.5	8	5.7%	-3.90 [-14.38, 6.58]
Kardesler et al. DM- 2009	44.2	6.3	12	39.5	13.5	8	5.8%	4.70 [-5.31, 14.71]
Lopez et al. 2014	54	18	24	53	18.6	26	5.8%	1.00 [-9.15, 11.15]
Navarro-Sanchez /Faria	11.1	12.2	8	7.9	9.7	10	5.7%	3.20 [-7.17, 13.57]
Peruzzo Lopes DM1 2017	4.87	0.55	14	31.52	3.49	6	6.5%	-26.65 [-29.46, -23.84]
Peruzzo Lopes DM2 2017	12.26	1.59	27	31.52	3.49	5	6.4%	-19.26 [-22.38, -16.14]
Tervonen et al. 1997	27.8	20.5	35	25.4	21.8	10	5.1%	2.40 [-12.72, 17.52]
**Total (95% CI)**			**323**			**268**	**100.0%**	**-0.48 [-7.65, 6.70]**

Heterogeneity: $Tau^2 = 205.34$; $Chi^2 = 397.08$, df = 16 ($P < 0.00001$); $I^2 = 96\%$
Test for overall effect: Z = 0.13 (P = 0.90)

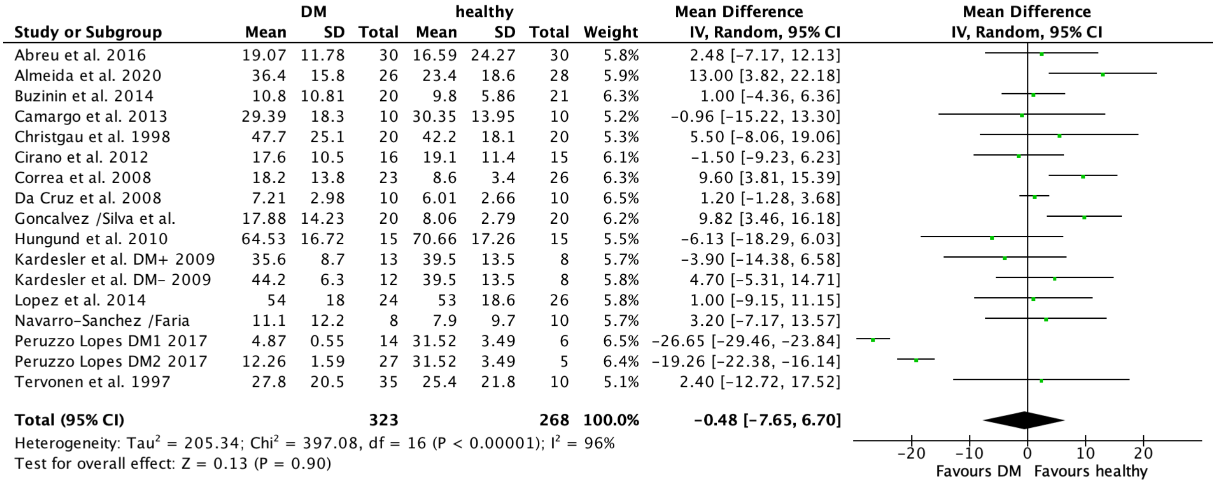

Online Appendix S7.21

Meta-analysis evaluating the effect of NSPT on DM compared to non-DM regarding plaque% on incremental scores.

Study or Subgroup	DM Mean	DM SD	DM Total	healthy Mean	healthy SD	healthy Total	Weight	Mean Difference IV, Random, 95% CI
Abreu et al. 2016	-54	21.27	30	-73.97	20.36	30	6.1%	19.97 [9.43, 30.51]
Almeida et al. 2020	-25.9	22.4	26	-23	23.8	28	5.1%	-2.90 [-15.22, 9.42]
Camargo et al. 2013	-54.25	13.02	10	-22.5	35.56	10	2.0%	-31.75 [-55.22, -8.28]
Christgau et al. 1998	-31.1	19.6	20	-37.5	18.7	20	5.3%	6.40 [-5.47, 18.27]
Correa et al. 2008	-79.1	13.9	23	-56.8	13.6	26	7.9%	-22.30 [-30.02, -14.58]
Da Cruz et al. 2008	-83.03	16.03	10	-85.32	13.44	10	4.8%	2.29 [-10.68, 15.26]
Goncalvez /Silva et al.	-79.79	14.05	20	-87.14	4.79	20	8.8%	7.35 [0.84, 13.86]
Hungund et al. 2010	-28.04	3.87	15	-22.8	6.63	15	10.6%	-5.24 [-9.12, -1.36]
Kardesler et al. DM+ 2009	-61.78	9.69	13	-56.1	13.96	8	5.8%	-5.68 [-16.69, 5.33]
Kardesler et al. DM- 2009	-55.44	6.86	12	-56.1	13.96	8	6.1%	0.66 [-9.76, 11.08]
Lopez et al. 2014	-35	5	24	-28	0.1	26	11.7%	-7.00 [-9.00, -5.00]
Navarro-Sanchez /Faria	-73.6	23.1	8	-76	14	10	3.0%	2.40 [-15.81, 20.61]
Peruzzo Lopes DM1 2017	-31.13	0.6	14	-25.11	3	6	11.5%	-6.02 [-8.44, -3.60]
Peruzzo Lopes DM2 2017	-27.51	2.02	27	-25.11	3	5	11.3%	-2.40 [-5.14, 0.34]
**Total (95% CI)**			**252**			**222**	**100.0%**	**-2.80 [-6.47, 0.87]**

Heterogeneity: $Tau^2 = 29.04$; $Chi^2 = 74.34$, df = 13 ($P < 0.00001$); $I^2 = 83\%$
Test for overall effect: Z = 1.49 (P = 0.14)

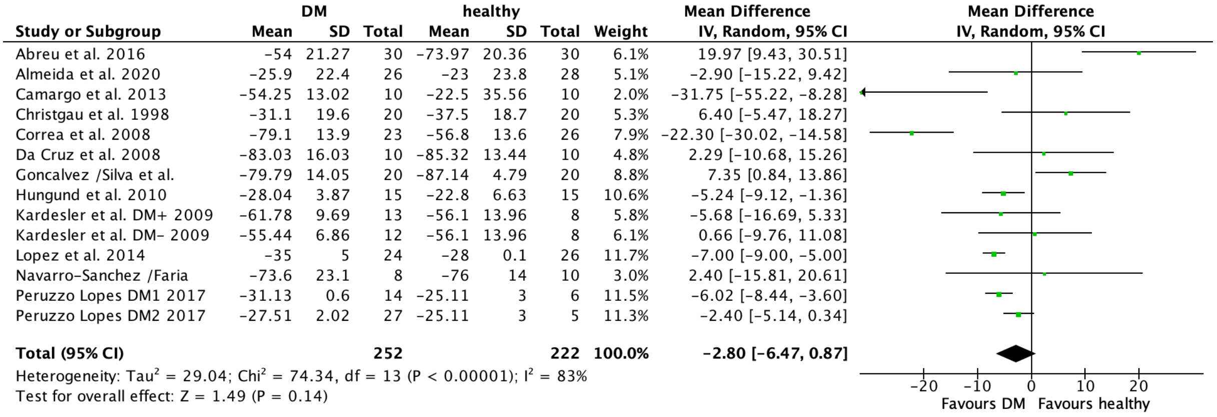

Online Appendix S7.22

Meta-analysis evaluating the effect of NSPT on DM compared to non-DM regarding PI (Silness-Loe) at baseline

Study or Subgroup	DM Mean	DM SD	DM Total	healthy Mean	healthy SD	healthy Total	Weight	Mean Difference IV, Random, 95% CI
Ahuja et al. 2019	2.45	0.33	30	2.09	0.4	30	8.1%	0.36 [0.17, 0.55]
Dag et al. DM + 2009	1.82	0.66	15	2.34	0.52	8	3.9%	−0.52 [−1.01, −0.03]
Dag et al. DM − 2009	2.05	0.68	15	2.34	0.52	8	3.8%	−0.29 [−0.79, 0.21]
Dogan et al. 2016	2.55	0.33	20	2.38	0.32	20	7.8%	0.17 [−0.03, 0.37]
Gomathi et al. 2023	2.69	0.41	15	2.78	0.24	15	7.2%	−0.09 [−0.33, 0.15]
Kara et al. 2015	1.65	0.3	15	1.83	0.034	15	8.5%	−0.18 [−0.33, −0.03]
Kaur et al. 2015	1.64	0.26	50	1.66	0.41	25	8.2%	−0.02 [−0.20, 0.16]
Kudva et al. 2010	1.51	0.44	15	1.63	0.46	15	5.9%	−0.12 [−0.44, 0.20]
Mirnic et al. DM + 2022	1.74	0.48	29	1.32	0.51	16	6.2%	0.42 [0.12, 0.72]
Mirnic et al. DM − 2022	1.88	0.37	32	1.32	0.51	16	6.5%	0.56 [0.28, 0.84]
Mishra et al. 2016	2.19	0.44	14	2.27	0.41	14	6.0%	−0.08 [−0.40, 0.24]
Pannicker et al. 2016	2.23	0.19	35	2.16	0.2	35	9.3%	0.07 [−0.02, 0.16]
Pragada et al. 2019	2.43	0.46	30	2.19	0.5	30	7.1%	0.24 [−0.00, 0.48]
Sundaram et al. DM+ 2017	2.11	0.44	40	2.13	0.69	20	5.8%	−0.02 [−0.35, 0.31]
Sundaram et al. DM− 2017	1.66	0.55	40	2.13	0.69	20	5.6%	−0.47 [−0.82, −0.12]
**Total (95% CI)**			**395**			**287**	**100.0%**	**0.03 [−0.09, 0.16]**

Heterogeneity: $Tau^2 = 0.04$; $Chi^2 = 60.99$, df = 14 ($P < 0.00001$); $I^2 = 77\%$
Test for overall effect: Z = 0.54 (P = 0.59)

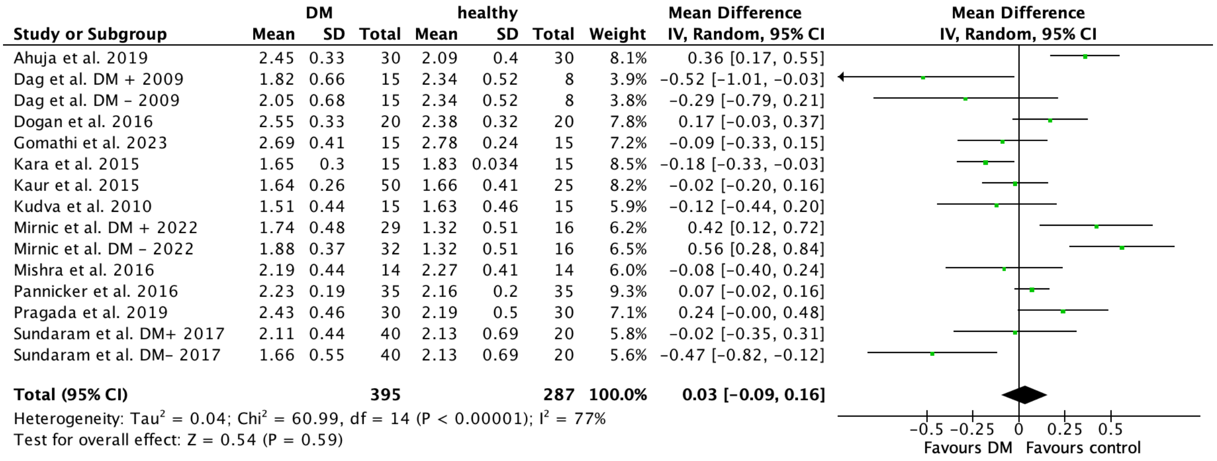

Online Appendix S7.23

Meta-analysis evaluating the effect of NSPT on DM compared to non-DM regarding PI (Silness-Loe) post-NSPT

Study or Subgroup	DM Mean	DM SD	DM Total	healthy Mean	healthy SD	healthy Total	Weight	Mean Difference IV, Random, 95% CI
Ahuja et al. 2019	0.34	0.2	30	1.03	0.41	30	6.8%	−0.69 [−0.85, −0.53]
Dag et al. DM + 2009	0.19	0.05	15	0.19	0.26	8	6.6%	0.00 [−0.18, 0.18]
Dag et al. DM − 2009	0.3	0.22	15	0.19	0.26	8	6.3%	0.11 [−0.10, 0.32]
Dogan et al. 2016	0.54	0.31	20	0.54	0.3	20	6.5%	0.00 [−0.19, 0.19]
Gomathi et al. 2023	1.86	0.12	15	1.4	0.31	15	6.7%	0.46 [0.29, 0.63]
Kara et al. 2015	0.46	0.193	15	0.32	0.174	15	7.1%	0.14 [0.01, 0.27]
Kaur et al. 2015	0.28	0.09	50	0.27	0.09	25	7.7%	0.01 [−0.03, 0.05]
Kudva et al. 2010	0.79	0.33	15	0.85	0.2	15	6.5%	−0.06 [−0.26, 0.14]
Mirnic et al. DM + 2022	1.14	0.46	29	0.66	0.49	16	5.4%	0.48 [0.19, 0.77]
Mirnic et al. DM − 2022	1.31	0.37	32	0.66	0.49	16	5.6%	0.65 [0.38, 0.92]
Mishra et al. 2016	1.29	0.2	14	1.47	0.29	14	6.6%	−0.18 [−0.36, 0.00]
Pannicker et al. 2016	1.48	0.23	35	1.45	0.3	35	7.2%	0.03 [−0.10, 0.16]
Pragada et al. 2019	1.31	0.48	30	1.02	0.018	30	6.7%	0.29 [0.12, 0.46]
Sundaram et al. DM+ 2017	0.94	0.08	40	0.8	0.24	20	7.3%	0.14 [0.03, 0.25]
Sundaram et al. DM− 2017	0.6	0.2	40	0.8	0.24	20	7.2%	−0.20 [−0.32, −0.08]
**Total (95% CI)**			**395**			**287**	**100.0%**	**0.07 [−0.06, 0.19]**

Heterogeneity: $Tau^2 = 0.05$; $Chi^2 = 165.06$, df = 14 ($P < 0.00001$); $I^2 = 92\%$
Test for overall effect: Z = 1.05 (P = 0.29)

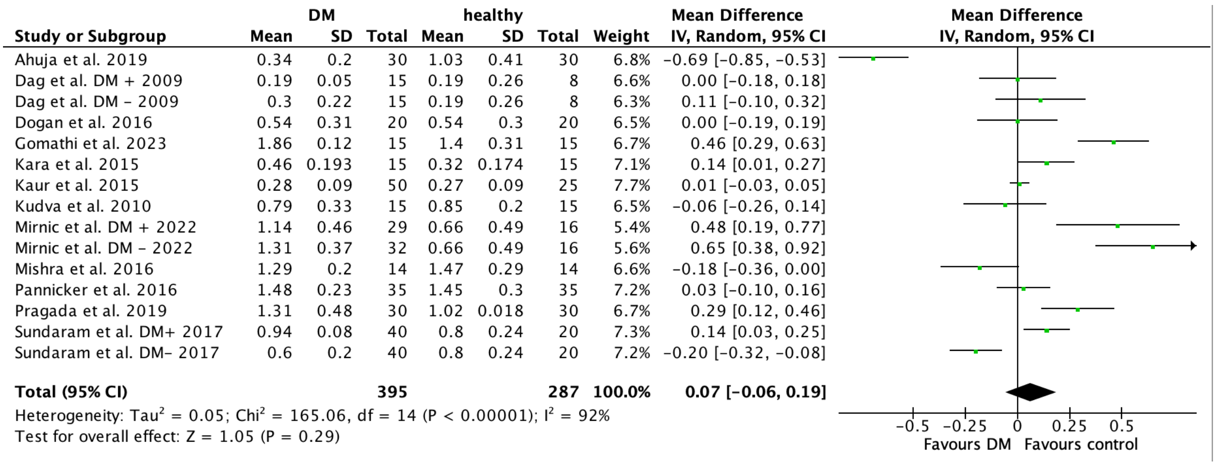

Online Appendix S7.24

Meta-analysis evaluating the effect of NSPT on DM compared to non-DM regarding PI (Silness-Loe) on incremental scores.

Study or Subgroup	DM Mean	DM SD	DM Total	healthy Mean	healthy SD	healthy Total	Weight	Mean Difference IV, Random, 95% CI
Ahuja et al. 2019	−2.11	0.45	30	−1.06	0.58	30	25.0%	−1.05 [−1.31, −0.79]
Kaur et al. 2015	−1.36	0.26	50	−1.39	0.37	25	26.1%	0.03 [−0.13, 0.19]
Kudva et al. 2010	−0.72	0.37	15	−0.88	0.56	15	24.0%	0.16 [−0.18, 0.50]
Mishra et al. 2016	−0.98	0.4	14	−0.72	0.31	14	25.0%	−0.26 [−0.53, 0.01]
**Total (95% CI)**			**109**			**84**	**100.0%**	**−0.28 [−0.80, 0.23]**

Heterogeneity: $Tau^2 = 0.26$; $Chi^2 = 52.40$, df = 3 ($P < 0.00001$); $I^2 = 94\%$
Test for overall effect: Z = 1.07 (P = 0.28)

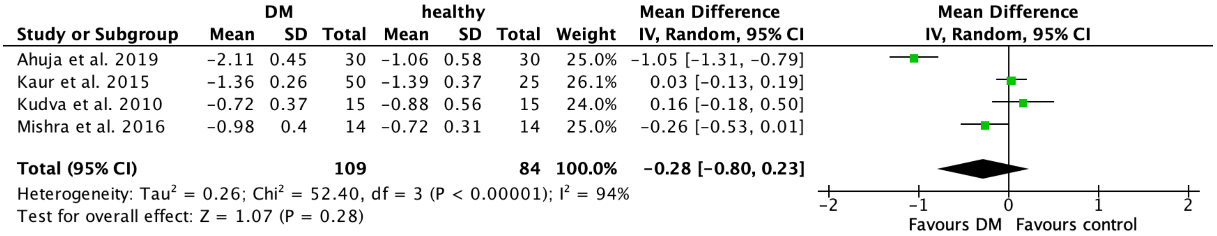

Online Appendix S7.25

Meta-analysis evaluating the effect of NSPT on good controlled DM compared to non-DM regarding PPD at baseline

	DM			Control				Mean Difference
Study or Subgroup	Mean	SD	Total	Mean	SD	Total	Weight	IV, Random, 95% CI
Abreu et al. 2016	3.16	0.39	30	2.97	0.63	30	8.7%	0.19 [−0.08, 0.46]
Ahuja et al. 2019	5.6	0.38	30	4.98	0.49	30	9.2%	0.62 [0.40, 0.84]
Dag et al. DM + 2009	2.67	0.45	15	2.61	0.38	8	7.7%	0.06 [−0.29, 0.41]
Dogan et al. 2016	4.11	0.34	20	4.09	0.37	20	9.2%	0.02 [−0.20, 0.24]
Gayathri et al. 2019	3.57	0.4	30	3.29	0.42	30	9.4%	0.28 [0.07, 0.49]
Gomathi et al. 2023	5.6	0.54	15	4.72	0.4	15	7.8%	0.88 [0.54, 1.22]
Kardesler et al. DM+ 2009	3.7	0.4	13	3.9	0.6	8	6.3%	−0.20 [−0.67, 0.27]
Kaur et al. DM + 2015	2.82	0.44	23	2.77	0.58	25	8.4%	0.05 [−0.24, 0.34]
Lopez et al. DM + 2014	3.17	0.56	13	3.1	0.5	26	7.6%	0.07 [−0.29, 0.43]
Mirnic et al. DM + 2022	2.09	0.51	29	2.38	0.6	31	8.5%	−0.29 [−0.57, −0.01]
Mishra et al. 2016	7.43	0.94	14	6.64	0.5	14	5.4%	0.79 [0.23, 1.35]
Pannicker et al. 2016	7.94	1.57	35	7.45	1.68	35	3.8%	0.49 [−0.27, 1.25]
Pragada et al. 2019	7.3	1.69	30	6.83	1.7	30	3.3%	0.47 [−0.39, 1.33]
Sundaram et al. DM+ 2017	4.04	1.02	40	3.84	1.33	20	4.5%	0.20 [−0.46, 0.86]
**Total (95% CI)**			**337**			**322**	**100.0%**	**0.23 [0.05, 0.42]**

Heterogeneity: $Tau^2 = 0.08$; $Chi^2 = 53.83$, df = 13 (P < 0.00001); $I^2 = 76\%$
Test for overall effect: Z = 2.46 (P = 0.01)

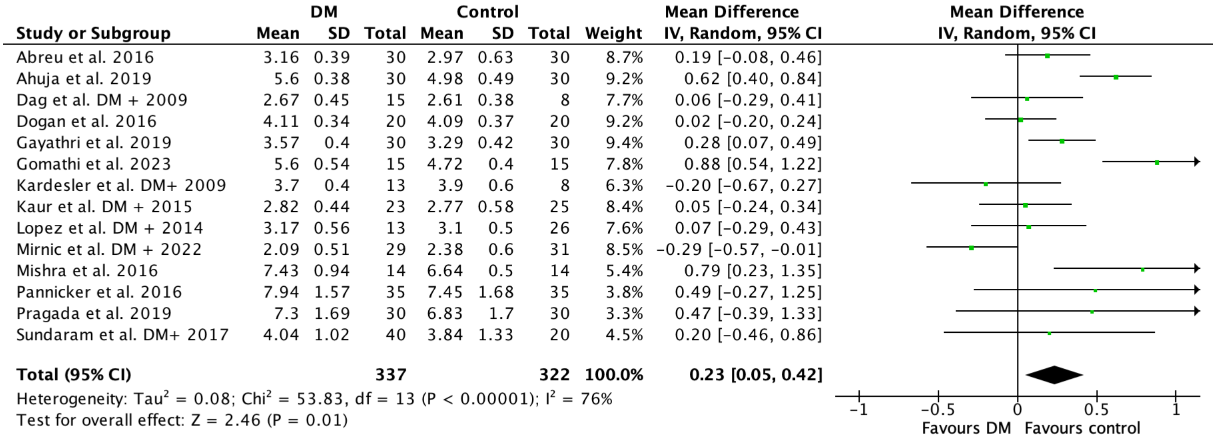

Online Appendix S7.26

Meta-analysis evaluating the effect of NSPT on good controlled DM compared to non-DM regarding PPD post-NSPT

	DM			Control				Mean Difference
Study or Subgroup	Mean	SD	Total	Mean	SD	Total	Weight	IV, Random, 95% CI
Abreu et al. 2016	2.54	0.74	30	2.2	0.79	30	5.8%	0.34 [−0.05, 0.73]
Ahuja et al. 2019	3.07	0.6	30	2.42	0.72	30	7.0%	0.65 [0.31, 0.99]
Dag et al. DM + 2009	2.3	0.43	15	2.36	0.68	8	3.8%	−0.06 [−0.58, 0.46]
Dogan et al. 2016	2.67	0.26	20	2.61	0.3	20	12.4%	0.06 [−0.11, 0.23]
Gayathri et al. 2019	3.44	0.42	30	3.16	0.46	30	10.5%	0.28 [0.06, 0.50]
Gomathi et al. 2023	2.97	0.2	15	3	0.16	15	14.2%	−0.03 [−0.16, 0.10]
Kardesler et al. DM+ 2009	2.8	0.4	13	2.7	0.3	8	7.9%	0.10 [−0.20, 0.40]
Kaur et al. DM + 2015	1.98	0.43	23	1.91	0.44	25	9.6%	0.07 [−0.18, 0.32]
Lopez et al. DM + 2014	2.86	0.56	12	2.75	0.57	26	5.9%	0.11 [−0.28, 0.50]
Mirnic et al. DM + 2022	1.98	0.53	29	2.05	0.52	31	9.0%	−0.07 [−0.34, 0.20]
Mishra et al. 2016	7	0.78	14	6.36	0.5	14	4.2%	0.64 [0.15, 1.13]
Pannicker et al. 2016	5.74	1.24	35	5.62	1.33	35	3.0%	0.12 [−0.48, 0.72]
Pragada et al. 2019	5.26	1.18	30	5.06	1.36	30	2.7%	0.20 [−0.44, 0.84]
Sundaram et al. DM+ 2017	1.88	0.54	40	1.87	1.06	20	4.1%	0.01 [−0.48, 0.50]
**Total (95% CI)**			**336**			**322**	**100.0%**	**0.15 [0.03, 0.26]**

Heterogeneity: $Tau^2 = 0.02$; $Chi^2 = 25.35$, df = 13 (P = 0.02); $I^2 = 49\%$
Test for overall effect: Z = 2.51 (P = 0.01)

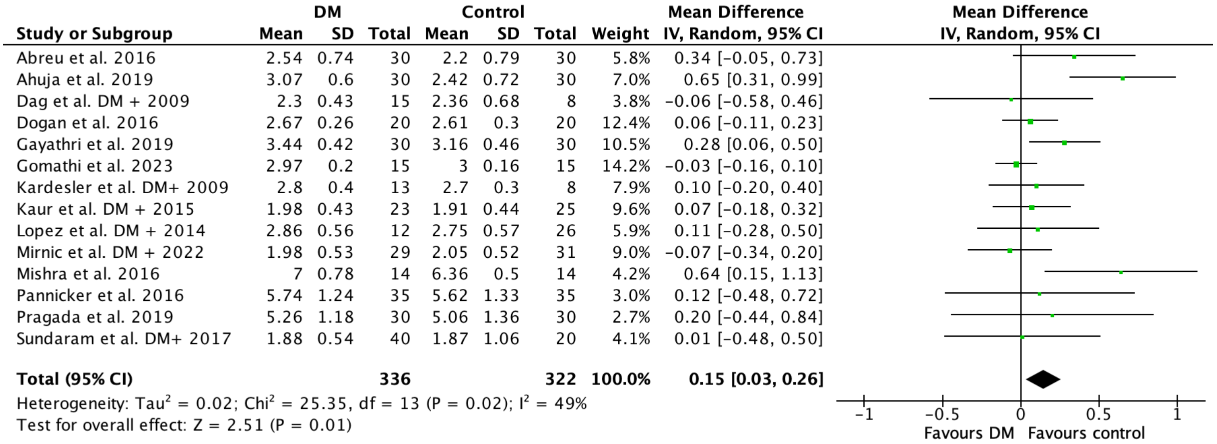

Online Appendix S7.27

Sub-analysis evaluating the effect of NSPT on good controlled DM compared to non-DM regarding PPD on incremental scores

	DM			Control				Mean Difference
Study or Subgroup	Mean	SD	Total	Mean	SD	Total	Weight	IV, Random, 95% CI
Abreu et al. 2016	−1.12	0.07	30	−0.74	0.74	30	9.8%	−0.38 [−0.65, −0.11]
Ahuja et al. 2019	−2.53	0.24	30	−2.57	0.26	30	18.4%	0.04 [−0.09, 0.17]
Kardesler et al. DM+ 2009	−0.93	0.37	13	−1.17	0.51	8	5.4%	0.24 [−0.17, 0.65]
Kaur et al. DM + 2015	−0.84	0.31	23	−0.86	0.29	25	15.2%	0.02 [−0.15, 0.19]
Lopez et al. DM + 2014	−0.31	0.01	12	−0.35	0.07	26	24.3%	0.04 [0.01, 0.07]
Mirnic et al. DM + 2022	−0.11	0.16	29	−0.34	0.23	31	20.4%	0.23 [0.13, 0.33]
Mishra et al. 2016	−0.43	0.51	14	−0.29	0.47	14	6.5%	−0.14 [−0.50, 0.22]
**Total (95% CI)**			**151**			**164**	**100.0%**	**0.03 [−0.07, 0.14]**

Heterogeneity: $Tau^2 = 0.01$; $Chi^2 = 25.07$, df = 6 (P = 0.0003); $I^2 = 76\%$
Test for overall effect: Z = 0.62 (P = 0.54)

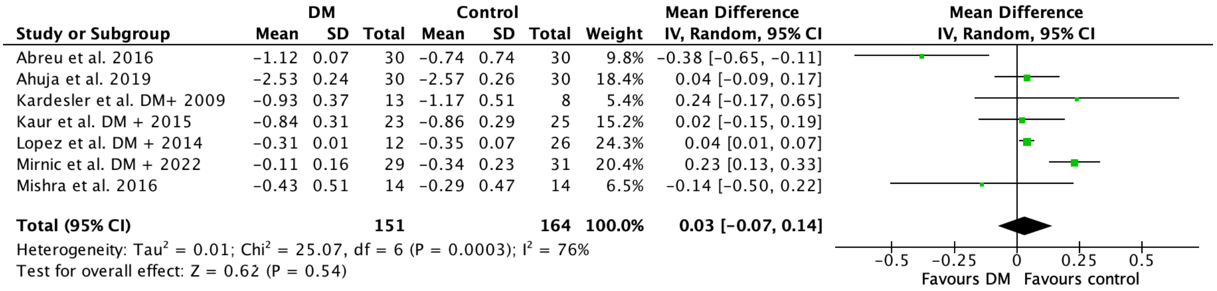

Online Appendix S7.28

Meta-analysis evaluating the effect of NSPT on uncontrolled controlled DM compared to non-DM regarding PPD at baseline

	DM			Control				Mean Difference
Study or Subgroup	Mean	SD	Total	Mean	SD	Total	Weight	IV, Random, 95% CI
Cirano et al. 2012	5.3	0.3	16	5.9	0.2	15	14.5%	−0.60 [−0.78, −0.42]
Dag et al. DM - 2009	2.84	0.65	15	2.61	0.38	8	12.6%	0.23 [−0.19, 0.65]
Goncalvez /Silva et al.	4.18	0.9	20	3.62	0.56	20	12.1%	0.56 [0.10, 1.02]
Kardesler et al. DM- 2009	4.1	1	12	3.9	0.6	8	9.7%	0.20 [−0.50, 0.90]
Kaur et al. DM - 2015	3.08	0.45	27	2.77	0.58	25	13.8%	0.31 [0.03, 0.59]
Lopez et al. DM - 2014	3.12	0.65	13	3.1	0.5	26	12.7%	0.02 [−0.38, 0.42]
Mirnic et al. DM - 2022	2.11	0.47	32	2.38	0.6	31	13.9%	−0.27 [−0.54, −0.00]
Sundaram et al. DM- 2017	4.8	0.57	40	3.84	1.33	20	10.6%	0.96 [0.35, 1.57]
**Total (95% CI)**			**175**			**153**	**100.0%**	**0.14 [−0.23, 0.51]**

Heterogeneity: $Tau^2 = 0.23$; $Chi^2 = 61.79$, df = 7 ($P < 0.00001$); $I^2 = 89\%$
Test for overall effect: Z = 0.74 (P = 0.46)

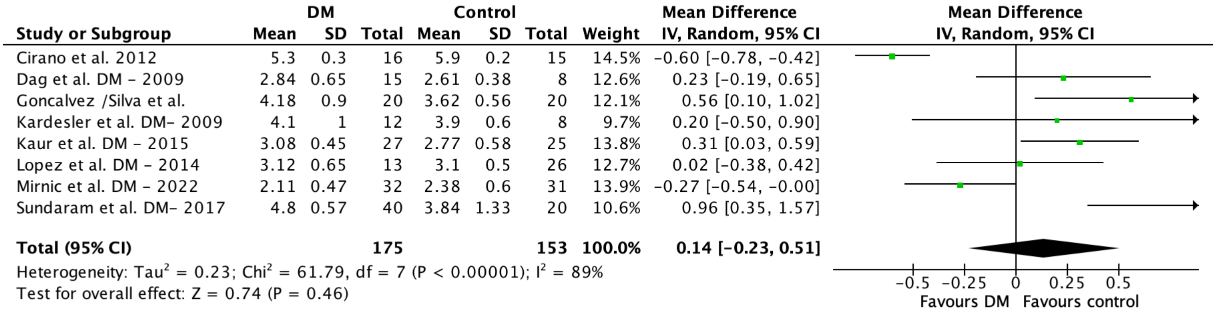

Online Appendix S7.29

Meta-analysis evaluating the effect of NSPT on uncontrolled controlled DM compared to non-DM regarding PPD post-NSPT

	DM			Control				Mean Difference
Study or Subgroup	Mean	SD	Total	Mean	SD	Total	Weight	IV, Random, 95% CI
Cirano et al. 2012	3	0.5	16	3.2	0.5	15	12.1%	−0.20 [−0.55, 0.15]
Dag et al. DM - 2009	2.37	0.59	15	2.36	0.68	8	6.6%	0.01 [−0.55, 0.57]
Goncalvez /Silva et al.	2.76	0.41	20	2.44	0.3	20	18.0%	0.32 [0.10, 0.54]
Kardesler et al. DM- 2009	3.1	0.6	12	2.7	0.3	8	10.5%	0.40 [0.00, 0.80]
Kaur et al. DM - 2015	2.29	0.37	27	1.91	0.44	25	18.1%	0.38 [0.16, 0.60]
Lopez et al. DM - 2014	2.79	0.69	12	2.75	0.57	26	9.0%	0.04 [−0.41, 0.49]
Mirnic et al. DM - 2022	2.03	0.35	32	2.05	0.52	31	18.2%	−0.02 [−0.24, 0.20]
Sundaram et al. DM- 2017	1.99	0.71	40	1.87	1.06	20	7.5%	0.12 [−0.39, 0.63]
**Total (95% CI)**			**174**			**153**	**100.0%**	**0.15 [−0.01, 0.32]**

Heterogeneity: $Tau^2 = 0.03$; $Chi^2 = 14.25$, df = 7 ($P = 0.05$); $I^2 = 51\%$
Test for overall effect: Z = 1.83 (P = 0.07)

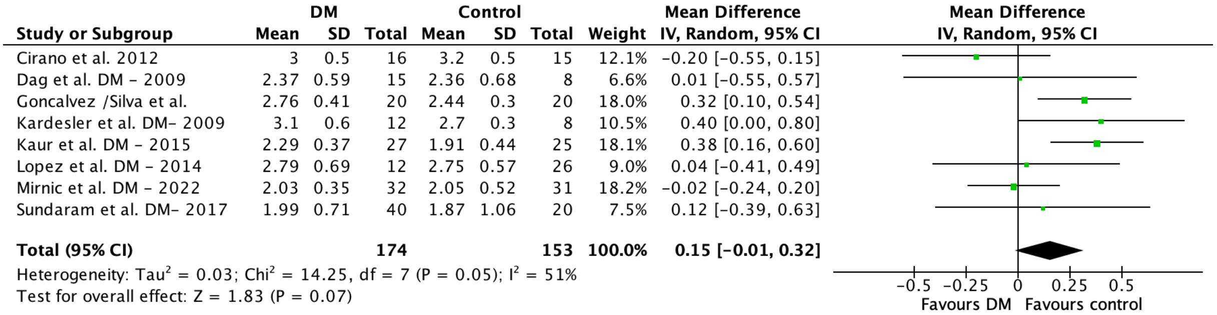

Online Appendix S7.30

Sub-analysis evaluating the effect of NSPT on uncontrolled DM compared to non-DM regarding PPD on incremental scores

	DM			Control				Mean Difference
Study or Subgroup	Mean	SD	Total	Mean	SD	Total	Weight	IV, Random, 95% CI
Goncalvez /Silva et al.	−1.42	0.61	20	−1.17	0.54	20	10.1%	−0.25 [−0.61, 0.11]
Kardesler et al. DM- 2009	−1.06	0.54	12	−1.17	0.51	8	6.8%	0.11 [−0.36, 0.58]
Kaur et al. DM - 2015	−0.79	0.26	27	−0.86	0.29	25	23.9%	0.07 [−0.08, 0.22]
Lopez et al. DM - 2014	−0.33	0.04	12	−0.35	0.07	26	32.9%	0.02 [−0.02, 0.06]
Mirnic et al. DM - 2022	−0.08	0.27	32	−0.34	0.23	31	26.3%	0.26 [0.14, 0.38]
**Total (95% CI)**			**103**			**110**	**100.0%**	**0.07 [−0.06, 0.21]**

Heterogeneity: $Tau^2 = 0.01$; $Chi^2 = 16.14$, df = 4 ($P = 0.003$); $I^2 = 75\%$
Test for overall effect: Z = 1.07 (P = 0.29)

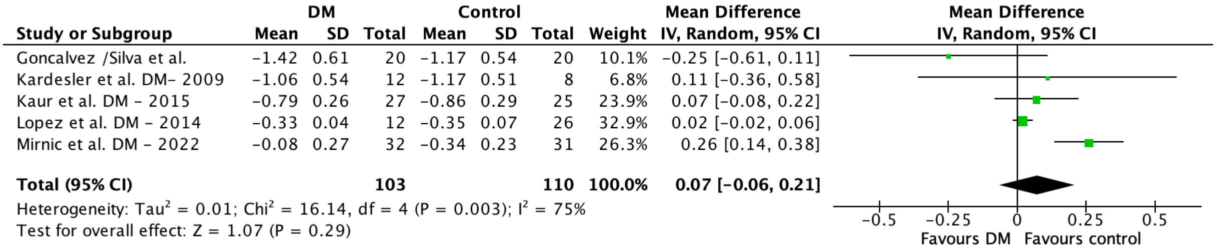

Online Appendix S7.31

Meta-analysis evaluating the effect of NSPT on good controlled DM compared to non-DM regarding CAL at baseline

	DM			Control				Mean Difference
Study or Subgroup	Mean	SD	Total	Mean	SD	Total	Weight	IV, Random, 95% CI
Abreu et al. 2016	4.15	0.9	30	3.77	0.71	30	7.7%	0.38 [−0.03, 0.79]
Ahuja et al. 2019	6.01	0.38	30	5.35	0.55	30	12.2%	0.66 [0.42, 0.90]
Dag et al. DM + 2009	4.25	0.82	15	4.31	0.59	8	4.9%	−0.06 [−0.64, 0.52]
Dogan et al. 2016	4.31	0.36	20	4.25	0.35	20	12.8%	0.06 [−0.16, 0.28]
Gayathri et al. 2019	3.65	0.45	30	3.6	0.42	30	12.8%	0.05 [−0.17, 0.27]
Gomathi et al. 2023	4.36	0.82	15	4.38	0.65	15	5.6%	−0.02 [−0.55, 0.51]
Kardesler et al. DM+ 2009	4.3	0.9	13	4.3	0.7	8	3.8%	0.00 [−0.69, 0.69]
Kaur et al. DM + 2015	3.3	0.52	23	3.14	0.53	25	10.5%	0.16 [−0.14, 0.46]
Lopez et al. DM + 2014	3.59	0.91	13	3.6	0.8	26	4.9%	−0.01 [−0.59, 0.57]
Mirnic et al. DM + 2022	2.81	0.98	29	2.32	1.39	31	4.7%	0.49 [−0.12, 1.10]
Mishra et al. 2016	2.51	0.27	14	2.08	0.37	14	12.2%	0.43 [0.19, 0.67]
Pannicker et al. 2016	11.97	2.43	35	9.82	1.31	35	0.0%	2.15 [1.24, 3.06]
Pragada et al. 2019	10.07	1.63	30	9.67	1.51	30	0.0%	0.40 [−0.40, 1.20]
Sundaram et al. DM+ 2017	4.36	0.67	40	4.13	0.79	20	7.9%	0.23 [−0.17, 0.63]
**Total (95% CI)**			**272**			**257**	**100.0%**	**0.23 [0.08, 0.38]**

Heterogeneity: $Tau^2 = 0.03$; $Chi^2 = 24.17$, df = 11 (P = 0.01); $I^2 = 54\%$

Test for overall effect: Z = 2.95 (P = 0.003)

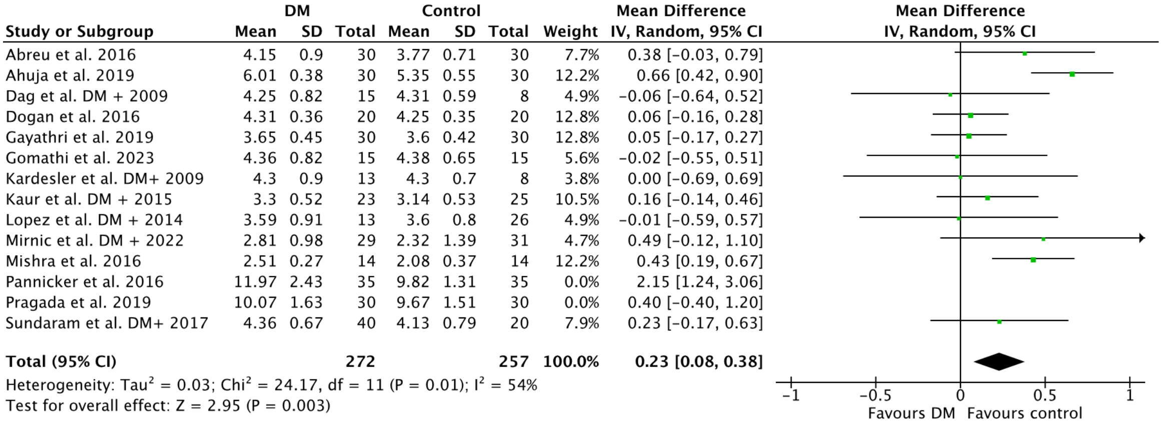

Online Appendix S7.32

Meta-analysis evaluating the effect of NSPT on good controlled DM compared to non-DM regarding CAL post-NSPT

	DM			Control				Mean Difference
Study or Subgroup	Mean	SD	Total	Mean	SD	Total	Weight	IV, Random, 95% CI
Abreu et al. 2016	3.58	0.92	30	3.1	1.12	30	5.5%	0.48 [−0.04, 1.00]
Ahuja et al. 2019	3.75	0.86	30	2.81	0.78	30	7.4%	0.94 [0.52, 1.36]
Dag et al. DM + 2009	3.03	0.79	15	2.91	0.56	8	5.0%	0.12 [−0.44, 0.68]
Dogan et al. 2016	3.09	0.43	20	2.88	0.38	20	11.8%	0.21 [−0.04, 0.46]
Gayathri et al. 2019	3.61	0.48	30	3.47	0.58	30	11.2%	0.14 [−0.13, 0.41]
Gomathi et al. 2023	3.03	0.19	15	3.05	0.15	15	16.0%	−0.02 [−0.14, 0.10]
Kardesler et al. DM+ 2009	3.9	0.9	13	3.9	0.7	8	3.6%	0.00 [−0.69, 0.69]
Kaur et al. DM + 2015	2.58	0.43	23	2.41	0.55	25	10.9%	0.17 [−0.11, 0.45]
Lopez et al. DM + 2014	3.44	0.86	12	3.43	0.74	26	4.9%	0.01 [−0.55, 0.57]
Mirnic et al. DM + 2022	2.56	1.01	29	1.98	1.31	31	4.6%	0.58 [−0.01, 1.17]
Mishra et al. 2016	1.63	0.24	14	1.41	0.27	14	13.8%	0.22 [0.03, 0.41]
Pannicker et al. 2016	9.85	2.39	35	7.48	1.22	35	0.0%	2.37 [1.48, 3.26]
Pragada et al. 2019	7.53	1.04	30	7.1	1.21	30	0.0%	0.43 [−0.14, 1.00]
Sundaram et al. DM+ 2017	3.06	1.05	40	2.97	0.99	20	5.2%	0.09 [−0.45, 0.63]
**Total (95% CI)**			**271**			**257**	**100.0%**	**0.22 [0.07, 0.37]**

Heterogeneity: $Tau^2 = 0.03$; $Chi^2 = 26.00$, df = 11 (P = 0.006); $I^2 = 58\%$

Test for overall effect: Z = 2.94 (P = 0.003)

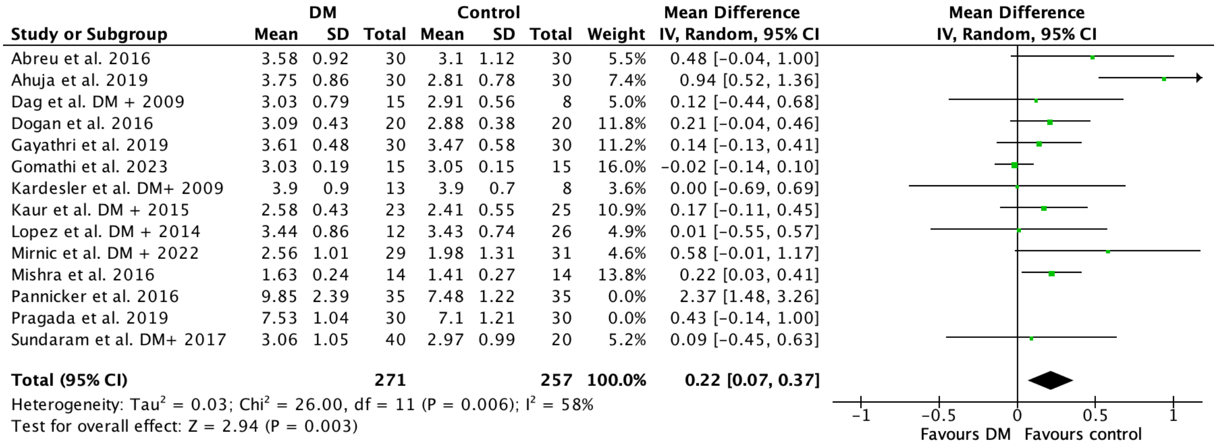

Online Appendix S7.33

Sub-analysis evaluating the effect of NSPT on good controlled DM compared to non-DM regarding CAL on incremental scores

	DM			Control				Mean Difference
Study or Subgroup	Mean	SD	Total	Mean	SD	Total	Weight	IV, Random, 95% CI
Abreu et al. 2016	−1.28	1.63	30	−0.64	1.09	30	1.9%	−0.64 [−1.34, 0.06]
Ahuja et al. 2019	−2.25	0.67	30	−2.55	0.25	30	11.1%	0.30 [0.04, 0.56]
Kardesler et al. DM+ 2009	−0.3	0.53	13	−0.36	0.62	8	3.4%	0.06 [−0.46, 0.58]
Kaur et al. DM + 2015	−0.72	0.34	23	−0.73	0.31	25	17.4%	0.01 [−0.17, 0.19]
Lopez et al. DM + 2014	−0.15	0.05	12	−0.17	0.06	26	42.2%	0.02 [−0.02, 0.06]
Mirnic et al. DM + 2022	−0.24	0.24	29	−0.34	0.3	31	24.0%	0.10 [−0.04, 0.24]
**Total (95% CI)**			**137**			**150**	**100.0%**	**0.06 [−0.04, 0.16]**

Heterogeneity: $Tau^2 = 0.01$; $Chi^2 = 9.12$, df = 5 (P = 0.10); $I^2 = 45\%$

Test for overall effect: Z = 1.14 (P = 0.25)

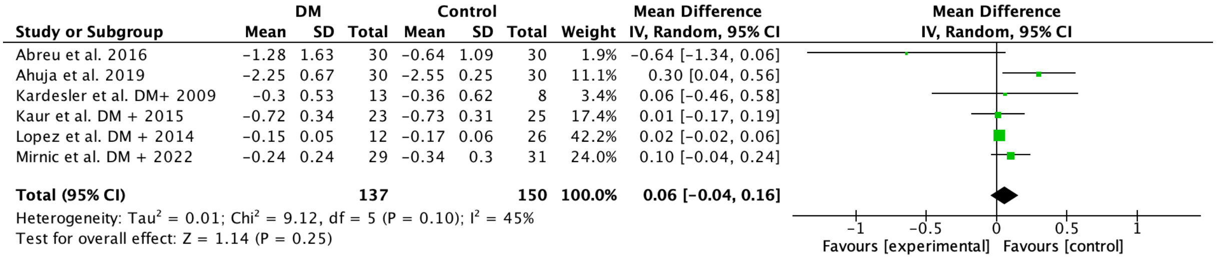

Online Appendix S7.34

Meta-analysis evaluating the effect of NSPT on uncontrolled DM compared to non-DM regarding CAL at baseline

	DM			Control				Mean Difference
Study or Subgroup	Mean	SD	Total	Mean	SD	Total	Weight	IV, Random, 95% CI
Cirano et al. 2012	6	0.7	16	6.3	0.7	15	13.9%	−0.30 [−0.79, 0.19]
Dag et al. DM - 2009	4.3	0.97	15	4.31	0.59	8	9.9%	−0.01 [−0.65, 0.63]
Goncalvez /Silva et al.	4.97	1.45	20	4.25	1.01	20	7.4%	0.72 [−0.05, 1.49]
Kardesler et al. DM- 2009	4.4	1.1	12	4.3	0.7	8	7.2%	0.10 [−0.69, 0.89]
Kaur et al. DM - 2015	3.59	0.52	27	3.14	0.53	25	22.8%	0.45 [0.16, 0.74]
Lopez et al. DM - 2014	3.73	0.9	13	3.6	0.8	26	11.4%	0.13 [−0.45, 0.71]
Mirnic et al. DM - 2022	2.62	1.36	32	2.32	1.39	31	9.1%	0.30 [−0.38, 0.98]
Sundaram et al. DM- 2017	4.03	0.47	40	4.13	0.79	20	18.4%	−0.10 [−0.48, 0.28]
**Total (95% CI)**			**175**			**153**	**100.0%**	**0.14 [−0.09, 0.38]**

Heterogeneity: $Tau^2 = 0.04$; $Chi^2 = 11.59$, df = 7 (P = 0.11); $I^2 = 40\%$
Test for overall effect: Z = 1.18 (P = 0.24)

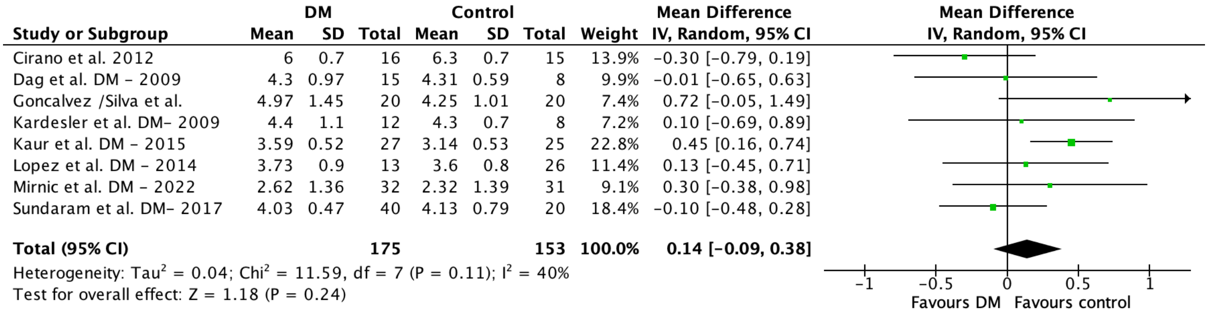

Online Appendix S7.35

Meta-analysis evaluating the effect of NSPT on uncontrolled DM compared to non-DM regarding CAL post-NSPT

	DM			Control				Mean Difference
Study or Subgroup	Mean	SD	Total	Mean	SD	Total	Weight	IV, Random, 95% CI
Cirano et al. 2012	3.9	0.8	16	4.2	0.9	15	10.9%	−0.30 [−0.90, 0.30]
Dag et al. DM - 2009	3.04	0.81	15	2.91	0.56	8	12.1%	0.13 [−0.43, 0.69]
Goncalvez /Silva et al.	4.34	1.29	20	3.97	0.83	20	9.0%	0.37 [−0.30, 1.04]
Kardesler et al. DM- 2009	4.2	0.9	12	3.9	0.7	8	8.3%	0.30 [−0.40, 1.00]
Kaur et al. DM - 2015	2.9	0.72	27	2.41	0.55	25	25.0%	0.49 [0.14, 0.84]
Lopez et al. DM - 2014	3.66	0.85	12	3.43	0.74	26	12.3%	0.23 [−0.33, 0.79]
Mirnic et al. DM - 2022	2.39	1.27	32	1.98	1.31	31	9.9%	0.41 [−0.23, 1.05]
Sundaram et al. DM- 2017	2.73	1.12	40	2.97	0.99	20	12.4%	−0.24 [−0.80, 0.32]
**Total (95% CI)**			**174**			**153**	**100.0%**	**0.20 [−0.01, 0.42]**

Heterogeneity: $Tau^2 = 0.02$; $Chi^2 = 8.52$, df = 7 (P = 0.29); $I^2 = 18\%$
Test for overall effect: Z = 1.84 (P = 0.07)

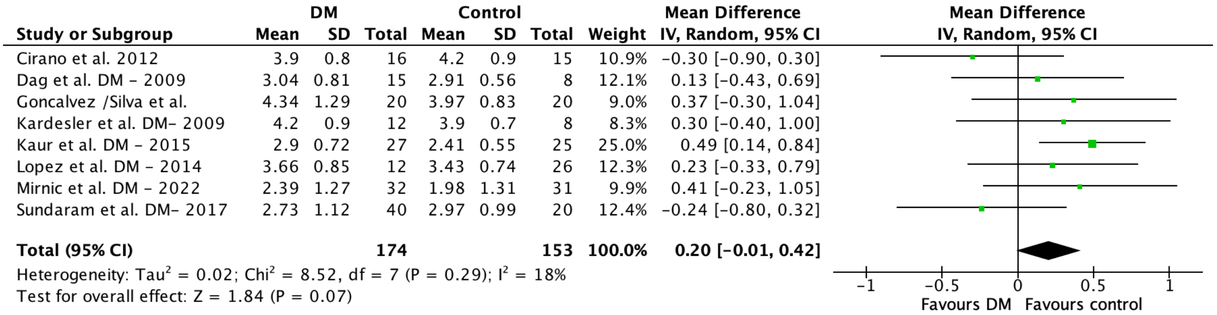

Online Appendix S7.36

Sub-analysis evaluating the effect of NSPT on uncontrolled DM compared to non-DM regarding CAL on incremental scores

	DM			Control				Mean Difference
Study or Subgroup	Mean	SD	Total	Mean	SD	Total	Weight	IV, Random, 95% CI
Goncalvez /Silva et al.	−0.63	0.43	20	−0.46	0.4	20	5.6%	−0.17 [−0.43, 0.09]
Kardesler et al. DM- 2009	−0.21	0.52	12	−0.36	0.62	8	1.4%	0.15 [−0.37, 0.67]
Kaur et al. DM - 2015	−0.71	0.38	27	−0.73	0.31	25	10.0%	0.02 [−0.17, 0.21]
Lopez et al. DM - 2014	−0.07	0.05	12	−0.17	0.06	26	66.7%	0.10 [0.06, 0.14]
Mirnic et al. DM - 2022	−0.23	0.27	32	−0.34	0.3	31	16.2%	0.11 [−0.03, 0.25]
**Total (95% CI)**			**103**			**110**	**100.0%**	**0.08 [0.02, 0.14]**

Heterogeneity: $Tau^2 = 0.00$; $Chi^2 = 4.83$, df = 4 (P = 0.30); $I^2 = 17\%$
Test for overall effect: Z = 2.46 (P = 0.01)

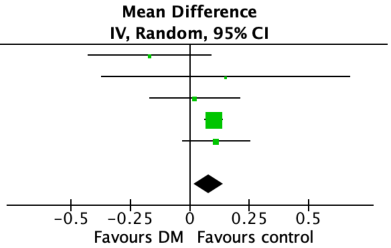

Online Appendix S7.37

Meta-analysis evaluating the effect of NSPT on DM compared to non-DM regarding deep pockets at baseline

Study or Subgroup	DM Mean	DM SD	DM Total	healthy Mean	healthy SD	healthy Total	Weight	Mean Difference IV, Random, 95% CI
**11.1.2 7 and greater**								
Cirano et al. 2012	7.9	0.6	16	8.2	1	15	38.8%	−0.30 [−0.89, 0.29]
Correa et al. 2008	13	12.9	23	8	6.7	26	8.6%	5.00 [−0.87, 10.87]
Kaur et al. 2015	12.24	3.52	50	10.82	4.09	25	29.2%	1.42 [−0.46, 3.30]
Navarro-Sanchez /Faria	4.1	3.5	8	1.2	1.5	10	23.4%	2.90 [0.30, 5.50]
**Subtotal (95% CI)**			**97**			**76**	**100.0%**	**1.41 [−0.53, 3.35]**
Heterogeneity: Tau² = 2.44; Chi² = 10.77, df = 3 (P = 0.01); I² = 72%								
Test for overall effect: Z = 1.42 (P = 0.16)								
**Total (95% CI)**			**97**			**76**	**100.0%**	**1.41 [−0.53, 3.35]**
Heterogeneity: Tau² = 2.44; Chi² = 10.77, df = 3 (P = 0.01); I² = 72%								
Test for overall effect: Z = 1.42 (P = 0.16)								
Test for subgroup differences: Not applicable								

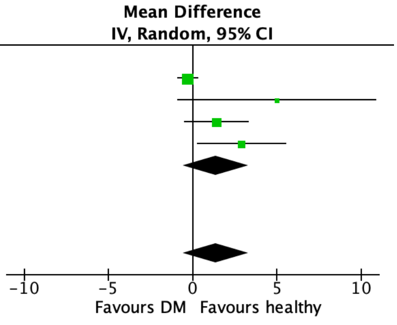

Online Appendix S7.38

Meta-analysis evaluating the effect of NSPT on DM compared to non-DM regarding deep pockets post-NSPT

Study or Subgroup	DM Mean	DM SD	DM Total	healthy Mean	healthy SD	healthy Total	Weight	Mean Difference IV, Random, 95% CI
**11.2.2 7 and greater**								
Cirano et al. 2012	4	1.2	16	4.2	1.3	15	26.5%	−0.20 [−1.08, 0.68]
Correa et al. 2008	1	1.7	23	0.3	0.9	26	27.1%	0.70 [−0.08, 1.48]
Kaur et al. 2015	5.94	2.91	50	2.91	1.3	25	26.0%	3.03 [2.08, 3.98]
Navarro-Sanchez /Faria	1.7	2.5	8	0.3	0.6	10	20.5%	1.40 [−0.37, 3.17]
**Subtotal (95% CI)**			**97**			**76**	**100.0%**	**1.21 [−0.25, 2.67]**
Heterogeneity: Tau² = 1.89; Chi² = 25.18, df = 3 (P < 0.0001); I² = 88%								
Test for overall effect: Z = 1.63 (P = 0.10)								
**Total (95% CI)**			**97**			**76**	**100.0%**	**1.21 [−0.25, 2.67]**
Heterogeneity: Tau² = 1.89; Chi² = 25.18, df = 3 (P < 0.0001); I² = 88%								
Test for overall effect: Z = 1.63 (P = 0.10)								
Test for subgroup differences: Not applicable								

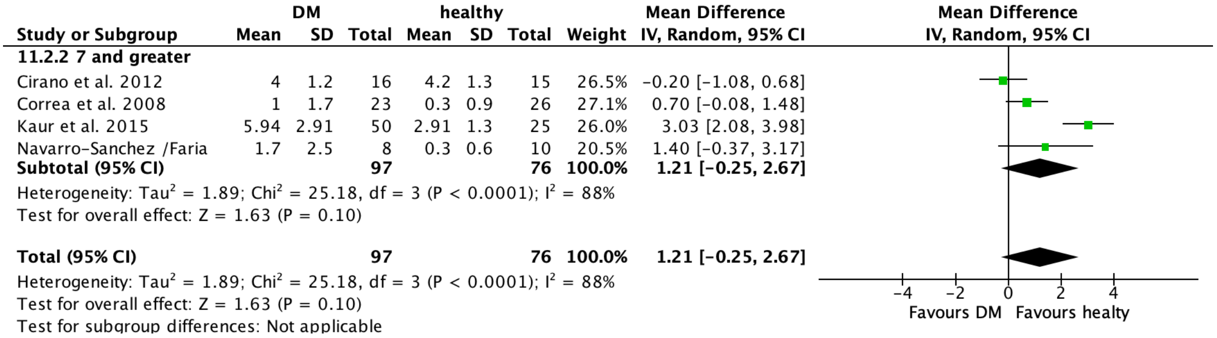

Online Appendix S7.39

Sub-analysis evaluating the effect of NSPT on DM compared to non-DM regarding deep pockets on incremental scores

Study or Subgroup	DM Mean	DM SD	DM Total	healthy Mean	healthy SD	healthy Total	Weight	Mean Difference IV, Fixed, 95% CI
**11.3.2 7 and greater**								
Correa et al. 2008	−12	12.8	23	−7.7	6.8	26	4.8%	−4.30 [−10.15, 1.55]
Kaur et al. 2015	−6.29	2.7	50	−7.91	3.64	25	63.4%	1.62 [0.01, 3.23]
Navarro-Sanchez /Faria	−2.4	3.1	8	−0.9	1.2	10	31.8%	−1.50 [−3.77, 0.77]
**Subtotal (95% CI)**			**81**			**61**	**100.0%**	**0.34 [−0.94, 1.62]**
Heterogeneity: Chi² = 7.36, df = 2 (P = 0.03); I² = 73%								
Test for overall effect: Z = 0.52 (P = 0.60)								
**Total (95% CI)**			**81**			**61**	**100.0%**	**0.34 [−0.94, 1.62]**
Heterogeneity: Chi² = 7.36, df = 2 (P = 0.03); I² = 73%								
Test for overall effect: Z = 0.52 (P = 0.60)								
Test for subgroup differences: Not applicable								

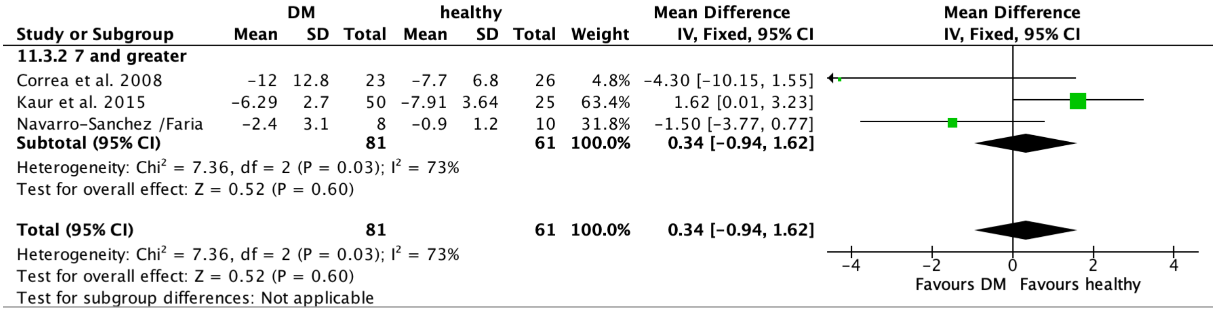

Online Appendix S7.40

Meta-analysis evaluating the effect of NSPT on DM compared to non-DM regarding PPD Europe at baseline

	DM			Control				Mean Difference
Study or Subgroup	Mean	SD	Total	Mean	SD	Total	Weight	IV, Random, 95% CI
Abreu et al. 2016	3.16	0.39	30	2.97	0.63	30	0.0%	0.19 [−0.08, 0.46]
Ahuja et al. 2019	5.6	0.38	30	4.98	0.49	30	0.0%	0.62 [0.40, 0.84]
Almeida et al. 2020	2.84	0.67	26	2.79	0.74	28	0.0%	0.05 [−0.33, 0.43]
Camargo et al. 2013	3.46	0.55	10	2.81	0.55	10	0.0%	0.65 [0.17, 1.13]
Christgau et al. 1998	3.6	1.6	20	3.8	1.7	20	3.0%	−0.20 [−1.22, 0.82]
Cirano et al. 2012	5.3	0.3	16	5.9	0.2	15	0.0%	−0.60 [−0.78, −0.42]
Correa et al. 2008	4.2	1	23	3.6	0.6	26	0.0%	0.60 [0.13, 1.07]
Da Cruz et al. 2008	5.72	1.13	10	4.7	0.9	10	0.0%	1.02 [0.12, 1.92]
Dag et al. DM + 2009	2.67	0.45	15	2.61	0.38	8	8.7%	0.06 [−0.29, 0.41]
Dag et al. DM − 2009	2.84	0.65	15	2.61	0.38	8	7.8%	0.23 [−0.19, 0.65]
Dogan et al. 2016	4.11	0.34	20	4.09	0.37	20	10.2%	0.02 [−0.20, 0.24]
Gayathri et al. 2019	3.57	0.4	30	3.29	0.42	30	0.0%	0.28 [0.07, 0.49]
Gomathi et al. 2023	5.6	0.54	15	4.72	0.4	15	0.0%	0.88 [0.54, 1.22]
Goncalvez /Silva et al.	4.18	0.9	20	3.62	0.56	20	0.0%	0.56 [0.10, 1.02]
Hungund et al. 2010	3.16	0.65	15	3.04	0.64	15	0.0%	0.12 [−0.34, 0.58]
Kara et al. 2015	4.13	0.44	15	4.12	0.428	15	9.2%	0.01 [−0.30, 0.32]
Kardesler et al. DM+ 2009	3.7	0.4	13	3.9	0.6	8	7.2%	−0.20 [−0.67, 0.27]
Kardesler et al. DM− 2009	4.1	1	12	3.9	0.6	8	4.9%	0.20 [−0.50, 0.90]
Kaur et al. 2015	2.96	0.46	50	2.77	0.58	25	0.0%	0.19 [−0.07, 0.45]
Kudva et al. 2010	3.31	1.54	15	3.63	1.61	15	0.0%	−0.32 [−1.45, 0.81]
Lopez et al. 2014	3.3	0.5	26	3.1	0.5	26	0.0%	0.20 [−0.07, 0.47]
Mirnic et al. DM + 2022	2.09	0.51	29	2.38	0.6	16	8.7%	−0.29 [−0.64, 0.06]
Mirnic et al. DM − 2022	2.11	0.47	32	2.38	0.6	16	8.9%	−0.27 [−0.61, 0.07]
Mishra et al. 2016	7.43	0.94	14	6.64	0.5	14	0.0%	0.79 [0.23, 1.35]
Navarro-Sanchez /Faria	4.1	0.3	8	3.7	0.5	10	8.4%	0.40 [0.03, 0.77]
Pannicker et al. 2016	7.94	1.57	35	7.45	1.68	35	0.0%	0.49 [−0.27, 1.25]
Peruzzo Lopes DM1 2017	1.83	0.05	14	2.12	0.06	6	11.5%	−0.29 [−0.34, −0.24]
Peruzzo Lopes DM2 2017	2.33	0.06	27	2.12	0.06	6	11.5%	0.21 [0.16, 0.26]
Pragada et al. 2019	7.3	1.69	30	6.83	1.7	30	0.0%	0.47 [−0.39, 1.33]
Sonoki et al. 2006	3.3	0.28	5	2.93	0.33	6	0.0%	0.37 [0.01, 0.73]
Sundaram et al. DM+ 2017	4.04	1.02	40	3.84	1.33	20	0.0%	0.20 [−0.46, 0.86]
Sundaram et al. DM− 2017	4.8	0.57	40	3.84	1.33	20	0.0%	0.96 [0.35, 1.57]
Total (95% CI)			220			141	100.0%	−0.01 [−0.22, 0.20]

Heterogeneity: $Tau^2 = 0.10$; $Chi^2 = 177.58$, df = 11 ($P < 0.00001$); $I^2 = 94\%$
Test for overall effect: Z = 0.09 (P = 0.93)

Mean Difference IV, Random, 95% CI

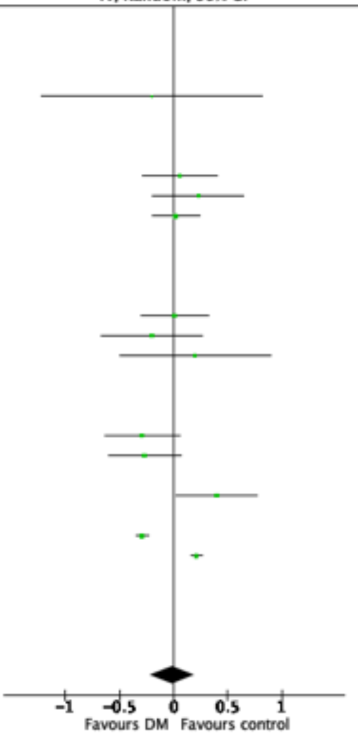

Online Appendix S7.41

Meta-analysis evaluating the effect of NSPT on DM compared to non-DM regarding PPD Europe post-NSPT

	DM			Control				Mean Difference
Study or Subgroup	Mean	SD	Total	Mean	SD	Total	Weight	IV, Random, 95% CI
Abreu et al. 2016	2.54	0.74	30	2.2	0.79	30	0.0%	0.34 [−0.05, 0.73]
Ahuja et al. 2019	3.07	0.6	30	2.42	0.72	30	0.0%	0.65 [0.31, 0.99]
Almeida et al. 2020	2.4	0.58	26	2.32	0.43	28	0.0%	0.08 [−0.19, 0.35]
Camargo et al. 2013	2.62	0.6	10	2.49	0.48	10	0.0%	0.13 [−0.35, 0.61]
Christgau et al. 1998	3.2	1.2	20	3.3	1.3	20	4.2%	−0.10 [−0.88, 0.68]
Cirano et al. 2012	3	0.5	16	3.2	0.5	15	0.0%	−0.20 [−0.55, 0.15]
Correa et al. 2008	2.8	0.4	23	2.4	0.3	26	0.0%	0.40 [0.20, 0.60]
Da Cruz et al. 2008	5	1.29	10	3.97	0.82	10	0.0%	1.03 [0.08, 1.98]
Dag et al. DM + 2009	2.3	0.43	15	2.36	0.68	8	6.3%	−0.06 [−0.58, 0.46]
Dag et al. DM − 2009	2.37	0.59	15	2.36	0.68	8	5.9%	0.01 [−0.55, 0.57]
Dogan et al. 2016	2.67	0.26	20	2.61	0.3	20	10.0%	0.06 [−0.11, 0.23]
Gayathri et al. 2019	3.44	0.42	30	3.16	0.46	30	0.0%	0.28 [0.06, 0.50]
Gomathi et al. 2023	2.97	0.2	15	3	0.16	15	0.0%	−0.03 [−0.16, 0.10]
Goncalvez /Silva et al.	2.76	0.41	20	2.44	0.3	20	0.0%	0.32 [0.10, 0.54]
Hungund et al. 2010	2.67	0.43	15	2.03	0.45	15	0.0%	0.64 [0.33, 0.95]
Kara et al. 2015	2.34	0.258	15	2.31	0.288	15	9.8%	0.03 [−0.17, 0.23]
Kardesler et al. DM+ 2009	2.8	0.4	13	2.7	0.3	8	8.7%	0.10 [−0.20, 0.40]
Kardesler et al. DM− 2009	3.1	0.6	12	2.7	0.3	8	7.6%	0.40 [0.00, 0.80]
Kaur et al. 2015	2.15	0.42	50	1.91	0.44	25	0.0%	0.24 [0.03, 0.45]
Kudva et al. 2010	1.69	1.06	15	1.78	1.06	15	0.0%	−0.09 [−0.85, 0.67]
Lopez et al. 2014	2.79	0.58	24	2.75	0.57	26	0.0%	0.04 [−0.28, 0.36]
Mirnic et al. DM + 2022	1.98	0.53	29	2.05	0.52	16	8.5%	−0.07 [−0.39, 0.25]
Mirnic et al. DM − 2022	2.03	0.35	32	2.05	0.52	16	8.9%	−0.02 [−0.30, 0.26]
Mishra et al. 2016	7	0.78	14	6.36	0.5	14	0.0%	0.64 [0.15, 1.13]
Navarro-Sanchez /Faria	3	0.4	8	2.6	0.2	10	8.7%	0.40 [0.10, 0.70]
Pannicker et al. 2016	5.74	1.24	35	5.62	1.33	35	0.0%	0.12 [−0.48, 0.72]
Peruzzo Lopes DM1 2017	1.36	0.02	14	1.76	0.04	6	10.7%	−0.40 [−0.43, −0.37]
Peruzzo Lopes DM2 2017	1.83	0.06	27	1.76	0.04	6	10.7%	0.07 [0.03, 0.11]
Pragada et al. 2019	5.26	1.18	30	5.06	1.36	30	0.0%	0.20 [−0.44, 0.84]
Sonoki et al. 2006	2.58	0.17	5	2.16	0.17	6	0.0%	0.42 [0.22, 0.62]
Sundaram et al. DM+ 2017	1.88	0.54	40	1.87	1.06	20	0.0%	0.01 [−0.48, 0.50]
Sundaram et al. DM− 2017	1.99	0.71	40	1.87	1.06	20	0.0%	0.12 [−0.39, 0.63]
Total (95% CI)			220			141	100.0%	0.03 [−0.17, 0.23]

Heterogeneity: $Tau^2 = 0.10$; $Chi^2 = 359.80$, df = 11 ($P < 0.00001$); $I^2 = 97\%$
Test for overall effect: Z = 0.31 (P = 0.76)

Mean Difference IV, Random, 95% CI

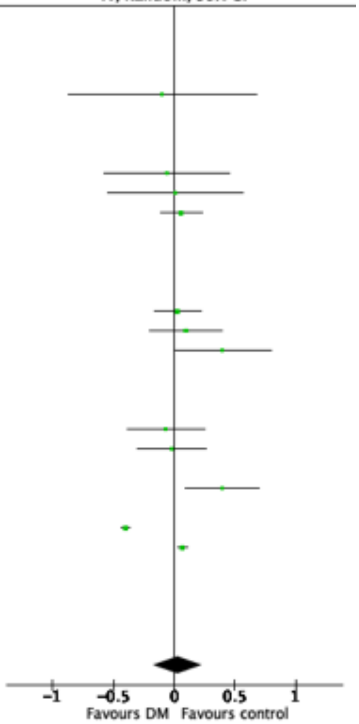

Online Appendix S7.42

Meta-analysis evaluating the effect of NSPT on DM compared to non-DM regarding PPD Europe on incremental scores

Study or Subgroup	DM Mean	DM SD	DM Total	Control Mean	Control SD	Control Total	Weight	Mean Difference IV, Random, 95% CI
Sonoki et al. 2006	−0.76	0.56	5	−0.72	0.46	6	0.0%	−0.04 [−0.65, 0.57]
Peruzzo Lopes DM2 2017	−0.5	0.03	27	−0.48	0.04	6	27.9%	−0.02 [−0.05, 0.01]
Peruzzo Lopes DM1 2017	−0.47	0.02	14	−0.48	0.04	6	28.0%	0.01 [−0.02, 0.04]
Navarro-Sanchez /Faria	−1.1	0.2	8	−1.1	0.3	10	7.7%	0.00 [−0.23, 0.23]
Mishra et al. 2016	−0.43	0.51	14	−0.29	0.47	14	0.0%	−0.14 [−0.50, 0.22]
Mirnic et al. DM − 2022	−0.08	0.27	32	−0.34	0.23	16	13.8%	0.26 [0.11, 0.41]
Mirnic et al. DM + 2022	−0.11	0.16	29	−0.34	0.23	16	16.0%	0.23 [0.10, 0.36]
Lopez et al. 2014	−0.51	0.08	24	−0.35	0.07	26	0.0%	−0.16 [−0.20, −0.12]
Kudva et al. 2010	−1.62	0.79	15	−1.85	0.81	15	0.0%	0.23 [−0.34, 0.80]
Kaur et al. 2015	−0.81	0.28	50	−0.86	0.29	25	0.0%	0.05 [−0.09, 0.19]
Kardesler et al. DM− 2009	−1.06	0.54	12	−1.17	0.51	8	2.3%	0.11 [−0.36, 0.58]
Kardesler et al. DM+ 2009	−0.93	0.37	13	−1.17	0.51	8	3.0%	0.24 [−0.17, 0.65]
Hungund et al. 2010	−0.49	0.18	15	−1.01	0.19	15	0.0%	0.52 [0.39, 0.65]
Goncalvez /Silva et al.	−1.42	0.61	20	−1.17	0.54	20	0.0%	−0.25 [−0.61, 0.11]
Da Cruz et al. 2008	−0.71	0.19	10	−0.82	0.13	10	0.0%	0.11 [−0.03, 0.25]
Correa et al. 2008	−1.5	0.7	23	−1.2	0.6	26	0.0%	−0.30 [−0.67, 0.07]
Christgau et al. 1998	−0.4	1.1	20	−0.5	1	20	1.3%	0.10 [−0.55, 0.75]
Camargo et al. 2013	−0.9	0.37	10	−0.33	0.31	10	0.0%	−0.57 [−0.87, −0.27]
Almeida et al. 2020	−0.44	0.28	26	−0.47	0.6	28	0.0%	0.03 [−0.22, 0.28]
Ahuja et al. 2019	−2.53	0.24	30	−2.57	0.26	30	0.0%	0.04 [−0.09, 0.17]
Abreu et al. 2016	−1.12	0.07	30	−0.74	0.74	30	0.0%	−0.38 [−0.65, −0.11]
Total (95% CI)			155			90	100.0%	0.08 [0.01, 0.16]

Heterogeneity: $Tau^2 = 0.00$; $Chi^2 = 27.23$, $df = 7$ ($P = 0.0003$); $I^2 = 74\%$
Test for overall effect: $Z = 2.13$ ($P = 0.03$)

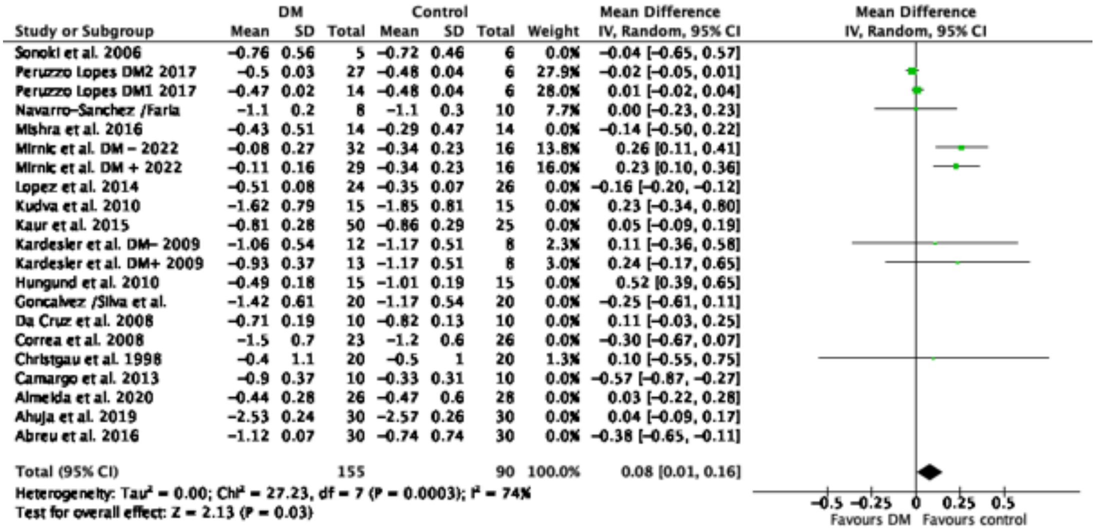

Online Appendix S7.43

Meta-analysis evaluating the effect of NSPT on DM compared to non-DM regarding PPD on Asia at baseline

Study or Subgroup	DM Mean	DM SD	DM Total	Control Mean	Control SD	Control Total	Weight	Mean Difference IV, Random, 95% CI
Abreu et al. 2016	3.16	0.39	30	2.97	0.63	30	0.0%	0.19 [−0.08, 0.46]
Ahuja et al. 2019	5.6	0.38	30	4.98	0.49	30	15.4%	0.62 [0.40, 0.84]
Almeida et al. 2020	2.84	0.67	26	2.79	0.74	28	0.0%	0.05 [−0.33, 0.43]
Camargo et al. 2013	3.46	0.55	10	2.81	0.55	10	0.0%	0.65 [0.17, 1.13]
Christgau et al. 1998	3.6	1.6	20	3.8	1.7	20	0.0%	−0.20 [−1.22, 0.82]
Cirano et al. 2012	5.3	0.3	16	5.9	0.2	15	0.0%	−0.60 [−0.78, −0.42]
Correa et al. 2008	4.2	1	23	3.6	0.6	26	0.0%	0.60 [0.13, 1.07]
Da Cruz et al. 2008	5.72	1.13	10	4.7	0.9	10	0.0%	1.02 [0.12, 1.92]
Dag et al. DM + 2009	2.67	0.45	15	2.61	0.38	8	0.0%	0.06 [−0.29, 0.41]
Dag et al. DM − 2009	2.84	0.65	15	2.61	0.38	8	0.0%	0.23 [−0.19, 0.65]
Dogan et al. 2016	4.11	0.34	20	4.09	0.37	20	0.0%	0.02 [−0.20, 0.24]
Gayathri et al. 2019	3.57	0.4	30	3.29	0.42	30	15.9%	0.28 [0.07, 0.49]
Gomathi et al. 2023	5.6	0.54	15	4.72	0.4	15	11.9%	0.88 [0.54, 1.22]
Goncalvez /Silva et al.	4.18	0.9	20	3.62	0.56	20	0.0%	0.56 [0.10, 1.02]
Hungund et al. 2010	3.16	0.65	15	3.04	0.64	15	8.9%	0.12 [−0.34, 0.58]
Kara et al. 2015	4.13	0.44	15	4.12	0.428	15	0.0%	0.01 [−0.30, 0.32]
Kardesler et al. DM+ 2009	3.7	0.4	13	3.9	0.6	8	0.0%	−0.20 [−0.67, 0.27]
Kardesler et al. DM− 2009	4.1	1	12	3.9	0.6	8	0.0%	0.20 [−0.50, 0.90]
Kaur et al. 2015	2.96	0.46	50	2.77	0.58	25	14.2%	0.19 [−0.07, 0.45]
Kudva et al. 2010	3.31	1.54	15	3.63	1.61	15	2.4%	−0.32 [−1.45, 0.81]
Lopez et al. 2014	3.3	0.5	26	3.1	0.5	26	0.0%	0.20 [−0.07, 0.47]
Mirnic et al. DM + 2022	2.09	0.51	29	2.38	0.6	16	0.0%	−0.29 [−0.64, 0.06]
Mirnic et al. DM − 2022	2.11	0.47	32	2.38	0.6	16	0.0%	−0.27 [−0.61, 0.07]
Mishra et al. 2016	7.43	0.94	14	6.64	0.5	14	7.1%	0.79 [0.23, 1.35]
Navarro-Sanchez /Faria	4.1	0.3	8	3.7	0.5	10	0.0%	0.40 [0.03, 0.77]
Pannicker et al. 2016	7.94	1.57	35	7.45	1.68	35	4.6%	0.49 [−0.27, 1.25]
Peruzzo Lopes DM1 2017	1.83	0.05	14	2.12	0.06	6	0.0%	−0.29 [−0.34, −0.24]
Peruzzo Lopes DM2 2017	2.33	0.06	27	2.12	0.06	6	0.0%	0.21 [0.16, 0.26]
Pragada et al. 2019	7.3	1.69	30	6.83	1.7	30	3.8%	0.47 [−0.39, 1.33]
Sonoki et al. 2006	3.3	0.63	5	2.93	0.81	6	3.8%	0.37 [−0.48, 1.22]
Sundaram et al. DM+ 2017	4.04	1.02	40	3.84	1.33	20	5.6%	0.20 [−0.46, 0.86]
Sundaram et al. DM− 2017	4.8	0.57	40	3.84	1.33	20	6.3%	0.96 [0.35, 1.57]
Total (95% CI)			319			255	100.0%	0.46 [0.27, 0.64]

Heterogeneity: $Tau^2 = 0.05$; $Chi^2 = 23.23$, $df = 11$ ($P = 0.02$); $I^2 = 53\%$
Test for overall effect: $Z = 4.82$ ($P < 0.00001$)

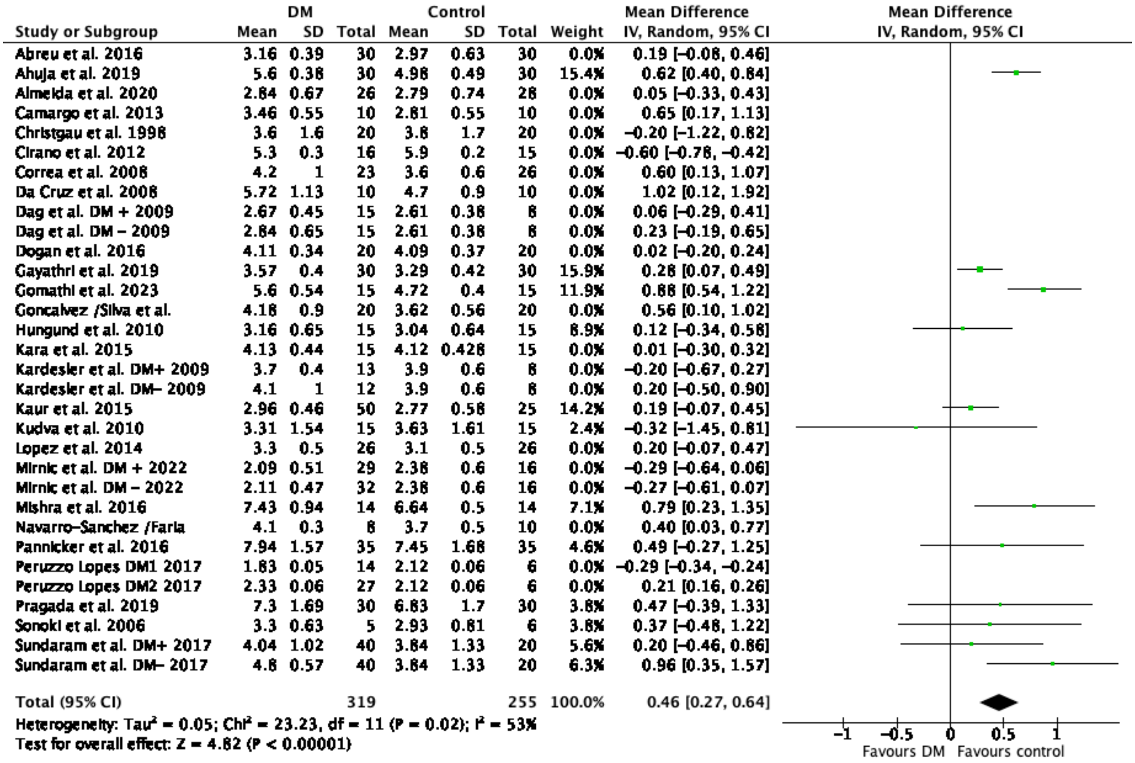

Online Appendix S7.44

Meta-analysis evaluating the effect of NSPT on DM compared to non-DM regarding PPD on Asia post-NSPT

Study or Subgroup	DM Mean	DM SD	DM Total	Control Mean	Control SD	Control Total	Weight	Mean Difference IV, Random, 95% CI
Abreu et al. 2016	2.54	0.74	30	2.2	0.79	30	0.0%	0.34 [−0.05, 0.73]
Ahuja et al. 2019	3.07	0.6	30	2.42	0.72	30	9.7%	0.65 [0.31, 0.99]
Almeida et al. 2020	2.4	0.58	26	2.32	0.43	28	0.0%	0.08 [−0.19, 0.35]
Camargo et al. 2013	2.62	0.6	10	2.49	0.48	10	0.0%	0.13 [−0.35, 0.61]
Christgau et al. 1998	3.2	1.2	20	3.3	1.3	20	0.0%	−0.10 [−0.88, 0.68]
Cirano et al. 2012	3	0.5	16	3.2	0.5	15	0.0%	−0.20 [−0.55, 0.15]
Correa et al. 2008	2.8	0.4	23	2.4	0.3	26	0.0%	0.40 [0.20, 0.60]
Da Cruz et al. 2008	5	1.29	10	3.97	0.82	10	0.0%	1.03 [0.08, 1.98]
Dag et al. DM + 2009	2.3	0.43	15	2.36	0.68	8	0.0%	−0.06 [−0.58, 0.46]
Dag et al. DM − 2009	2.37	0.59	15	2.36	0.68	8	0.0%	0.01 [−0.55, 0.57]
Dogan et al. 2016	2.67	0.26	20	2.61	0.3	20	0.0%	0.06 [−0.11, 0.23]
Gayathri et al. 2019	3.44	0.42	30	3.16	0.46	30	12.2%	0.28 [0.06, 0.50]
Gomathi et al. 2023	2.97	0.2	15	3	0.16	15	14.2%	−0.03 [−0.16, 0.10]
Goncalvez /Silva et al.	2.76	0.41	20	2.44	0.3	20	0.0%	0.32 [0.10, 0.54]
Hungund et al. 2010	2.67	0.43	15	2.03	0.45	15	10.1%	0.64 [0.33, 0.95]
Kara et al. 2015	2.34	0.258	15	2.31	0.288	15	0.0%	0.03 [−0.17, 0.23]
Kardesler et al. DM+ 2009	2.8	0.4	13	2.7	0.3	8	0.0%	0.10 [−0.20, 0.40]
Kardesler et al. DM− 2009	3.1	0.6	12	2.7	0.3	8	0.0%	0.40 [0.00, 0.80]
Kaur et al. 2015	2.15	0.42	50	1.91	0.44	25	12.6%	0.24 [0.03, 0.45]
Kudva et al. 2010	1.69	1.06	15	1.78	1.06	15	3.8%	−0.09 [−0.85, 0.67]
Lopez et al. 2014	2.79	0.58	24	2.75	0.57	26	0.0%	0.04 [−0.28, 0.36]
Mirnic et al. DM + 2022	1.98	0.53	29	2.05	0.52	16	0.0%	−0.07 [−0.39, 0.25]
Mirnic et al. DM − 2022	2.03	0.35	32	2.05	0.52	16	0.0%	−0.02 [−0.30, 0.26]
Mishra et al. 2016	7	0.78	14	6.36	0.5	14	6.9%	0.64 [0.15, 1.13]
Navarro–Sanchez /Faria	3	0.4	8	2.6	0.2	10	0.0%	0.40 [0.10, 0.70]
Pannicker et al. 2016	5.74	1.24	35	5.62	1.33	35	5.3%	0.12 [−0.48, 0.72]
Peruzzo Lopes DM1 2017	1.36	0.02	14	1.76	0.04	6	0.0%	−0.40 [−0.43, −0.37]
Peruzzo Lopes DM2 2017	1.83	0.06	27	1.76	0.04	6	0.0%	0.07 [0.03, 0.11]
Pragada et al. 2019	5.26	1.18	30	5.06	1.36	30	4.8%	0.20 [−0.44, 0.84]
Sonoki et al. 2006	2.58	0.38	5	2.16	0.42	6	7.1%	0.42 [−0.05, 0.89]
Sundaram et al. DM+ 2017	1.88	0.54	40	1.87	1.06	20	6.8%	0.01 [−0.48, 0.50]
Sundaram et al. DM− 2017	1.99	0.71	40	1.87	1.06	20	6.5%	0.12 [−0.39, 0.63]
Total (95% CI)			319			255	100.0%	0.28 [0.11, 0.45]

Heterogeneity: $Tau^2 = 0.05$; $Chi^2 = 32.35$, df = 11 (P = 0.0007); $I^2 = 66\%$
Test for overall effect: Z = 3.24 (P = 0.001)

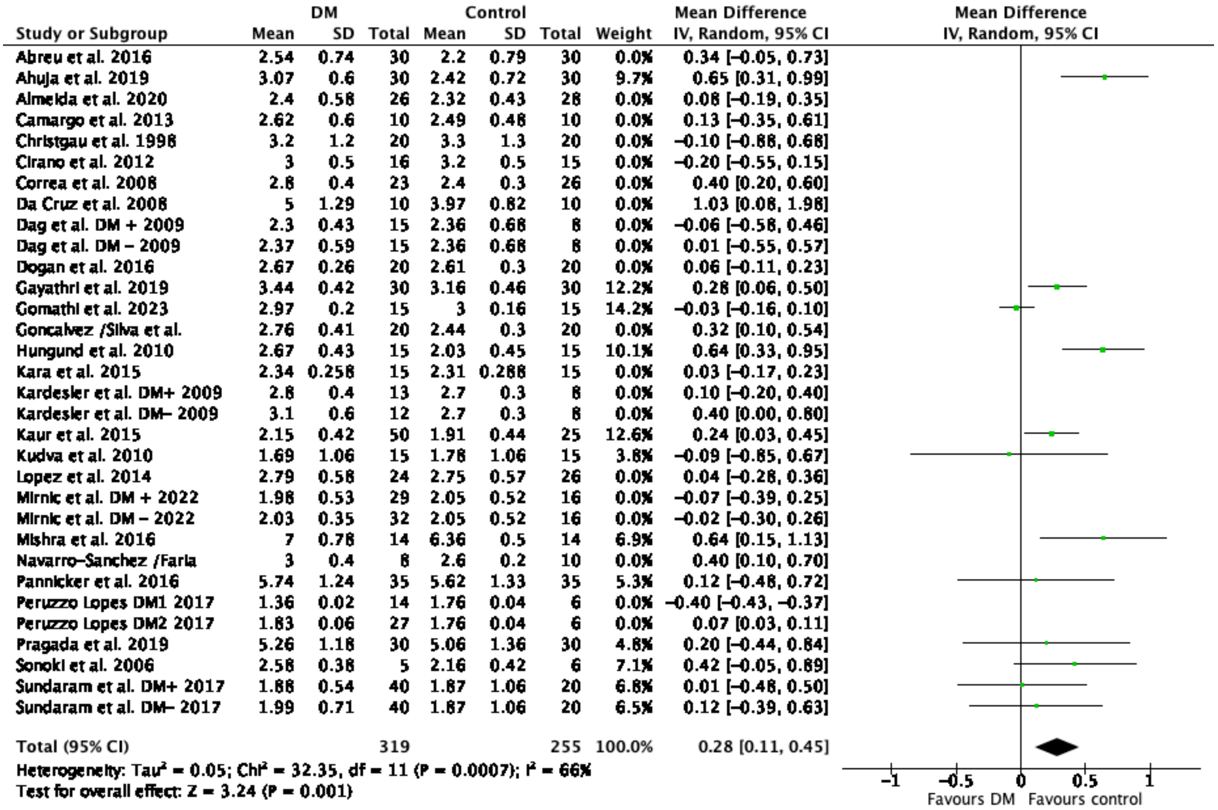

Online Appendix S7.45

Meta-analysis evaluating the effect of NSPT on DM compared to non-DM regarding PPD on Asia on incremental scores

Study or Subgroup	DM Mean	DM SD	DM Total	Control Mean	Control SD	Control Total	Weight	Mean Difference IV, Random, 95% CI
Sonoki et al. 2006	−0.76	0.56	5	−0.72	0.46	6	9.2%	−0.04 [−0.65, 0.57]
Peruzzo Lopes DM2 2017	−0.5	0.03	27	−0.48	0.04	6	0.0%	−0.02 [−0.05, 0.01]
Peruzzo Lopes DM1 2017	−0.47	0.02	14	−0.48	0.04	6	0.0%	0.01 [−0.02, 0.04]
Navarro–Sanchez /Faria	−1.1	0.2	8	−1.1	0.3	10	0.0%	0.00 [−0.23, 0.23]
Mishra et al. 2016	−0.43	0.51	14	−0.29	0.47	14	15.2%	−0.14 [−0.50, 0.22]
Mirnic et al. DM − 2022	−0.08	0.27	32	−0.34	0.23	16	0.0%	0.26 [0.11, 0.41]
Mirnic et al. DM + 2022	−0.11	0.16	29	−0.34	0.23	16	0.0%	0.23 [0.10, 0.36]
Lopez et al. 2014	−0.51	0.08	24	−0.35	0.07	26	0.0%	−0.16 [−0.20, −0.12]
Kudva et al. 2010	−1.62	0.79	15	−1.85	0.81	15	10.0%	0.23 [−0.34, 0.80]
Kaur et al. 2015	−0.81	0.28	50	−0.86	0.29	25	21.8%	0.05 [−0.09, 0.19]
Kardesler et al. DM− 2009	−1.06	0.54	12	−1.17	0.51	8	0.0%	0.11 [−0.36, 0.58]
Kardesler et al. DM+ 2009	−0.93	0.37	13	−1.17	0.51	8	0.0%	0.24 [−0.17, 0.65]
Hungund et al. 2010	−0.49	0.18	15	−1.01	0.19	15	21.9%	0.52 [0.39, 0.65]
Goncalvez /Silva et al.	−1.42	0.61	20	−1.17	0.54	20	0.0%	−0.25 [−0.61, 0.11]
Da Cruz et al. 2008	−0.71	0.19	10	−0.82	0.13	10	0.0%	0.11 [−0.03, 0.25]
Correa et al. 2008	−1.5	0.7	23	−1.2	0.6	26	0.0%	−0.30 [−0.67, 0.07]
Christgau et al. 1998	−0.4	1.1	20	−0.5	1	20	0.0%	0.10 [−0.55, 0.75]
Camargo et al. 2013	−0.9	0.37	10	−0.33	0.31	10	0.0%	−0.57 [−0.87, −0.27]
Almeida et al. 2020	−0.44	0.28	26	−0.47	0.6	28	0.0%	0.03 [−0.22, 0.28]
Ahuja et al. 2019	−2.53	0.24	30	−2.57	0.26	30	22.0%	0.04 [−0.09, 0.17]
Abreu et al. 2016	−1.12	0.07	30	−0.74	0.74	30	0.0%	−0.38 [−0.65, −0.11]
Total (95% CI)			129			105	100.0%	0.13 [−0.11, 0.37]

Heterogeneity: $Tau^2 = 0.06$; $Chi^2 = 36.92$, df = 5 (P < 0.00001); $I^2 = 86\%$
Test for overall effect: Z = 1.08 (P = 0.28)

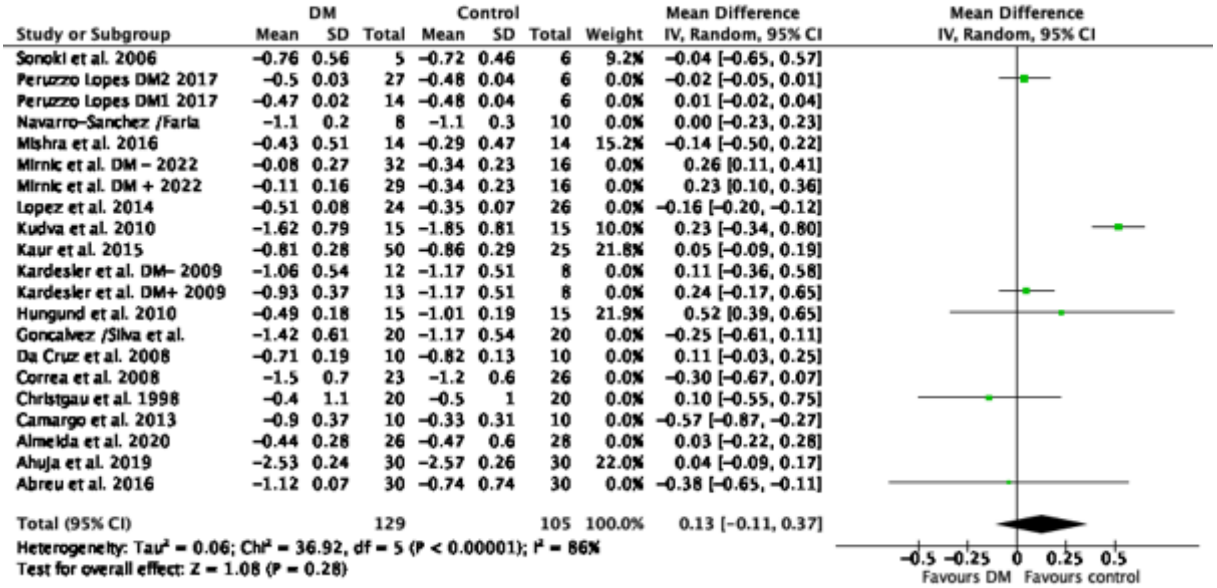

Online Appendix S7.46

Meta-analysis evaluating the effect of NSPT on DM compared to non-DM regarding PPD on South America at baseline

Study or Subgroup	DM Mean	DM SD	DM Total	Control Mean	Control SD	Control Total	Weight	Mean Difference IV, Random, 95% CI
Abreu et al. 2016	3.16	0.39	30	2.97	0.63	30	13.9%	0.19 [−0.08, 0.46]
Ahuja et al. 2019	5.6	0.38	30	4.98	0.49	30	0.0%	0.62 [0.40, 0.84]
Almeida et al. 2020	2.84	0.67	26	2.79	0.74	28	13.0%	0.05 [−0.33, 0.43]
Camargo et al. 2013	3.46	0.55	10	2.81	0.55	10	12.1%	0.65 [0.17, 1.13]
Christgau et al. 1998	3.6	1.6	20	3.8	1.7	20	0.0%	−0.20 [−1.22, 0.82]
Cirano et al. 2012	5.3	0.3	16	5.9	0.2	15	14.4%	−0.60 [−0.78, −0.42]
Correa et al. 2008	4.2	1	23	3.6	0.6	26	12.2%	0.60 [0.13, 1.07]
Da Cruz et al. 2008	5.72	1.13	10	4.7	0.9	10	8.3%	1.02 [0.12, 1.92]
Dag et al. DM + 2009	2.67	0.45	15	2.61	0.38	8	0.0%	0.06 [−0.29, 0.41]
Dag et al. DM − 2009	2.84	0.65	15	2.61	0.38	8	0.0%	0.23 [−0.19, 0.65]
Dogan et al. 2016	4.11	0.34	20	4.09	0.37	20	0.0%	0.02 [−0.20, 0.24]
Gayathri et al. 2019	3.57	0.4	30	3.29	0.42	30	0.0%	0.28 [0.07, 0.49]
Gomathi et al. 2023	5.6	0.54	15	4.72	0.4	15	0.0%	0.88 [0.54, 1.22]
Goncalvez /Silva et al.	4.18	0.9	20	3.62	0.56	20	12.2%	0.56 [0.10, 1.02]
Hungund et al. 2010	3.16	0.65	15	3.04	0.64	15	0.0%	0.12 [−0.34, 0.58]
Kara et al. 2015	4.13	0.44	15	4.12	0.428	15	0.0%	0.01 [−0.30, 0.32]
Kardesler et al. DM+ 2009	3.7	0.4	13	3.9	0.6	8	0.0%	−0.20 [−0.67, 0.27]
Kardesler et al. DM− 2009	4.1	1	12	3.9	0.6	8	0.0%	0.20 [−0.50, 0.90]
Kaur et al. 2015	2.96	0.46	50	2.77	0.58	25	0.0%	0.19 [−0.07, 0.45]
Kudva et al. 2010	3.31	1.54	15	3.63	1.61	15	0.0%	−0.32 [−1.45, 0.81]
Lopez et al. 2014	3.3	0.5	26	3.1	0.5	26	13.8%	0.20 [−0.07, 0.47]
Mirnic et al. DM + 2022	2.09	0.51	29	2.38	0.6	16	0.0%	−0.29 [−0.64, 0.06]
Mirnic et al. DM − 2022	2.11	0.47	32	2.38	0.6	16	0.0%	−0.27 [−0.61, 0.07]
Mishra et al. 2016	7.43	0.94	14	6.64	0.5	14	0.0%	0.79 [0.23, 1.35]
Navarro-Sanchez /Faria	4.1	0.3	8	3.7	0.5	10	0.0%	0.40 [0.03, 0.77]
Pannicker et al. 2016	7.94	1.57	35	7.45	1.68	35	0.0%	0.49 [−0.27, 1.25]
Peruzzo Lopes DM1 2017	1.83	0.05	14	2.12	0.06	6	0.0%	−0.29 [−0.34, −0.24]
Peruzzo Lopes DM2 2017	2.33	0.06	27	2.12	0.06	6	0.0%	0.21 [0.16, 0.26]
Pragada et al. 2019	7.3	1.69	30	6.83	1.7	30	0.0%	0.47 [−0.39, 1.33]
Sonoki et al. 2006	3.3	0.28	5	2.93	0.33	6	0.0%	0.37 [0.01, 0.73]
Sundaram et al. DM+ 2017	4.04	1.02	40	3.84	1.33	20	0.0%	0.20 [−0.46, 0.86]
Sundaram et al. DM− 2017	4.8	0.57	40	3.84	1.33	20	0.0%	0.96 [0.35, 1.57]
Total (95% CI)			161			165	100.0%	0.28 [−0.11, 0.67]

Heterogeneity: Tau² = 0.26; Chi² = 70.72, df = 7 (P < 0.00001); I² = 90%

Test for overall effect: Z = 1.41 (P = 0.16)

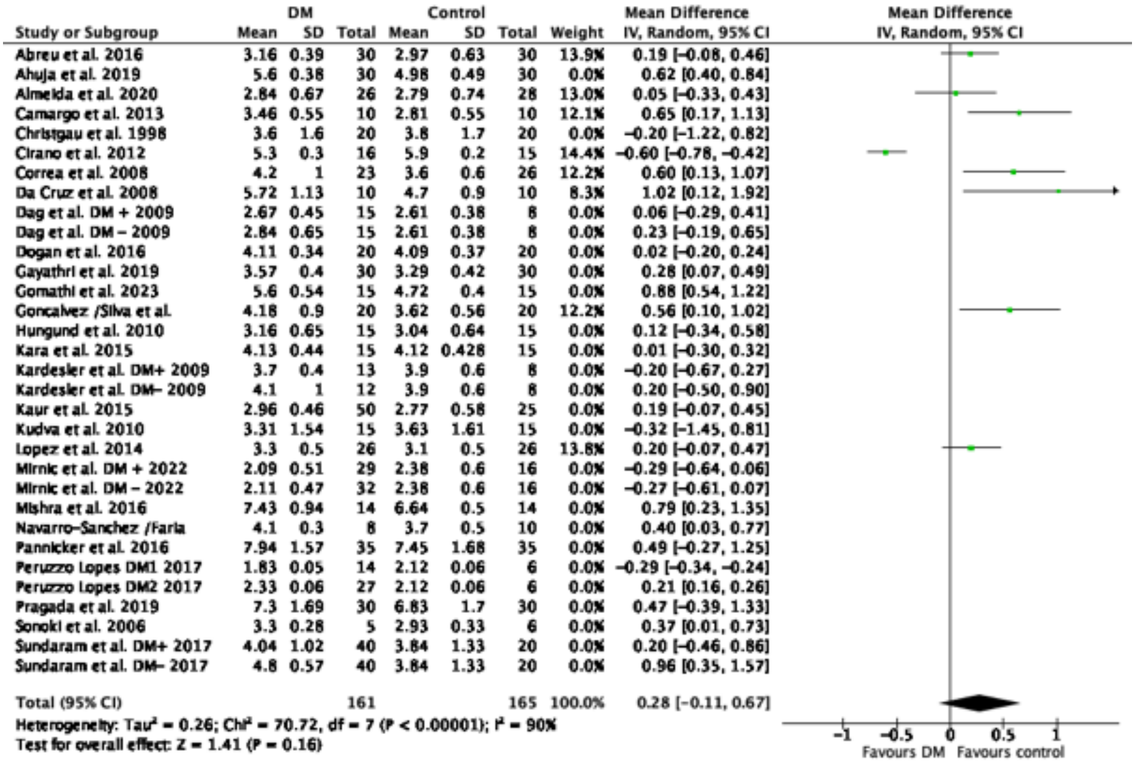

Online Appendix S7.47

Meta-analysis evaluating the effect of NSPT on DM compared to non-DM regarding PPD on South America post-NSPT

Study or Subgroup	DM Mean	DM SD	DM Total	Control Mean	Control SD	Control Total	Weight	Mean Difference IV, Random, 95% CI
Abreu et al. 2016	2.54	0.74	30	2.2	0.79	30	10.9%	0.34 [−0.05, 0.73]
Ahuja et al. 2019	3.07	0.6	30	2.42	0.72	30	0.0%	0.65 [0.31, 0.99]
Almeida et al. 2020	2.4	0.58	26	2.32	0.43	28	15.4%	0.08 [−0.19, 0.35]
Camargo et al. 2013	2.62	0.6	10	2.49	0.48	10	8.4%	0.13 [−0.35, 0.61]
Christgau et al. 1998	3.2	1.2	20	3.3	1.3	20	0.0%	−0.10 [−0.88, 0.68]
Cirano et al. 2012	3	0.5	16	3.2	0.5	15	12.1%	−0.20 [−0.55, 0.15]
Correa et al. 2008	2.8	0.4	23	2.4	0.3	26	19.0%	0.40 [0.20, 0.60]
Da Cruz et al. 2008	5	1.29	10	3.97	0.82	10	2.8%	1.03 [0.08, 1.98]
Dag et al. DM + 2009	2.3	0.43	15	2.36	0.68	8	0.0%	−0.06 [−0.58, 0.46]
Dag et al. DM − 2009	2.37	0.59	15	2.36	0.68	8	0.0%	0.01 [−0.55, 0.57]
Dogan et al. 2016	2.67	0.26	20	2.61	0.3	20	0.0%	0.06 [−0.11, 0.23]
Gayathri et al. 2019	3.44	0.42	30	3.16	0.46	30	0.0%	0.28 [0.06, 0.50]
Gomathi et al. 2023	2.97	0.2	15	3	0.16	15	0.0%	−0.03 [−0.16, 0.10]
Goncalvez /Silva et al.	2.76	0.41	20	2.44	0.3	20	17.9%	0.32 [0.10, 0.54]
Hungund et al. 2010	2.67	0.43	15	2.03	0.45	15	0.0%	0.64 [0.33, 0.95]
Kara et al. 2015	2.34	0.258	15	2.31	0.288	15	0.0%	0.03 [−0.17, 0.23]
Kardesler et al. DM+ 2009	2.8	0.4	13	2.7	0.3	8	0.0%	0.10 [−0.20, 0.40]
Kardesler et al. DM− 2009	3.1	0.6	12	2.7	0.3	8	0.0%	0.40 [0.00, 0.80]
Kaur et al. 2015	2.15	0.42	50	1.91	0.44	25	0.0%	0.24 [0.03, 0.45]
Kudva et al. 2010	1.69	1.06	15	1.78	1.06	15	0.0%	−0.09 [−0.85, 0.67]
Lopez et al. 2014	2.79	0.58	24	2.75	0.57	26	13.4%	0.04 [−0.28, 0.36]
Mirnic et al. DM + 2022	1.98	0.53	29	2.05	0.52	16	0.0%	−0.07 [−0.39, 0.25]
Mirnic et al. DM − 2022	2.03	0.35	32	2.05	0.52	16	0.0%	−0.02 [−0.30, 0.26]
Mishra et al. 2016	7	0.78	14	6.36	0.5	14	0.0%	0.64 [0.15, 1.13]
Navarro-Sanchez /Faria	3	0.4	8	2.6	0.2	10	0.0%	0.40 [0.10, 0.70]
Pannicker et al. 2016	5.74	1.24	35	5.62	1.33	35	0.0%	0.12 [−0.48, 0.72]
Peruzzo Lopes DM1 2017	1.36	0.02	14	1.76	0.04	6	0.0%	−0.40 [−0.43, −0.37]
Peruzzo Lopes DM2 2017	1.83	0.06	27	1.76	0.04	6	0.0%	0.07 [0.03, 0.11]
Pragada et al. 2019	5.26	1.18	30	5.06	1.36	30	0.0%	0.20 [−0.44, 0.84]
Sonoki et al. 2006	2.58	0.17	5	2.16	0.17	6	0.0%	0.42 [0.22, 0.62]
Sundaram et al. DM+ 2017	1.88	0.54	40	1.87	1.06	20	0.0%	0.01 [−0.48, 0.50]
Sundaram et al. DM− 2017	1.99	0.71	40	1.87	1.06	20	0.0%	0.12 [−0.39, 0.63]
Total (95% CI)			159			165	100.0%	0.20 [0.04, 0.37]

Heterogeneity: Tau² = 0.03; Chi² = 14.86, df = 7 (P = 0.04); I² = 53%

Test for overall effect: Z = 2.37 (P = 0.02)

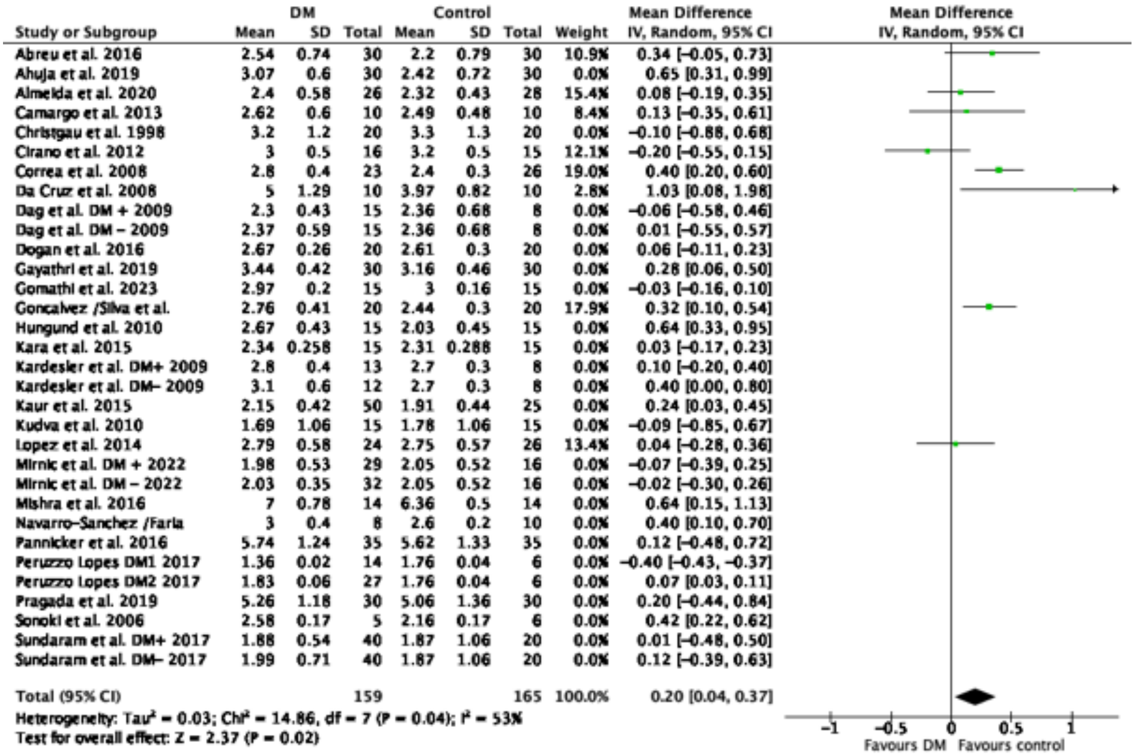

Online Appendix S7.48

Meta-analysis evaluating the effect of NSPT on DM compared to non-DM regarding PPD on South America on incremental scores

	DM			Control				Mean Difference
Study or Subgroup	Mean	SD	Total	Mean	SD	Total	Weight	IV, Random, 95% CI
Abreu et al. 2016	−1.12	0.07	30	−0.74	0.74	30	13.4%	−0.38 [−0.65, −0.11]
Ahuja et al. 2019	−2.53	0.24	30	−2.57	0.26	30	0.0%	0.04 [−0.09, 0.17]
Almeida et al. 2020	−0.44	0.28	26	−0.47	0.6	28	14.1%	0.03 [−0.22, 0.28]
Camargo et al. 2013	−0.9	0.37	10	−0.33	0.31	10	12.1%	−0.57 [−0.87, −0.27]
Christgau et al. 1998	−0.4	1.1	20	−0.5	1	20	0.0%	0.10 [−0.55, 0.75]
Correa et al. 2008	−1.5	0.7	23	−1.2	0.6	26	9.8%	−0.30 [−0.67, 0.07]
Da Cruz et al. 2008	−0.71	0.19	10	−0.82	0.13	10	18.6%	0.11 [−0.03, 0.25]
Goncalvez /Silva et al.	−1.42	0.61	20	−1.17	0.54	20	10.1%	−0.25 [−0.61, 0.11]
Hungund et al. 2010	−0.49	0.18	15	−1.01	0.19	15	0.0%	0.52 [0.39, 0.65]
Kardesler et al. DM+ 2009	−0.93	0.37	13	−1.17	0.51	8	0.0%	0.24 [−0.17, 0.65]
Kardesler et al. DM− 2009	−1.06	0.54	12	−1.17	0.51	8	0.0%	0.11 [−0.36, 0.58]
Kaur et al. 2015	−0.81	0.28	50	−0.86	0.29	25	0.0%	0.05 [−0.09, 0.19]
Kudva et al. 2010	−1.62	0.79	15	−1.85	0.81	15	0.0%	0.23 [−0.34, 0.80]
Lopez et al. 2014	−0.51	0.08	24	−0.35	0.07	26	21.8%	−0.16 [−0.20, −0.12]
Mirnic et al. DM + 2022	−0.11	0.16	29	−0.34	0.23	16	0.0%	0.23 [0.10, 0.36]
Mirnic et al. DM – 2022	−0.08	0.27	32	−0.34	0.23	16	0.0%	0.26 [0.11, 0.41]
Mishra et al. 2016	−0.43	0.51	14	−0.29	0.47	14	0.0%	−0.14 [−0.50, 0.22]
Navarro-Sanchez /Faria	−1.1	0.2	8	−1.1	0.3	10	0.0%	0.00 [−0.23, 0.23]
Peruzzo Lopes DM1 2017	−0.47	0.02	14	−0.48	0.04	6	0.0%	0.01 [−0.02, 0.04]
Peruzzo Lopes DM2 2017	−0.5	0.03	27	−0.48	0.04	6	0.0%	−0.02 [−0.05, 0.01]
Sonoki et al. 2006	−0.76	0.56	5	−0.72	0.46	6	0.0%	−0.04 [−0.65, 0.57]
Total (95% CI)			143			150	100.0%	−0.18 [−0.34, −0.03]

Heterogeneity: $Tau^2 = 0.03$; $Chi^2 = 26.40$, df = 6 ($P = 0.0002$); $I^2 = 77\%$

Test for overall effect: $Z = 2.35$ ($P = 0.02$)

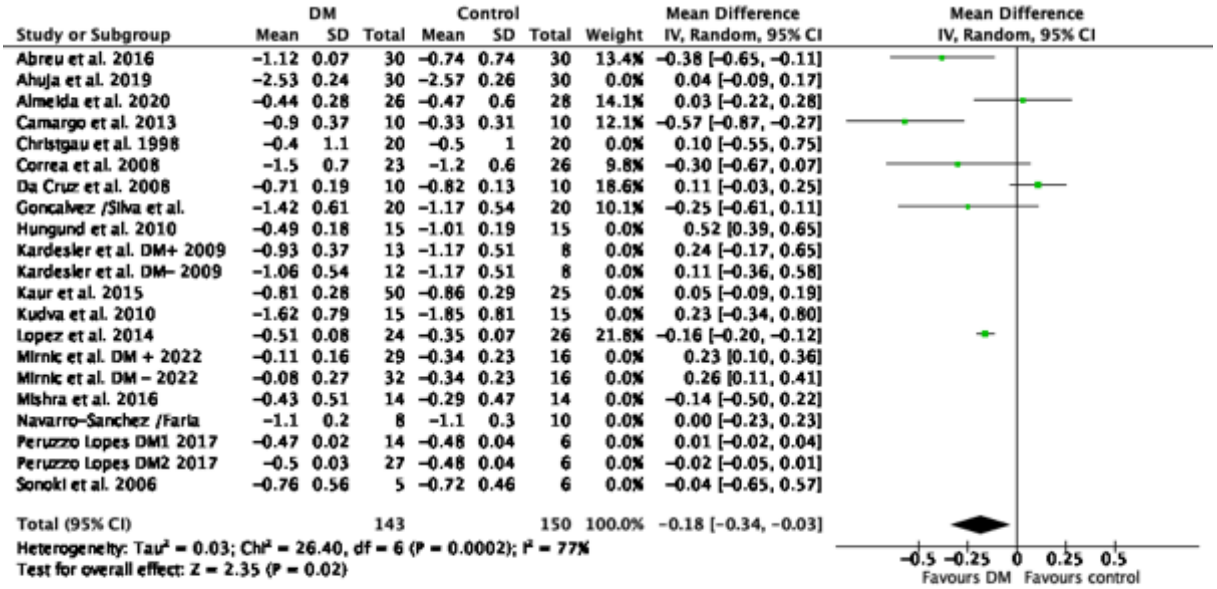

Online Appendix S7.49

Meta-analysis evaluating the effect of NSPT on DM compared to non-DM regarding CAL on Europe at baseline

	DM			Control				Mean Difference
Study or Subgroup	Mean	SD	Total	Mean	SD	Total	Weight	IV, Random, 95% CI
Abreu et al. 2016	4.15	0.99	30	3.77	0.71	30	0.0%	0.38 [−0.06, 0.82]
Ahuja et al. 2019	6.01	0.38	30	5.35	0.55	30	0.0%	0.66 [0.42, 0.90]
Almeida et al. 2020	3.83	2.02	26	2.36	1.57	28	0.0%	1.47 [0.50, 2.44]
Camargo et al. 2013	3.71	0.62	10	3.3	0.9	10	0.0%	0.41 [−0.27, 1.09]
Christgau et al. 1998	4.3	2	20	4.3	1.9	20	3.1%	0.00 [−1.21, 1.21]
Cirano et al. 2012	6	0.7	16	6.3	0.7	15	0.0%	−0.30 [−0.79, 0.19]
Correa et al. 2008	5.4	1.3	23	4.5	1.1	26	0.0%	0.90 [0.22, 1.58]
Da Cruz et al. 2008	4.49	0.7	10	4.03	0.37	10	0.0%	0.46 [−0.03, 0.95]
Dag et al. DM + 2009	4.25	0.82	15	4.31	0.59	8	8.0%	−0.06 [−0.64, 0.52]
Dag et al. DM – 2009	4.3	0.97	15	4.31	0.59	8	7.3%	−0.01 [−0.65, 0.63]
Dogan et al. 2016	4.31	0.36	20	4.25	0.35	20	13.7%	0.06 [−0.16, 0.28]
Gayathri et al. 2019	3.65	0.45	30	3.6	0.42	30	0.0%	0.05 [−0.17, 0.27]
Gomathi et al. 2023	4.36	0.82	15	4.38	0.65	15	0.0%	−0.02 [−0.55, 0.51]
Goncalvez /Silva et al.	4.97	1.45	20	4.25	1.01	20	0.0%	0.72 [−0.05, 1.49]
Kara et al. 2015	4.78	0.579	15	4.49	0.411	15	11.4%	0.29 [−0.07, 0.65]
Kardesler et al. DM+ 2009	4.3	0.9	13	4.3	0.7	8	6.7%	0.00 [−0.69, 0.69]
Kardesler et al. DM− 2009	4.4	1.1	12	4.3	0.7	8	5.7%	0.10 [−0.69, 0.89]
Kaur et al. 2015	3.46	0.53	50	3.14	0.53	25	0.0%	0.32 [0.07, 0.57]
Lopez et al. 2014	3.6	0.8	26	3.6	0.8	26	0.0%	0.00 [−0.43, 0.43]
Mirnic et al. DM + 2022	2.81	0.98	29	2.32	1.39	16	5.9%	0.49 [−0.28, 1.26]
Mirnic et al. DM – 2022	2.62	1.36	32	2.32	1.39	16	5.3%	0.30 [−0.53, 1.13]
Mishra et al. 2016	2.51	0.27	14	2.08	0.37	14	0.0%	0.43 [0.19, 0.67]
Navarro-Sanchez /Faria	5.7	1.9	8	4.9	0.8	10	2.4%	0.80 [−0.61, 2.21]
Pannicker et al. 2016	11.97	2.43	35	9.82	1.31	35	0.0%	2.15 [1.24, 3.06]
Peruzzo Lopes DM1 2017	2.62	0.09	14	2.49	0.06	6	15.4%	0.13 [0.06, 0.20]
Peruzzo Lopes DM2 2017	3.1	0.12	27	2.49	0.06	6	15.4%	0.61 [0.54, 0.68]
Pragada et al. 2019	10.07	1.63	30	9.67	1.51	30	0.0%	0.40 [−0.40, 1.20]
Sundaram et al. DM+ 2017	4.36	0.67	40	4.13	0.79	20	0.0%	0.23 [−0.17, 0.63]
Sundaram et al. DM− 2017	4.03	0.47	40	4.13	0.79	20	0.0%	−0.10 [−0.48, 0.28]
Total (95% CI)			220			141	100.0%	0.22 [−0.02, 0.45]

Heterogeneity: $Tau^2 = 0.09$; $Chi^2 = 112.83$, df = 11 ($P < 0.00001$); $I^2 = 90\%$

Test for overall effect: $Z = 1.82$ ($P = 0.07$)

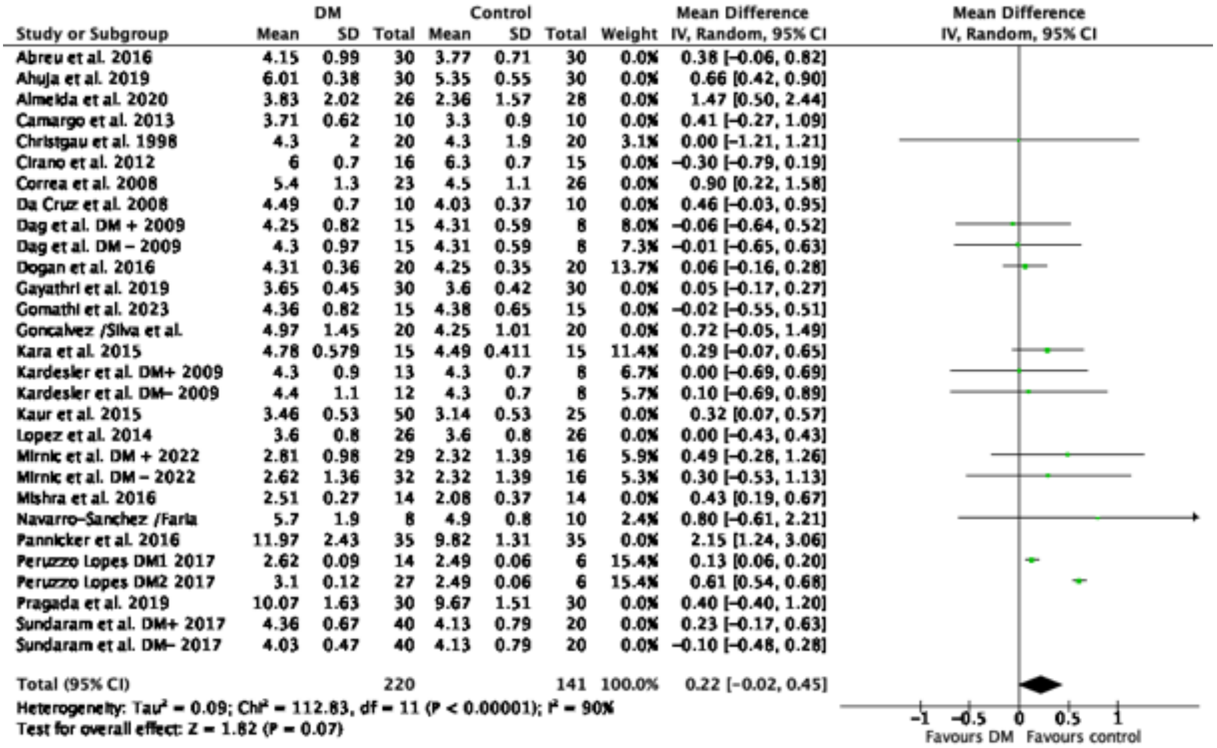

Online Appendix S7.50

Meta-analysis evaluating the effect of NSPT on DM compared to non-DM regarding CAL on Europe post NSPT

Study or Subgroup	DM Mean	DM SD	DM Total	Control Mean	Control SD	Control Total	Weight	Mean Difference IV, Random, 95% CI
Abreu et al. 2016	3.58	0.92	30	3.1	1.12	30	0.0%	0.48 [−0.04, 1.00]
Ahuja et al. 2019	3.75	0.86	30	2.81	0.78	30	0.0%	0.94 [0.52, 1.36]
Almeida et al. 2020	3.52	1.91	26	2.11	1.2	28	0.0%	1.41 [0.55, 2.27]
Camargo et al. 2013	2.85	0.66	10	2.92	0.48	10	0.0%	−0.07 [−0.58, 0.44]
Christgau et al. 1998	4.1	1.8	20	4.1	1.8	20	5.6%	0.00 [−1.12, 1.12]
Cirano et al. 2012	3.9	0.8	16	4.2	0.9	15	0.0%	−0.30 [−0.90, 0.30]
Correa et al. 2008	4.6	1.1	23	4	0.8	26	0.0%	0.60 [0.06, 1.14]
Da Cruz et al. 2008	3.28	0.54	10	2.9	0.26	10	0.0%	0.38 [0.01, 0.75]
Dag et al. DM + 2009	3.03	0.79	15	2.91	0.56	8	8.8%	0.12 [−0.44, 0.68]
Dag et al. DM − 2009	3.04	0.81	15	2.91	0.56	8	8.8%	0.13 [−0.43, 0.69]
Dogan et al. 2016	3.09	0.43	20	2.88	0.38	20	10.4%	0.21 [−0.04, 0.46]
Gayathri et al. 2019	3.61	0.48	30	3.47	0.58	30	0.0%	0.14 [−0.13, 0.41]
Gomathi et al. 2023	3.03	0.19	15	3.05	0.15	15	0.0%	−0.02 [−0.14, 0.10]
Goncalvez /Silva et al.	4.34	1.29	20	3.97	0.83	20	0.0%	0.37 [−0.30, 1.04]
Kara et al. 2015	3.06	0.726	15	2.75	0.498	15	9.5%	0.31 [−0.14, 0.76]
Kardesler et al. DM+ 2009	3.9	0.9	13	3.9	0.7	8	8.0%	0.00 [−0.69, 0.69]
Kardesler et al. DM− 2009	4.2	0.9	12	3.9	0.7	8	7.9%	0.30 [−0.40, 1.00]
Kaur et al. 2015	2.75	0.62	50	2.41	0.55	25	0.0%	0.34 [0.06, 0.62]
Lopez et al. 2014	3.57	0.99	24	3.43	0.74	26	0.0%	0.14 [−0.35, 0.63]
Mirnic et al. DM + 2022	2.57	1.01	29	1.98	1.31	16	7.7%	0.59 [−0.15, 1.33]
Mirnic et al. DM − 2022	2.39	1.27	32	1.98	1.31	16	7.5%	0.41 [−0.37, 1.19]
Mishra et al. 2016	1.63	0.24	14	1.41	0.27	14	0.0%	0.22 [0.03, 0.41]
Navarro-Sanchez /Faria	5.2	2	8	4.5	0.8	10	4.1%	0.70 [−0.77, 2.17]
Pannicker et al. 2016	9.85	2.39	35	7.48	1.22	35	0.0%	2.37 [1.48, 3.26]
Peruzzo Lopes DM1 2017	1.56	0.04	14	1.82	0.04	6	10.9%	−0.26 [−0.30, −0.22]
Peruzzo Lopes DM2 2017	2.44	0.12	27	1.82	0.04	6	10.9%	0.62 [0.56, 0.68]
Pragada et al. 2019	7.53	1.04	30	7.1	1.21	30	0.0%	0.43 [−0.14, 1.00]
Sundaram et al. DM+ 2017	3.06	1.05	40	2.97	0.99	20	0.0%	0.09 [−0.45, 0.63]
Sundaram et al. DM− 2017	2.73	1.12	40	2.97	0.99	20	0.0%	−0.24 [−0.80, 0.32]
Total (95% CI)			220			141	100.0%	0.24 [−0.14, 0.62]

Heterogeneity: $Tau^2 = 0.34$; $Chi^2 = 664.08$, df = 11 ($P < 0.00001$); $I^2 = 98\%$

Test for overall effect: Z = 1.25 (P = 0.21)

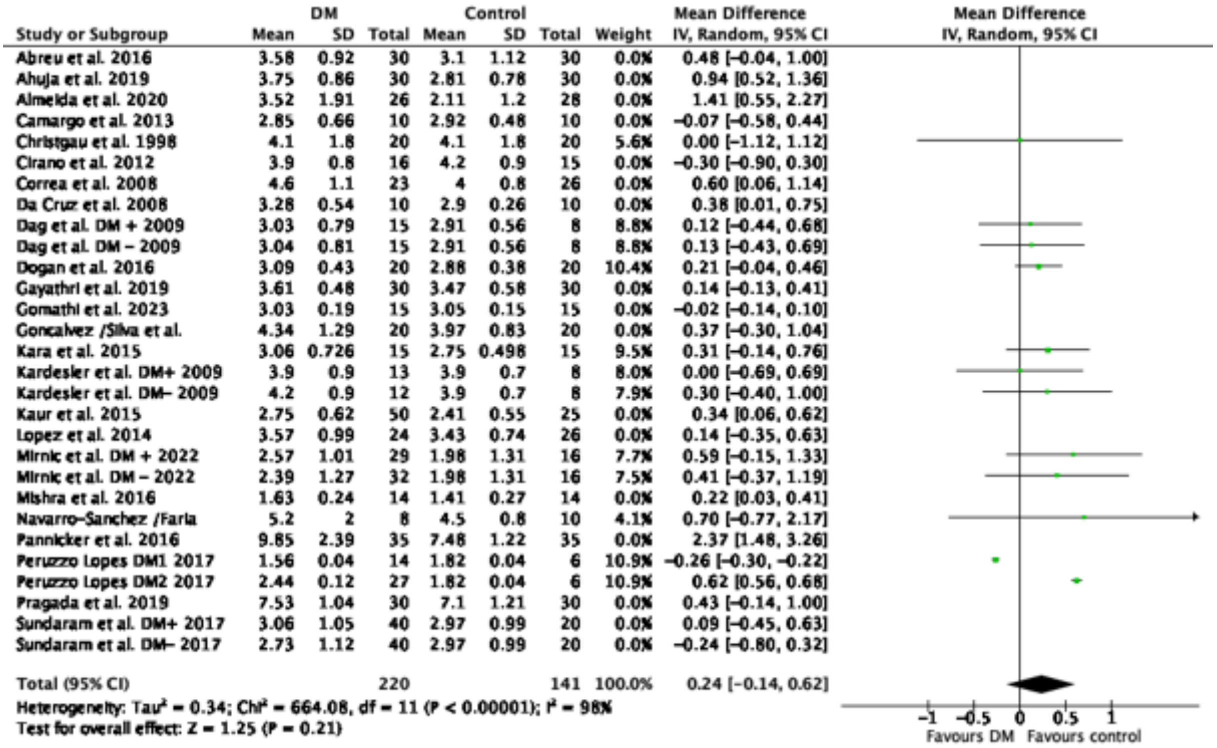

Online Appendix S7.51

Meta-analysis evaluating the effect of NSPT on DM compared to non-DM regarding CAL on Europe on incremental scores

Study or Subgroup	DM Mean	DM SD	DM Total	Control Mean	Control SD	Control Total	Weight	Mean Difference IV, Random, 95% CI
Abreu et al. 2016	−1.28	1.63	30	−0.64	1.09	30	0.0%	−0.64 [−1.34, 0.06]
Ahuja et al. 2019	−2.25	0.67	30	−2.55	0.25	30	0.0%	0.30 [0.04, 0.56]
Almeida et al. 2020	−0.3	0.79	26	−0.25	0.74	28	0.0%	−0.05 [−0.46, 0.36]
Camargo et al. 2013	−0.85	0.28	10	−0.37	0.63	10	0.0%	−0.48 [−0.91, −0.05]
Christgau et al. 1998	−0.2	1.1	20	−0.2	1.1	20	6.1%	0.00 [−0.68, 0.68]
Correa et al. 2008	−0.8	0.6	23	−0.6	0.6	26	0.0%	−0.20 [−0.54, 0.14]
Da Cruz et al. 2008	−1.21	0.24	10	−1.12	0.15	10	0.0%	−0.09 [−0.27, 0.09]
Goncalvez /Silva et al.	−0.63	0.43	20	−0.46	0.4	20	0.0%	−0.17 [−0.43, 0.09]
Kardesler et al. DM+ 2009	−0.3	0.5	13	−0.4	0.6	8	8.6%	0.10 [−0.40, 0.60]
Kardesler et al. DM− 2009	−0.2	0.5	12	−0.4	0.6	8	8.5%	0.20 [−0.30, 0.70]
Kaur et al. 2015	−0.71	0.36	50	−0.73	0.31	25	0.0%	0.02 [−0.14, 0.18]
Lopez et al. 2014	−0.03	0.19	24	−0.17	0.06	26	0.0%	0.14 [0.06, 0.22]
Mirnic et al. DM + 2022	−0.24	0.24	29	−0.34	0.3	16	14.8%	0.10 [−0.07, 0.27]
Mirnic et al. DM − 2022	−0.23	0.27	32	−0.34	0.3	16	14.8%	0.11 [−0.06, 0.28]
Mishra et al. 2016	−0.88	0.13	14	−0.66	0.31	14	0.0%	−0.22 [−0.40, −0.04]
Navarro-Sanchez /Faria	−0.5	0.2	8	−0.4	0.2	10	14.6%	−0.10 [−0.29, 0.09]
Peruzzo Lopes DM1 2017	−1.06	0.04	14	−0.67	0.04	6	16.3%	−0.39 [−0.43, −0.35]
Peruzzo Lopes DM2 2017	−0.66	0.08	27	−0.67	0.04	6	16.3%	0.01 [−0.03, 0.05]
Total (95% CI)			155			90	100.0%	−0.02 [−0.23, 0.19]

Heterogeneity: $Tau^2 = 0.07$; $Chi^2 = 211.49$, df = 7 ($P < 0.00001$); $I^2 = 97\%$

Test for overall effect: Z = 0.18 (P = 0.86)

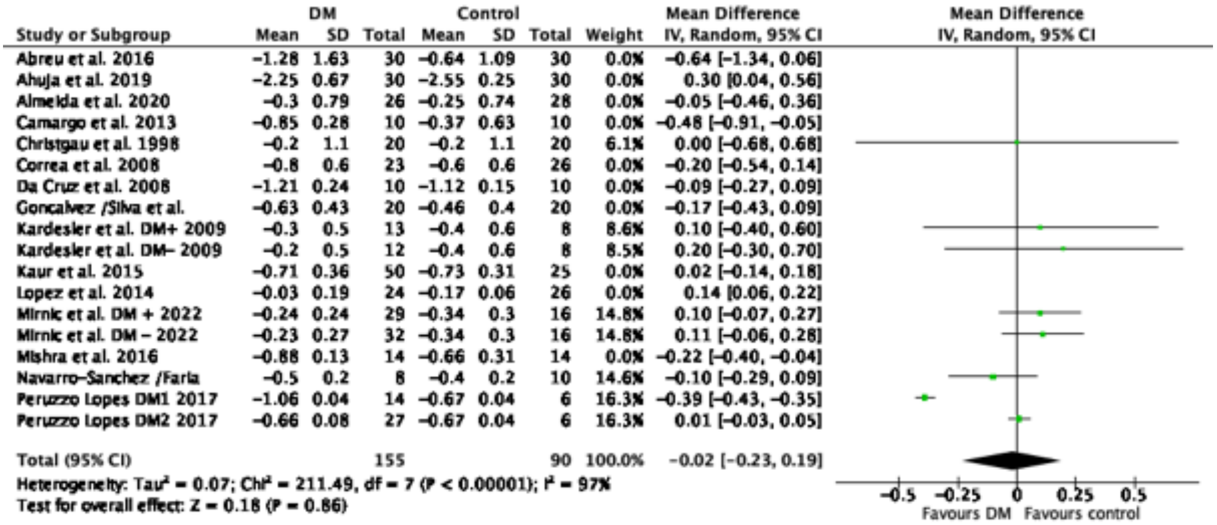

Online Appendix S7.52

Meta-analysis evaluating the effect of NSPT on DM compared to non-DM regarding CAL on Asia at baseline

Study or Subgroup	DM Mean	DM SD	DM Total	Control Mean	Control SD	Control Total	Weight	Mean Difference IV, Random, 95% CI
Abreu et al. 2016	4.15	0.99	30	3.77	0.71	30	0.0%	0.38 [−0.06, 0.82]
Ahuja et al. 2019	6.01	0.38	30	5.35	0.55	30	16.6%	0.66 [0.42, 0.90]
Almeida et al. 2020	3.83	2.02	26	2.36	1.57	28	0.0%	1.47 [0.50, 2.44]
Camargo et al. 2013	3.71	0.62	10	3.3	0.9	10	0.0%	0.41 [−0.27, 1.09]
Christgau et al. 1998	4.3	2	20	4.3	1.9	20	0.0%	0.00 [−1.21, 1.21]
Cirano et al. 2012	6	0.7	16	6.3	0.7	15	0.0%	−0.30 [−0.79, 0.19]
Correa et al. 2008	5.4	1.3	23	4.5	1.1	26	0.0%	0.90 [0.22, 1.58]
Da Cruz et al. 2008	4.49	0.7	10	4.03	0.37	10	0.0%	0.46 [−0.03, 0.95]
Dag et al. DM + 2009	4.25	0.82	15	4.31	0.59	8	0.0%	−0.06 [−0.64, 0.52]
Dag et al. DM − 2009	4.3	0.97	15	4.31	0.59	8	0.0%	−0.01 [−0.65, 0.63]
Dogan et al. 2016	4.31	0.36	20	4.25	0.35	20	0.0%	0.06 [−0.16, 0.28]
Gayathri et al. 2019	3.65	0.45	30	3.6	0.42	30	17.2%	0.05 [−0.17, 0.27]
Gomathi et al. 2023	4.36	0.82	15	4.38	0.65	15	9.0%	−0.02 [−0.55, 0.51]
Goncalvez /Silva et al.	4.97	1.45	20	4.25	1.01	20	0.0%	0.72 [−0.05, 1.49]
Kara et al. 2015	4.78	0.579	15	4.49	0.411	15	0.0%	0.29 [−0.07, 0.65]
Kardesler et al. DM+ 2009	4.3	0.9	13	4.3	0.7	8	0.0%	0.00 [−0.69, 0.69]
Kardesler et al. DM− 2009	4.4	1.1	12	4.3	0.7	8	0.0%	0.10 [−0.69, 0.89]
Kaur et al. 2015	3.46	0.53	50	3.14	0.53	25	16.2%	0.32 [0.07, 0.57]
Lopez et al. 2014	3.6	0.8	26	3.6	0.8	26	0.0%	0.00 [−0.43, 0.43]
Mirnic et al. DM + 2022	2.81	0.98	29	2.32	1.39	16	0.0%	0.49 [−0.28, 1.26]
Mirnic et al. DM − 2022	2.62	1.36	32	2.32	1.39	16	0.0%	0.30 [−0.53, 1.13]
Mishra et al. 2016	2.51	0.27	14	2.08	0.37	14	16.6%	0.43 [0.19, 0.67]
Navarro-Sanchez /Faria	5.7	1.9	8	4.9	0.8	10	0.0%	0.80 [−0.61, 2.21]
Pannicker et al. 2016	11.97	2.43	35	9.82	1.31	35		Not estimable
Peruzzo Lopes DM1 2017	2.62	0.09	14	2.49	0.06	6	0.0%	0.13 [0.06, 0.20]
Peruzzo Lopes DM2 2017	3.1	0.12	27	2.49	0.06	6	0.0%	0.61 [0.54, 0.68]
Pragada et al. 2019	10.07	1.63	30	9.67	1.51	30		Not estimable
Sundaram et al. DM+ 2017	4.36	0.67	40	4.13	0.79	20	11.8%	0.23 [−0.17, 0.63]
Sundaram et al. DM− 2017	4.03	0.47	40	4.13	0.79	20	12.6%	−0.10 [−0.48, 0.28]
Total (95% CI)			219			154	100.0%	0.25 [0.05, 0.46]

Heterogeneity: $Tau^2 = 0.05$; $Chi^2 = 20.65$, df = 6 (P = 0.002); $I^2 = 71\%$
Test for overall effect: Z = 2.39 (P = 0.02)

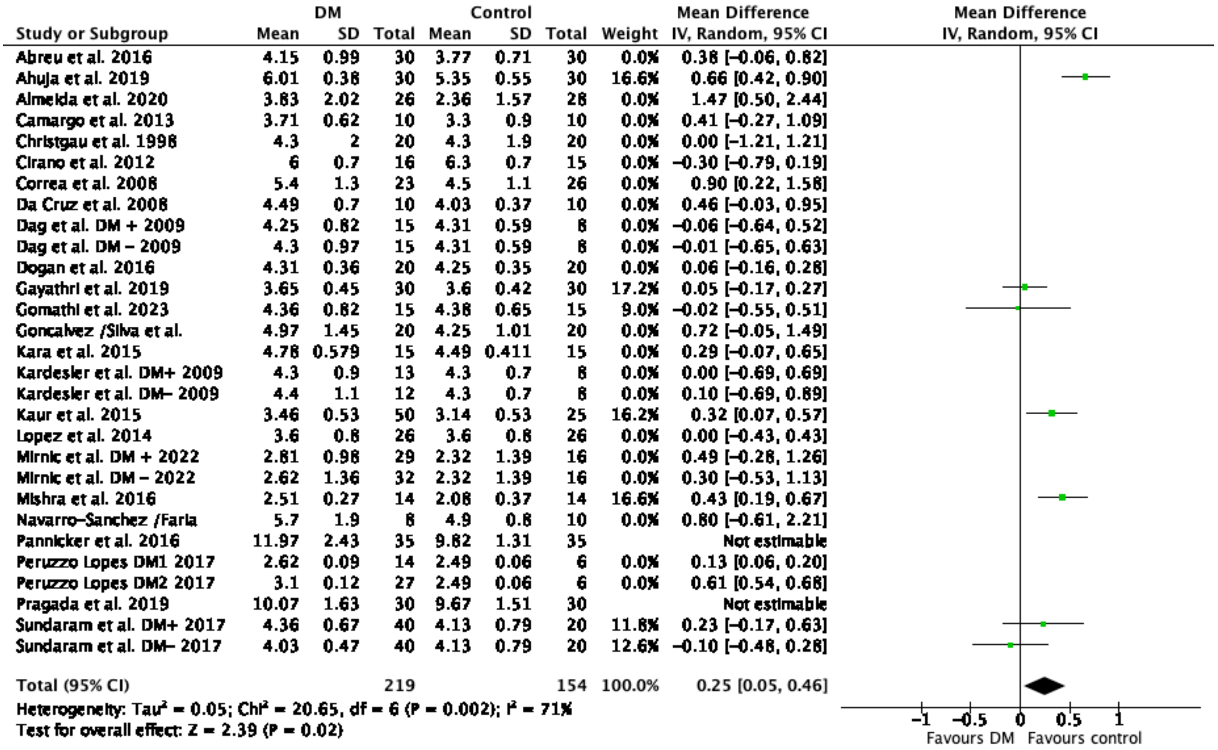

Online Appendix S7.53

Meta-analysis evaluating the effect of NSPT on DM compared to non-DM regarding CAL on Asia post NSPT

Study or Subgroup	DM Mean	DM SD	DM Total	Control Mean	Control SD	Control Total	Weight	Mean Difference IV, Random, 95% CI
Abreu et al. 2016	3.58	0.92	30	3.1	1.12	30	0.0%	0.48 [−0.04, 1.00]
Ahuja et al. 2019	3.75	0.86	30	2.81	0.78	30	11.8%	0.94 [0.52, 1.36]
Almeida et al. 2020	3.52	1.91	26	2.11	1.2	28	0.0%	1.41 [0.55, 2.27]
Camargo et al. 2013	2.85	0.66	10	2.92	0.48	10	0.0%	−0.07 [−0.58, 0.44]
Christgau et al. 1998	4.1	1.8	20	4.1	1.8	20	0.0%	0.00 [−1.12, 1.12]
Cirano et al. 2012	3.9	0.8	16	4.2	0.9	15	0.0%	−0.30 [−0.90, 0.30]
Correa et al. 2008	4.6	1.1	23	4	0.8	26	0.0%	0.60 [0.06, 1.14]
Da Cruz et al. 2008	3.28	0.54	10	2.9	0.26	10	0.0%	0.38 [0.01, 0.75]
Dag et al. DM + 2009	3.03	0.79	15	2.91	0.56	8	0.0%	0.12 [−0.44, 0.68]
Dag et al. DM − 2009	3.04	0.81	15	2.91	0.56	8	0.0%	0.13 [−0.43, 0.69]
Dogan et al. 2016	3.09	0.43	20	2.88	0.38	20	0.0%	0.21 [−0.04, 0.46]
Gayathri et al. 2019	3.61	0.48	30	3.47	0.58	30	16.0%	0.14 [−0.13, 0.41]
Gomathi et al. 2023	3.03	0.19	15	3.05	0.15	15	20.2%	−0.02 [−0.14, 0.10]
Goncalvez /Silva et al.	4.34	1.29	20	3.97	0.83	20	0.0%	0.37 [−0.30, 1.04]
Kara et al. 2015	3.06	0.726	15	2.75	0.498	15	0.0%	0.31 [−0.14, 0.76]
Kardesler et al. DM+ 2009	3.9	0.9	13	3.9	0.7	8	0.0%	0.00 [−0.69, 0.69]
Kardesler et al. DM− 2009	4.2	0.9	12	3.9	0.7	8	0.0%	0.30 [−0.40, 1.00]
Kaur et al. 2015	2.75	0.62	50	2.41	0.55	25	15.8%	0.34 [0.06, 0.62]
Lopez et al. 2014	3.57	0.99	24	3.43	0.74	26	0.0%	0.14 [−0.35, 0.63]
Mirnic et al. DM + 2022	2.57	1.01	29	1.98	1.31	16	0.0%	0.59 [−0.15, 1.33]
Mirnic et al. DM − 2022	2.39	1.27	32	1.98	1.31	16	0.0%	0.41 [−0.37, 1.19]
Mishra et al. 2016	1.63	0.24	14	1.41	0.27	14	18.4%	0.22 [0.03, 0.41]
Navarro-Sanchez /Faria	5.2	2	8	4.5	0.8	10	0.0%	0.70 [−0.77, 2.17]
Pannicker et al. 2016	9.85	2.39	35	7.48	1.22	35		Not estimable
Peruzzo Lopes DM1 2017	1.56	0.04	14	1.82	0.04	6	0.0%	−0.26 [−0.30, −0.22]
Peruzzo Lopes DM2 2017	2.44	0.12	27	1.82	0.04	6	0.0%	0.62 [0.56, 0.68]
Pragada et al. 2019	7.53	1.04	30	7.1	1.21	30		Not estimable
Sundaram et al. DM+ 2017	3.06	1.05	40	2.97	0.99	20	9.0%	0.09 [−0.45, 0.63]
Sundaram et al. DM− 2017	2.73	1.12	40	2.97	0.99	20	8.7%	−0.24 [−0.80, 0.32]
Total (95% CI)			219			154	100.0%	0.21 [−0.00, 0.42]

Heterogeneity: $Tau^2 = 0.05$; $Chi^2 = 25.15$, df = 6 (P = 0.0003); $I^2 = 76\%$
Test for overall effect: Z = 1.95 (P = 0.05)

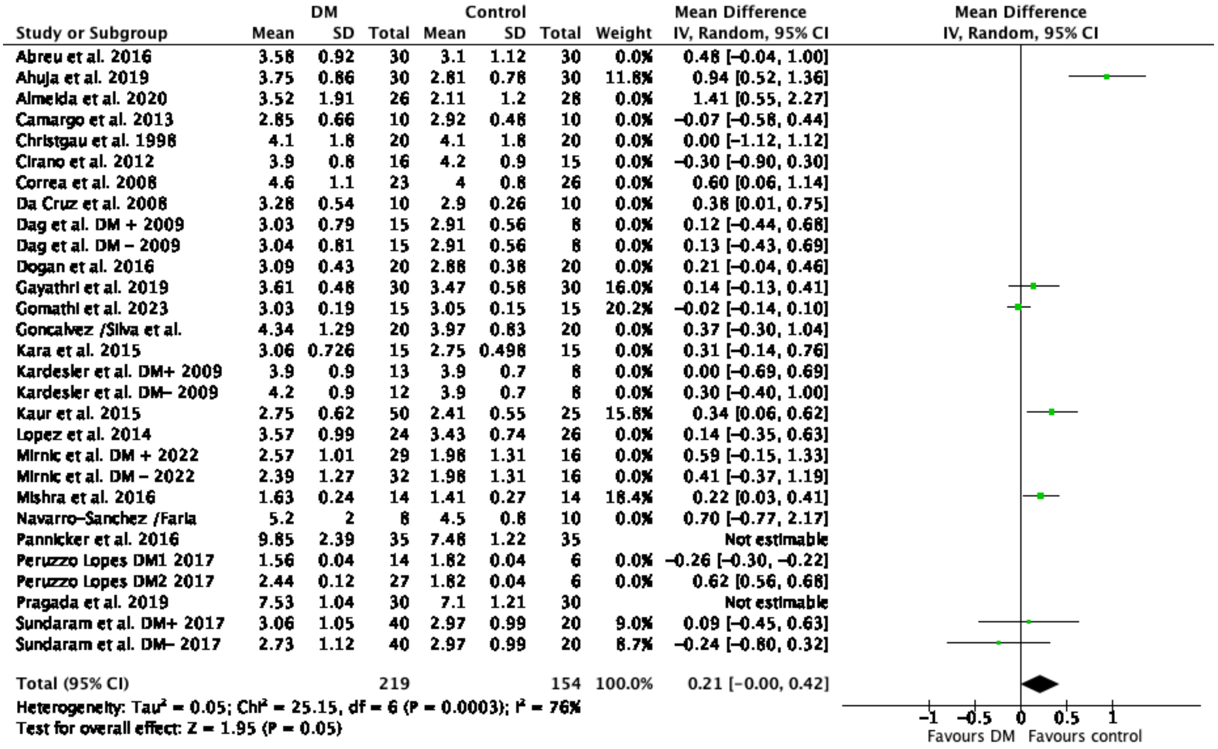

Online Appendix S7.54

Meta-analysis evaluating the effect of NSPT on DM compared to non-DM regarding CAL on Asia on incremental scores

	DM			Control				Mean Difference
Study or Subgroup	Mean	SD	Total	Mean	SD	Total	Weight	IV, Fixed, 95% CI
Abreu et al. 2016	−1.28	1.63	30	−0.64	1.09	30	0.0%	−0.64 [−1.34, 0.06]
Ahuja et al. 2019	−2.25	0.67	30	−2.55	0.25	30	17.4%	0.30 [0.04, 0.56]
Almeida et al. 2020	−0.3	0.79	26	−0.25	0.74	28	0.0%	−0.05 [−0.46, 0.36]
Camargo et al. 2013	−0.85	0.28	10	−0.37	0.63	10	0.0%	−0.48 [−0.91, −0.05]
Christgau et al. 1998	−0.2	1.1	20	−0.2	1.1	20	0.0%	0.00 [−0.68, 0.68]
Correa et al. 2008	−0.8	0.6	23	−0.6	0.6	26	0.0%	−0.20 [−0.54, 0.14]
Da Cruz et al. 2008	−1.21	0.24	10	−1.12	0.15	10	0.0%	−0.09 [−0.27, 0.09]
Goncalvez /Silva et al.	−0.63	0.43	20	−0.46	0.4	20	0.0%	−0.17 [−0.43, 0.09]
Kardesler et al. DM+ 2009	−0.3	0.5	13	−0.4	0.6	8	0.0%	0.10 [−0.40, 0.60]
Kardesler et al. DM− 2009	−0.2	0.5	12	−0.4	0.6	8	0.0%	0.20 [−0.30, 0.70]
Kaur et al. 2015	−0.71	0.36	50	−0.73	0.31	25	46.0%	0.02 [−0.14, 0.18]
Lopez et al. 2014	−0.03	0.19	24	−0.17	0.06	26	0.0%	0.14 [0.06, 0.22]
Mirnic et al. DM + 2022	−0.24	0.24	29	−0.34	0.3	16	0.0%	0.10 [−0.07, 0.27]
Mirnic et al. DM − 2022	−0.23	0.27	32	−0.34	0.3	16	0.0%	0.11 [−0.06, 0.28]
Mishra et al. 2016	−0.88	0.13	14	−0.66	0.31	14	36.7%	−0.22 [−0.40, −0.04]
Navarro-Sanchez /Faria	−0.5	0.2	8	−0.4	0.2	10	0.0%	−0.10 [−0.29, 0.09]
Peruzzo Lopes DM1 2017	−1.06	0.04	14	−0.67	0.04	6	0.0%	−0.39 [−0.43, −0.35]
Peruzzo Lopes DM2 2017	−0.66	0.08	27	−0.67	0.04	6	0.0%	0.01 [−0.03, 0.05]
Total (95% CI)			94			69	100.0%	−0.02 [−0.13, 0.09]

Heterogeneity: $Chi^2 = 11.21$, df = 2 (P = 0.004); $I^2 = 82\%$

Test for overall effect: Z = 0.36 (P = 0.72)

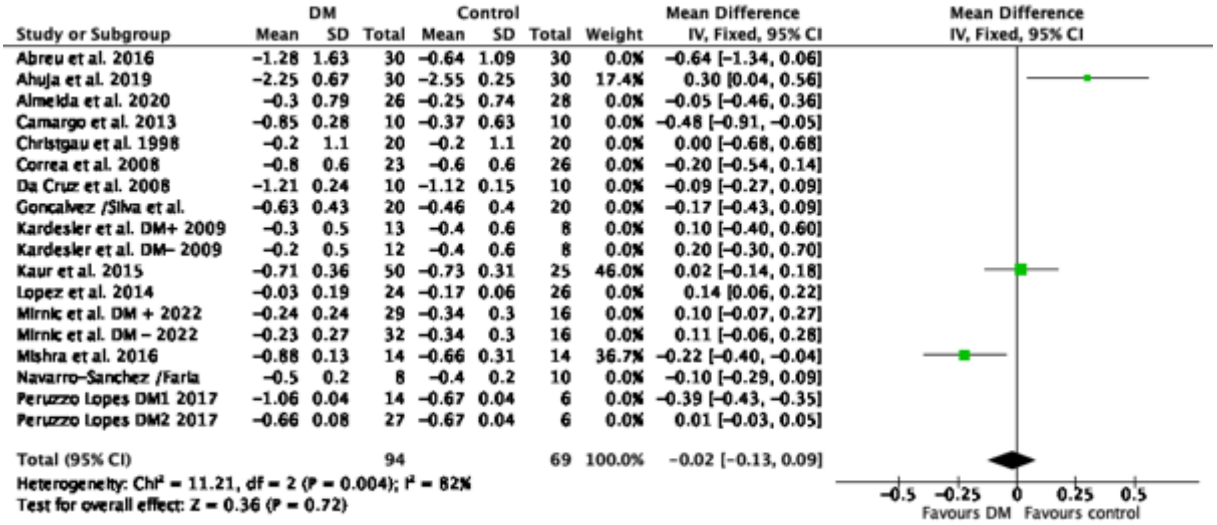

Online Appendix S7.55

Meta-analysis evaluating the effect of NSPT on DM compared to non-DM regarding CAL on South America at baseline

	DM			Control				Mean Difference
Study or Subgroup	Mean	SD	Total	Mean	SD	Total	Weight	IV, Random, 95% CI
Abreu et al. 2016	4.15	0.99	30	3.77	0.71	30	15.6%	0.38 [−0.06, 0.82]
Ahuja et al. 2019	6.01	0.38	30	5.35	0.55	30	0.0%	0.66 [0.42, 0.90]
Almeida et al. 2020	3.83	2.02	26	2.36	1.57	28	7.5%	1.47 [0.50, 2.44]
Camargo et al. 2013	3.71	0.62	10	3.3	0.9	10	11.2%	0.41 [−0.27, 1.09]
Christgau et al. 1998	4.3	2	20	4.3	1.9	20	0.0%	0.00 [−1.21, 1.21]
Cirano et al. 2012	6	0.7	16	6.3	0.7	15	14.5%	−0.30 [−0.79, 0.19]
Correa et al. 2008	5.4	1.3	23	4.5	1.1	26	11.2%	0.90 [0.22, 1.58]
Da Cruz et al. 2008	4.49	0.7	10	4.03	0.37	10	14.6%	0.46 [−0.03, 0.95]
Dag et al. DM + 2009	4.25	0.82	15	4.31	0.59	8	0.0%	−0.06 [−0.64, 0.52]
Dag et al. DM − 2009	4.3	0.97	15	4.31	0.59	8	0.0%	−0.01 [−0.65, 0.63]
Dogan et al. 2016	4.31	0.36	20	4.25	0.35	20	0.0%	0.06 [−0.16, 0.28]
Gayathri et al. 2019	3.65	0.45	30	3.6	0.42	30	0.0%	0.05 [−0.17, 0.27]
Gomathi et al. 2023	4.36	0.82	15	4.38	0.65	15	0.0%	−0.02 [−0.55, 0.51]
Goncalvez /Silva et al.	4.97	1.45	20	4.25	1.01	20	9.8%	0.72 [−0.05, 1.49]
Kara et al. 2015	4.78	0.579	15	4.49	0.411	15	0.0%	0.29 [−0.07, 0.65]
Kardesler et al. DM+ 2009	4.3	0.9	13	4.3	0.7	8	0.0%	0.00 [−0.69, 0.69]
Kardesler et al. DM− 2009	4.4	1.1	12	4.3	0.7	8	0.0%	0.10 [−0.69, 0.89]
Kaur et al. 2015	3.46	0.53	50	3.14	0.53	25	0.0%	0.32 [0.07, 0.57]
Lopez et al. 2014	3.6	0.8	26	3.6	0.8	26	15.7%	0.00 [−0.43, 0.43]
Mirnic et al. DM + 2022	2.81	0.98	29	2.32	1.39	16	0.0%	0.49 [−0.28, 1.26]
Mirnic et al. DM − 2022	2.62	1.36	32	2.32	1.39	16	0.0%	0.30 [−0.53, 1.13]
Mishra et al. 2016	2.51	0.27	14	2.08	0.37	14	0.0%	0.43 [0.19, 0.67]
Navarro-Sanchez /Faria	5.7	1.9	8	4.9	0.8	10	0.0%	0.80 [−0.61, 2.21]
Pannicker et al. 2016	11.97	2.43	35	9.82	1.31	35	0.0%	2.15 [1.24, 3.06]
Peruzzo Lopes DM1 2017	2.62	0.09	14	2.49	0.06	6	0.0%	0.13 [0.06, 0.20]
Peruzzo Lopes DM2 2017	3.1	0.12	27	2.49	0.06	6	0.0%	0.61 [0.54, 0.68]
Pragada et al. 2019	10.07	1.63	30	9.67	1.51	30	0.0%	0.40 [−0.40, 1.20]
Sundaram et al. DM+ 2017	4.36	0.67	40	4.13	0.79	20	0.0%	0.23 [−0.17, 0.63]
Sundaram et al. DM− 2017	4.03	0.47	40	4.13	0.79	20	0.0%	−0.10 [−0.48, 0.28]
Total (95% CI)			161			165	100.0%	0.41 [0.08, 0.74]

Heterogeneity: $Tau^2 = 0.13$; $Chi^2 = 17.83$, df = 7 (P = 0.01); $I^2 = 61\%$

Test for overall effect: Z = 2.45 (P = 0.01)

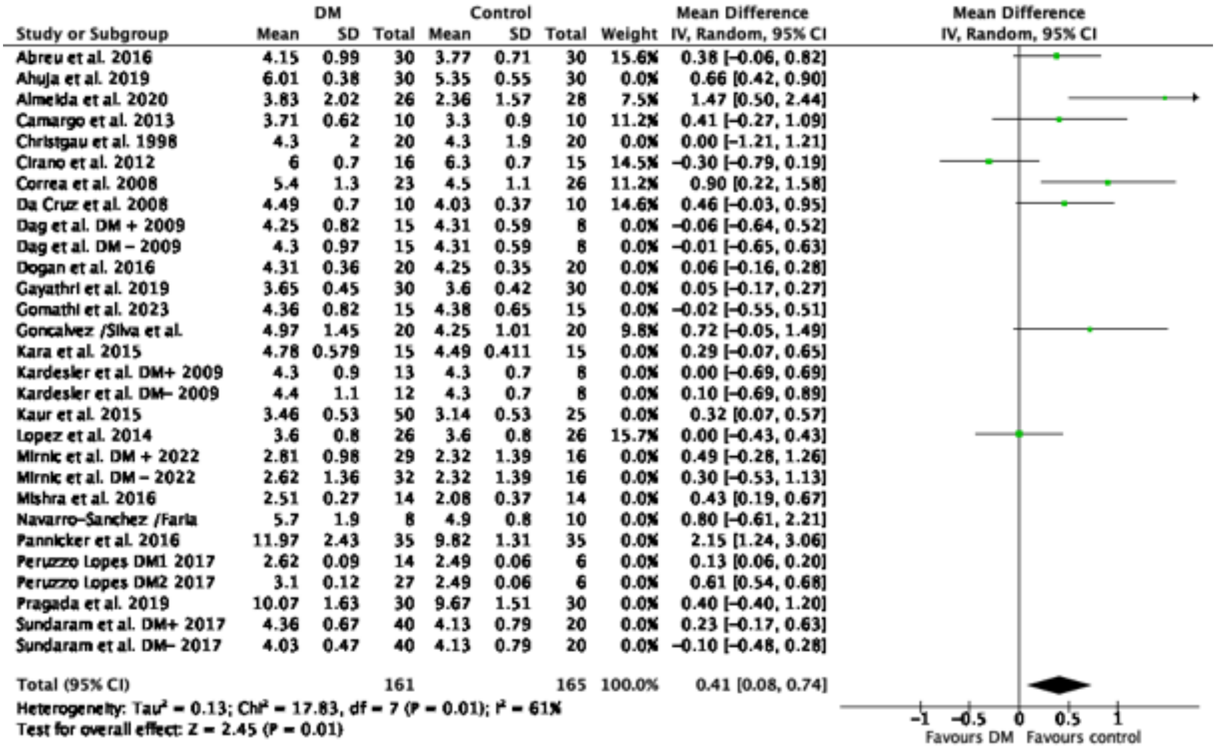

Online Appendix S7.56

Meta-analysis evaluating the effect of NSPT on DM compared to non-DM regarding CAL on South America post NSPT

Study or Subgroup	DM Mean	DM SD	DM Total	Control Mean	Control SD	Control Total	Weight	Mean Difference IV, Random, 95% CI
Abreu et al. 2016	3.58	0.92	30	3.1	1.12	30	13.3%	0.48 [−0.04, 1.00]
Ahuja et al. 2019	3.75	0.86	30	2.81	0.78	30	0.0%	0.94 [0.52, 1.36]
Almeida et al. 2020	3.52	1.91	26	2.11	1.2	28	7.4%	1.41 [0.55, 2.27]
Camargo et al. 2013	2.85	0.66	10	2.92	0.48	10	13.6%	−0.07 [−0.58, 0.44]
Christgau et al. 1998	4.1	1.8	20	4.1	1.8	20	0.0%	0.00 [−1.12, 1.12]
Cirano et al. 2012	3.9	0.8	16	4.2	0.9	15	11.5%	−0.30 [−0.90, 0.30]
Correa et al. 2008	4.6	1.1	23	4	0.8	26	12.7%	0.60 [0.06, 1.14]
Da Cruz et al. 2008	3.28	0.54	10	2.9	0.26	10	17.2%	0.38 [0.01, 0.75]
Dag et al. DM + 2009	3.03	0.79	15	2.91	0.56	8	0.0%	0.12 [−0.44, 0.68]
Dag et al. DM − 2009	3.04	0.81	15	2.91	0.56	8	0.0%	0.13 [−0.43, 0.69]
Dogan et al. 2016	3.09	0.43	20	2.88	0.38	20	0.0%	0.21 [−0.04, 0.46]
Gayathri et al. 2019	3.61	0.48	30	3.47	0.58	30	0.0%	0.14 [−0.13, 0.41]
Gomathi et al. 2023	3.03	0.19	15	3.05	0.15	15	0.0%	−0.02 [−0.14, 0.10]
Goncalvez /Silva et al.	4.34	1.29	20	3.97	0.83	20	10.1%	0.37 [−0.30, 1.04]
Kara et al. 2015	3.06	0.726	15	2.75	0.498	15	0.0%	0.31 [−0.14, 0.76]
Kardesler et al. DM+ 2009	3.9	0.9	13	3.9	0.7	8	0.0%	0.00 [−0.69, 0.69]
Kardesler et al. DM− 2009	4.2	0.9	12	3.9	0.7	8	0.0%	0.30 [−0.40, 1.00]
Kaur et al. 2015	2.75	0.62	50	2.41	0.55	25	0.0%	0.34 [0.06, 0.62]
Lopez et al. 2014	3.57	0.99	24	3.43	0.74	26	14.1%	0.14 [−0.35, 0.63]
Mirnic et al. DM + 2022	2.57	1.01	29	1.98	1.31	16	0.0%	0.59 [−0.15, 1.33]
Mirnic et al. DM − 2022	2.39	1.27	32	1.98	1.31	16	0.0%	0.41 [−0.37, 1.19]
Mishra et al. 2016	1.63	0.24	14	1.41	0.27	14	0.0%	0.22 [0.03, 0.41]
Navarro-Sanchez /Faria	5.2	2	8	4.5	0.8	10	0.0%	0.70 [−0.77, 2.17]
Pannicker et al. 2016	9.85	2.39	35	7.48	1.22	35	0.0%	2.37 [1.48, 3.26]
Peruzzo Lopes DM1 2017	1.56	0.04	14	1.82	0.04	6	0.0%	−0.26 [−0.30, −0.22]
Peruzzo Lopes DM2 2017	2.44	0.12	27	1.82	0.04	6	0.0%	0.62 [0.56, 0.68]
Pragada et al. 2019	7.53	1.04	30	7.1	1.21	30	0.0%	0.43 [−0.14, 1.00]
Sundaram et al. DM+ 2017	3.06	1.05	40	2.97	0.99	20	0.0%	0.09 [−0.45, 0.63]
Sundaram et al. DM− 2017	2.73	1.12	40	2.97	0.99	20	0.0%	−0.24 [−0.80, 0.32]
Total (95% CI)			159			165	100.0%	0.32 [0.05, 0.60]

Heterogeneity: $Tau^2 = 0.08$; $Chi^2 = 14.57$, df = 7 ($P = 0.04$); $I^2 = 52\%$

Test for overall effect: $Z = 2.28$ ($P = 0.02$)

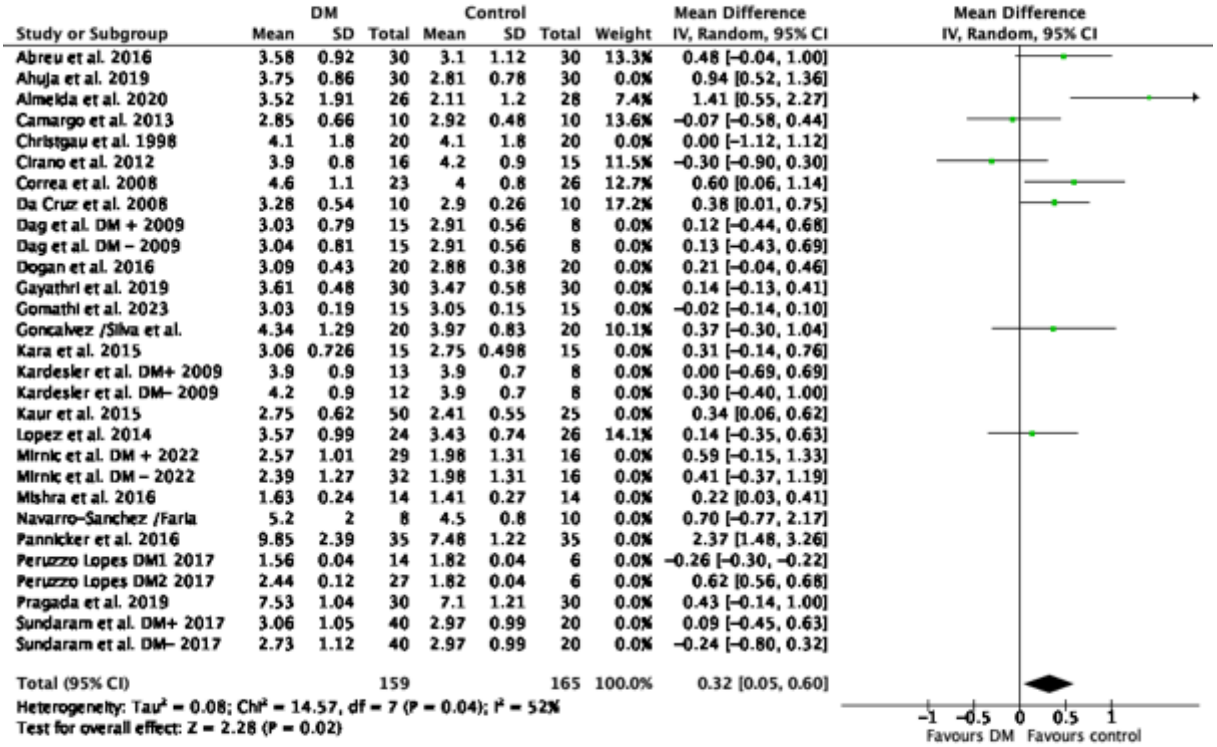

Online Appendix S7.57

Meta-analysis evaluating the effect of NSPT on DM compared to non-DM regarding CAL on South America on incremental scores

Study or Subgroup	DM Mean	DM SD	DM Total	Control Mean	Control SD	Control Total	Weight	Mean Difference IV, Random, 95% CI
Abreu et al. 2016	−1.28	1.63	30	−0.64	1.09	30	5.4%	−0.64 [−1.34, 0.06]
Ahuja et al. 2019	−2.25	0.67	30	−2.55	0.25	30	0.0%	0.30 [0.04, 0.56]
Almeida et al. 2020	−0.3	0.79	26	−0.25	0.74	28	11.1%	−0.05 [−0.46, 0.36]
Camargo et al. 2013	−0.85	0.28	10	−0.37	0.63	10	10.6%	−0.48 [−0.91, −0.05]
Christgau et al. 1998	−0.2	1.1	20	−0.2	1.1	20	0.0%	0.00 [−0.68, 0.68]
Correa et al. 2008	−0.8	0.6	23	−0.6	0.6	26	13.5%	−0.20 [−0.54, 0.14]
Da Cruz et al. 2008	−1.21	0.24	10	−1.12	0.15	10	19.8%	−0.09 [−0.27, 0.09]
Goncalvez /Silva et al.	−0.63	0.43	20	−0.46	0.4	20	16.5%	−0.17 [−0.43, 0.09]
Kardesler et al. DM+ 2009	−0.3	0.5	13	−0.4	0.6	8	0.0%	0.10 [−0.40, 0.60]
Kardesler et al. DM− 2009	−0.2	0.5	12	−0.4	0.6	8	0.0%	0.20 [−0.30, 0.70]
Kaur et al. 2015	−0.71	0.36	50	−0.73	0.31	25	0.0%	0.02 [−0.14, 0.18]
Lopez et al. 2014	−0.03	0.19	24	−0.17	0.06	26	23.0%	0.14 [0.06, 0.22]
Mirnic et al. DM + 2022	−0.24	0.24	29	−0.34	0.3	16	0.0%	0.10 [−0.07, 0.27]
Mirnic et al. DM − 2022	−0.23	0.27	32	−0.34	0.3	16	0.0%	0.11 [−0.06, 0.28]
Mishra et al. 2016	−0.88	0.13	14	−0.66	0.31	14	0.0%	−0.22 [−0.40, −0.04]
Navarro-Sanchez /Faria	−0.5	0.2	8	−0.4	0.2	10	0.0%	−0.10 [−0.29, 0.09]
Peruzzo Lopes DM1 2017	−1.06	0.04	14	−0.67	0.04	6	0.0%	−0.39 [−0.43, −0.35]
Peruzzo Lopes DM2 2017	−0.66	0.08	27	−0.67	0.04	6	0.0%	0.01 [−0.03, 0.05]
Total (95% CI)			143			150	100.0%	−0.13 [−0.32, 0.05]

Heterogeneity: $Tau^2 = 0.04$; $Chi^2 = 22.15$, df = 6 ($P = 0.001$); $I^2 = 73\%$

Test for overall effect: $Z = 1.39$ ($P = 0.17$)

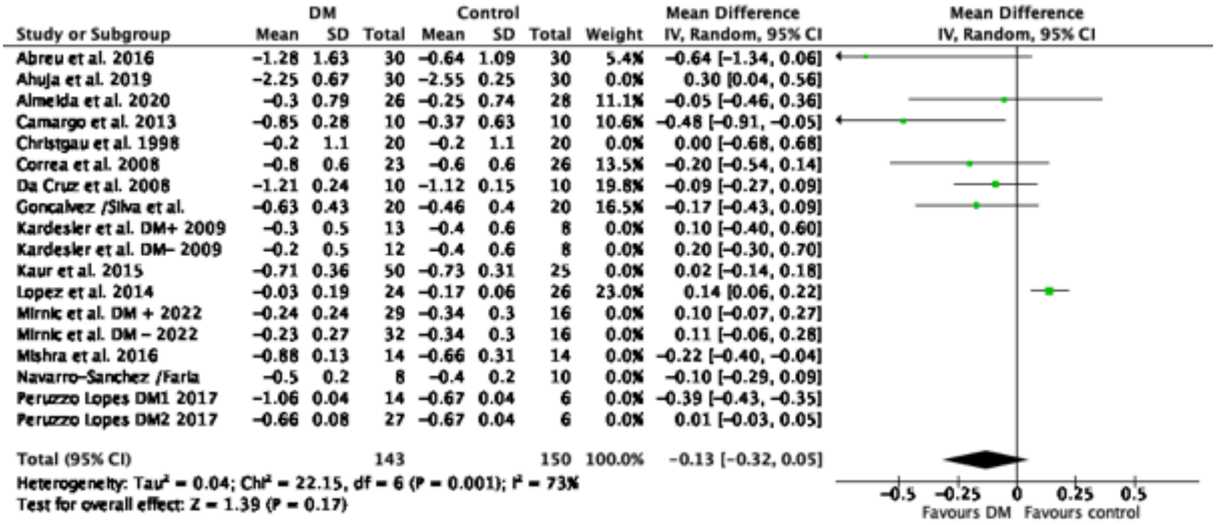

Online Appendix S8.1

Funnel plot regarding the effect of NSPT on DM compared to non-DM on PPD at baseline

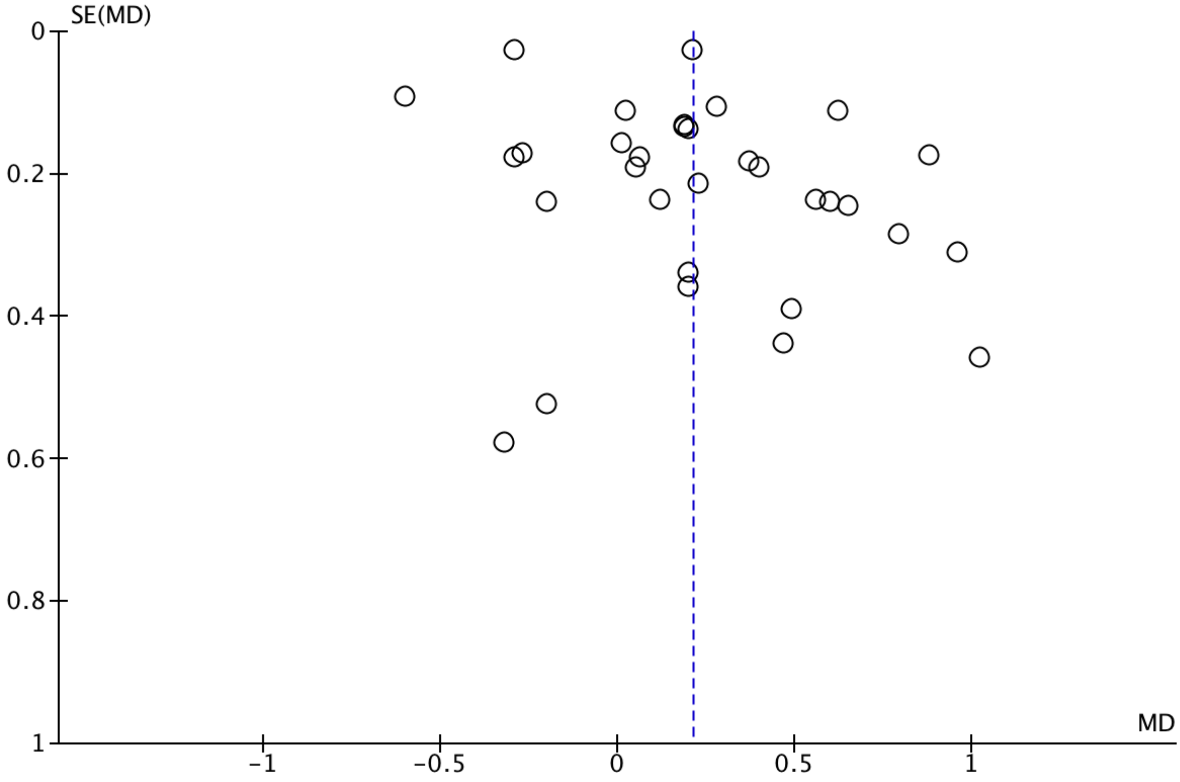

Online Appendix S8.2

Funnel plot regarding the effect of NSPT on DM compared to non-DM on PPD post-NSPT

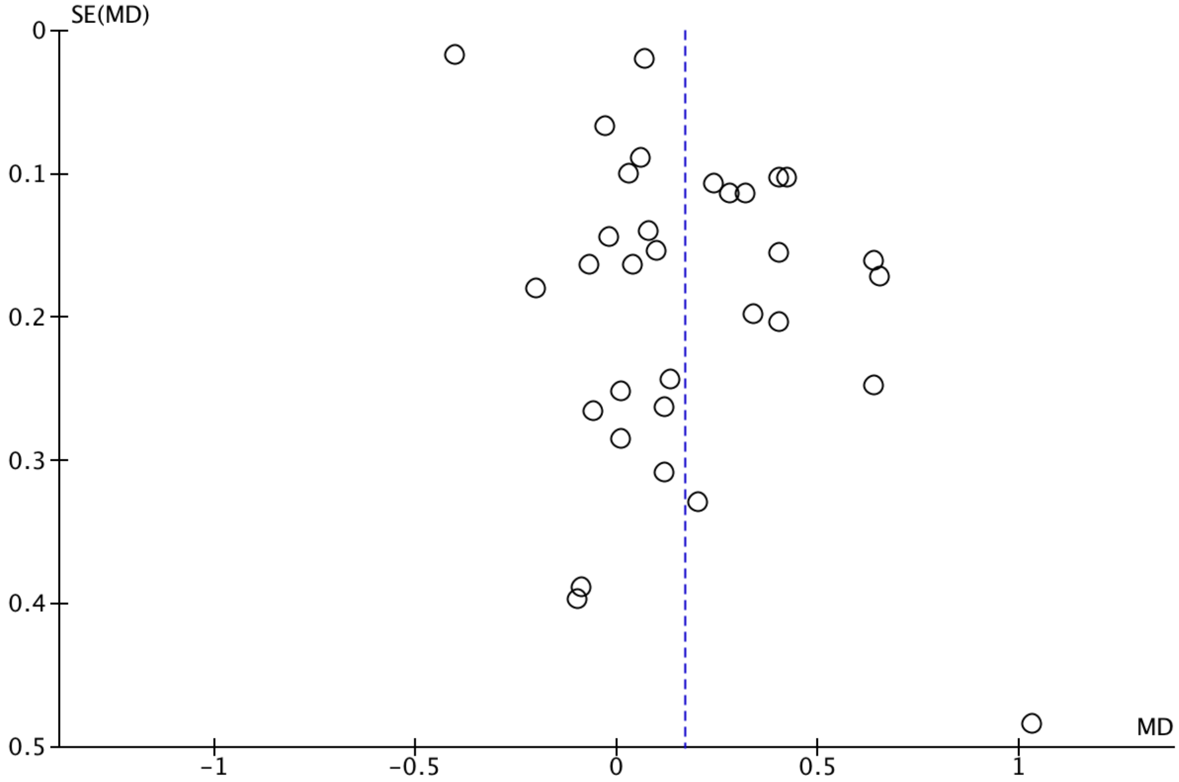

Online Appendix S8.3
Funnel plot regarding the effect of NSPT on DM compared to non-DM on PPD at incremental scores

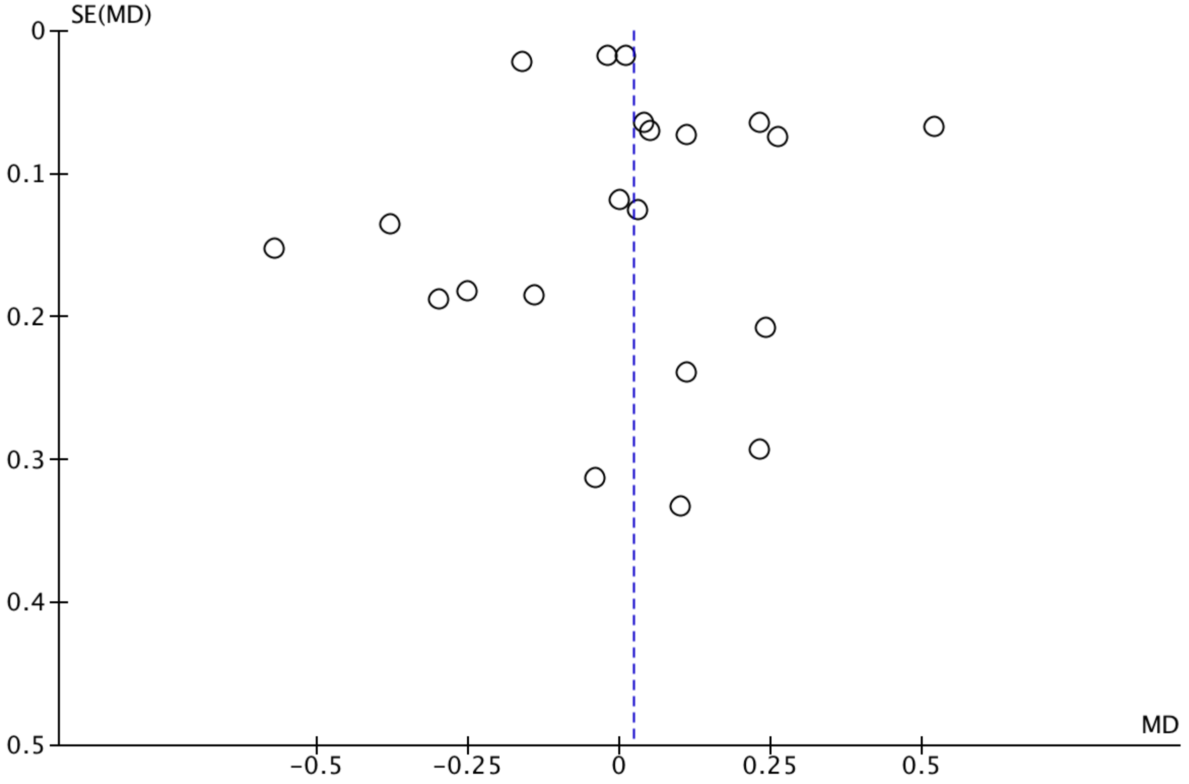

Online Appendix S8.4
Funnel plot regarding the effect of NSPT on DM compared to non-DM on CAL at baseline

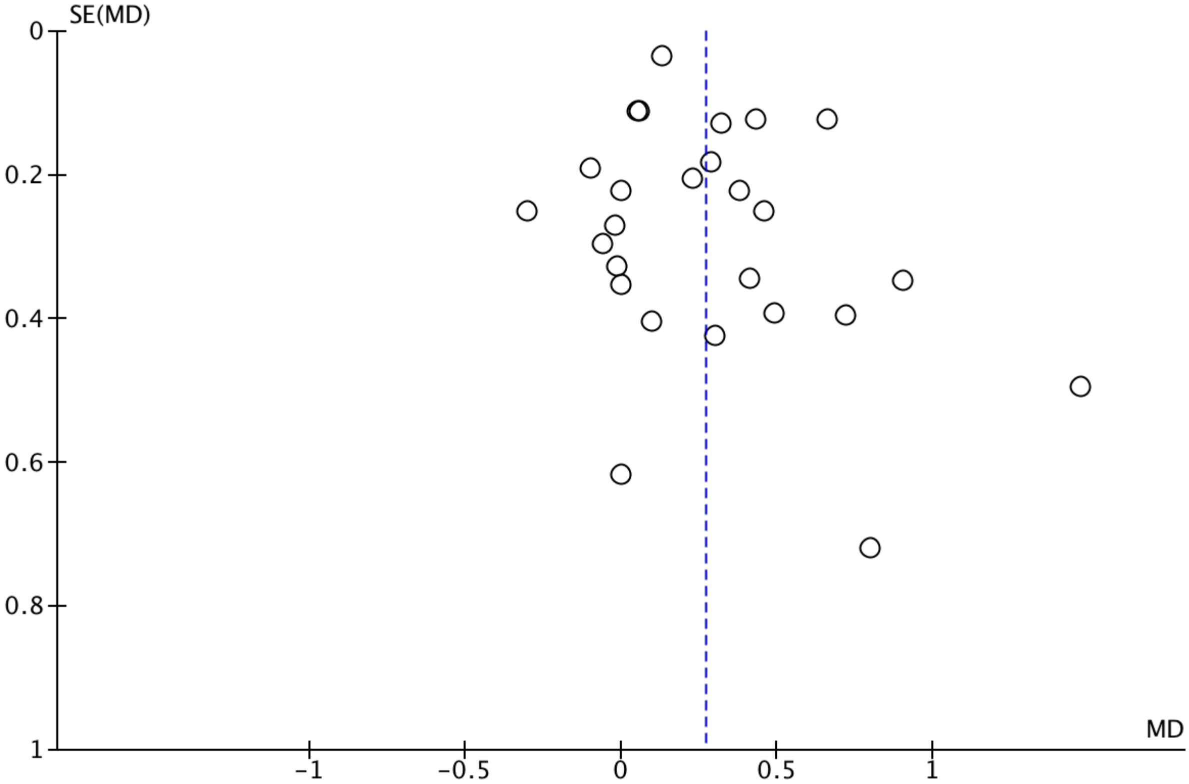

Online Appendix S8.5

Funnel plot regarding the effect of NSPT on DM compared to non-DM on CAL post-NSPT

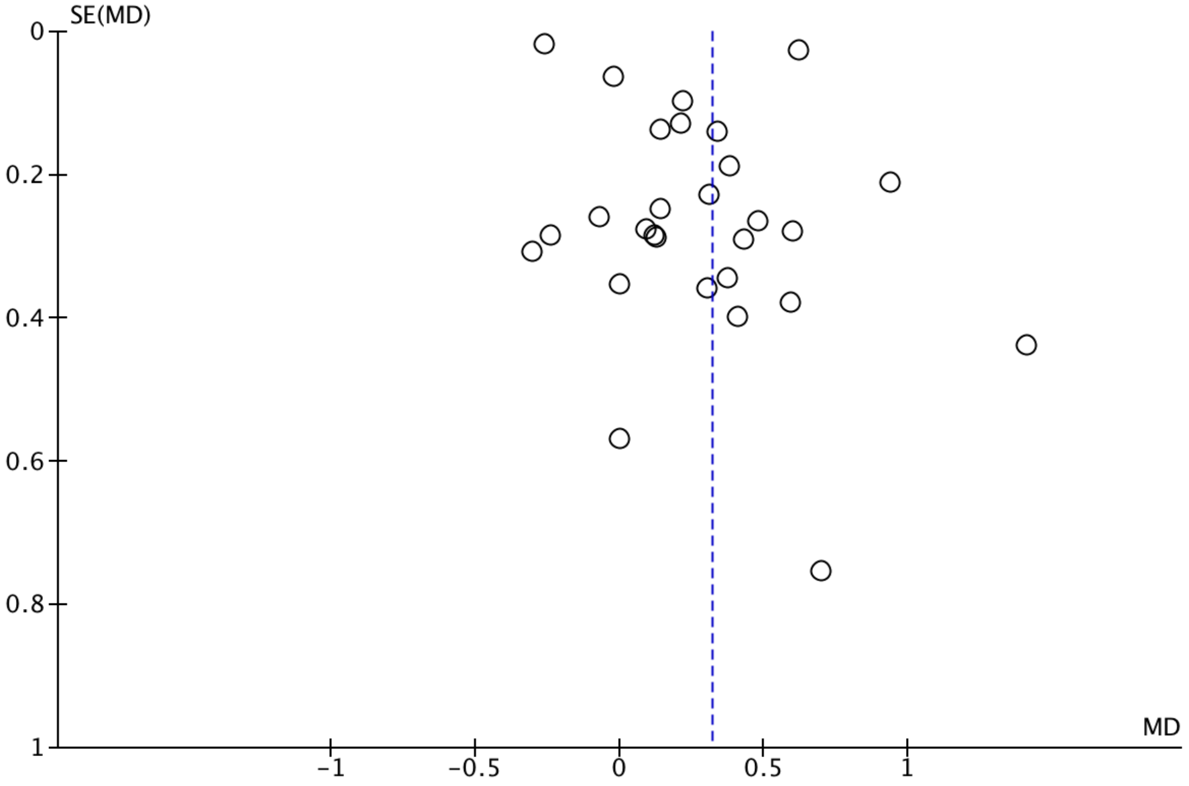

Online Appendix S8.6

Funnel plot regarding the effect of NSPT on DM compared to non-DM on CAL at incremental scores

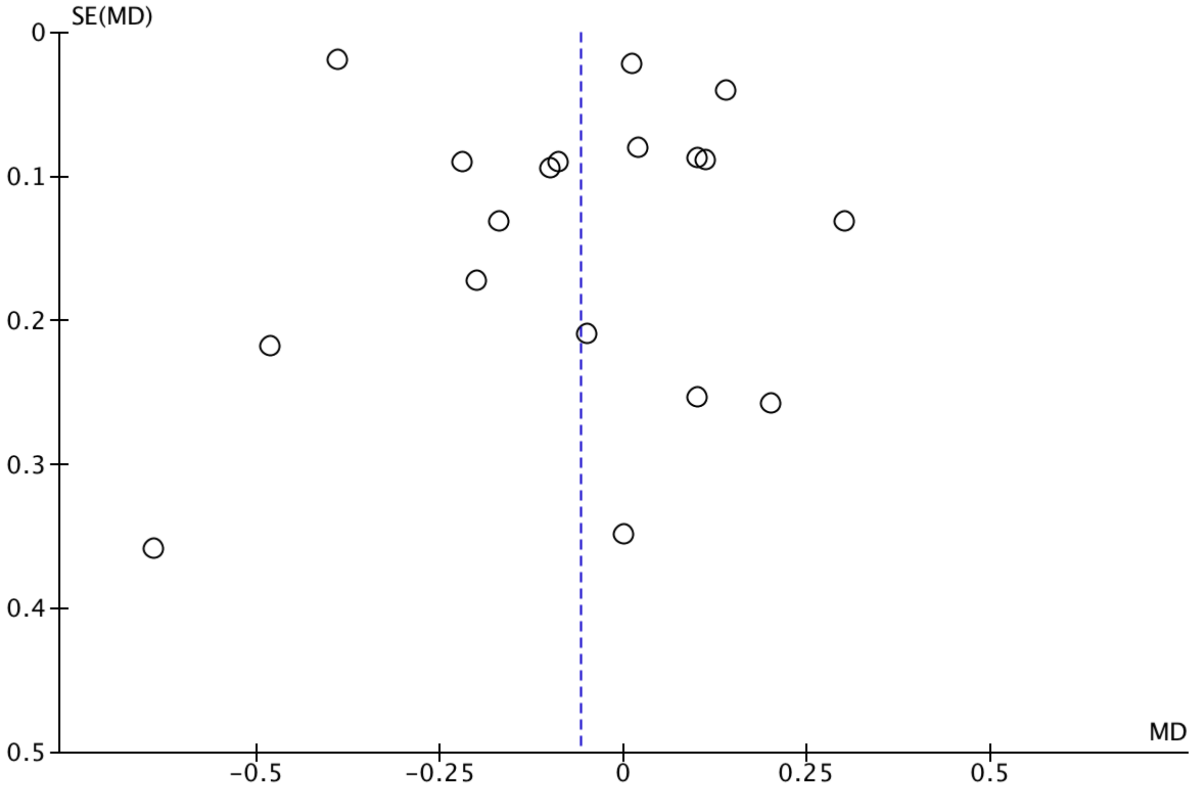

Online Appendix S8.7
Funnel plot regarding the effect of NSPT on DM compared to non-DM on GI at baseline

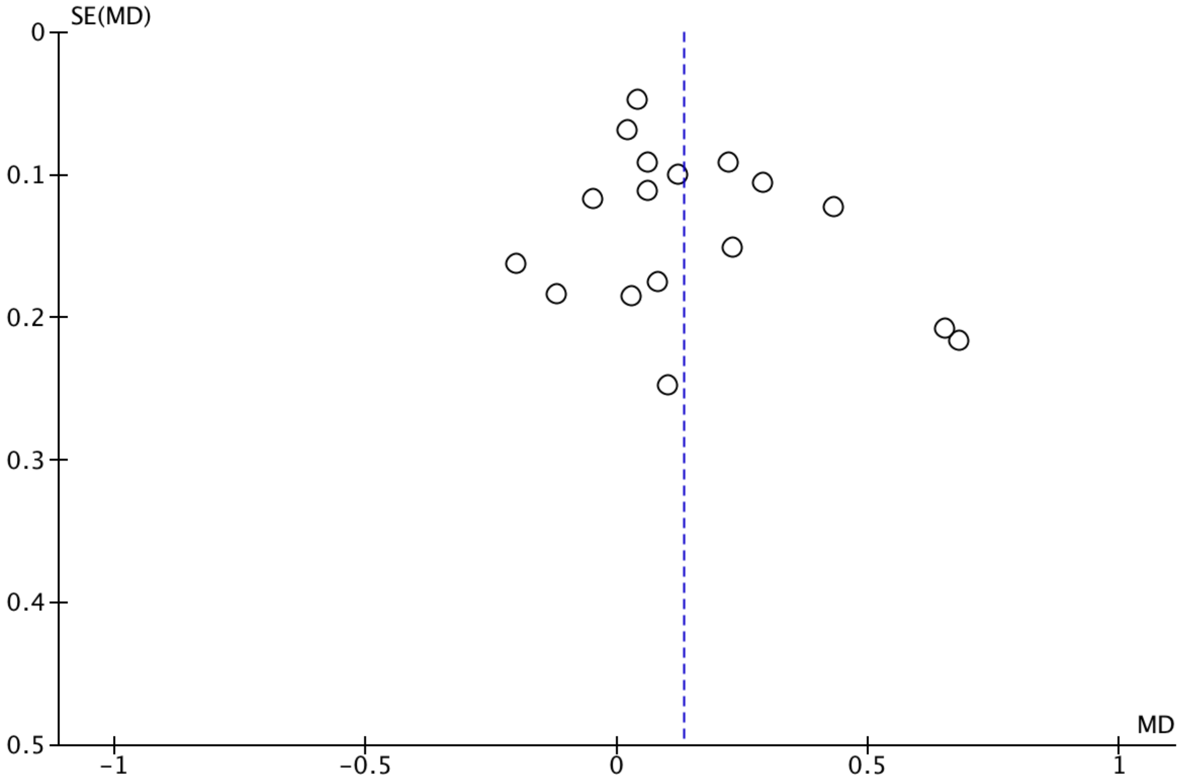

Online Appendix S8.8
Funnel plot regarding the effect of NSPT on DM compared to non-DM on GI post-NSPT

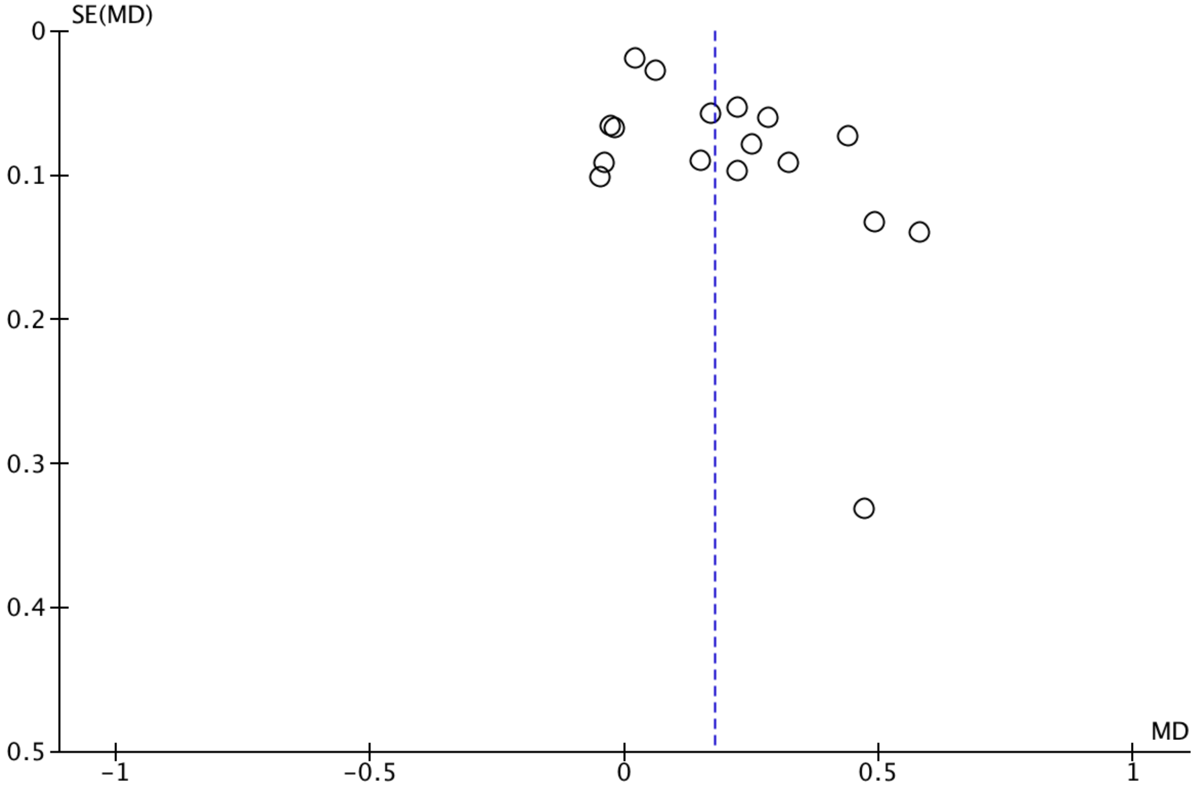

Online Appendix S8.9

Funnel plot regarding the effect of NSPT on DM compared to non-DM on BOP at baseline

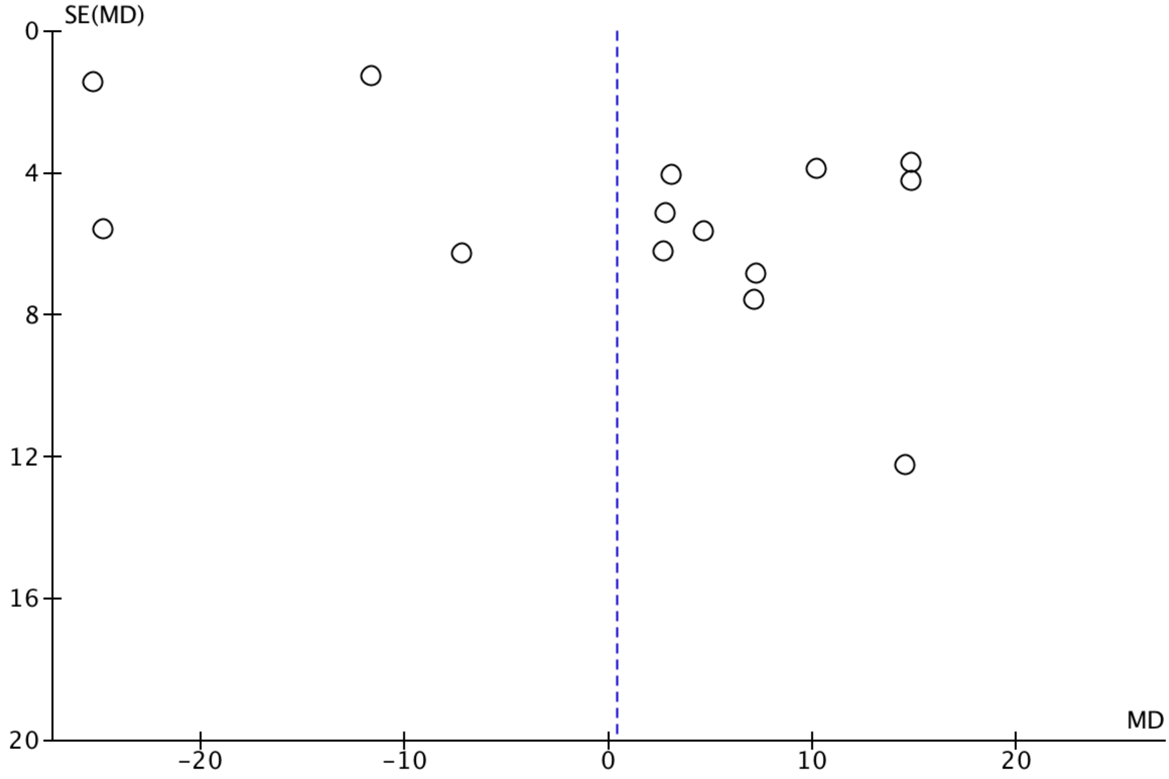

Online Appendix S8.10

Funnel plot regarding the effect of NSPT on DM compared to non-DM on BOP post-NSPT

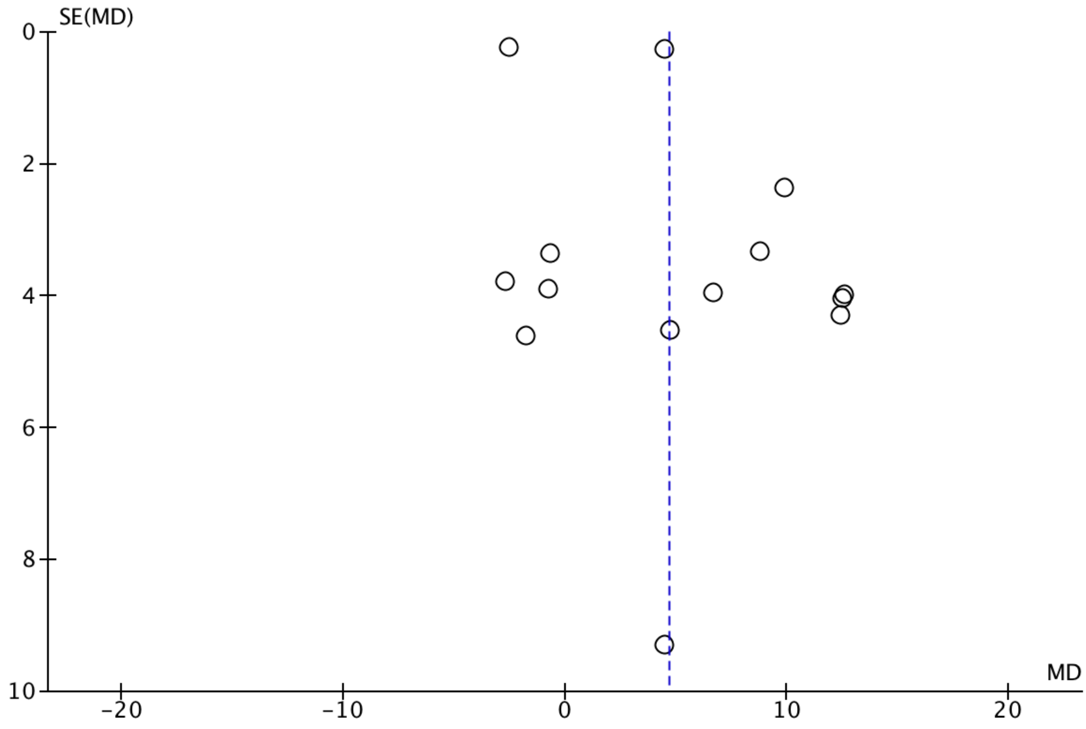

Online Appendix S8.11
Funnel plot regarding the effect of NSPT on DM compared to non-DM on BOP on incremental scores

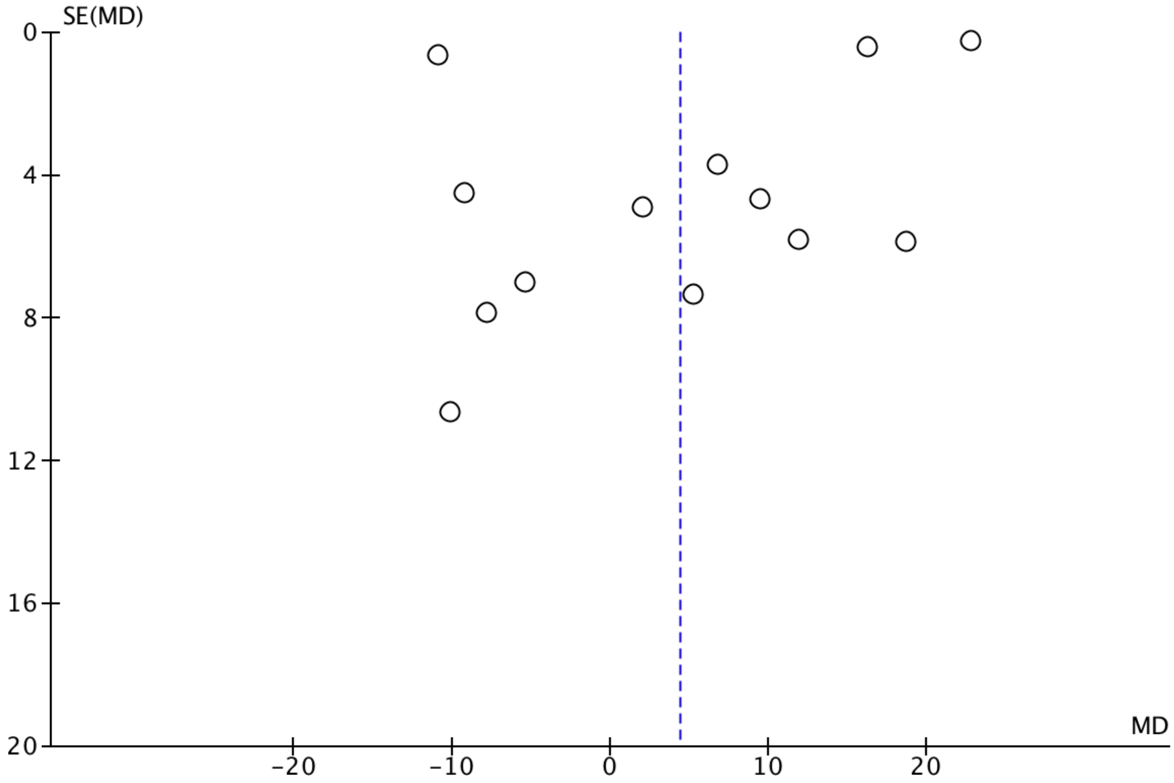

Online Appendix S8.12
Funnel plot regarding the effect of NSPT on DM compared to non-DM on plaque% at baseline

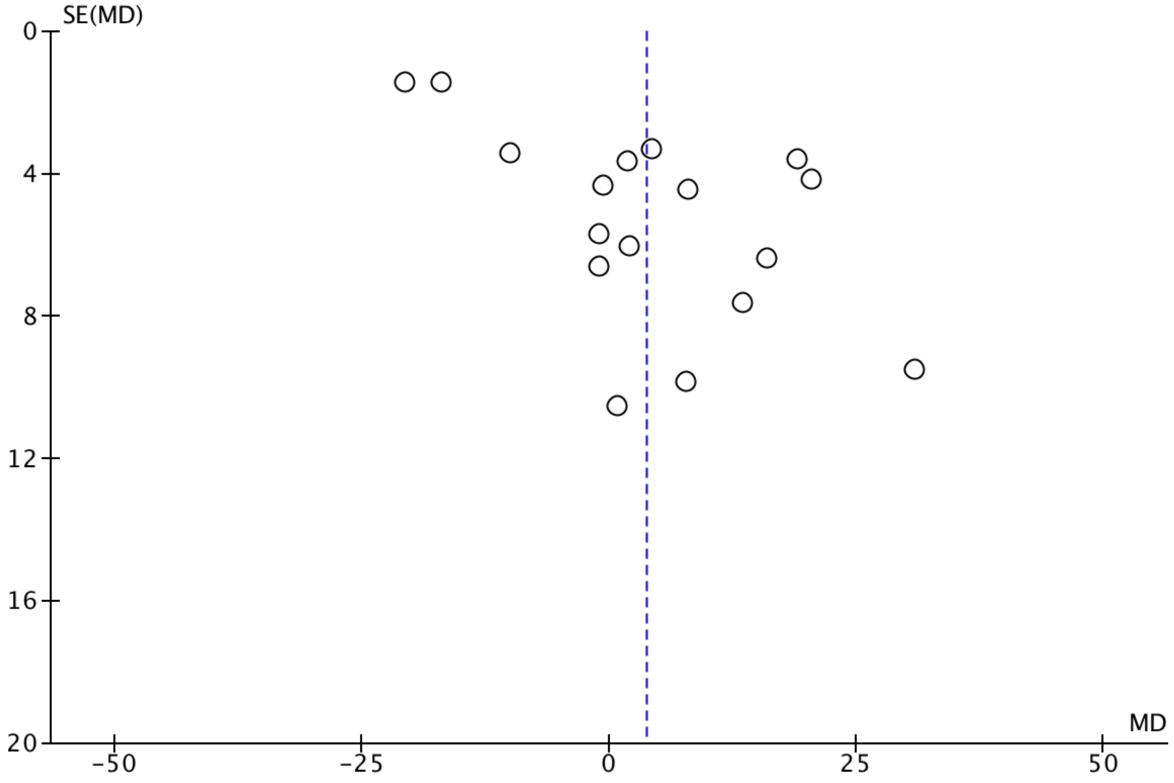

Online Appendix S8.13

Funnel plot regarding the effect of NSPT on DM compared to non-DM on plaque% post-NSPT

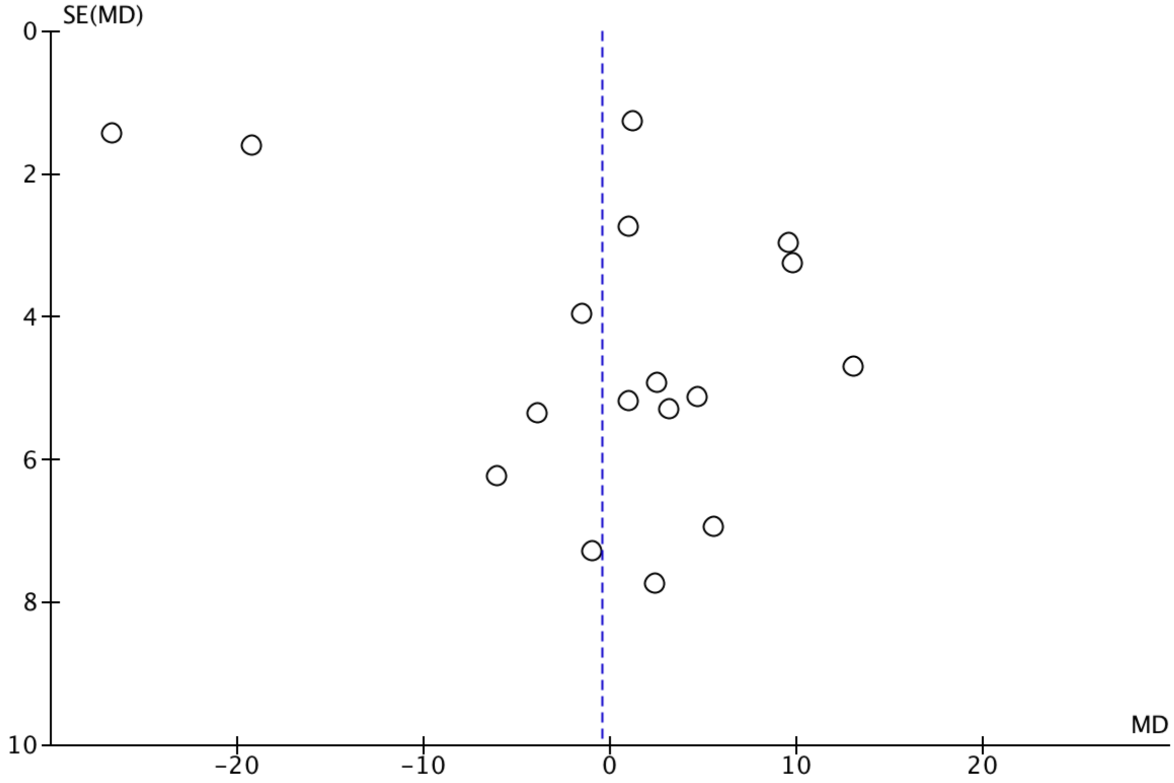

Online Appendix S8.14

Funnel plot regarding the effect of NSPT on DM compared to non-DM on plaque% on incremental scores

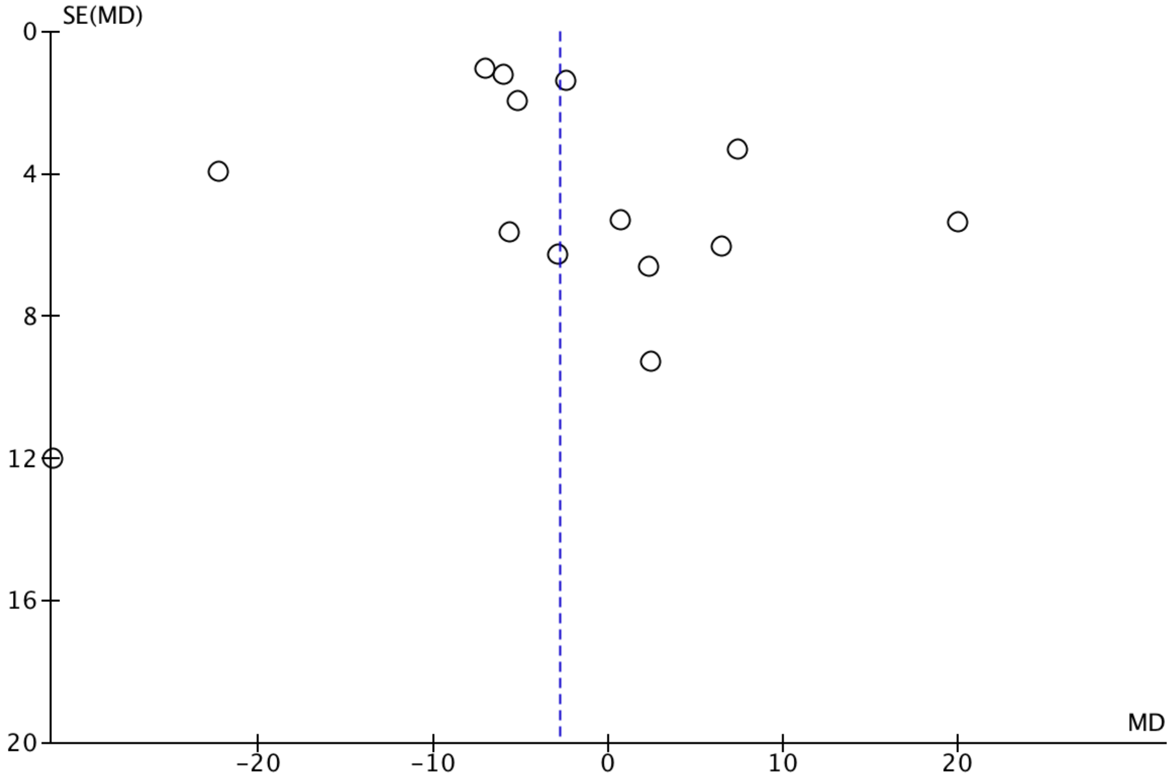

Online Appendix S8.15

Funnel plot regarding the effect of NSPT on DM compared to non-DM on PI (Silness-Loe) at baseline

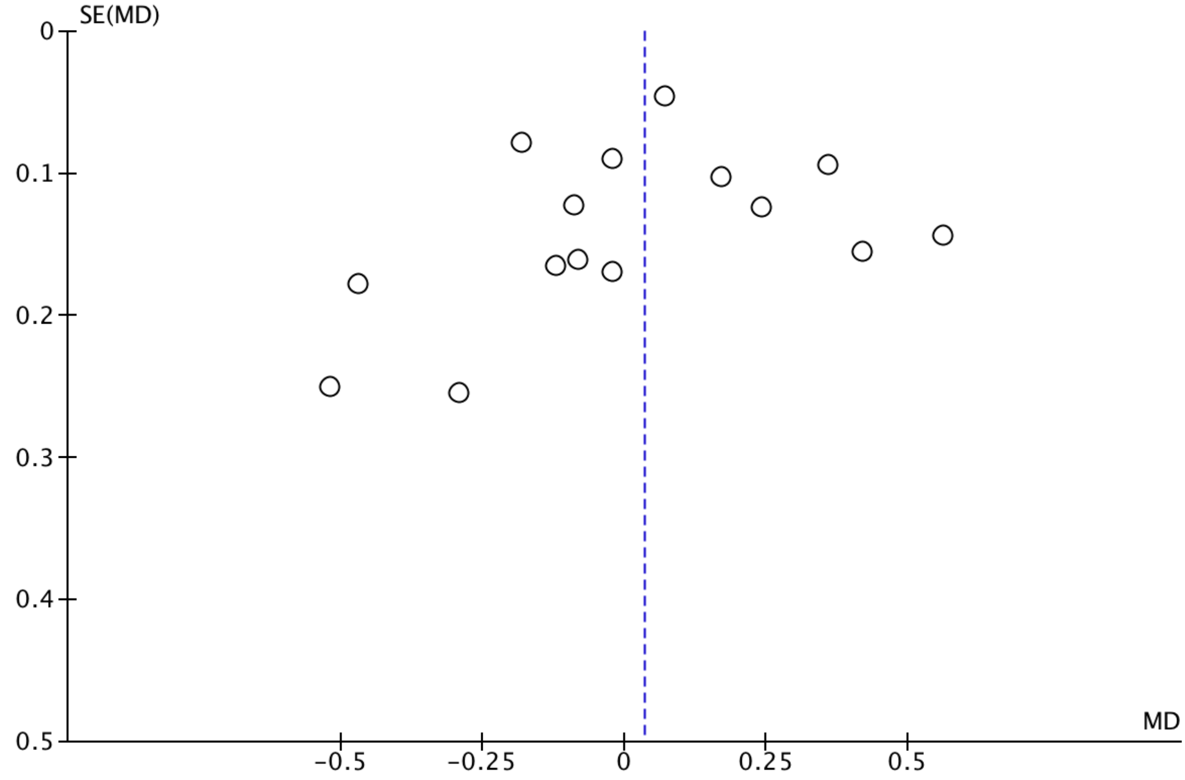

Online Appendix S8.16

Funnel plot regarding the effect of NSPT on DM compared to non-DM on PI (Silness-Loe) post-NSPT

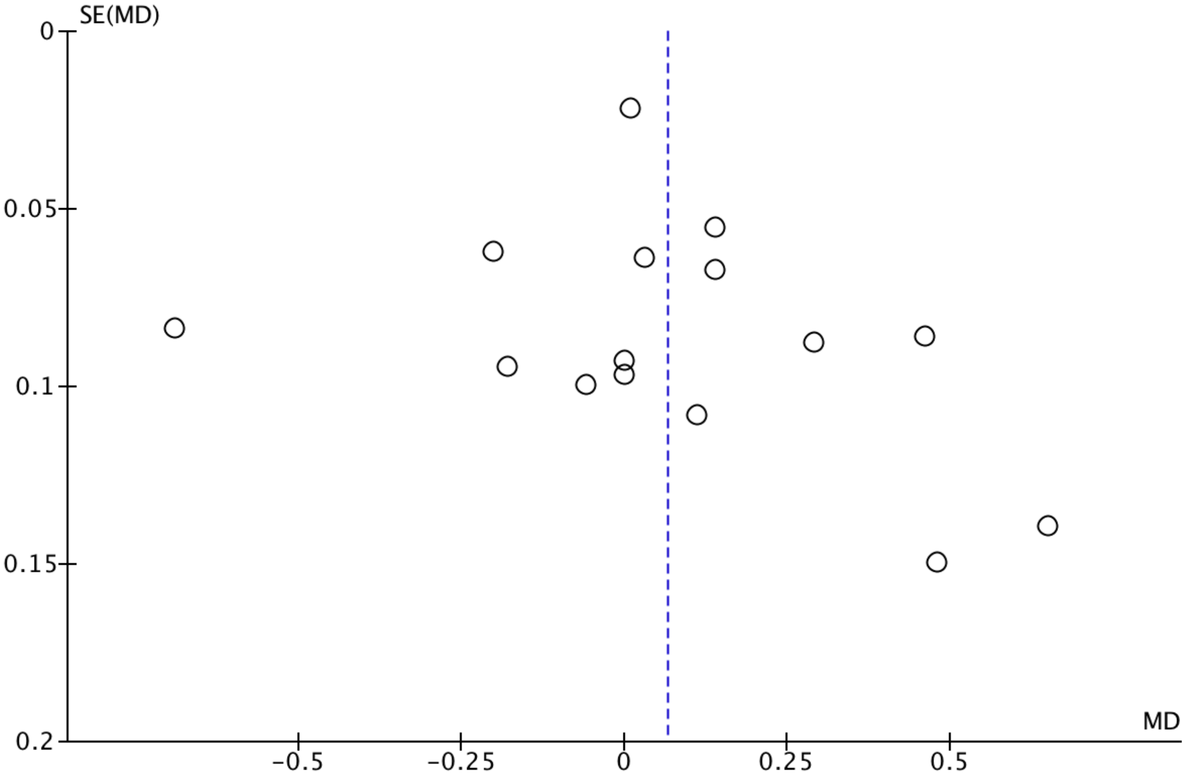

Online Appendix S8.17

Funnel plot regarding the effect of NSPT on good controlled DM compared to non-DM on PPD at baseline

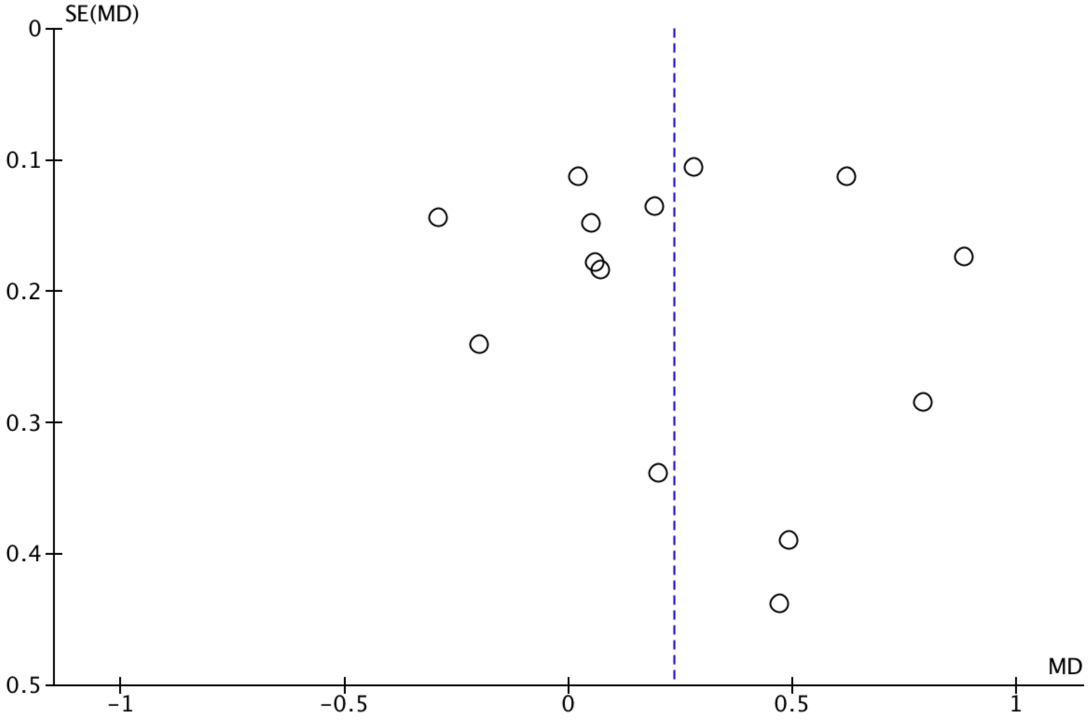

Online Appendix S8.18

Funnel plot regarding the effect of NSPT on good controlled DM compared to non-DM on PPD post-NSPT

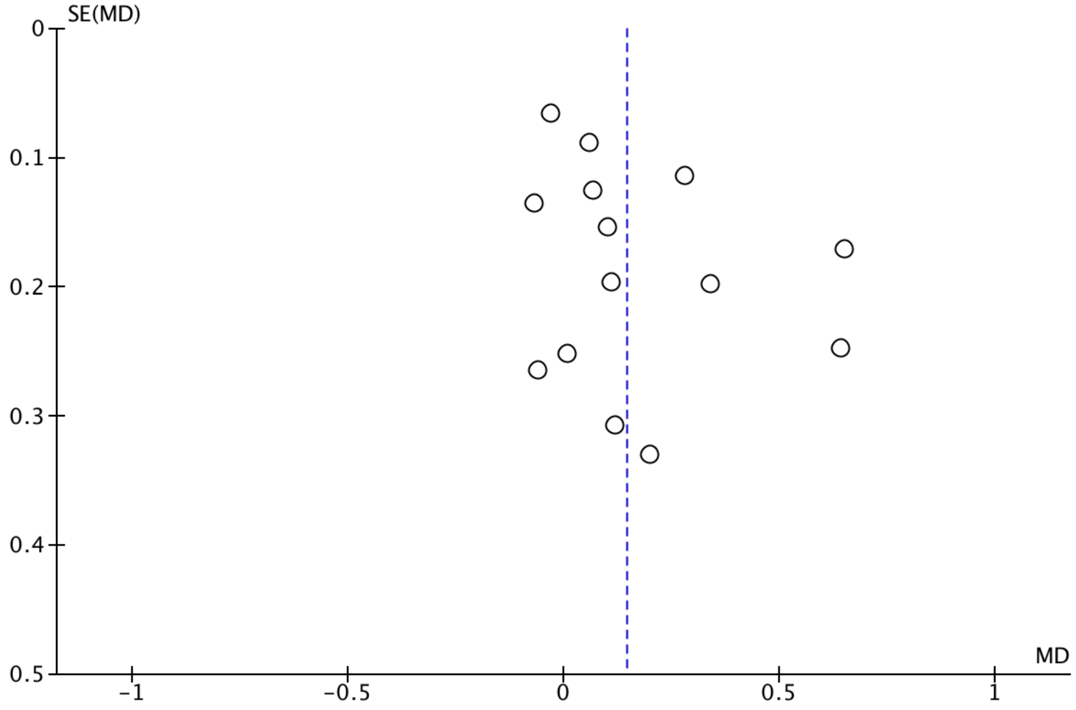

Online Appendix S8.19
Funnel plot regarding the effect of NSPT on good controlled DM compared to non-DM on CAL at baseline

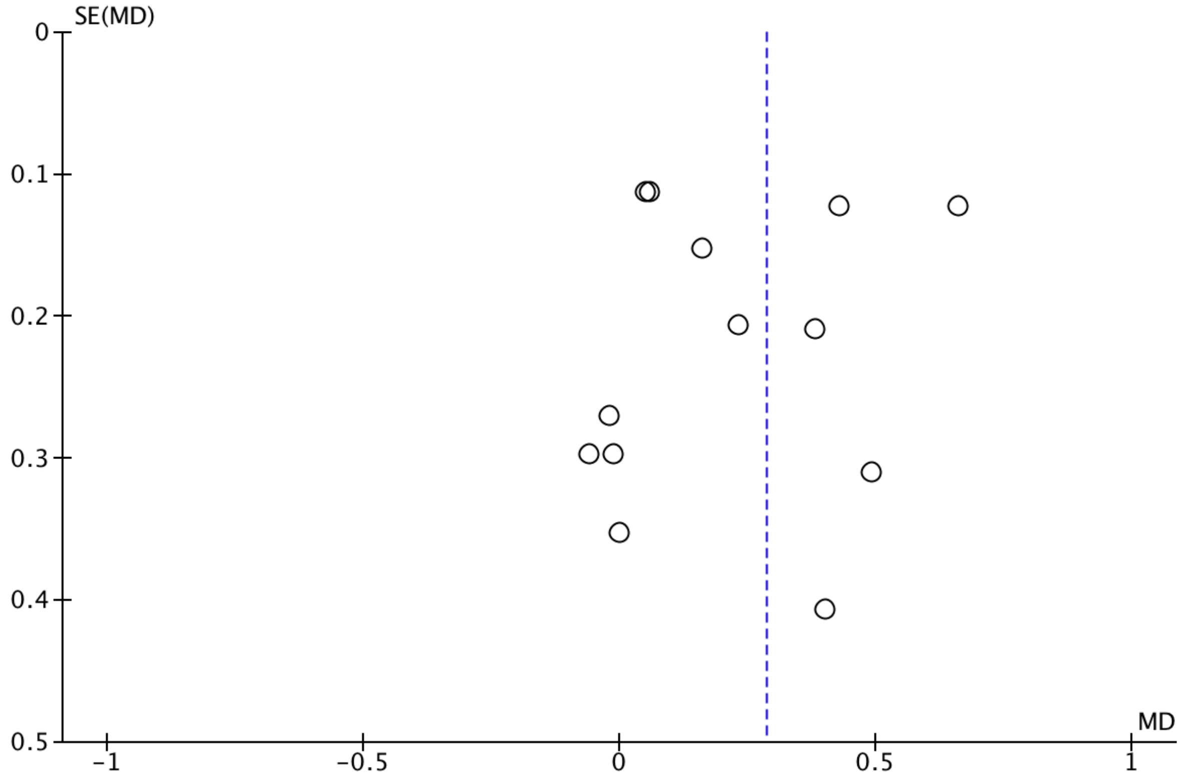

Online Appendix S8.20
Funnel plot regarding the effect of NSPT on good controlled DM compared to non-DM on CAL post-NSPT

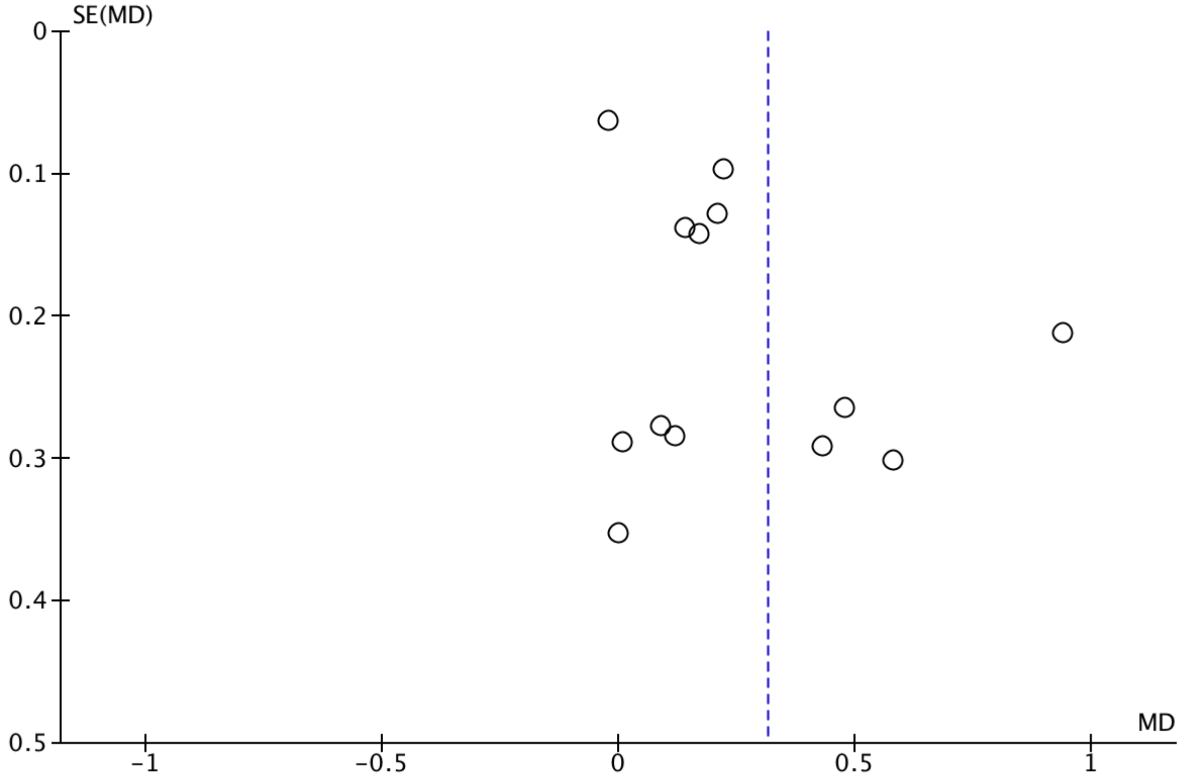

Online Appendix S9
MOOSE checklist

Reporting Criteria	Reported (Yes/No)	Reported on Page
**Reporting of Background**		
Problem definition	Yes	8
Hypothesis statement	Yes	8
Description of Study Outcome(s)	Yes	11
Type of exposure or intervention used	Yes	11
Type of study design used	Yes	8
Study population	Yes	9
**Reporting of Search Strategy**		
Qualifications of searchers (e.g., librarians and investigators)	Yes	9
Search strategy, including time period included in the synthesis and keywords	Yes	9
Effort to include all available studies, including contact with authors	Yes	8
Databases and registries searched	Yes	8
Search software used, name and version, including special features used (e.g., explosion)	Yes	8
Use of hand searching (e.g., reference lists of obtained articles)	Yes	8
List of citations located and those excluded, including justification	Yes	OA S1
Method for addressing articles published in languages other than English	Yes	OA S1
Method of handling abstracts and unpublished studies	Yes	OA S1
Description of any contact with authors	Yes	OA S1
**Reporting of Methods**		
Description of relevance or appropriateness of studies assembled for assessing the hypothesis to be tested	Yes	9+10
Rationale for the selection and coding of data (eg, sound clinical principles or convenience)	Yes	10-12
Documentation of how data were classified and coded (e.g., multiple raters, blinding, and interrater reliability)	Yes	10-12
Assessment of confounding (eg, comparability of cases and controls in studies where appropriate	Yes	10-13
**Reporting Criteria**		
Assessment of study quality, including blinding of quality assessors; stratification or regression on possible predictors of study results	Yes	10

Assessment of heterogeneity	Yes	11+12
Description of statistical methods (e.g., complete description of fixed or random effects models, justification of whether the chosen models account for predictors of study results, dose-response models, or cumulative meta-analysis) in sufficient detail to be replicated	Yes	12
Provision of appropriate tables and graphics	Yes	Table 1-4
**Reporting of Results**		
Table giving descriptive information for each study included	Yes	Table 2
Results of sensitivity testing (eg, subgroup analysis)	Yes	OA S7
Indication of statistical uncertainty of findings	Yes	Table 4
**Reporting of Discussion**		
Quantitative assessment of bias (e.g., publication bias)	Yes	OA S8
Justification for exclusion (e.g., exclusion of non–English-language citations)	Yes	OA S1
Assessment of quality of included studies	Yes	OA S3
**Reporting of Conclusions**		
Consideration of alternative explanations for observed results	Yes	19-22
Generalization of the conclusions (i.e., appropriate for the data presented and within the domain of the literature review)	Yes	19-22
Guidelines for future research	Yes	19-22
Disclosure of funding source	Yes	1

Online Appendix S10.1

PRISMA checklist

Section/topic	#	Checklist item	Reported on page #
**TITLE**			
Title	1	Identify the report as a systematic review, meta-analysis, or both.	1
**ABSTRACT**			
Structured summary	2	Provide a structured summary including, as applicable: background; objectives; data sources; study eligibility criteria, participants, and interventions; study appraisal and synthesis methods; results; limitations; conclusions and implications of key findings; systematic review registration number.	4
**INTRODUCTION**			
Rationale	3	Describe the rationale for the review in the context of what is already known.	6+7
Objectives	4	Provide an explicit statement of questions being addressed with reference to participants, interventions, comparisons, outcomes, and study design (PICOS).	8
**METHODS**			
Protocol and registration	5	Indicate if a review protocol exists, if and where it can be accessed (e.g., Web address), and, if available, provide registration information including registration number.	8
Eligibility criteria	6	Specify study characteristics (e.g., PICOS, length of follow-up) and report characteristics (e.g., years considered, language, publication status) used as criteria for eligibility, giving rationale.	8
Information sources	7	Describe all information sources (e.g., databases with dates of coverage, contact with study authors to identify additional studies) in the search and date last searched.	8
Search	8	Present full electronic search strategy for at least one database, including any limits used, such that it could be repeated.	Figure 1
Study selection	9	State the process for selecting studies (i.e., screening, eligibility, included in systematic review, and, if applicable, included in the meta-analysis).	9
Data collection process	10	Describe method of data extraction from reports (e.g., piloted forms, independently, in duplicate) and any processes for obtaining and confirming data from investigators.	10+11
Data items	11	List and define all variables for which data were sought (e.g., PICOS, funding sources) and any assumptions and simplifications made.	11

Risk of bias in individual studies	12	Describe methods used for assessing risk of bias of individual studies (including specification of whether this was done at the study or outcome level), and how this information is to be used in any data synthesis.	10
Summary measures	13	State the principal summary measures (e.g., risk ratio, difference in means).	12+13
Synthesis of results	14	Describe the methods of handling data and combining results of studies, if done, including measures of consistency (e.g., I^2) for each meta-analysis.	13

Section/topic	#	Checklist item	Reported on page #
Risk of bias across studies	15	Specify any assessment of risk of bias that may affect the cumulative evidence (e.g., publication bias, selective reporting within studies).	10
Additional analyses	16	Describe methods of additional analyses (e.g., sensitivity or subgroup analyses, meta-regression), if done, indicating which were pre-specified.	12+13
**RESULTS**			
Study selection	17	Give numbers of studies screened, assessed for eligibility, and included in the review, with reasons for exclusions at each stage, ideally with a flow diagram.	Figure 1
Study characteristics	18	For each study, present characteristics for which data were extracted (e.g., study size, PICOS, follow-up period) and provide the citations.	Table 2
Risk of bias within studies	19	Present data on risk of bias of each study and, if available, any outcome level assessment (see item 12).	Table 3, OA S3
Results of individual studies	20	For all outcomes considered (benefits or harms), present, for each study: (a) simple summary data for each intervention group (b) effect estimates and confidence intervals, ideally with a forest plot.	14-18
Synthesis of results	21	Present results of each meta-analysis done, including confidence intervals and measures of consistency.	Table 3, OA S7
Risk of bias across studies	22	Present results of any assessment of risk of bias across studies (see Item 15).	14
Additional analysis	23	Give results of additional analyses, if done (e.g., sensitivity or 1up analyses, meta-regression [see Item 16]).	17, Table 3, OA S7
**DISCUSSION**			
Summary of evidence	24	Summarize the main findings including the strength of evidence for each main outcome; consider their relevance to key groups (e.g., healthcare providers, users, and policy makers).	19
Limitations	25	Discuss limitations at study and outcome level (e.g., risk of bias), and at review-level (e.g., incomplete retrieval of identified research, reporting bias).	22
Conclusions	26	Provide a general interpretation of the results in the context of other evidence, and implications for future research.	19-22
**FUNDING**			

Funding	27	Describe sources of funding for the systematic review and other support (e.g., supply of data); role of funders for the systematic review.	1

Online Appendix S10.2

PRISMA checklist for abstract

Section and Topic	Item #	Checklist item	Reported (Yes/No)
**TITLE**			
Title	1	Identify the report as a systematic review.	Yes
**BACKGROUND**			
Objectives	2	Provide an explicit statement of the main objective(s) or question(s) the review addresses.	Yes
**METHODS**			
Eligibility criteria	3	Specify the inclusion and exclusion criteria for the review.	Yes
Information sources	4	Specify the information sources (e.g. databases, registers) used to identify studies and the date when each was last searched.	Yes
Risk of bias	5	Specify the methods used to assess risk of bias in the included studies.	Yes
Synthesis of results	6	Specify the methods used to present and synthesise results.	Yes
**RESULTS**			
Included studies	7	Give the total number of included studies and participants and summarise relevant characteristics of studies.	Yes
Synthesis of results	8	Present results for main outcomes, preferably indicating the number of included studies and participants for each. If meta-analysis was done, report the summary estimate and confidence/credible interval. If comparing groups, indicate the direction of the effect (i.e. which group is favoured).	Yes
**DISCUSSION**			
Limitations of evidence	9	Provide a brief summary of the limitations of the evidence included in the review (e.g. study risk of bias, inconsistency and imprecision).	Yes
Interpretation	10	Provide a general interpretation of the results and important implications.	Yes
**OTHER**			
Funding	11	Specify the primary source of funding for the review.	NA
Registration	12	Provide the register name and registration number.	NA
